# The evolution of genes encoding for green fluorescent proteins: insights from cephalochordates (amphioxus)

**DOI:** 10.1038/srep28350

**Published:** 2016-06-17

**Authors:** Jia-Xing Yue, Nicholas D. Holland, Linda Z. Holland, Dimitri D. Deheyn

**Affiliations:** 1 Institute for Research on Cancer and Aging, Nice (IRCAN), CNRS UMR 7284, INSERM U1081, Nice, France; 2 Marine Biology Research Division, Scripps Institution of Oceanography, UC San Diego, 9500 Gilman Drive, La Jolla, CA 92093, USA

## Abstract

Green Fluorescent Protein (GFP) was originally found in cnidarians, and later in copepods and cephalochordates (amphioxus) (*Branchiostoma* spp). Here, we looked for GFP-encoding genes in *Asymmetron*, an early-diverged cephalochordate lineage, and found two such genes closely related to some of the *Branchiostoma* GFPs. Dim fluorescence was found throughout the body in adults of *Asymmetron lucayanum*, and, as in *Branchiostoma floridae*, was especially intense in the ripe ovaries. Spectra of the fluorescence were similar between *Asymmetron* and *Branchiostoma*. Lineage-specific expansion of GFP-encoding genes in the genus *Branchiostoma* was observed, largely driven by tandem duplications. Despite such expansion, purifying selection has strongly shaped the evolution of GFP-encoding genes in cephalochordates, with apparent relaxation for highly duplicated clades. All cephalochordate GFP-encoding genes are quite different from those of copepods and cnidarians. Thus, the ancestral cephalochordates probably had GFP, but since GFP appears to be lacking in more early-diverged deuterostomes (echinoderms, hemichordates), it is uncertain whether the ancestral cephalochordates (i.e. the common ancestor of *Asymmetron* and *Branchiostoma*) acquired GFP by horizontal gene transfer (HGT) from copepods or cnidarians or inherited it from the common ancestor of copepods and deuterostomes, i.e. the ancestral bilaterians.

Green fluorescent proteins (GFPs) are useful reagents for measuring molecular and cellular properties, such as gene expression, protein-protein interactions, and protein turnover^1^,^2^,^3^. They are structurally complex, being dimers of a monomer consisting of eleven beta sheets arranged in a cylinder with the fluorophore in the center and alpha helices at the top and bottom of the cylinder. This motif is so unique that GFP forms its own protein class with no other known protein with a similar structure^4^. While most GFPs fluoresce green, there are also some structurally related molecules that emit at other wavelengths^5^,^6^,^7^,^8^. Paradoxically, although the biotechnological applications of these molecules are well understood, much less is known about the functions of endogenous GFPs in animal cells. GFP was initially discovered in a luminous jellyfish (phylum Cnidaria) in which blue luminescent light is absorbed by the chromophore of the GFP which consequently gained electronic energy and then subsequently relaxed as photons in green fluorescence^9^. Endogenous GFPs have been found in at least three-dozen cnidarians (many of them non-luminous), six non-luminous copepods, and three non-luminous cephalochordate species in the genus *Branchiostoma*^10^,^11^,^12^, although GFP can be found in non-fluorescent species as well (GFP is then a chromoprotein)^13^. Clearly the occurrence of fluorescence does not necessarily indicate a relationship to GFP. Indeed, many compounds and proteins can trigger fluorescence in invertebrates and also vertebrates^14^,^15^,^16^.

There has been much discussion about the possible ecological relevance of light production in marine invertebrates, whether or not GFP is involved^17^. In bioluminescent cnidarians generally, the emitted light has been implicated in warning, defense, or attraction of prey. Since many pelagic organisms including jellyfishes perform diel vertical migration in the water column^18^,^19^, it has been suggested anecdotally for luminous jellyfishes that GFP might help to adjust the wavelength of light emitted to make it most visible at a given depth—for instance, blue at depth and green nearer the sea surface. The functions of GFP in non-luminescent organisms remain enigmatic. Suggested functions include photoprotection, spectral optimization for photosynthesis by mutualistic dinoflagellates, and protective antioxidation^20^,^21^,^22^. In addition, there is some evidence that prey are attracted to predators fluorescing when their GFP is excited by the blue wavelengths of sunlight penetrating relatively shallow water^23^.

When GFPs were initially discovered in copepods and cephalochordates, it was not clear whether the molecules were acquired from the diet, inherited from a common bilaterian ancestor or acquired via horizontal gene transfer (HGT)^12^,^24^. Sampling of more copepods indicated that GFPs had diversified within the group but did not answer the question of whether GFP had entered this lineage by horizontal gene transfer^11^. The same question was raised for cephalochordates (amphioxus, also known as lancelets), which have many GFPs^12^,^25^. The evolutionary conundrum about GFP (inheritance from common ancestor vs. HGT) largely derived from the fact that in all species studied thus far, GFPs share high similarity at both the structural and sequence levels, and yet the evolutionary lineages having GFPs (namely cnidarians, copepods and cephalochordates) are very sparsely distributed across the tree of life and are very distantly related to one another.

To address whether GFPs in the cephalochordate genus *Branchiostoma* were acquired by HGT, we also examined them from *Asymmetron lucayanum,* the most distant cephalochordate relative of *Branchiostoma*. There are three known genera of cephalochordates, where the *Asymmetron* genus branched off from the clade comprising *Branchiostoma* and *Epigonichthys* at least 120 mya^26^,^27^. Our comparison reveals that the genera *Asymmetron* and *Branchiostoma* share clearly homologous GFP-encoding genes. Thus, a recent acquisition of GFP-encoding genes in *Branchiostoma* via HGT is unlikely; instead GFP-encoding genes were probably present in the ancestral cephalochordates, although it remains to be determined whether they were horizontally transferred (e.g., via food intake, or symbiosis) to the ancestral cephalochordates or were inherited from the ancestral bilaterians.

## Results

### Asymmetron lucayanum fluorescent display and emission spectra

**Figure 1:**
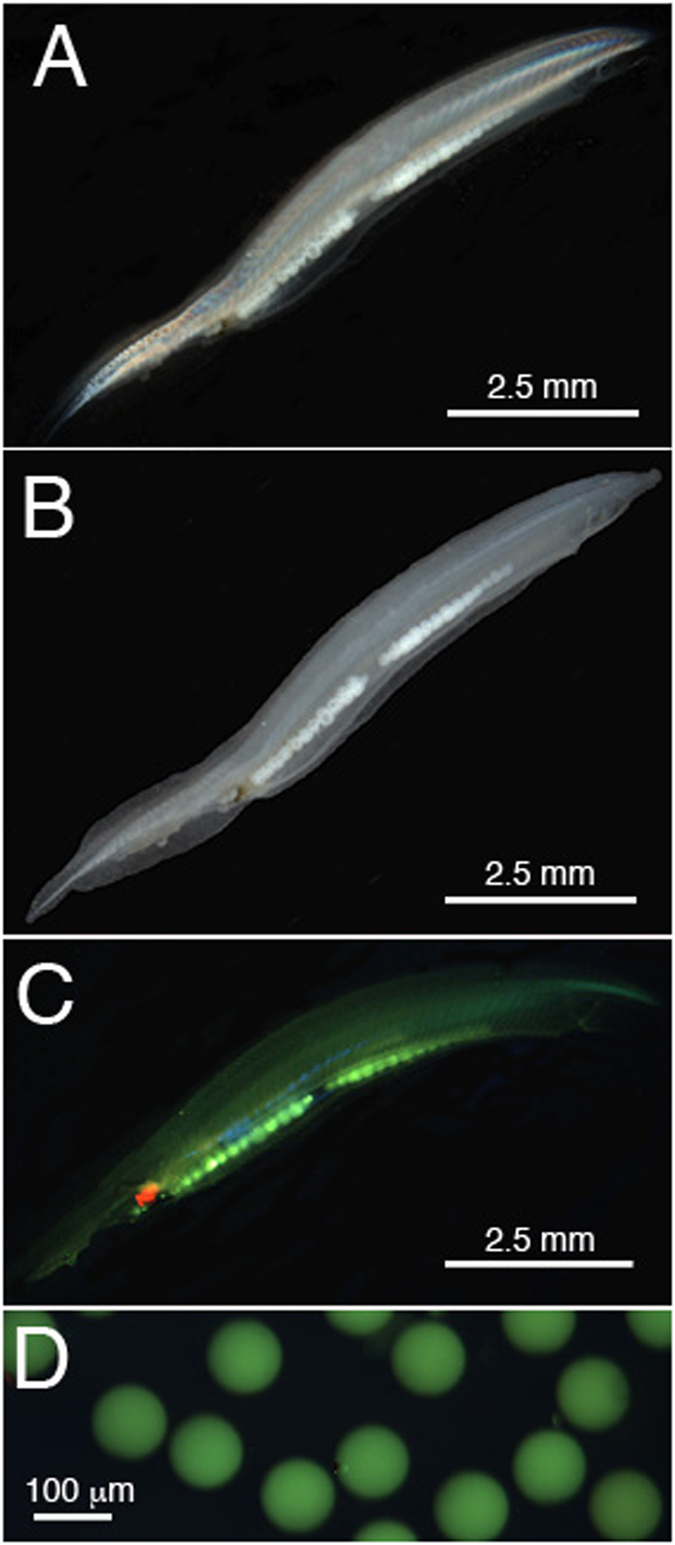
Imaging *Asymmetron lucayanum*. (**A–C**) Adult animal. (**A**) In bright field light with polarizer. (**B)** Under bright field light reflectance. (**C**) Under fluorescence excited at 470 nm. D. Egg fluorescence excited at 470 nm.

**Figure 2:**
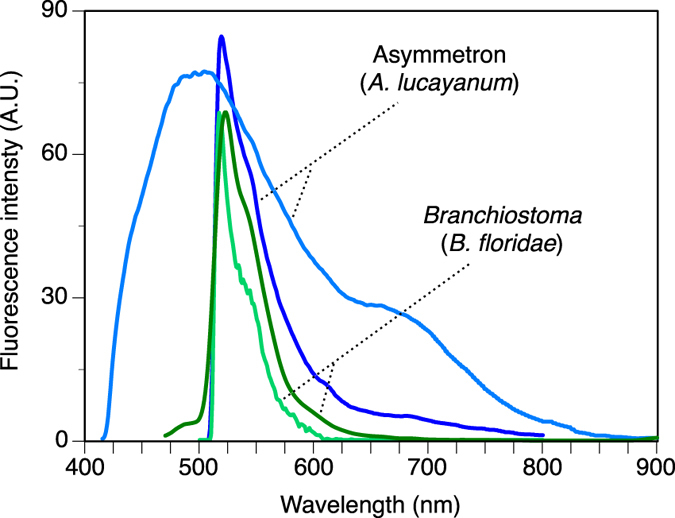
Fluorescence spectrum of cephalochordate GFPs excited at 470 nm (for both *Asymmetron lucayanum* and *Branchiostoma floridae*) and 390 nm (for *A. lucayanum* only). For the excitation at 470 nm, the *A. lucayanum* spectrum curve was shown in dark blue while the *B. floridae* specturm curve was shown in light green *B. floridae* GFPa) and darker green *B. floridae* GFPe). For the excitation at 390 nm, only the spectrum curve for *A. lucyanum* was shown (in light blue), since no fluorescence at that excitation wavelength was observed for *B. floridae*.

The notochord of *A. lucayanum* is iridescent under polarized light because the notochord cells are regularly spaced in a stack-of-coins arrangement that differentially refracts the incident light (Fig. 1A,B). In fluorescence mode (ex: 470 nm), dim green light was emitted diffusely throughout the body (in both genders) and was intense from the ripe ovaries (in females). Sometimes there was a red component at the distal end of the digestive tract, which was probably due to the chlorophyll in the algal diet (Fig. 1C). In fluorescence mode, the spawned eggs appeared bright green (Fig. 1D). The emission spectrum for the notochord, ovaries, and eggs had a sharp peak in the green (em: 525 nm), when excited at 470 nm, which was similar to *Branchiostoma floridae* (Fig. 2). In contrast, *A. lucayanum* also showed fluorescence when excited at 390 nm, with a broad blue-green spectrum, which was not observed for *B. floridae* (showing no fluorescence at all for that excitation). Excitation at 355 nm also triggered dim fluorescence in *A. lucayanum* (Fig. S1). Such blue fluorescence excitable at the shorter wavelength appears more specifically and more intensely in the eggs of *A. lucayanum.* This shorter-wavelength excitable fluorescence could originate from a variety of compounds other than GFP since broad fluorescence spectrum is typically not characteristic of any known GFP-family molecules. In cephalochordates however, some GFPs can produce fluorescence under a broader spectrum than the classic commercial GFP, such as described for the clade d GFPs in *B. floridae*^25^. One therefore cannot exclude that a GFP, or a maturation step of one of the *Asymmetron* GFPs (or its association with certain compounds) could lead to broader fluorescence under low excitation wavelength. This was also supported from the observation that in addition to the eggs having blue-green fluorescence, *Asymmetron* larvae also produce bright blue-shifted fluorescence (Fig. S1). This is consistent with the possible photoprotection against lower-wavelengths these life stages can be exposed to in the water column.

### GFP-encoding genes identified in A. lucayanum and other cephalochordates

In this study, we identified one and two GFP-encoding genes from the *A. lucayanum* adult and larval transcriptomes, respectively. Our gene orthology identification analysis suggests the GFP-encoding gene in the adult transcriptome is the ortholog of one of the two GFP-encoding genes in the larva transcriptome. Therefore, we used the two GFP-encoding genes identified in the larva transcriptome as the non-redundant set for GFP-encoding genes in *A. lucayanum*. It is unlikely that additional GFP-encoding genes are present in *A. lucayanum*, since the transcriptome assembly we used was the most complete available^27^; however, we cannot rule out such a possibility. A definitive answer will be available until the *A. lucayanum* genome eventually becomes entirely sequenced.

In parallel, we found 13 GFP-encoding genes in both the Asian amphioxus *Branchiostoma belcheri* and the Florida amphioxus *B. floridae*. When we compared the 13 *B. floridae* GFP-encoding genes identified in this study with the 16 *B. floridae* GFP-encoding genes reported earlier^25^, we found the 3 “missing” GFPs had been deleted in the v2.0 assembly of the *B. floridae* genome when the two haploid genomes of the v1.0 assembly were collapsed into a single representative one; thus the 3 missing genes are likely to be redundant. In addition, 21 GFP-encoding genes have been cloned for the European amphioxus *Branchiostoma lanceolatum* (all deposited in NCBI GenBank database). In sum, we compiled a total of 49 cephalochordate GFP-encoding sequences from four cephalochordate species, two from *A. lucayanum*, 13 from *B. belcheri*, 13 from *B. floridae* and 21 from *B. lanceolatum* (Table 1). The nucleotide coding sequences (CDSs) and protein sequences of these genes are provided in Supplementary file 1 and 2, respectively.

We compared the average sequence diversity of GFP-encoding genes within each cephalochordate species using four different measurements: nucleotide distance (D_JC_), protein sequence distance (D_Pois_), nonsynonymous substitution rate (D_n_) and synonymous substitution rate (D_s_). In general, the *Asymmetron* GFP-encoding genes showed consistently higher sequence diversity than GFP-encoding genes from *Branchiostoma* species based on different measurements (Table 2). Within the genus *Branchiostoma*, GFP-encoding genes from *B. belcheri* seem to show higher sequence diversity than their *B. floridae* counterparts, whereas the *B. lanceolatum* GFP-encoding sequences showed much lower average sequence diversity. Since those *B. lanceolatum* GFP-encoding sequences were not identified from a systematic genome-wide survey such as we performed for the other two *Branchiostoma* species, it is highly likely that these sequences come from a biased GFP-encoding gene sampling in this species. This may, explain the extremely low sequence diversity among these sequences compared with their counterparts from the other two *Branchiostoma* species.

### Phylogenetic relationship of cephalochordate GFP-encoding genes

**Figure 3:**
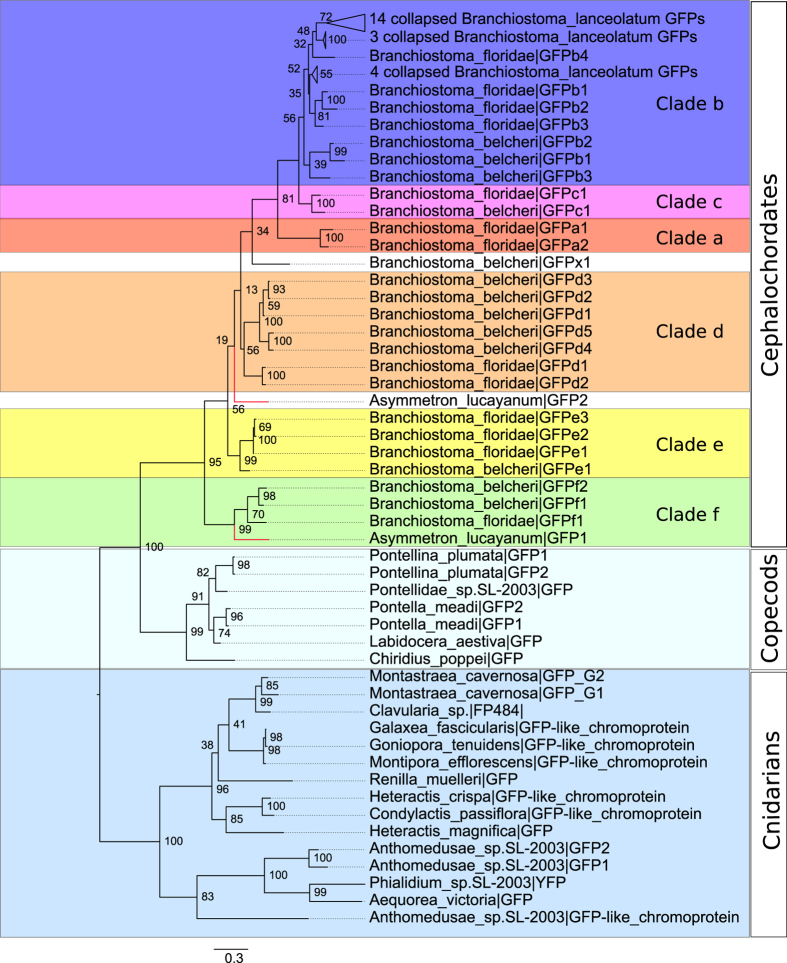
Phylogenetic tree of cephalochordate GFP-encoding genes, with cnidarian and copepod GFP-encoding genes as outgroups. Branches representing the two GFP-encoding genes from *A. lucayanum* are highlighted in red. Branches corresponding to *B. lanceolatum* GFP-encoding sequences were collapsed and the collapsed nodes were represented by triangles in the tree. For each internal node, the local support value was calculated by 100 rapid bootstrapping via RAxML. The clades are highlighted with colors previously designating *B. floridae* GFP clades (following our earlier study)^25^. The full version of this tree without collapsing the *B. lanceolatum* sequences is provided as Fig. S2.

In addition to the 49 cephalochordate GFP-encoding genes, we further incorporated 22 GFP-encoding genes from copepods and cnidarians as outgroups for the phylogenetic analysis (Fig. 3, Fig. S2 and Table S1). The trimmed sequence alignment used for this analysis is provided in Supplementary file 3. Overall, the tree showed that the cephalochordate GFPs have a closer affinity to those of copepods than to those of cnidarians (Figs 3 and S2). Within the cephalochordate GFP subtree, 47 out of 49 cephalochordate GFP-encoding genes were assigned to the six major clades (a–f) of cephalochordate GFPs previously demonstrated in *B. floridae*^25^, with the remaining two as unassigned (*A. lucayanum* GFP2 and *B. belcheri* GFPx1). One *A. lucayanum* GFP-encoding gene was positioned between clade d and clade e, whereas the other one fell unambiguously into clade f (Fig. 3). GFP-encoding genes from *B. belcheri* spread across clades b through f with only one member left as unassigned (Fig. 3), suggesting that clades b through f should have been established before the divergence of *B. floridae* and *B. belcheri*.

Interestingly, the unassigned *B. belcheri* GFP-encoding gene (*B. belcheri* GFPx1) encodes two other domains (class-A Low-density lipoprotein receptor domain and MFS/sugar transport protein domain) in addition to the GFP domain. One explanation is that this gene was mis-annotated during the original *B. belcheri* gene annotation by accidentally being joined to its flanking neighbors. However, our parallel phylogenetic analysis based on a modified gene model that does not contain those two additional domains placed this gene to the same phylogenetic position, suggesting the phylogenetic positioning of *B. belcheri* GFPx1 is not an artifact due to gene annotation error. All the 21 *B. lanceolatum* GFP-encoding sequences were tightly packed into clade b (Fig. 3), consistent with our previous conjecture that these closely sequences should come from strongly biased gene sampling in *B. lanceolatum*.

### Lineage-specific expansion of GFP-encoding genes in the Branchiostoma genus

**Figure 4:**
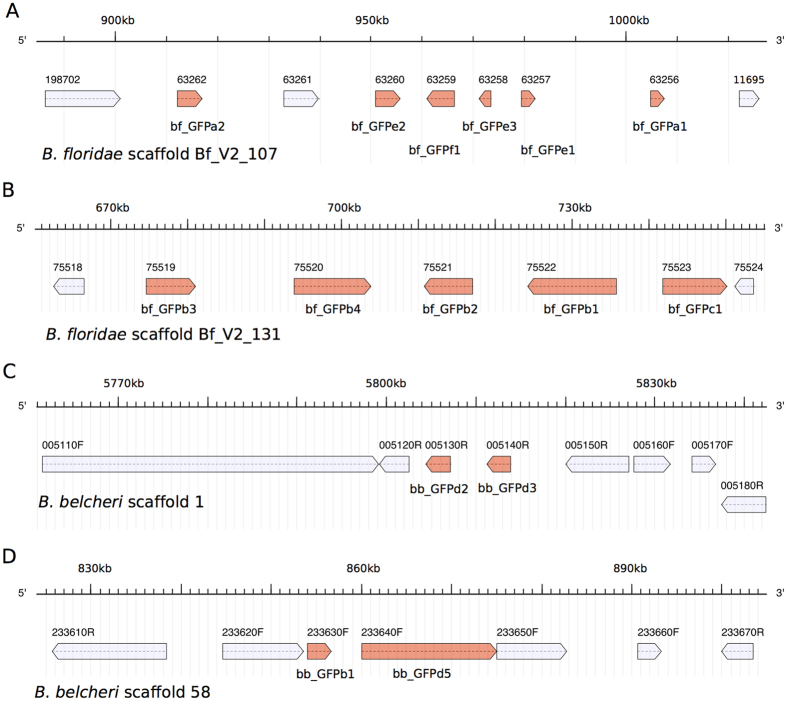
The tandemly duplicated gene clusters of GFP-encoding genes in *Branchiostoma floridae* and *Branchiostoma belcheri*. All the GFP-encoding genes were highlighted in red. (**A**) The genomic region with GFP-encoding gene tandem duplication on *B. floridae* scaffold Bf_V2_107. (**B**) The genomic region with GFP-encoding gene tandem duplication on *B. floridae* scaffold Bf_V2_131. (**C**) The genomic region with GFP-encoding gene tandem duplication on *B. belcheri* scaffold 1. (**D**) The genomic region with GFP-encoding gene tandem duplication on *B. belcheri* scaffold 58.

Compared with the earlier diverged *A. lucayanum*, copepod and even most cnidarians, the large number of GFP-encoding genes in *B. floridae* and *B. belcheri* seems to be the result of lineage-specific expansion. The lower average sequence diversity of *Branchiostoma* GFP-encoding genes compared with that of *Asymmetron* further supports this idea. Our phylogenetic analysis indicates that the starting condition for this expansion was the presence of at least five GFP genes corresponding to clade b through f (one for each clade) in the ancestral *Branchiostoma* species. The physical distribution of the *Branchiostoma* GFP-encoding genes (Table 1) suggests that at least some of the expansion resulted from tandem duplications. For example, there are 11 *B. floridae* GFP-encoding genes located in two tightly packed clusters: one 95.2 kb long (containing 6 genes) on scaffold Bf_V2_107 (Fig. 4A) and the other 75.4 kb long (containing 5 genes) on scaffold Bf_V2_113 (Fig. 4B). For *B. belcheri*, at least four GFP-encoding genes are attributable to tandem gene duplication (Fig 4C,D) while there could be even more since the current *B. belcheri* genome assembly is less complete than the *B. floridae* genome assembly.

### Purifying selection of GFP-encoding genes in cephalochordates

Expanding gene families can be shaped by diversifying selection when adaptive changes accumulate in different duplicated gene copies. Here, we assessed the selection imposed on cephalochordate GFP-encoding genes by measuring non-synonymous/synonymous substitution rate ratio (D_n_/D_s_). Neutral theory predicts D_n_/D_s_ = 1 in the absence of natural selection, whereas deviations from this null model will suggest either diversifying selection (D_n_/D_s_ > 1) or purifying selection (D_n_/D_s_ < 1). For each cephalochordate species, we consistently observed D_n_/D_s_ < 1 for all the GFP-encoding paralogs (Table 2), suggesting a general purifying selection scheme for GFP-encoding genes in cephalochordates despite the formation of different phylogenetic clades. Theoretically, it is possible that some specific codon sites could evolve under diversifying selection while purifying selection is shaping the evolution of the rest of the gene. However, no such sites were identified as statistically significant based on hypothesis testing of codeml’s site models (M1a vs. M2a and M7 vs. M8). We further examined selection scheme for different clades (b–f) based on *B. floridae*-*B. belcheri* (co-)orthologs^28^ within each individual clade. The clade a was excluded since not identified in GFP-encoding genes from *B. belcheri*. Apparently, D_n_/D_s_ values were more elevated in clades b and d. In combination with their higher inter-specific sequence divergence (D_JC_ and D_Pois_), this indicates likely relaxation of purifying selection in these two clades (Table 3). Interestingly, they also have the most lineage-specific duplication events, suggesting that functional redundancy, due to recent gene duplication, might help relax the selection constraints in these two clades.

### Evolutionary relationships between cephalochordate GFPs and those of other major clades

**Figure 5:**
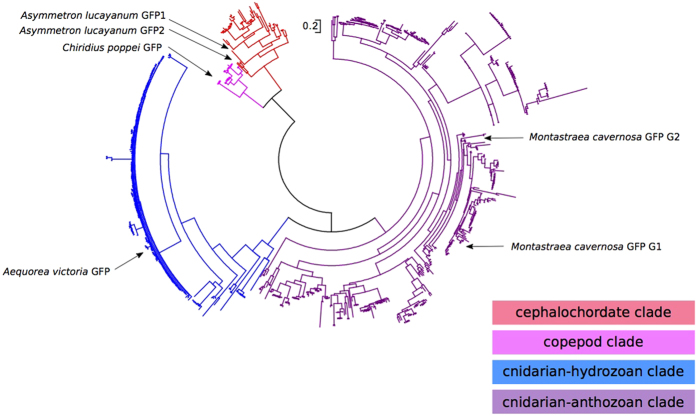
Phylogenetic relationship of cephalochordate GFPs relative to those in other evolutionary lineages. The major taxonomic categories are cephalochordates (red), copepods (magenta), hydrozoan cnidarians (blue) and anthozoan cnidarians (purple). The phylogenetic position of GFP-encoding genes from representative species (one for each clade) of each clade was indicated.

As Fig. 3 already shows, cephalochordate GFP-encoding genes formed their own phylogenetic group and are more closely related to those of copepods than to those of cnidarians. However, we cannot assess the possibility that there might be some unsampled copepod or cnidarian GFP-encoding genes that are closely related to cephalochordate ones, especially considering the small sample size of copepod and cnidarian outgroups considered in Fig. 3. To address this more comprehensively, we examined our data in the context of the entire National Center for Biotechnology Information (NCBI) non-redundant (nr) database of 1,832 GFP-encoding sequences. We manually reviewed the taxonomic information of these 1,832 GFP-encoding sequences and noticed that 1,128 of them are labeled as artificial constructs or recombinant vectors. These GFPs are lab-modified versions of natural GFPs (most of them are based on the same GFP: *Aequorea victoria* GFP). In addition, there are 22 GFPs in the nr database that were annotated with various origins (six from virus, seven from bacteria, one from oomycete parasite, five from protist parasites, one from mosquito, one from rat and one from human) but all of them are probably due to artificially introduced GFP vectors. There are 50 GFPs remaining with no traceable taxonomic origins. The 39 cephalochordate GFPs in the nr database were replaced with our 49 better-curated cephalochordate GFPs. This left us with a total of 642 GFP-encoding sequences with seemingly natural origins (174 from hydrozoan cnidarians, 403 from anthozoan cnidarians, 16 from copepods, and 49 from cephalochordates). The phylogenetic tree based on these sequences (Fig. 5) reveals three well-diverged clades: namely cephalochordates, copepods and cnidarians (with the last being divided into distinct hydrozoan and anthozoan subclades). This more comprehensive analysis emphasizes that the cephalochordate clade has clearly diverged from the copepod and cnidarian clades while the copepod and cephalochordate clades are closer to each other than either is to the cnidarian clade. The trimmed sequence alignment used for this analysis is provided in Supplementary file 4.

**Figure 6:**
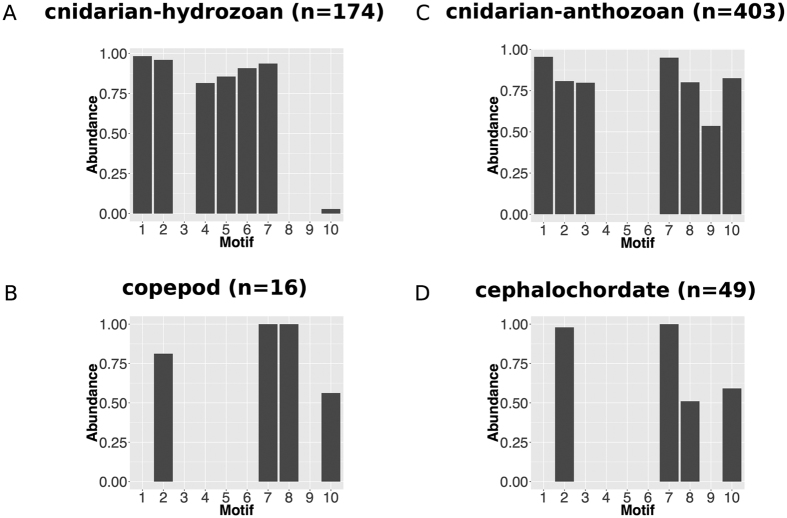
The conserved motif composition of GFP domains from different evolutionary lineages. The relative abundance of ten compositional motifs of the GFP domain was shown for hydrozoan cnidarians (**A**), anthozoan cnidarians (**B**), copepods (**C**), and cephalochordates (**D**). The numbers in the parenthesis indicate the total GFP-encoding sequences from the corresponding evolutionary lineage that were used in the analysis.

To compare the GFP domains from different evolutionary lineages in more detail, we further identified the top ten most conserved amino acid motifs (Table 4) within the GFP domain region and plotted their relative abundance (proportion of the sequences with this motif) in each evolutionary lineage (hydrozoans, anthozoans, copepods and cephalochordates) (Fig. 6A–D). In general, the GFP domains from all four evolutionary lineages show clear differences in overall conserved motif composition, reflecting lineage-specific changes of their GFP domains in their respective evolutionary histories. Consistent with what we observed from the phylogenetic tree, the cephalochordate and copepod lineages share more similarity in their motif composition compared with two cnidarian lineages, which once again suggests much closer evolutionary relationship of GFP-encoding genes from these two lineages relative to those cnidarian GFP-encoding genes.

## Discussion

### The origin of GFP-encoding genes in cephalochordates

This study is the first demonstration of GFP-encoding gene evolution within the cephalochordate clade*. A. lucayanum* contains two GFP-encoding genes, in contrast to about a dozen such genes in each of two species in the genus *Branchiostoma*. Lineage-specific expansion of GFP-encoding genes was observed for the genus *Branchiostoma*, probably due at least in part to tandem duplication. Both of our phylogenetic and conserved motif analyses have emphasized that the sequences of the GFP-encoding genes of hydrozoans, anthozoans, and cephalochordates differ considerably among these four evolutionary lineages. Such marked sequence divergence does not favor the idea that there was recent HGT from one of these groups to the next—for instance, from cnidarians to cephalochordates.

Inheritance of GFP-encoding genes from a common ancestor is more likely, with three possible alternatives. First, GFP-encoding genes emerged from a common ancestor to cnidarians, copepods and cephalochordates, which should be indicative of GFP present in other early bilatarian lineages. We screened the proteomes of the sea urchin *Strongylocentrotus purpuratus* (Echinodermata), the acorn worm *Saccoglossus kowalevskii* (Hemichordata) and the sea snail limpet *Lottia gigantea* (Mollusca) and found no sequence with close similarity to GFP. It is still possible however that other representatives of these groups could have GFP-like genes, and looking at luminous species within these groups might resolve this question.

A second possibility is that HGT transfer did occur, but from some animal phyla containing GFP-encoding genes yet to be discovered. This speculation would then suggest that such an animal would have particular ecological relationships with the current groups of organisms with GFP-encoding genes—that is either being an important component of the diet, or a common symbiont or parasite.

Third, GFPs independently arose multiple times in different evolutionary lineages and then diversified within each of these three evolutionary lineages. The scattered taxonomic distribution of currently known GFP-encoding genes appears to favor this hypothesis. This scenario however seems less likely in light of the elaborate and unique structure of GFP proteins, which is specifically tuned to absorb and possibly re-emit light (not all GFP proteins produce fluorescence). If the specific functions of independently evolved GFP proteins had to be kept across different taxa, it is likely that the different group of organisms would have come up with different solutions to cover these functions. As an analogy, production of visible light through bioluminescence has appeared independently about 30–40 times across taxa during evolution, each time using different sets of proteins and molecules to produce light^17^. Clearly there is a need for a more thorough search for GFP-encoding genes throughout the animal kingdom, especially in luminescent but also non-luminescent taxa that have not previously attracted the attention of photobiologists.

### The anatomical localization of fluorescence in cephalochordates could allow multiple ecological and/or biochemical functions

All cephalochordates so far assayed for fluorescence have conspicuous amounts of GFP proteins in their oocytes and spawned eggs. Then, during the subsequent embryonic and larval development, the GFP fluorescence is progressively lost, but in adults of *Branchiostoma*, remains strong in localized anatomical regions^12^. However in *Asymmetron* it remains diffusely distributed throughout the adult body (this study). The reason for the high concentrations of GFP signal in early developmental stages of cephalochordates is not known, although one possibility is photoprotection. Indeed, the embryos and larvae live planktonically in relatively shallow water, where they are sometimes captured during daylight hours^29^; under such conditions GFP would absorb blue (high-energy, possibly damaging) light and transform it to some extent into green (non-damaging) fluorescence. In any case, the main (most intense and sharper) spectrum of fluorescence for *Asymmetron* has a profile close to that of *Branchiostoma* GFPs from clade d or e, which is likely produced by *A. lucyanum* GFP2 although with a different amino acid sequence around the chromophore (Table 1). A previous study^25^ considered the *Branchiostoma* clade f GFP proteins as chromoproteins given that their spectra were too weak to be measured. In *Asymmetron*, it is possible that the broad blue-shifted weak spectrum is produced by *A. lucayanum* GFP1, which is also located in clade f in our phylogenetic analysis. This is not a rigorous demonstration, but is reasonable as the amino acid sequences are identical around their respective chromophore (Table 1).

Bomati *et al.*^30^ has shown that the amino acid sequence around the chromophore is critical for the fluorescence capacity of GFP, with a few changes changing the quantum efficiency from 0.1–100%^30^. The GFP chromophore sequence itself has been widely preserved through different taxa, and consistently reported as “GYG”, with the third glycine essential for the chromophore formation and fluorescence. The GYG reflects the electro- and stereo-chemical stability of this triplet allowing efficient energy transfer into fluorescence output, and found so far in all GFP templates^31^,^32^. In *B. floridae*, two GFPs with the GYA chromophore sequence were described in clade b (Bf GFPb1 and Bf GFPb2), although not associated with any detectable fluorescence. Also, there are two GFPs in *B. belcheri* with AYG (Bb GFPb2) and GFG (Bb GFPd5) chromophore sequences. These indicate that the general GFP protein motif could be preserved while performing different sets of biological/biochemical functions depending on the chromophore sequence, in association with fluorescence, but not necessarily.

In adults, differences in the fluorescent body regions between *A. lucayanum* and *Branchiostoma* spp are particularly striking—the chief site of fluorescence is diffuse distribution through the body in the former (present results) and restricted to the oral cirri of the latter^12^. All cephalochordates burrow shallowly in soft substrata with their anterior ends just within the burrow opening. There the mouth sucks in overlying sea water containing food particles that include motile planktonic organisms smaller than about 100 μm in diameter^33^. In the three species of *Branchiostoma* for which GFPs have been studied^12^, the oral cirri surrounding the mouth are highly fluorescent. The recent finding that jellyfish attracted prey with GFP fluorescence^23^ suggests that green light emanating from amphioxus cirri might attract motile planktonic prey, thus increasing their chance of being entrained in the feeding current entering the mouth. The stimulus for the fluorescence is ambient blue light in shallow sea water, which fits with species of *Branchiostoma* living from the shoreline to fairly moderate depths (max. ca. 100 m); by contrast, *A. lucayanum*, a species with non-fluorescent oral cirri, can be found in shallow water, but is more often captured at considerable depths, up to 1,000 m^34^.

It would be interesting to see if the oral cirri are fluorescent in a wider sample of *Branchiostoma* species and are non-fluorescent in additional species of *Asymmetron*, and in the single known species in the cephalochordate genus *Epigonichthys*, which is sister to *Branchiostoma*. Moreover, the idea that capture of small phototrophic prey items is enhanced by the fluorescence of the oral cirri in *Branchiostoma* species could be tested experimentally by manipulating the wavelengths of incident light impinging on the feeding animals. The data presented here, therefore, offer a new set of tools to address both the evolution and function of GFP in nature, which has largely been ignored.

## Methods

### Animal collection and fluorescent imaging

The Bahamas lancelet, *Asymmetron lucayanum*, was collected in Bimini, Bahamas^35^,^36^, and cultured and spawned in the laboratory according to previously described protocol^37^. Adults and unfertilized eggs were imaged in bright field and fluorescence under a Nikon SMZ 1500 stereoscope, equipped with a digital color QI camera. Fluorescence spectra were acquired using the PARISS hyperspectral imaging system (LightForm Inc.) mounted on a Nikon 80i microscope and spectra were generated in Excel and Deltagraph (Red Rock Inc.). All filters used were LP for all excitation wavelengths. These excitation wavelengths included 355, 390, 436, 470 nm, as per filter cubes commercially available from Nikon.

### The identification of GFP-encoding genes in cephalochordates

Yue and colleagues^27^ constructed two non-redundant *A. lucayanum* transcriptome assemblies, respectively, from adult and larval libraries with protein-coding gene predictions. The details of the transcriptome assembly, redundancy removal, protein-coding gene annotation was described here^27^. The predicted coding DNA sequences (CDSs) and proteome sets based on these two *A. lucayanum* transcriptome assemblies was used in this study. We further added the CDSs and proteomes of *B. floridae*^38^ sequences (based on v2.0 assembly) and *B. belcheri*^39^ (based on v18h27.r3 assembly) for our GFP search. We used proteinortho (v5.11) with default settings^40^ to identify orthologous relationship among the cephalochordate proteomes that we used in this study. For each cephalochordate proteome, we used hmmsearch (option: -E 1e-4) from the hmmer (v3.1b2) package to search for all GFP-encoding genes based on the hidden Markov model of the GFP domain (PF01353) curated by the Pfam database (v27.0). The GFP-encoding genes that we identified from the two *Asymmetron* proteome sets were further collapsed based on the orthology identified by proteinortho. For each orthologous groups, the longest sequence was selected for the downstream analysis. In addition, existing *B. lanceolatum* GFP-encoding sequences deposited in NCBI GenBank was further added into our final cephalochordate GFP-encoding gene set after verifying their protein domains by the hmmer package.

### Sequence alignment and phylogenetic reconstruction

In addition to the cephalochordate GFP-encoding genes that we compiled, we added 22 more GFP-encoding genes from copepods and cnidarians (including both hydrozoans and anthozoans) from GenBank as outgroups (Fig. S2 and Table S1). The protein sequences of these GFP-encoding genes were aligned by PROMALS3D^41^ with default settings. PROMALS3D searches against known protein structures and uses both structural and sequence constraints to generate highly accurate protein sequence alignment. The corresponding CDS sequence alignment was generated based on the PROMALS3D protein sequences alignment by PAL2NAL (v14)^42^ with default setting for later analysis. The PROMALS3D protein sequences alignment was further trimmed by trimAl (v1.4) (option: -gt 0.75)^43^ for phylogenetic analysis. We employed RAxML (v 8.2.6)^44^ for maximum likelihood (ML) phylogenetic tree construction with automatic model selection (model = PROTGAMMAAUTO) with 100 fast bootstrapping tests (option: -# 100) to assess topology stability. The final tree was visualized in FigTree (v1.4.2) (http://tree.bio.ed.ac.uk/software/figtree/). The different GFP clades were highlighted in different color to correspond to *B. floridae* GFP clades (a through f) defined in our earlier study^25^. In addition, during our phylogenetic analysis, we noticed that one *B. belcheri* GFP gene model (GFPx1: 276530F) has unusual domain composition and phylogenetic positioning. In order to test whether these anomalies are artifacts due to gene annotation error. We extracted the genomic sequence of this region together with 30 kb flanking region on both sides to run *de novo* gene annotation using FGENESH^45^ (organism specific gene-finding parameters: *B. floridae*). The resulting gene model was used to re-run our phylogenetic analysis described above for testing if such gene annotation change will affect the phylogenetic positioning of this gene.

### Calculation of evolutionary rates

The Jukes-Cantor model^46^ and the Poisson model^47^ were used to calculate nucleotide (D_JC_) and amino acid substitution rate (D_Pois_). The Nei-Gojobori model^48^ with Jukes-Cantor correction was used to calculate nonsynonymous substitution rate (D_n_) and synonymous substitution rate (D_s_). The CDS nucleotide alignment of cephalochordate GFP-encoding genes was used in this analysis. All such evolutionary rate calculation was performed in MEGA 9v6.06-mac^49^ with “pairwise deletion” option selected for alignment gap handling.

### Detection of sites under diversifying (positive) selection

We used the codeml program from the PAML package^50^ (v.4.8a) to detect sites under diversifying (positive) selection based on the CDS nucleotide alignment of GFP-encoding genes within each cephalochordate species. The alternative codon model M2 and M8 were compared with null model M1 and M7 respectively. The statistical significance of potential positively selected sites was assessed by Bayes empirical Bayes (BEB) analysis^51^.

### Screening and analyzing GFP-encoding sequences from the NCBI nr database and other bilaterian proteomes

The NCBI nr database was downloaded (ftp://ftp.ncbi.nih.gov/blast/db/FASTA/) and all of its GFP-encoding sequences were identified by hmmsearch (option: -E 1e-4). The taxonomic origins of these sequences were mapped by MEGAN (v5.10.5)^52^ based on NCBI’s Gl (GenInfo identifier) number. By manual inspection, we eliminated all artificial constructs, recombinant vectors, as well as data with no traceable taxonomic information. After replacing the cephalochordate GFP-encoding sequences in the nr database with our better-curated sequences (two from *A. lucayanum,* 21 from *B. lanceolatum,* and 13 each from *B. floridae* and *B. belcheri*), sequence alignment, alignment trimming, and tree building were carried out by the method already described. We used FigTree (v1.4.2) to highlight the tree branches based on the taxonomic origin of the corresponding sequences.

In addition, we retrieved proteomes of several representative early-diverged bilaterian animals including sea urchin *Strongylocentrotus purpuratus* (Echinodermata), the sea snail limpet *Lottia gigantea* (Mollusca) and the acorn worm *Saccoglossus kowalevskii* (Hemichordata) from EnsemblMetazoa (http://metazoa.ensembl.org) (for *S. purpuratus* and *L. gigantea*) and Metazome v3.0 (www.metazome.net) (for *S. kowalevskii*). GFP-encoding gene was screened by hmmsearch (option: -E 1e-4) for these proteomes.

### Characterizing conserved motif composition for the GFP domain

To characterize and compare conserved motif composition of the GFP domain in different evolutionary lineages. For each GFP-encoding gene investigated in this study, we performed hmmscan (option: -E 1e-4) to characterize its full domain composition and extracted the protein sequence of its GFP domain region accordingly. The protein sequences of all these GFP domains were scanned together by MEME (v4.11.1)^53^ (options: -protein -mod zoops -nmotifs 10 -evt 0.01 -maxsize 200000) to detect conserved motif composition shared among them. For each detected motif, we calculated the motif abundance (proportion of the test sequences with this motif) for all the GFP-encoding sequences within the corresponding evolutionary lineages (hydrozoan cnidarians, anthozoan cnidarians, copepods, and cephalochordates).

## Additional Information

**How to cite this article**: Yue, J.-X. *et al.* The evolution of genes encoding for green fluorescent proteins: insights from cephalochordates (amphioxus). *Sci. Rep.* **6**, 28350; doi: 10.1038/srep28350 (2016).

## Supplementary Material

Supplementary Information

## Figures and Tables

**Table 1:** GFP-encoding genes identified in cephalochordates.

Clade	Species	Gene name	Gene model ID	Chromophore region	Genomic coordinate (scaffold:start-end:strand)
GFPa	*B. floridae*	GFPa1	63256	HL GYG YY	Bf_V2_107:1004669–1007396:+
		GFPa2	63262	HL GYG YY	Bf_V2_107:912177–916945:+
GFPb	*B. floridae*	GFPb1	75522	HL GYA YY	Bf_V2_131:724134–735690:−
		GFPb2	75521	HL GYA FN	Bf_V2_131:710687–716998:−
		GFPb3	75519	QI GYG FH	Bf_V2_131:674613–680969:+
		GFPb4	75520	HI GYG FY	Bf_V2_131:693853–703767:+
	*B. belcheri*	GFPb1	233630F	HF GYG YD	scaffold58:854039–856682:+
		GFPb2	264830F	HF AYG YD	scaffold718:9844–13364:+
		GFPb3	240030F	HV GYG YH	scaffold6:4139317–4157041:+
GFPc	*B. floridae*	GFPc1	75523	NI GYG FH	Bf_V2_131:741722–750056:+
	*B. belcheri*	GFPc1	199320R	NL GYG FH	scaffold44:34684–36671:-
GFPd	*B. floridae*	GFPd1	86184	HL GYG HY	Bf_V2_226:1594162–1619868:−
		GFPd2	126982	HL GYG HY	Bf_V2_106:577911–582844:+
	*B. belcheri*	GFPd1	282900F	HL GYG FY	scaffold83:996645–1004389:+
		GFPd2	005130R	HL GYG FY	scaffold1:5804381–5807161:−
		GFPd3	005140R	HL GYG FY	scaffold1:5811232–5813910:−
		GFPd4	145360F	HL GYG FY	scaffold291:172581–179080:+
		GFPd5	233640F	HL GFG FY	scaffold58:860037–874989:+
GFPe	*B. floridae*	GFPe1	63257‡	NL GYG FY	Bf_V2_107:979539–982204:+
		GFPe2	63260	NL GYG FY	Bf_V2_107:950946–955643:+
		GFPe3	63258	NL GYG FY	Bf_V2_107:971176–973558:−
	*B. belcheri*	GFPe1	144690R	NL GYG FY	scaffold29:2454423–2458156:−
GFPf	*B. floridae*	GFPf1	63259	NL GYG YH	Bf_V2_107:961008–966261:−
	*B. belcheri*	GFPf1	144780R	NL GYG YH	scaffold29:2657919–2677300:−
	*B. belcheri*	GFPf2	212280R	NL GYG YH	scaffold5:926780–961355:−
	*A. lucayanum*	GFP1	asym20h*	NL GYG YH	
Un- classified	*B. belcheri*	GFPx1	276530F	HL GYG FY	scaffold8:2499615–2549717:+
	*A. lucayanum*	GFP2	asym20h**	HL GYG LY	

^‡^The internal sequence gap within the *B. floridae* gene model 63257 was filled based on our previous study^23^.

^*^Asym20h_comp74545_c2_seq1_m.28460.

^**^Asym20h_comp64813_c0_seq2_m.13123.

**Table 2:** Average molecular evolutionary rates for cephalochordate GFP-encoding genes within each species.

	D_JC_	D_Pois_	D_n_	D_s_	D_n_/D_s_
*A. lucayanum*	0.649	0.809	0.562	1.033	0.544
*B. belcheri*	0.506	0.640	0.433	0.819	0.532
*B. floridae*	0.451	0.619	0.392	0.697	0.567
*B. lanceolatum*	0.202	0.280	0.163	0.357	0.421

D_jc_: Jukes-Cantor nucleotide substitution rate; D_Pois_: Poisson amino acid substitution rate; D_n_: nonsynonymous substitution rate; D_s_: synonymous substitution rate.

**Table 3:** Average molecular evolutionary rates between *B. belcheri* and *B. floridae* co-orthologs within each GFP clade.

	D_JC_	D_Pois_	D_n_	D_s_	D_n_/D_s_
Clade b	0.301	0.424	0.249	0.507	0.493
Clade c	0.198	0.203	0.104	0.591	0.176
Clade d	0.312	0.387	0.230	0.649	0.354
Clade e	0.207	0.206	0.113	0.594	0.190
Clade f	0.277	0.279	0.191	0.656	0.290

D_jc_: Jukes-Cantor nucleotide substitution rate; D_Pois_: Poisson amino acid substitution rate; D_n_: nonsynonymous substitution rate; D_s_: synonymous substitution rate. Data for clade a is not available since no *B. belcheri* GFPa gene was identified.

**Table 4:** Top ten compositional motifs of the GFP domain.

Motif ID	Regular expression for the motif*	E-value	Width
Motif 1	D[YF]FK[QS][SA][FM]PEG[YF][SVT][WQ]ER	5.7 e-6277	15
Motif 2	[ML][ED]G[DST]VNGH[KE]FS[IV][ES]GEGEG[KDN][PA][YTF][EY]G[KT][QL]T[LM]K[LF]	1.2 e-9032	29
Motif 3	GVNFP[AP][ND]GPVMQKKTL[GK]WEPSTE[KR][ML]	2.7 e-6330	25
Motif 4	NYNSHNVYI[MT]ADKQKNGIK[VA]NFKIRHNIEDGSVQLADHYQQ	1.2 e-6048	41
Motif 5	PVLLPDNHYLSTQSALSKDPNEKRDHMVL	1.0 e-4182	29
Motif 6	DGNYKTRAEVKFEGDTLVNRIELKGIDFKEDGNILGHKLEY	2.0 e-6106	41
Motif 7	LP[FV][ASP][WF][DP][IT]L[SVT][TP][TA][FL]XYG	3.9 e-4433	15
Motif 8	L[LK]L[EK][GD]GGH[YL]RC[DQ]F[KR][TS]TYKAKK	1.1 e-4095	21
Motif 9	YH[FY]VDH[RK][IL][ED]I[TL]SH[DN][KE]DY[TN]KV[EK][LQ][YH]EHA[EV]A[RH]	4.9 e-4025	29
Motif 10	M[TN][FY]EDG[GA][VI]CT[AV][TS][NQ]DI[ST]L[EQ]G[DNG]C	1.7 e-3959	21

^*^Amino acids within the brackets are interchangeable.
