# Supplementary material for: The evolution of genes encoding for green fluorescent proteins: insights from cephalochordates (amphioxus)
Source: Sci Rep. 2016 Jun 17;6:28350. doi: 10.1038/srep28350 (PMC4911609; doi:10.1038/srep28350)
Supplement: Supplementary Information [file srep28350-s1.pdf]

The evolution of genes encoding for green fluorescent proteins: insights from cephalochordates (amphioxus)

Jia-Xing Yue, Nicholas D. Holland, Linda Z. Holland, Dimitri D. Deheyn

Supporting Information Online:

Supplementary file 1: The CDS nucleotide sequences of the 49 cephalochordate GFP-encoding genes used in this study (in FASTA format).

>Asymmetron_lucayanum|GFP2|asym20h_comp64813_c0_seq2_m.13123
GACTCTCAGCTTCCACAACTCGCATCAGACGCACAACTCAGTTCGATCATGGCAAT
CCCGACAAGCCACGACACGCACATCTTCGGCTCCATCAACGGCGTGGAGTTCGAC
ATGGTGGGCGGAGGTACCGGCAACCCGAAGGACGGGTCCGTGAACAGCACCGTG
AAGTCCACCAAAGGCGGCCTGCCCTGCTCCCCGCTCCTGATCGGACCGCACCTC
GGGTACGGCTTGTACCAGTACCTGCCCTTCCCGGACGGGCCGTCGCCTTTCCAGG
CCGACATGGGGTACGAAGTCCTCCGTACCATGAAGTTTGAGGACGGCGCTGTGCT
GAGCGCCAACTACCGCTACTCCTACCAGGGCGGCAAGATCAAGAGCGAGCATAAG
CTGGTGGGCAGCGGTTTTCCTGCCGACGGGCCGGTCCTGAAGAACAAGCTGGCC
TGCCTGGACCGCAGCGTGACCAAGCTGAAGTACGTGGACGACAGGAACCTGGTG
GACACGGTGGATTGGGCCTACAGCACTACCGACGGCAAGAAATACAACGCCGTG
GTGCAGACCAACTATTCCTGGGGAAACCCCAGCAACGCCGCCTTTAAGGACAAGA
TGCCGGCGTTCGTGTTCCGCCAGATCGACGTGAGCGGGTCTAAGACCGAGGTCC
ACCTGGTGGAGAAGCAGAAGGCGTTCTACGACTTGATC

>Asymmetron_lucayanum|GFP1|asym20h_comp74545_c2_seq1_m.28460
ATGTCTCTGCCAACCCAGCATGATTGCCACATCTTTGGCAGCATCAATGGCATCGA
CTTCGACATGGTGGGAGGAGGGAAGGGTGACCCTCACAAGGGCATACTGGAGAC
GACTGTGAGGTCCACCAAGGGTCCTCTGCCGTTCTCTCCACTCATCCTGGGCCCG
AATCTTGGGTACGGCTACCACCAGTACACGCCCTTTCCCAACGGGCAGTCCCCGT
ATCAGAACGCTGTCCACGACGGAGGCTACAAAAAGCACCGCACCTTCACGTTTGA
AGATGGTGGCATTCTGAGTATCACCTTCAACTACACCTACGAGGGAAATAAGATCT
ATGGAGAGTTCCAGTGTGTCGGCACTGGGTTCCCGGATGTGGGTCCTGTGATGAC
CAACGCGCTGGTGGGTGAACATCCCAACTGTGAGAAGCTGATGATTTCTGGAGCC
AACAGCATCGGGAGTGACAACAACTGGAGCTTCAGCACCAACCAGAAAGGCAAGT
ACTACAAGGCCAACGTGGTGACCAACTCCACCTTCGCCAAGAAGCTCCCCGCAGA
CCTTGAGGACATCATGCCTCTGTTCGTGTTCCGTAAGACGCAGGTCAAGTGCACCA
AGACCGAGATCACCCTGCATGAGTCGGAGAAATCCTTCTCGGATCTC

>Branchiostoma_belcheri|GFPd2|005130R
ATGCCGACAAGCCACGACCTGCACATCCATGGCTCCATCAACGGTTTGGAGTTTG
ATATGGTGGGAGGAGGTAGCGGCAACCCGAACGACGGGTCCGTGGAGACCCGTG
TGAAGTCCACCAAAGGTGGCCTGCAATTCTCCCCGCTCATCGTGGGCCCCCATCT
GGGGTACGGGTTCTACCAGTACCTGCCCTTCCCCAGCGGACCGTCGCCTTACCAG

GCCGCCATGAAGGACGGAGGGTACAAGATGCACCGTACCATGCAGTTTGAGGAC
GGCGCCGTTCTGACTGCCAACTATCGTTACACCTACGAGGGCGGCAAGATCAAAG
GAGATTTCCATCTGGTTGGCAGCGGTTTCCCCACCAACGGACCCGTGATGACCAA
CTCCCTGGCCTCTCAGGATAGGTCCGTGGCGAAGCTGACCAGTGTCGACGATCAC
ACCGTGGTCGAAAACATCGACTGGGCCTACCGCACCAGCGACGGCGGAAGCTAC
CGTGCCATGGTGCGGACCAACTGCACCTTCGGCAGGCCGGCCGCGGGCGTCAAG
GGGAACATGCCCATGTTCGTGTTCCGTCAGCTGGAGATCTCGGGCTCCAAGAGCG
AGATCAGCCTGCAGGAGAGGCAGAAGGTGTTCTCTGAGGTG

>Branchiostoma_belcheri|GFPd3|005140R
ATGTCTCTCCCGACAAGCCACGACCTGCACATCCATGGCTCCATCAACGGTTTGG
AGTTTGATATGGTGGGAGGAGGAAGCGGCAACCCGAAAGACGGGTCCGTGGAGA
CCCGTGTGAAGTCCACCAAAGGTGGCCTGCAATTCTCCCCGCTAATCGTGGGCCC
CCATCTGGGGTACGGGTTCTACCAGTACCTGCCCTTCCCCAGCGGACCGTCGCCT
TACCAGGCCGCCATGAAGGACGGAGGGTACAAGATGCACCGTACCATGCAGTTTG
AGGACGGCGCCGTTCTGACTGCCAACTATCGTTACACCTACGAGGGCGGCAAGAT
CAAAGGAGAGTTCCATCTGGTTGGCAGCGGTTTTCCCGCCAACGGACCCGTGATG
ACCAACTCCCTGGCCTCTCAGGATCGGTCCGTGGCGAAGCTGACGAGTGTCGAC
GATCACACCGTGGTCGAAAACATCGACTGGGCCTACCGCACCAGCGACGGCGGA
AGCTACCGTGCCAATGTGCGGACCAACTGCACCTTCGGCAGGCCGGCCGCTGGC
GTCAAGGGGAACATGCCCATGTTCGTGTTCCGTCAGCTGGAGATCTCGGGCTCCA
AGAGCGAGATCAGCCTGCAGGAGAGGCAAAAGGTGTTCTCCGAGGTG

>Branchiostoma_belcheri|GFPe1|144690R
ATGGCTCTGCCCACGAATCACGACCTGCACATCTTCGGCTCTGTGAACGGACTGG
AGTTCGACATGGTGGGAGGCGGCAGCGGCAACCCGAAGGCCGGAACCCTGGAGA
CGAGTGTGAAGTCCACCCGCGGTGCCCTGCCCTTCTCCCCCCTGCTTGTGGCGCC
CAACCTGGGTTACGGGTTTTACCAGTACCTGCCATTTCCGGACGGCCCTCACCTT
TCCAGACCGCCATCACGGACGGAGGGTACGAGGTGAACCGCGTTTTCAAGTTTGA
GGACGGCGGCGTTCTGAGTGCACACTTCCGCTACACATACGAGGGCGGCAAGAT
CAAAGGGGAATTCCAGTTGGTTGGTTCCGGTTTTCCCGCCAGTGGGCCCGTCATG
ACCAGCGGCCTGACCTCACTTGACCGGAGCGTGGCCAAACTGATGTGCTCCGACA
ACCGCACCCTTACAGGCCTCAACAACTGGAGCTTCTGCACCTCTGATGGGCAGCG
CCACCAGGCGGTGGTCCAGACGAACTACACCTTCGCCAAGCCCTTCCCAGCAGG
CACTGCGGAGAAGATGCCTGTGTTCATGGGACACCAGATCGAGGTCAACGCCTCC
AAGACCGAAATCGCCCTGACCGAGAAAGCCAAGGCCTTCTGTAACACTGTG

>Branchiostoma_belcheri|GFPf1|144780R
ATGATGTCTCTGCCAACCACTCACGATTGCCACATCTTCGGAACCATCAACGGACA
CGCCTTCGACATGGTGGGAGGCGGCAGCGGCAACCCCAAGGATGGATCGCTGCA
GACCACCGTGAGGTCTACCAAAGGTCCTCTGCCCTTCTCCGGCGTGATCCTGGGG
CCGAACCTGGGGTACGGCTACCACCAGTACCTCCCCTTCCCCAGCGGGATGTCTC
CCTACCAAAACGCCATCAAGAACGGAGGGTATGAGAAGCACCGCACGTTCCAGTT
CGAGGACGGCGGCGTGATGAGCATCAACTTCCGCTACAGCTACGAGGGCAACAA
GATCAAGGGAGAGTTCCACGTTGTGGGTTCCGGGTTCCCTGATGACGGCCCTGTG
ATGACCAACTCTCTGCAGGCTCACGACAATAACGTCGAGCGTCTGCAAGTTCTTGG

CGACAGGACCATCGGCAGCGACAACGTCTGGACTTACACCGGGAAGGGCAAAGG
CGGCACCTACAAGGCCAACGTGATGACCAACGCTACCTTCGCCCAAAACCTGCAG
CCAGGCCTGGAGGCCATCATGCCGCTCTTCGTCTTCCGCAAGGTTGACATCGAGG
CCTCGAAGACGGAGATCACCCTGGTGGAACGCGAGAAAGTTTTCTCTGATCTCTG
CCATATTGAGGCGGCGATAACTGGTTTCTATAGTGACAGAGAATCTGAAGCGTCG
GTGAAGGTTAGACATCCAGTGTTTTCTACCATCTACAAGCTCGCAACTCACCAGAA
ACCCAACACAAGATCAATACAAACATCACCAGCCAACTCGGCCCCGATCGGCAGG
AGACCGGCTGCAGACATGGGAGCCAATCAGAGCAGAGCGATGTACGCCGCGTCG
TTCGCAGTGGCGGGGGCGGGGCTTCTCTACATTTTCAAAGATACACTGACAGGAG
GACTCAACGCTTCCTCAAGAAAGAATGGAAAAGCTGAACCGGTGGCGCCACCAGT
TGAGAAGGCAAAGGCGCCAAAACCAGTTGCCGAGGCAGTGGTGGAGAAACCGGC
GGCACCGGAGGTTGTAGCGGAGGTTTTGGAGGTTGCGAAGCCAGCTGCAGAGGT
TGTGCCAGTGGCAGAGGCCGTCGTTTTAGAGGAGGTTGCTGCAGTAGCCGCCGA
CGTTGCAGCCGCTGCCCCGGAAGCAGTTCCAGAGGTCGCCGCCGCCCCGGAAGC
AGTTCCCGAGGTCATCGCCGAGACTGCAGTTCCGGTAGCGGAAGTGGTGGAGGC
AGCACCGGAAGTAGCAGTGGAAGCTCCCGCACCAGTTGTAGAGGCCGCCGTGGA
AGAGGTTGCAGTGGTTGTTGAGGAGGTAGCTGCTGCCCCGGAAGCAGTTCCAGA
GGTCGTGGCAGAGGAAGTGGCTGCTGCCCCGGAAGCAGTTCAAGAGGTTACAGT
GGAAGTGGCTGCTGCTCCGGAAGCAGTTCAAGAGGTTACAGTGGAAGTGGCTGCT
GCCCTGGAAGCAGTTGAAGAGGTTGTAACAGAGGCTGCCCCGGAAGCAGTTCCA
GAGATCAAGGAGGAAGTGGCTGCTGCCCCGGAAGCAGTTCAAGAGGTCGTTACA
GAGGCTGCTCCGGAAGCAGTTACAGAGGTTACAGTGGAAGTTGCTGCTGCCCCG
GAAGCAGTTACAGAGGTCGTGACAGAAGCTGCTCCGGAAGCAGTTACAGAGGTCA
CAGATGAAGTAGCTGCTGCTCCGGAAGTAGTTAAAGAGGTCGTTGCAGAAGTTGC
AGAGGCCGTGGAGATCGCTGTAGCTGAACCCGAACCTACAGCGGTGGTGGAAGA
AACCCCTGCAGCAGCCGAAGACACGCCAGCTGCAGTGGCTGCAGAAGAACCAAT
CCCTGCAGAGGAACCAGTCGCTGCAGTCGAAGACACGCCAGTCGTCGCAGCAAC
AGGGCCATTTACTGTGACAACAGAATTCGCTGAGGTTCCTGAACCACCCCTAGAC
GCAACGCACTGCCTGGTTACAGCCGAAAGCAGCACACCAGTCGTCGCAGAAACAG
AGGCAGTCGCTGCAGAAATTCCTGCAGCTGAAGAATCACCTGCAGAAGCAACGCA
GCCTGCAGCAGCTGCAGTCGATGTTCCCGCCGAAAACGGCGTGCCCGAAGAAGC
ACCCCCTACCGACGAAATCCTCCCCGACAAAAAGGCAGCCCTAGCGGATGCAGTA
CCGTCGAAGGCCACAGAGGGCGCTGCCGCCTCA

>Branchiostoma_belcheri|GFPd4|145360F
ATGTCTTTCCCGACAAGCCACGACCTGCACATCCATGGCTCCATCAACGGTTTGGA
GTTTGATATGGTGGGAGGCGGTAGCGGCAACCCTAAAGACGGGTCAGTTGAGACC
CGCGTGACGTCCACCAAAGGTGACCTGCCGGCCTTCTCGCCCCACATCGTTGCGC
CCCATCTGGGGTACGGGTTCTACCAGTACCTGCCCTTCCCCGGCGGACCGTCACC
TTACCAGGCCGCCATCAAGGACGGAGGCAAGTACAACGAACATCGTACCATACAG
TTTGAGGACGGCGCCGTTCTGACTGCCAACTATCGTTACACCTACAAGGGCGACA
AGATCAAGGGAGACTTCCACCTGGTTGGCAGCGGTTTTCCCGCCAGTGGACCGGT
GATGACCAACTCCATGGCCTCGCTGGACAGGACCGTGGCGAAGATCACGTGTGTC
GACGAGCACTACACCAGCAGCGGCGGAAGCTACCGTGCCATGGTGTTACATCATG
TAATCCAACACCACAGGGTGTTTGCTACAGCACCAGTTACCATAGTGACAGCCATC
TGGTTCAAGTCATTCACAATTACCACCAGCAGCGGCGGAAGCTACCGTGCCATGG

TGCAGACCAACTGTACGTTCCAGAAGCCGGCCGCGGGCGTCCAGGGGAACATGC
CCATGTTCGTGTTCCGTCAGCTGGAGGTCAAAGCCACCAAGACCGAGATCAGCCT
GCAGGAGAAGCAAAAGGCGTTCACAGAGGTGCAG

>Branchiostoma_belcheri|GFPc1|199320R
ATGCAGATAAAGATGACGATCATCCATGTATTTCTGCAGCCTGTCCCAGCGACCCA
CGAGCTGCACGTCTCCGGCTCCATCAACGGCCGGGAGTTTGACCTGGCCGGGCG
GGGCACAGGCAACGCAAAGGATGGTTCAGAGGAGATCCAGGTGAAGTCCACCAA
GGGTGCTCTGGGTTTCTCCCCGGTCCTCCTGGTGCCGAACCTCGGGTACGGCTTC
CACCAGTACCTGCCCTATCCGGACGGGATGTCGCCTTTCCAGGCCGCCGCGGAC
GACGGCTCGGGGTACGTAGTCCATCGTACATTGCAGTTTGAGGACGGTGGATCTG
TGACGGGGATCTACCGATATTCCTACGACGGAAGCCACATCAAAGGAGAGTTTAA
CGTGACCGGGAGCGGTTTTCCCGCTGACGGCCCTGTGATGACCAACTCGCTGAC
GGCAGTGGACCCCAGCGTGGCCACGGTGTTCTGTCCCAACGACACCACTGTTGTT
TCCACCATTGACTGGAGTTGCACCACTACCAGTGGCAAGCGCTACCACGGCACTG
TGCGGACCAACTACACCTTCGCCAAGCCGATCGCCGCCAGCTTCCTGCAGCAGCA
GCCGATGTTCGTGTTCCGTAAGACGGAGCTGAAGGCCTCCGACACCGAGCTCAGT
CTCAAGGAGTCCCAGAAGGCGTTCCATGGCCTG

>Branchiostoma_belcheri|GFPf2|212280R
ATGCCAGAGGCACTTCCGCTGACACACACGGTTAAGGCGCTAAGCCTCGGGCTCA
ACGCTGTCCTGCTACTTGCCATCAACATCTGTATACGGGAAGCTCCATCCTCAACA
AAGGCTCCAGAAAAGGTTCAAGAAGCAGCAATCGATAAAGGAAATGACCTCAGCA
GGGTCACTCCAGGTCATCCCCTGACCATCTGTCATCAGTGGCGTGACCCAGGGGA
CGTGTCCCGTCGGGCGGTGTCTCCGCCTCACTTACCACCGTACACCCCTGCCATC
ACCATTAACCCGCAATCCTCGGGAAAGGTGCATGTCCAGTTTCCAGTTCTCACGTT
TTGTTCCCATCTCAAACCACCTGAAGGCATGGCTGAATATAGGTATGAGATCAGGT
ATGAATGGGTGATTACATCTCTGCCTACCACTCACGATTGCCACATCTTCGGCACC
ATCAACGGACATGCCTTCGACATGGTGGGAGGCGGCAGCGGAAACCCGAAGGAT
GGATCGTTGCAGACCACCGTGCGATCCACCAAAGGTCCTCTGCCCTTCTCCGGCG
TTATCCTGGGGCCTAACCTGGGGTACGGCTACCACCAGTACCTACCCTTCCCCAG
CGGGAAGTCTCCCTACCAAAACGCCATCAAGAACGGAGGGTACGAGAAGCACCGT
ACGTTCCATTTCGAGGACGGCGGAGTGATGAGCATCAACTTCCGCTACACCTACG
AGGGCAACAAGATCAAGGGAGAGTTCCACGTTGTCGGCTCCGGGTTCCCTGACGA
TGGCCCTGTGATGACCAACTCTGTCCAGGCTCATGATAACAACGTGGAGCGGCTG
CAGGTTCTCGGCGACAGGACCATCGGCAGCGACAACGTCTGGACTTTCACCGGG
AAGGGGAAAGGCGACACCTACAATGCCAACGTGATGACCAACGCGACCTTCGCCC
AGAACCTGCAGCCCGGGCTGGAGTCCATCATGCCGCTCTTCGTCTTCCGGAAGGT
TGACATTCAGGCCTCCAAGACTGAGATCACCCTGGTGGAGCGGGAGAAGGTCTTC
TCTGATCTATGCCAGCATCTTCTCCGCGGTGCCTGCCGGGAAGGGCTTGGCGAAG
GTGTAGTTCGTCTGGACCACCGCCTGGTGGCGCTGCCCATCAGCGGTGCTGACAA
GTTAGGAGAAATCGGAAAAGAGTTCATCATCACCATCAGCATCATCGCCATCATCA
CCATCAGTAGCAGCATCAGCATCATCAGTGACAGTGTCTTCAGCATCATCGTCTTC
AGCACCAGGGCTAGTCATTTGTGCAGACCAGTTGGACAGCCTTATGTGAATGCCC
CTGTA

>Branchiostoma_belcheri|GFPb1|233630F
ATGGAGTGCCCCACGACTAGGAAGCCAGCCCTCTTCTCTCCCTACGTCCTCGCCC
CTCACTTCGGCTATGGCTACGACCAGTACCTGCCTTTTCCCGACCAAGGGGTGTC
GCCTTTCCAGGCCACCATGCAAGGCAGCTCGGGGTACCAAGTTATCCGCACGGTG
CATTTTGAAGACGGTGCCATCCTGAGTGCTCACTTCCGCTACACCTACGAGGGAA
GCCACATCAAAGGGGAGTTTCAGGTGATCGGGGGCGGTTTCCCTGCTGATGGCCC
GGTGATGACCAAATCACTGACGGCTATGGACTGGAGCGTGGCCAAACTGGTGTCC
CCTAACGACAACACCGTCCAGAGCACCATTGACATGGCATACACCACTACCAGCG
GTAAGCGCTACCAGAGCACTGTGCGCAACATCCACACCTTCGCCAAGCCGATGAC
CGCCAGCATCCTGCAGCAGAAGCCGGTGTTCGTGTTCCGTAAGACGGACCTCAAG
GCCAACAAGACCGAGGTCACCTTCAAGGAGTGGCAGAAGGCATTCACCGACGTGA
TG

>Branchiostoma_belcheri|GFPd5|233640F
ATGGGCAGCACAGGTGGCCCACAGCCTTACATGTCTTCCATAGTGCGTAGTGCAC
TTACATTTCAGTCTATCCCGACAAGCCACGACCTGCACATCCATGGCTCCATCAAC
GGTTTGGAGTTTGATATGGTGGGAGGCGGTAGCGGCAACCCTAAAGACGGGTCA
GTTGAGAGCCGCGTGACGTCCACCAAAGGTGACCTGCCGGCTTTCTCGCCCCACA
TCGTGGCGCCCCATCTGGGGTTCGGGTTCTACCAGTACCTGCCCTTCCCCGGCGG
GCCGTCACCTTACCAGGCCGCCATCGATGTCGGAGGCAAGTACAACGAACATCGT
ACCATACAGTTTGAGGACGGCGCCGTTCTGACTGCCAACTATCGTTACACCTACAA
GGGCGACAAGATCAAGGGAGACTTCCACCTGGTTGGCAGCGGTTTTCCCGCCAGT
GGACCCGTGATGACCAACTCCCTGGCCTCGCTGGACAGGACCGTGGCGAAGATC
ACGTGTGTCGACGAGCACAGTCTGGTCGACAACATTGACTGGACCTACCGCACCA
GCAGTGGCGGAAGCTACCGTGCCATGGTGCAGACCAACTGTACGTTCCAGAAGCC
GGTCAGGGGCGTCCAGGGGAACATGCCCATGTTCGTGTTCCGTCAGCTGGAGGT
CAAAGCCACCAAGACCGAGATCAGCCTGCAGGAGAAGCAAAAGGCGTTCACAGAG
GTGCAGACCCTGGGCCCTAAAATGAAGAAGCTGAATCCATTTAAAAAATGGCAGGA
GAAGGAGAAGGAGAAGGAGAAGGAGAAGGAGAAGGAGAAGGAGAAGGAGAAGG
AGAAGGAGAAGGAGAAGGAGAAGAAACACCAGAAAAAGCAATATGGTTTCACCAC
ACCACTTCGTGGTGTGGTGAACCCA

>Branchiostoma_belcheri|GFPb3|240030F
ATGTTAACCTGGGAGGTTCGCGCGGATGTTAACCTGGGCGCTACCCACGAGATTC
ACGTTTTTGGCTCCCTCAATGGTATTGAGTTTGACATGGTGGGCCGTGGCAGCGG
CAACCCAAAAGATGGTTCCCAGGAGCTACAAGTGAAGTCCACCAAGGGTTCCCTC
CCGTTCTCTGCCCAAATCCTCATTCCCCACTTCGGGTACGGCTTCTACCAGTACCT
GCCCTACCCTGACGGGATGTCGCCTTTCCAGGCCGCCATGCACGACGGCAAGGG
GTACAAGGTTAGCCGCACAATGATGTTTCAAGACGGTGCCATTATGGATGCCCAGT
ACCGCTACACCTACGAGGGAAGCCACATCAAAGGAGAGATGCAGGTGGTCGGGA
CCGGATTCCCAGCTGATGGCCCTGTGATGACCAAGTCGCTCAAGGCTGCTGACTG
GAATGTGACCAAGATGGTGTACCCTGACAACAAGACCATCCGGGCCACCTATGAC
TGGACATACACCACTACCAGTGGGAAACGCTACCAGAGCTCGGTGCGGACCAACC
ACACCTTCGCCAGCCCGATAGCCGCCAACATCCTGCAGCAGGAGCCGATGTTCGT
GTTCCGTAAGACGGAGCTCAAGGGAAATGCACAGGACACCGAATTCACCTTCAGG
GAGTTGCAGAAGGCCTTTAGCGATTTGATAGATCTGTACACCAAGAAGCTTGAAAG

TGACAACCCTTTCGATCTGCTTACCATCTACGCTACCCACGAGATTCACATTTATGG
CTCCTTCAATGGTGTTGAGTTTGACATGGTGGGCCGTGGCACCGGCAACCCAAAA
GATGGTTCTGAGGAGCTACACATGAAGTCCACCAAGGGTCCCCTTCCGTTCTCTG
CCCAAATCCTCATCCCTCACGTCGGGTACGGTTACCACCAGTACCTGCCTTATCCT
GACGGGATGTCGCCTTTCCAGGCCGCCATGCAGGACGGTTCGGGGTACCAAGTC
ACCCGCATAATGCAGTTTGAAGACGGTGCCAATGTAACTGGCCACTACCGCTACA
CCTACGAGGGAAGCCATATCAAAGGAGATATGCAGGTGATCGGGACTGGATTCCC
TGCTGATGGCCCAGTGATGACCAAGTCGCTCACGGCTGTGGACTGGTGCGTGGC
CAAGAATGTGCACCCTGACAACAAGACCATCAAGGCCACCTTTGACTGGACACTCA
CCACTACCAGTGGGAAACGCTACCAGGCCTCGGCGAGGACCAACTACACCTTCGC
CAAGCCGATGGCCGCCAATTTCCTGCAGCAGGAGCCGATGTTCGTGTTTCGTAAG
ACGGCGATCCAGGGTAATGCAGATGACACCGAATTCGCCTTCAAGGAGGAACAGA
AGGCCTTCGCCGATTTGATG

>Branchiostoma_belcheri|GFPb2|264830F
ATGTCGAGTCGTCTTCTGTGCTTTTTTGGACGAAAGAAGAAACTTGCGTCCCACGA
GCTTCACATTTTTGGCTCCATCAATGGCGTTGAGTTTGACATGGTGGGCCATGGCA
CCGGCAACCCCAAAGATGGGTCCCAGGAGCTACAAGTGAAGTCCACCAAGGGTC
CCCTCCCGTTCTCTCCCTACGTCCTCGCCCCTCACTTCGCGTATGGCTACGACCA
GTACCTGCCTTTTCCCGACCAAGGGGTGTCGCCTTTCCAGGCCACCATGCAAGGC
AGCTCGGGG

>Branchiostoma_belcheri|GFPx1|276530F
ATGGCGATGACTCGATGCCTTACGCTGGTGGTAGTGACTGCATTTATGTTCGCGAG
AGGAGGGTATGCAAATATCCTCCACTGTCTGTACTCATCGCAACAGACATTCCACT
GCATGAACGGTGACTGTGTGCCGCAGGAGTACGTGTGTGATGGCTGGTACGACTG
CGGCGACTGGAGCGACGAGCTCAGCGGCTGCCGCACCAGAGCTACCCTGGATAT
AACTACAAGACAGTTATCTCCGTCAGCTTTACCTCACAAATCTACAAAGATGGCACA
ACACGCAACCCAGGGTGACAACATAAAGTATTCAACGGGTTTAGGAATCGGAGGA
AATGCTGACACACAGAAAGAAGACGAGAAAGCAGGGGCGCGAACCAAGCTCCCC
GCGGCTATCGGAGGGACCGTCGGCGCTGTGGCGCTACTTGGACTGCTACCTCTC
CCAGCTACGCACGAGTTACACATCTATGGCTCTATCAACGGCCATGAGTTTGACAT
GGTAGGCGGTGGTGACGGCAACCCGAAGGACGGACACATGCAGACGAATGTGAA
GTCCACAAAGGGCCCCCTCGCCTTCTCCCCCTACATCGTGACCCCCCATCTCGGG
TACGGGTTCTACCAGTACCTCCCCTTCCCTAACGGGGAGATGTCACCGTTCCAGG
CCGCTATGCAGGACGGCGGGTACGAAATCCACCGCACGTTCCGGTTTGAGGACG
GCGCCATGCTGAGCGCCAACTACAAGTACTTCTTCGACGGCACACACATCAAGGG
AGAGTTTTGTCTGACTGGAAGCGGTTTTCCCGCTAACGGCCCCGTGATGACCAGC
GCCATCACCGGTGTGGACGACACCGTGTCCCGCATGGTCCCGCTCGACAACAACA
TGCTCTTCGACGCCTTCAGCTGGGGCTACCGAACCAGCAGTGGAAAGACGTACGA
GGCCATGGTCAACACCAACTACCAGTTCGGCAAGCCGATCCCGGCAGGCATCAAG
AGCAAGCTGCCGATGTTCGTGTTCCGTAAACTGGACATCAAGGTCTCCAAGACCG
AGGTCACCTTGGTGGAGTGGCAGAAAGCTTTCTCCGAGCCACACCTGACGAGCTC
GCTATCGGCAGAGATGAAGACCGAAGAGCAGGCGCCGCTGCTGCGGAGAGACTC
CGCGGCGTCTCCCGCCGGCGTGGCGGCCGTGCCGCGTCGCTCCTGGCGGCAGC
TGCTGATGAATGGGTCGATCCTGCTCGGGCGGGAGTTTTGCTACGCTCTGGAGGC

CGCCCTCGTGCTGCCCGTTCTCATGACCATCGGCATGCCCAGGGAGATGTACAGG
TTCTTTGGCCTGTGTTATGACTCAGTTCACAATATTTTGTCGAACATCCTGGGGATG
AACAAACTGTCTGCATACATTGTGTGGCTGATCCCGCCAGTCTTCGGGTTCATCTT
CGTGCCTCTGCTCGGCTCGGTGAGCGACCACTGCCGCTGCCGGTGGGGCCGCCG
CCGCCCCTTCATCCTGGCGCTCGGCCTCGCCATCATCCTCGGCTTCGCGCTCTTC
CTGAACGGCGACGCTCTCGTAACGCTGATCGCAGGGGACGTGAGTAACAGCCCC
AAGAGAGCCGACCGGAACACCATGCGCACTGCCACCCTGGCCGTCAGCATGTTC
GGCGCCATCTTGTTCGACTTCGCCGCAGACTTTATTGAGAGTCCGATCAAGGCTTA
CCTGCTGGACAACTGTGTGGAGAGGGACCGTAGGCGGGGTCTGGACATGCAGGG
AGCGCTCTCAGGTTTGGGCGGCTTTCTCGGCTACGCGACTGGTGCGATCGACTGG
ATAGACCTCGGCATTCCCCCGGGCTCAGAATACCACGTCATCTTCGGCATTTCGTG
CTCCGTGTTCACCCTCTGCCTGCTGCTGAACCTGGTCAGCATACGGGAGGTCCCG
CTGGACGACCTGACGGCAAACTCGCCGGACAATGGTGACAGAAAGCCGGGGAGA
GAGGAGCCAGTCCCGCCTCAGAACCCCGAAGCCGTGGTCGCCCTGGACCCCAAG
TATGGCGTCATGTACATGAGCGATGATCCTGATCTTCTCCAGGACGTGATGACCTG
TGATGACGGGCTACAGAAGGTGCTCGTCACGACCGTGTTCGGGGGAGGGGATCA
GTCTGACGACGGGTACGGCAGCATCGCGCAGTCGGAGGAAGGTAGCATCGACAC
CGCGGAGGACACAGACACTACCGAGCTGGCCCAGCGGCTGTCCATCACCGCCTA
CTTCAGATCGATCCTCCGGATGCCCAAGGAACTGGCCTGCTTGTGCGTGTCCCAC
TTCCTCGGCTGGGCGAGCTTCCTGTCCGTCATGTTGTTCTTCACCGACTTCATGGG
CCGGGGGGTGTACAAGGGGAACCCGAGCGCCCCGGTGGACAGTCCTGACAGGG
TCCTGTACGAACAGGGCGTGATGATCGGCTGCTGGGGGCTCACCATCAACGCAG
CCTCGTGTGCTCTCTACTCAAGTGTTTGTCAATCGATCCTCCGGATGCCCAAGGAA
CTGGCCTGCCTGTGCGTGTCCCACTTCCTCGGCTGGGCGAGCTTCCTGTCCGTCA
TGTTGTTCTTCACCGACTTCATGGGCCGGGGGGTGTACAAGGGGAACCCGAGCGC
CCCGGTGGACAGTCCTGACAGGGTCCTGTACGAACAGGGCGTGATGATCGGCTG
CTGGGGGCTCACCATCAACGCAGCCTCGTGTGCTCTCTACTCAATGCTCTTTGGG
CGGATTCTAAACCACCTGTCGTACCGGACCATGTACATCTTCGGGTACTTGGCGTT
CGGCGCAGGGATCGGCAGTATGGCCATCATCGCCCAGCTGACGGAGGTGCGCTG
GGAGATCATCTTCCTGTGTCCGGTCATGGGGATTATGTACGGCACCCTCAACAACA
TCCCCTACAAACTCATCTCACGGTACCACACCAGTCAAACGTACATCCGTACCGGC
GTGGACGGGTCGGAGCGCCGCGGCATGGGGATCGACTGCGCGCTGGTGTCCTC
CCAAAACCAGCTGTCTCAGATCGTCATCGGCGCCAGTATGGGCTCCATCGTGGCC
GCCGTGGGCAGCGTCATCTCCGTGACGGTGTGCTCCAGCGTGCTGGCCTTCACC
GCCTGCATCGCCGCAGCCTTACTGGTGCACTACGGCGTCGACAGGCGAGAGGAC
GGGGAGCCAGACGTCGAAGACTTACTGTCTATGTGCTTTGGACGGATCCTAAACC
ACCTGTCGTACCGGACCATGTACATCTTCGGGTACTTGGCGTTCGGCGCAGGGAT
CGGCAGTATGGCCATCATCGCCCAGCTGACGGAGGTGCGCTGGGAGATCATCTTC
CTGTGTCCGGTCATGGGGATTATGTACGGCACCCTCAACAACATCCCCTACAAACT
CATCTCACGGTACCACACCAGTCAAACGTACATTCGGTGCGGCGTGGACGGGTCG
GAGCGCCGCGGCATGGGGATCGACTGCGCGCTGGTGTCCTCCCAAAACCAGCTG
TCTCAGATCGTCATCGGCGCCAGTATGGGCTCCATCGTGGCCGCCGTGGGCAGC
GTCATCTCCGTGACGGTGTGCTCCAGCGTGCTGGCCTTCACCGCCTGCATCGCCG
CAGCCTTACTGGTGCACTACGACGTCGACAGGCGAGAGGACGGGGAGCCAGACG
TAGAAGACTTACTGTCTAGTATG

>Branchiostoma_belcheri|GFPd1|282900F
ATGTCTCTCCCGACAAGCCACGACTTGCACATCCATGGCTCCATCAACGGTTTGGA
GTTTGATATGGTGGGAGGCGGTAGCGGCAACCCGAAAGACGGGTCCGTGGAGAC
CCGTGTGAAGTCCACCAAAGGTGGCCTGGCCTTCTCCCCGCTCATCGTGGCCCA
CATCTGGGGTACGGGTTCTACCAGTACCTGCCCTTCCCCAGCGGACCGTCGCCTT
ACCAGGCCGCCATCAAGGACGGAGGGTACAACGAACATCGTACCATGCAGTTTGA
GGACGGCGCCGTTCTGACTGCCAATTATCGTTACACCTACGAGGGCGGCAAGATC
AAAGGAGACTTCCATCTGGTTGGCAGCGGTTTTCCCGCCAGCGGACCCGTGATGA
CCAACTCCCTGGCCTCCCTGGATAGGTCCGTGGCGAAGCTGACCAGTGTCGACGA
TCACACCGTGGTCGAGAACATTGACTGGGCCTACCGCACCAGCAGCGGCGACAC
CTACCGTGCCATGGTGCGGACCAACTGCACCTTCGGCAGGCCGGCCGCGGGCGT
CAAGGGGAACATGCCCATGTTCGTGTTCCGTCAGCTGGAGGTCGCGGGCTCCAA
GACCGAGATCAGCCTACAGGAGAGGCAAAAGGCGTTCTCAGAGGTGCAG

>Branchiostoma_floridae|GFPa1|63256
ATGCCTCTGCCCGCAACCCACGACATCCACCTTCACGGCTCCATCAACGGCCACG
AGTTCGACATGGTGGGGGGAGGAAAAGGCGACCCGAACGCCGGCTCGCTGGTGA
CCACAGCGAAATCCACCAAGGGTGCCCTGAAGTTCTCTCCCTACTTGATGATCCCC
CACCTCGGGTACGGGTACTACCAGTACCTCCCCTACCCGGACGGACCCTCGCCTT
TCCAGACCTCCATGTTGGAAGGATCGGGGTATGCAGTCTACCGCGTGTTCGACTTT
GAAGACGGAGGCAAGCTGACTACCGAGTTTAAGTACTCCTACGAGGGTTCCCATA
TCAAGGCCGACATGAAGCTGATGGGAAGCGGTTTCCCTGACGACGGCCCAGTCAT
GACCAGCCAGATTGTCGACCAGGACGGCTGCGTGTCCAAGAAGACGTATCTTAAC
AACAACACCATCGTGGACAGCTTCGACTGGAGTTACAACCTGCAGAATGGGAAGC
GCTACAGGGCCCGAGTGTCGAGCCACTACATCTTCGACAAGCCCTTTTCAGCCGA
TCTCATGAAGAAGCAGCCGGTCTTCGTGTACCGCAAGTGCCACGTGAAGGCTTCC
AAGACCGAAGTCACCCTGGACGAGAGGGAGAAGGCGTTCTACGAGCTGGCT

>Branchiostoma_floridae|GFPa2|63262
ATGCCTCTGCCCGCAACCCATGAAATCCACCTTCACGGCTCTGTCAACGGCCACG
AGTTCGACTTGGTGGGCAGTGGAAAAGGTGACCCGAAAGCCGGCTCGCTGGTGA
CCGAAGTGAAATCCACCATGGGTCCCCTGAAGTTCTCTCCTCACTTGATGATCCCC
CACCTCGGGTACGGGTACTACCAGTACCTCCCCTACCCGGACGGACCCTCGCCTT
TCCAGACCGCCATGCTCGATGGATCGGGGTATAAAGTCCACCGTGTGTTCAACTTT
GAGGACGGTGGCGTGTTGTCCATCGACTACAATTATGCCTACGAGGGGACTCACA
TCAAGTCCGACTTTAAGCTGATGGGAAGCGGTTTCCCTGACGACGGCCCAGTCAT
GACCAGCCAAATTGTCGACCAGGACGGCTGCGTGTCCAAGAAGACGTATCTTAAC
GACAACACCATCGTGGACAGCTTCGACTGGTCTTACAATCTGCAGAATGGGAAGC
GCTACAGGGCTCGAGTGACGAGCAACTACATCTTCGGGAAGCCCCTCGCGGCCG
ATGTAATGAAGAAGCAGCCGGTCTTCGTGTACCGCAAGTGCTACGTGAAGTCTAC
CCAGACCGAGATCACCCTGGACGAGAGGGAGAAGGCGTTCTACGAAGTGGTT

>Branchiostoma_floridae|GFPb1|75522
ATGCCTCTTCCAAAGACCCACGAGTTGCACATCTTTGGTTCCTTCAATGGTGTTGA
GTTTGACATGGTTGGTCGCGGCATCGGCAACCCTAATGAGGGGTCTGAGGAACTG
AACGCGAAGTTCACCAAGGGTCCCCTGAAGTTCTCCCCCTACATCCTGGTCCCCC

ACCTCGGGTACGCTTACTACCAGTACCTGCCCTTCCCTGACGGGATGTCGCCTTTC
CAGGCCGCCATGCACGACGGCTCGGGCTATCAAGTGCATCGCACGATTCAGTATG
AAGACGGTGCCTCCGTGACTGCCCACTACCGCTACACCTACGAGGGAAGCCACAT
CAAAGGGGAGTTTCAGGTGATCGGGACCGGATTTCCTCCTGACGGTCCTGTGATG
ACCAACAAGCTCACCGCTATGGACTGGAGCGTGACCAAGATGCTGTACCCGAACG
ACAAGACCATCCTCAGCACTGTCGACTGTAGCTACACCACCACCGAGGGCAAACG
CTACCAGAGCAAAATGCGTGAAAACAACACCTTCGCCAAGCCGATGGCGGCCGAC
ATCCTGCAGAAGCAGCCGATGTTCGTGTTCCGTAAGACGGAGCTCCAGCACTCCA
AGACCGAGCTCACCTTCAAGGAGTGGCAGAAAGCCTTCACCGATGTGATG

>Branchiostoma_floridae|GFPb2|75521
ATGCCTCTTCCAAAGACCCACGAGTTGCACATCTTTGGTTCCTTCAATGGTGTAGA
GTTTGACATGGTTGGTGAAGGCACCGGTAACCCTAATGAGGGGTCTGAGGAGCTA
AAACTGAAGTCCACCAATGGTCCCCTGAAGTTCTCCCCCTACATCCTGGTCCCTCA
CCTCGGGTACGCTTTCAACCAGTACCTGCCCTTCCCTGACGGGATGTCGCCTTTC
CAGGCCGCCATGCAGGACGAATCGGGGTACCAAGTGCATCGCACGTTGCAGTAT
GAAGACGGTGCCTTCGTGACTGCTAACTTACGCTACACCTACGAGGGAAGCCACA
TCAAAGGGGAGTTTCAGGTGATCGGGACCGGTTTTCCTCCTGACGGTCCTGTGAT
GACCAACAAGCTCACCGCTATGGACTGGAGCGTGGTCAAGTTTGTGTACCCCAAC
GACAAGACCATCCTCAGCACTTTCGACAAGACCTACACCACCACCGAGGGCAAAC
GCTACCAGTGCACATTTCGTGAAAACAGCACCTTCGCCAAGCCGATGGCGGCCGA
CATCCTGCAGAAGCAGCCGATGTTCATCTTCCATAAGACGGAGCTCCAGCACTCC
AACAATGCCGAGCTCACCTTCAAGGAGAAGCAGACAGCTTTCTCCGATATGAAG

>Branchiostoma_floridae|GFPb3|75519
ATGCCTCTTCCAACAACCCACGAGTTGCATATCTTTGGTACCTTCAATGGTGTCGA
GTATGACATGGTGGGCCGTGGCAAGGGTAACCCTAATGATGGATACGAAGAGCTA
AACCTGAAGTCCACCAAGGGTCCCCTCAAGTTCTCCCCATGGATCCTGGTCCCTCA
GATTGGGTACGGCTTCCACCAGTACCTGCCCTACCCTGACGGGATGTCGCCTTTC
CAGGCCGCCATGCACGATGGCTCCGGCTATCAAGTGCATCGCACGCTGGACTTTG
AAGACGGTGCCACCCTGACTGCCGACTTCCGCTACACCTACGAGGGGAGCCACAT
CAAAGGAGAGTTTAAGGTGATCGGGACCGGATTCCCTGCTGACGGTCCTGTGATG
ACCAACAAGCTCACTGCTGCGGACTGGTGTGTGAACAAGATGCTGTACCCGGACG
ACAAGACCATCAACAGCACCTTCGACTGGAGCTACACCACTTCCGAGGGCAAACG
CTACCAGAGCACAGTGAGGGAAAACTACACCTTCGCCAAGCCAATGGCCGCCAAC
ATCCTGCAGAAGCAGCCGATGTTCGTGTTCCGTAAGACGGAGCTCCAGCACTCCA
AGACCGAGCTCACCTTCAAGGAGTGGCAGAAAGCCTTCACCGATGTGATG

>Branchiostoma_floridae|GFPb4|75520
ATGCCTCTTCCAACTACCCACGAGTTGCACATCTTTGGTTCCTTCAATGGTGTGGA
GTTTGACTTGGTGGGCCGTGGCGAAGGTAATCCAAAGGATGGGTCTCAGAACCTA
CACCTGAAGTCCACCAAGGGTCCCCTACAGTTCTCCCCGTGGATGCTGATCCCTC
ACATCGGGTACGGCTTCTACCAGTACCTGCCTTACCCGGACGGCGAGATGTCGCC
TTACCAGGCCGCCATGTATGGTGGCTCAGGGTACCTAATGCATCGCACAATGCAA
TATGAAGACGGTGCCAAGATTAGTGGCCACTACAAATACACCTACGAGGGAAGCC
ACGTGAAAGGAGAATTTCAGCTCATCGGGACCGGATTCCCTACTGACGGTCCTGT

GATGACCAACCAGCTCACCGCTGCGGACTGGTGCGTGGACAAGTTGCTGTACCCC
AACGACAAGACCATTATCAGCAAGTTCGACTGGAGCTACACCACTACCGATGGCAA
ACGCTACCAAGCCAAGGTGCAGACAAACTTCGACTTCGCTAAGCCGATGGCGGCC
AACTACCTGCAGAAGCAGCCGATGTTCGTGTTCCGTAAGGTTGAGCTCGAGCACT
CCAAGACCGAGGTCAAATTCAAGCAGTGGCAGAAAGCATTCCACGATATCATG

>Branchiostoma_floridae|GFPc1|75523
ATGCCTCTTCCAACGACTCACGAGGTGCACGTCTATGGCTCTATCAACGGTGTTGA
GTTTGACTTGGTGGGTAGCGGCAAAGGCAACCCGAAGGATGGTTCTGAGGAGATC
CAAGTGAAGTCCACTAAGGGTCCCCTCGGGTTCTCCCCGTACATCGTGGTCCCCA
ACATCGGGTACGGCTTCCACCAGTACTTGCCCTTCCCCGACGGGATGTCGCCTTT
CCAGGCCGCCGCGGACGATGGCTCGGGGTACGTAGTCCATCGTAATATTCAGTTT
GAAGACGGCGCCTCGCTGACTGGCATCTACCGATATTCCTACGATGCAGGTCACA
TCAAAGGAGAGTTTCGTGTGGTTGGCAGTGGTTTCCCTGCTGACGGTCCTGTGAT
GACCAAATCGCTCACGGCTGTGGACTGGAGCGTGGCTACCATGCTGTTCCCGAAC
GACACCACCGTTGTCTCCACCATTGACTGGACTTGCCCCACTACCAGCGGCAAAC
GCTACCACGCCACGGTGAGGACCAACTACACCTTCGCCAAGCCGATAGCGGGCA
GCATTCTCCAGAAGCAGCCAATGTTCGTGTTTCGTAAGACGGAAGTCAAGGCCTCT
GACTCCGAGATCAACCTCAAGGAGTCGCAGAAGGCCTTTCATGACCTCGTG

>Branchiostoma_floridae|GFPd1|86184
ATGTCTGTCCCGACAAACCTCGACTTGCACATCTACGGCTCCATCAACGGTATGGA
GTTTGACATGGTGGGCGGTGGGAGTGGCAACCCAAATGACGGATCGCTGAGCGT
AAACGTGAAGTCTACCAAAGGTGCCCTGCGCGTTTCTCCTCTGCTGGTGGGCCCG
CATCTGGGGTACGGCCACTACCAGTACCTACCCTTCCCTGACGGTCCGTCGCCTT
TCCAGGCAGCCGTGAACAACGGCGGATATCAAATGCATCGCTCTTTCAACTTCGA
GGACGGTGCCGTGCTGACTGCCACCTACAACTACTCCTACAGCGGCGGCAAGATC
CAGGGAGAGTTTCATCTGGTGGGCAGCTGTTTCCCCGACGATAGTCCGGTGATGA
CCAACGCGCTGACCGGTTTGGACAGGAGCGTGGCCAAGCTGATGTGCGTGTCCG
ATGACAAGCTTGCCGAGTTCGTGGACTGGACCTACCGCACCAGCAGCGGCGGGC
GCTACCGTGCCACGGTGCAGACCAACTTCACCTTCGCAAAGCCCATCGCAGCTGG
CCTGAAGAACAACATGCCGATGTTCGTGTTCCGTCAGCTGGAAGTCACCGGCTCC
AAAACCGAGATCAGCCTTCAGGAGCAGCAAAAGGCGTTCTCCACCGTTCTG

>Branchiostoma_floridae|GFPd2|126982
ATGTCTGTCCCGACAAACCTCGACTTGCACATCTACGGCTCCATCAACGGTATGGA
GTTCGACATGGTGGGCGGTGGGAGTGGCAACCCAAAGGATGGATCGCTGGCCGT
AAACGTGAAGTCCACCAAAGGAGCTCTGTGCGTTTCCCCCCTGCTGGTGGGCCCG
CATCTGGGGTACGGCCACTACCAGTACCTACCCTTCCCTGACGGCGCGTCGCCTT
TCCAGGCAGCCGTGAACAACGGCGGGTATCAAATGCATCGCAGTTTCAACTTCGA
GGACGGGGCCGTGCTGACTGCCACCTACAACTACTCCTACAGCGGCGGCAAGAT
CCAGGGAGAGTTTCATCTGGTGGGCAGCGGTTTCCCCGACGATAGTCCGGTGATG
ACCAACGCGCTGACCGGTCTGGACAGGAGCGTGTCCAAGCTGATGTGCACGTCC
GATGACAAGCTCGTCGAGTCCGTGCACTGGAGCTACCGCACCAGCAGCGGCGGG
CGCTACCGTGCCACGGTGCAGACCAACTTCACCTTCGCAAAGCCCATCGAAGCTG

GCCTGAAGAACAACATGCCGATGTTCGTGTTCCGTCAGCTGGAAGTCACCGGCTC
CAAAACCGAGATCGGCCTTCAGGAGCAGCAAAAGGCGTTCTCCACCGTTCTG

>Branchiostoma_floridae|GFPe1|63257
ATGTCTCTCCCTACGACTCACGACCTTCACATCTTCGGCTCCGTCAATGGCGCGGA
GTTCGACCTGGTGGGAGGCGGCAGGGGCAACCCGAACGATGGAACGCTGGAGAC
CAGTGTGAAATCCACCCGGGGCGCCCTGCCCTGCTCCCCGCTGCTGATCGGACC
CAACCTGGGGTACGGCTTCTACCAGTACCTGCCCTTCCCTGGCGGCGCCTCACCC
TTCCAAACCGCCATCACGGACGGAGGGTACCAGGTTCACCGTGTGTTCAAGTTTG
AAGACGGCGGAGTGCTGAATTGCAACTTCCGCTACACCTACGAGGGCGGCAAGAT
CAAAGGGGAGTTCCAGCTGATCGGGTCAGGTTTCCCTGCCGGCGGGCCTGTGAT
GTCCGGCGGACTGACCACCCTGGACAGGAGCGTGGCCAAACTGCAGTGCTCGGA
CGACCGCACCATCACCGGCACTAACAACTGGAGCTTCTGCACCACCGATGGGAAG
CGCCACCAGGCGGATGTGCAGACGAACTACACCTTCGCCAAGCCGCTCCCGGCC
GGTCTGAAGGAGAAGATGCCGATCTTCCTGGGGCACCAGATCGAGGTCAAGGCG
TCCAAGACCGAGATCACCCTGAGCGAGAAAGTGAAGGCCTTCATCGACACTGTG

>Branchiostoma_floridae|GFPe2|63260
ATGTCTCTCCCAACGGCTCACGACCTTCACATCTTCGGCTCCGTCAATGGCGCGG
AGTTCGACCTGGTGGGAGGCGGAAAGGGCAACCCGAACGATGGAACGCTGGAGA
CCAGTGTGAAATCCACCCGGGGCGCCCTGCCCTGCTCCCCGCTGCTGATCGGAC
CCAACCTGGGGTACGGCTTCTACCAGTACCTGCCCTTCCCTGGCGGCGCCTCACC
CTTCCAAACCGCCATCACGGACGGAGGTTACCAGGTTCACCGTGTGTTCAAGTTTG
AAGACGGCGGAGTGCTGAATTGCAACTTCCGCTACACCTACGAGGGCGGCAAGAT
CAAAGGGGAGTTCCAGCTGATCGGGTCAGGTTTCCCTGCCGGCGGTCCTGTGATG
TCCGGCGGACTGACCACCCTGGACAGGAGCGTGGCCAAACTGCAGTGCTCGGAC
GACTGCACCATCACCGGCACTAACAACTGGAGCTTCTGCACCACCGATGGGAAGC
GCCATCAGGCGGATGTGCAGACGAACTACATCTTCGCCAAGCCGCTCCCGGCCG
GTCTGAAGGAGAAGATGCCGATCTTCCTGGGGCACCAGATCGAGGTCAAGGCGTC
CAAGACCGAGATCACCCTGTGCGAGAAAGTGAAGGCCTTCATCGACACTGTG

>Branchiostoma_floridae|GFPe3|63258
ATGTCTCTCCCTACGACTCACGACCTTCACATCTTCGGCTCCGTCAATGGCGCGGA
GTTCGACCTGGTGGGAGGCGGAAAGGGCAACCCGAACGATGGAACGCTGGAGAC
CAGTGTGAAATCCACCCGGGGCGCCCTGCCCTGCTCCCCGCTGTTGATCGGACC
CAACCTGGGGTACGGCTTCTACCAGTACCTGCCCTTCCCTGGCGGCGCCTCACCC
TTCCAAACCGCCATCACGGACGGAGGGTACCAGGTTCACCGTGTGTTCAAGTTTG
AAGACGGCGGAGTGCTGAGTTGCAACTTCCGCTACACCTACGAGGGCGGCAAGAT
CAAAGGGGAGTTCCAGCTGATCGGGTCAGGTTTCCCTGCCGGCGGGCCTGTGAT
GTCCGGCGGACTGACCACCCTGGACAGGAGCGTGGCCAAACTGCAGTGCTCGGA
CGACTGCACCATCACCGGCACCAACAACTGGAGCTTCTGCACCACCGATGGGAAG
CGCCACCAGGCGGATGTGCAGACGAACTACACCTTCGCCAAGCCGCTCCCGGCC
GGTCTCAAGGAGAAGATGCCGATCTTCCTGGGGCACCAGATCGAGGTCCAGGCG
TCCAAGACCGAGATCAACCTGAGCGAGAAAGTGAAGGCCTTCATCGACACTGTG

>Branchiostoma_floridae|GFPf1|63259
ATGTCTCTGCCTACGGCCCATGACTGCCACATGTTCGGCTCCATCAACGGCCACG
AGTTTGACCTGGTGGGCGGTGGAAACGGAACCCGAATGACGGGACACTGGAGA
CCAAGGTGCGCTCCACCAAGGGTGCCCTGCCCTTCTCCCCCGTGATCCTGGCCCC
TAACCTGGGGTACGGGTACCACCAGTACCTGCCCTTCCCGGCCGGGACCTCACC
GTACCAGCAGGCCATCACCAACGGAGTGTACCAAAAGCACCGCACCTTCAAGTTC
GAGGACGGCGGCGTCATGACCATCAACTTCCGCTACACCTACTCAGGGAACAAGA
TCAAGGGAGAGTTCCACGTGGTTGGATCCGGGTTCCCTGATGACGGCCCTGTGAT
GACCAACTCACTCCAGCAGCATGATCATAACGTGGAGAGGCTGATGGTGCTGGGA
GACAAGACCATCGGCAGCGACAACATGTGGACTTTCACTGAGAAAGGCGGCAAGG
ACAAGCGCTACAAGGCTGAAGTCATGACCAACGCCACCTTCGCGCAGAACCTCCA
GCCGGGTCTGAAGAACGTCATGCCGCTCTTCGTCTTCCGCAAGGTGGATATCGAC
TGCTCCAAGACGGAGGTCACCTTGATCGAGCGAGAGAAGGTCTTCCAGGATCTTC
TCCAG

>Branchiostoma_lanceolatum|FPX6|gi|169125832|gb|EU482407.1|
ATGTCTCTCCCAGCGACCCATGAGTTGCACATTTTTGGCAAAATCAATGGCCATGA
GTTTGACATGAGGGGTAAAGGCACCGGTAACCCAAATGACGGTTATGAGGACCTT
GACCTGAAGTCCACCAAGGATGACCTTCCATTCTCCCCCTGGATCCTGGTCCAAAA
CATCGGGTACGGCTTTAACCAGTACCTGCCCTACCCCGACGGAGCGATGTCGCCT
TTTCAGGCTGCCATGTACAATGGCTCCGGGTACCACGTCCATCGTGAAATGGAGTT
TGAAGACGGTGCCACGCTGACTGGCATCTACCGCTACACCTACGAGGGAAGCCAC
ATCAAAGGAGAGTTTCAGGTGGATGGGACCGGTTTCCCTGCTGACGGCCCTGTGA
TGACCGACTCGCTCACTGATCTGGACTGGGTCGTGACAAAGATGGTGTATCCCGA
CGACAAAACCGTCTTCAGCACCTCTGACCAGACCTACACCACTACAAGTGGCAAG
GGCTACCAGAGCACAGTGCGGACCAACAACATTTTTGCCGAGCCGATAGCGGCCG
ACATGATGCAGAGCCAGCCGGTATTCGTGTCCCGCAAG

>Branchiostoma_lanceolatum|FPX5|gi|169125830|gb|EU482406.1|
ATGTCTCTCCCAGCGACCCATGAGTTGCACATTTTTGGCAAAATCAATGGCCATGA
GTACGACATGAGGGGTAAAGGCACTGGTAACCCAAATGACGGTTATGAGGACCTT
GACCTGAAGTCCACCAAGGATGACCTTCCATTCTCCCCCTGGATCCTGGTCCAAAA
CATCGGGTACGGCTTTAACCAGTACTTGCCCTACCCCGACGGAGCGATGTCGCCT
TTCCAGGCTGCCATGTGCGATGGCTCCGGGTACGAGGTCCATCGTGAAATGGAGT
TTGAAGACGGTGCCACGGTGACTGGTATCTACCGCTACACCTACGAGGGAAGCCA
CATCAAAGGAGAGTTTCAGGTGGATGGGACCGGTTTCCCTGATGACGGCCCTGTG
ATGACCGACTCGCTCACTGATCTGGACTGGGTCGTGACCAAGATGGTGTACCCCG
ACGAGAAAACCGTCTTCAGCACCTCCGACCAGACCTACACCACTACAAGTGGCAA
GGGCTACAAGAGCACAGTGCGGACCAACAACATTTTTGCCAAGCCAATAGCGGCC
GACATGATGCAGAGCCAGCCGGTATTCGTGTCCCGCAAG

>Branchiostoma_lanceolatum|FPX4|gi|169125828|gb|EU482405.1|
ATGTCTCTCCCTAAGACCCACGAGTTGCATATTTTTGGCAAAATCAATGGCCATGA
GTACGACATGAGGGGTAAAGGCACAGGTAACCCAAATGACGGTTATGAGGACCTT
GACCTGAAGTCCAAGGATGACCTTCCATTCTCCCCCTGGATCCTGGTTCAAAACAT
CGGATACGGCTTTAACCAGTACCTGCCCTACCCCGACGGAGCGATGTCGCCTTTC

CAGGCTGCCATGTGCGATGGCTCCGGGTACGAGGTCCATCGTGAAATGGAGTTTG
AAGACGGTGCCACGCTGACTGGCATCTACCGCTACACCTACGAGGGAAGCCACAT
CAAAGGAGAGTTTCAGGTGGATGGACCGGTTTCCCTGATGACGGCCCTGTGATG
ACCGACTCGCTCACCGATCTGGACTGGGTCGTGACCAAGATGGTGTATCCCGACG
AGAAAACCGTCTTCAGCACCTCCGACCAGACCTACACCACTGCAAGTGGCAAGGG
TTACAAGAGTACAGTGCGGACCAACAACATTTTTGCCAAGCCAATGGCGGCCGAC
ATGATGCAGAACCAGCCGATATTCGTGTCCCGCAAG

>Branchiostoma_lanceolatum|FPX3|gi|169125826|gb|EU482404.1|
ATGTCTCTCCCAGCGACCCATGAGTTGCACATTTTTGACAAAATCAATGGCCATGA
GTTTGACATGAGGGGTAAAGGCACCGGTAACCCAAATGACGGTTATGAGGACCTT
GACCTGAAGTCCACCAAGGATGACCTTCCATTCTCCCCCTGGATCCTGGTCCAAAA
CATCGGGTACGGCTTTAACCAGTACCTGCCCTACCCCGACGGAGCGATGTCGCCT
TTTCAGGCTGCCATGTACAATGGCTCCGGGTACCACGTCCATCGTGAAATGGGGT
TTGAAGACGGTGCCACGGTGACTGGCATCTACCGCTACACCTACGAGGGAAGCCA
CATCAAAGGAGAGTTTCAGGTGGATGGACCGGATTCCCTGCTGACGGCCCTGTG
ATGACCAACTCGCTCACTGATCAGGACTGGTCCGTGACCAAGATGATGTACCTTGA
TAACAAAACCGTCACTAGCACCGCTGACCAGACCTACACCACTGCAAGTGGCAAG
CGCTACCAGGGCACAGTGCGGACCAACAACACCTTTGCCAAGCCGATAGCGGCC
AACATCCTGCAGAAGCAGCCGGTATTCGTGTCCCGCAAG

>Branchiostoma_lanceolatum|FPR9|gi|169125824|gb|EU482403.1|
ATGTCTCTCCCTAAGACCCACGATTTACACATCTCCGGCTCTGTCAATGGACATGA
GTTTGACTTGGAGGGCAGTGGCAAGGGCGATGCAAAAGAAGGTTATCAGGAGCTC
CACCTAAAGTCCAACAGGGGTGACCTGTCATTCTCCCCTTGGATCCTGGTCCCAAA
CATCGGCTATGGCTTCTACCAGTACCTGCCCTTCCCCGACGGAGCGATGTCGCCT
TACCAGGCCGCCATGCACGATGGCTCCGGATACGTGATGCATCGTGCAATGCGGT
TTGAGGATGGTGCTATGCTGCATTCAGACCACCGCTATACCTACAACGGAAACAAT
ATCAAAGGAGAGTTTCGGCTGACCGGGAGCGGTTTCCCTGCTGACGGCCCTGTGA
TGACCAACTCGCTGACCGCTGCGGACTGGTGTGTGGACAAGCTGCTGTACCCCAA
CGAGAACACCATTATCGGCAAATTCGACTGGACATACACCACTACAAGTGGCAAGC
GCTACCAAAGTGATGTGCAGACCAACGTCACCTTTGGCAAGCCAATATCGGCCGA
CATTCTGAAGAAGCAGCCGATGTTCGTGTTCCGTAAGGTGGAACTCAAGCACTCCA
AGACCGAGCTCAACTTCAAGCAGTGGCAGAAGGCATTCCAGGACATCGTG

>Branchiostoma_lanceolatum|FPR8|gi|169125822|gb|EU482402.1|
ATGTCTCTCCCAGCGACCCACGATTTACACATCTCCGGCTCTATCAATGGACATGA
GTTTGACTTGGAGGGCAGTGGCAAGGGCAATGCAAAAGAAGGTTATCAGGAGCTC
CACCTAAAATCCAACAAGGGTGACCTGTCATTCTCCCCCTGGATCCTGGTCCCAAA
CATCGGCTACGGCTTCTACCAGTACCTGCCCTTCCCCGACGGAGCGATGTCGCCT
TACCAGGCCGCCATGCACGATGGCTCCGGATACGTGATGCATCGTGCAATGCGGT
TTGAGGATGGTGCCATGCTGCATTCAGACCACCGCTATACCTACAACGGAAACCAT
ATCAAAGGAGAGTTTCGGCTGACCGGGAGCGGTTTCCCTGCTGACGGCCCTGTGA
TGACCAACTCGCTGACCGCTGCGGACTGGTGTGTGGATAAGCTGCTGTACCCCGA
CGAGAACACCATTATCGGCAAATTCGACTGGACATACACCACTACCAGTGGCAAG
CGCTACCAAAGTGATGTGCAGACCAACGTCACATTTGCCAAGCCAATATCGGCCG

ACATTCTGAAGAAGCAGCCGATGTTCGTGTTCCGTAAGGTGGAGCTCAAGCACTC
CAAGACCGAGCTCAACTTCAAGCAGTGGCAAAAGGCATTCCAGGACATCGTG

>Branchiostoma_lanceolatum|FPR7|gi|169125820|gb|EU482401.1|
ATGCCTCTTCCAGCGACCCACGATTTACACATCTCCGGCTCTATCAATGGACATGA
GTTTGACTTGGAGGGCAGTGGCAAGGGCAATGCAAAAGAAGGTTATCAGGAGCTC
CACCTAAAATCCAACAAGGGTGACCTGTCATTCTCCCCCTGGATCCTGGTCCCAAA
CATCGGCTACGGCTTCTACCAGTACCTGCCCTTCCCCGACGGAGCGATGTCGCCT
TACCAGGCCGCCATGCACGATGGCTCCGGATACGTGATGCATCGTGCAATGCGGT
TTGAGGATGGTGCCATGCTGCATTCAGACCACCGCTATACCTACAACGGAAACCAT
ATCAAAGGAGAGTTTCGGCTGACCGGGAGCGGTTTCCCTGCTGACGGCCCTGTGA
TGACCAACTCGCTGACCGCTGCGGACTGGTGTGTGGATAAGCTGCTGTACCCCGA
CGAGAACACCATTATCGGCAAATTCGACTGGACATACACCACTACCAGTGGCAAG
CGCTACCAAAGTGATGTGCAGACCAACGTCACATTTGCCAAGCCAATATCGGCCG
ACATTCTGAAGAAGCAGCCGATGTTCGTGTTCCGTAAGGTGGAGCTCAAGCACTC
CAAGACCGAGCTCAACTTCAAGCAGTGGCAAAAGGCATTCCAGGACATCGTG

>Branchiostoma_lanceolatum|FPR6|gi|169125818|gb|EU482400.1|
ATGTCTCTCCCTAAGACCCACGATTTACACATCTCCGGCTCTGTCAATGGACATGA
GTTTGACTTGGAGGGCAGTGGCAAGGGCAATGCAAAAGAAGGTTATCAGGAGCTC
CACCTAAAGTCCAACAGGGGTGACCTGTCATTCTCCCCTTGGATCCTGGTCCCAAA
CATCGGCTATGGCTTCTACCAGTACCTGCCCTTCCCCGACGGAGCGATGTCGCCT
TACCAGGCCGCCATGCACGATGGCTCCGGATACGTGATGCATCGTGCAATGCGGT
TTGAGGATGGTGCTATGCTGCATTCAGACCACCGCTATACCTACAACGGAAACAAT
ATCAAAGGAGAGTTTCGGCTGACCGGGAGCGGTTTCCCTGCTGACGGCCCTGTGA
TGACCAACTCGCTGACCGCTGCGGACTGGTGTGTGGACAAGCTGCTGTACCCCAA
CGAGAACACCATTATCGGCAAATTCGACTGGACATACACCACTACAAGTGGCAAGC
GCTACCAAAGTGATGTGCAGACCAACGTCACCTTTGGCAAGCCAATATCGGCCGA
CATTCTGAAGAAGCAGCCGATGTTCGTGTTCCGTAAGGTGGAACTCAAGCACTCCA
AGACCGAGCTCAACTTCAAGCAGTGGCAGAAGGCATTCCAGGACATCGTG

>Branchiostoma_lanceolatum|FPR5|gi|169125816|gb|EU482399.1|
ATGCCTCTCCCAGCAACCCACGATTTACACATCTCCGGCTCAATCAATGGACATGA
GTTTGACTTGGAAGGCAGTGGCAAGGGCAATGCAAAAGAAGGTTATCAGGAGCTC
CACCTAAAGTCCAACAAGGGTGACCTGTCATTCTCCCCCTGGATCCTGGTCCCAAA
CATCGGCTACGGCTTCTACCAGTACCTGCCCTTCCCCGACGGAGCGATGTCGCCT
TACCAGGCCGCCATGCACGATGGCTCCGGATACGTGATGCATCGTTCAATGCAGT
TTGAGGATGGTGCCATGCTGCATTCAGACCACCGCTACATCTATAAGGGAAACCAT
ATCAAAGGAGAGTTTCGGCTGACCGGAAGCGGTTTCCCTGCTGACGGCCCTGTGA
TGACCAACTCGCTGACCGCTGCGGACTGGTGCGTCGACAAGCTGCTGTACCCAAA
CGACAACACCATAATCGGCAAATTCGACTGGACCTACACCACTACCAGTGGCAAG
CGCTACCAAAGTGATGTGCAGACCAACGTCACATTTGGCAAGCCAATAGCGGCCG
ACATTTTGAAGAAGCAGCCAATGTTCGTGTTCCGCAAGGTGGAACTCAAGCACACC
AAGACTGAGCTCAACTTCAAGCAGTGGCAGAAGGCATTCCAGGACATCGCC

>Branchiostoma_lanceolatum|FPR4|gi|169125814|gb|EU482398.1|
ATGTCTCTCCCAGCGACCCACGATTTACACATCTCCGGCTCTATCAATGGACATGA
GTTTGACTTGGAGGGCAGTGGCAAGGGCAATGCAAAAGAAGGTTATCAGGAGCTC
CACCTAAAGTCCAACAAGGGTGACCTGTCATTCTCCCCCTGGATCCTGGTCCCAAA
CATCGGCTACGGCTTCTACCAGTACCTGCCCTTCCCCGACGGAGCGATGTCGCCT
TACCAGGCCGCCATGCACGATGGCTCCGGCTACGTGATGCATCGTACAATGCAAT
TTGAGGATGGTGCCATGCTGCATTCAGACCACCGCTATACCTATAAGGGAAACCAT
ATCAAAGGAGAGTTTAGGCTGACCGGGAGCGGTTTCCCTGCTGACGGCCCTGTGA
TGACCAACTCGCTGACCGCGGTGGACTGGTGTGTGGATAAGCTGCTGTACCCCAA
CGAGAACACCATAATCGGCAAATTCGACTGGACCTACACCACTACCAGTGGCAAG
CGCTACCAAAGTGATGTGCAGACCAACGTCACCTTTGCCAAGCCAATAGCGGCCG
ACATTCTGAAGAAGCAGCCGATGTTCGTGTTCCGCAAGGTGGAGCTCAAGCACTC
CAAGACCGAGCTCAACTTCAAGCAGTGGCAGAAGGCATTCCAGGACATCGTG

>Branchiostoma_lanceolatum|FPR3|gi|169125812|gb|EU482397.1|
ATGCCTCTTCCAGCGACCCACGATTTACACATCTCCGGCTCTATCAATGGACATGA
ATTTGACTTGGAGGGCAGTGGCAAAGGCAATGCAAAAGAAGGTTATCAGGAGCTA
CACCTAAAGTCCAACAGGGGTGACCTGTCATTCTCCCCCTGGATCCTGGTCCCAA
ACATCGGCTACGGCTACTACCAGTACCTGCCCTCCCCTGGC

>Branchiostoma_lanceolatum|FPR2|gi|169125810|gb|EU482396.1|
ATGTCTCTCCCAGCGACCCACGATTTACACATCTCCGGCTCAATCAATGGACATGA
GTTTGACTTGGAAGGCAGTGGCAAGGGCAATGCAAAAGAAGGTTATCAGGAGCTC
CACCTAAAGTCCAACAAGGGTGACCTGTCATTCTCCCCCTGGATCCTGGTCCCAAA
CATCGGCTACGGCTTCTACCAGTACCTGCCCTTCCCCGACGGAGCGATGTCGCCT
TACCAGGCCGCCATGCACGATGGCTCCGGATACGTGATGCATCGTTCAATGCAGT
TTGAGGATGGTGCCATGCTGCATTCAGACCACCGCTACATCTATAAGGGAAACCAT
ATCAAAGGAGAGTTTCGGCTGACCGGAAGCGGTTTCCCTGCTGACGGCCCTGTGA
TGACCAACTCGCTGACCGCTGCGGACTGGTGCGTCGACAAGCTGCTGTACCCAAA
CGACAACACCATAATCGGCAAATTCGACTGGACCTACACCACTACCAGTGGCAAG
CGCTACCAAAGTGATGTGCAGACCAACGTCACATTTGGCAAGCCAATAGCGGCCG
ACATTTTGAAGAAGCAGCCAATGTTCGTGTTCCGCAAGGTGGAACTCAAGCACACC
AAGACTGAGCTCAACTTCAAGCAGTGGCAGAAGGCATTCCAGGACATCGCC

>Branchiostoma_lanceolatum|FPR1|gi|169125808|gb|EU482395.1|
ATGCCTCTTCCAGCGACCCACGATTTACACATCTCCGGCTCTATCAATGGACATGA
GTTTGACTTGGAGGGCAGTGGCAAGGGCAATGCAAAAGAAGGTTATCAGGAGCTC
CACCTAAAGTCCAACAGGGGTGACCTGTCATTCTCCCCCTGGATCCTGGTCCCAA
ACATCGGCTACGGCTTCTACCAGTACCTGCCCTTCCCCGACGGAGCGATGTCGCC
TTACCAGGCCGCCATGCACGATGGCTCCGGCTACGTGATGCATCGTACAATGCAA
TTTGAGGATGGTGCCATGCTGCATTCAGACCACCGCTATACCTATAAGGGAAACCA
TATCAAAGGAGAGTTTAGGCTGACCGGAAGTGGTTTCCCTGCTGACGGCCCTGTG
ATGACCAACTCGCTGACCGCTGCGGACTGGTGCGTGGACAAGCTGCTGTATCCTA
CTGAGAACACCCTAATCGGCAAATTCGACTGGACTTACACCACTACCAGCGGCAA
GCGCTACCAAAGTGATGTGCAGACCAACGTCACCTTTGCCAAGCCAATGGCTGCC

GACATTCTGAAGAAGCAGCCGATGTTCGTGTTTCGCAAGGTCGAACTCAAGCACA
CCAAGACAGAGCTCAACTTCAAGCAGTGGCAGAAGGCATTCCAGGACATCGTG

>Branchiostoma_lanceolatum|FPO2|gi|169125806|gb|EU482394.1|
ATGCCTCTTCCAGCGACCCACGATTTACACATCTCCGGCTCAATCAATGGACATGA
GTTTGACTTGGAAGGGTCTGGTAAGGGCAATGAAAAAGAAGGTTATCAGGAGCTC
CACCTAAAGTCCAACAAGGGTGACCTGTCATTCTCCCCCTGGATCCTGGTCCCAAA
CACCGGCTACGGTTTCTACCAGTACCTGCCCTTCCCCGACGGAGCGATGTCGCCT
TACCAGGCCGCCATGCACGATGGCTCCGGATACGTGATGCATCGTTCAATGCAGT
TTGAGGATGGTGCCATGCTGCATTCAGACCACCGCTACATCTATAAGGGAAACCAT
ATCAAAGGAGAGTTTCGGCTGACCGGAAGCGGTTTCCCTGCTGACGGCCCTGTGA
TGACCAACTCGCTGACCGCTGCGGACTGGTGCGTCGACAAGCTGCTGTACCCAAA
CGACAACACCATAATCGGCAAATTCGACTGGACCTACACCACTACCAGTGGCAAG
CGCTACCAAAGTGATGTGCAGACCAACGTCACATTTGGCAAGCCAATAGCGGCCG
ACATTTTGAAGAAGCAGCCAATGTTCGTGTTCCGCATGATGGAACTCAAGCACACC
AAGACTGAGCTCAACTTCAAGCAGTGGCAGAAGGCATTCCAGGACATCGCC

>Branchiostoma_lanceolatum|FPO1|gi|169125804|gb|EU482393.1|
ATGCCTCTTCCAGCGACCCATGAGTTACACATCTTTGGCTCCTTCAATGGTGTGGA
GTTTGACATGGTTGGTCGCGGCACTGGCAACCCAAATGATGGGTCTGAGGATTTA
CACCTGAAGTCCACCAAGGGTGCCCTCCAGTTCTCCCCCTGGATCCTAATCCCTCA
CATCGGGTACGGCTTTCACCAGTACCTGCCCTTTCCCGACGGGATGTCGCCTTTC
CAGGCCGCCATGCAAGACGGCTCCGGATACCAAGTGCATCGCACTATGCAGTTTG
AAGACGGTGCCTCCCTGACTGCAAACTTCCGCTACACCTACGAGGGAAGCCACAT
CAAAGGAGAGTTTCAGGTGATCGGGTCCGGTTTCCCTGCTGACGGCCCTGTGATG
ACCAACTCGCTGACCGCTGTGGACTGGTGCGTGGCCAAGATGCTGTACCCCAACG
ACAAGACCATCATAAGCACCTTTGACTGGACCTACACCACTGGAAGTGGCAAGCG
CTACCAGAGCACAGTGCGGACCAACTACACCTTTGCCAAGCCAATGGCGGCCAAC
ATCCTGAAGAACCAGCCGATGTTCGTGTTCCGCAAGACGGAGCTCAAGCACTCCA
AGACCGAGCTCAACTTCAAGGAGTGGCAAAAGGCCTTTGCCGATGTGATG

>Branchiostoma_lanceolatum|FPY6|gi|169125802|gb|EU482392.1|
ATGTCTCTCCCAGCGACCCACGAGTTACACATCTTTGGCTCCATCAATAGTTTGGA
GTTTGACCTGGTGGGTCGTGGCACCGGCAACCCAAAGGAAGGTTATGAGGAACTC
CACCTGAAGTCCACCAAGAGTGCCCTCCAGTTCTCCCCATGGATCCTGGTCCCTC
AAATCGGGTACGGCTTTTACCAGTACCTGCCCTTCCCCGATGGAGCGATGTCGCC
TTTTCAGGCCGCTATGAACGATGGCTCCGGATACCAAGTCCATCGCACGATGCAG
TTTGAAGACGGTGCAACCCTGACTGGCATCTACCGCTATACCTACGAGGGAACCC
ACATCAAAGGAGAGTTTCAGGTGATCGGGACTGGTTTCCCTGCTGACGGCCCTGT
GATGACCAACTCGCTGACCGCTGCGGACTGGTGCGTGACCAAGATTGTATACCCG
AACGAGAATACCATCATCGACAAATTCGACTGGACCTACACCACTACAAGTGGCAA
GCGCTACCAGAGCAATGTGCGGTCCAACTTCACCTTTGCCAAGCCGATCGCGGCC
AACATCCTGCAGAAGCAGCCGATGTTCGTGTTCCGTAAGACGGAGCTAAAGCACT
CCAAGACCGAGCTCAACTTCAAGGAGTGGCAGACGGCCTTTAGCGATGTGATG

>Branchiostoma_lanceolatum|FPY5|gi|169125800|gb|EU482391.1|
ATGCCTCTCCCAGCAACACACGAGTTACACATCTTTGGTTCCTTCAATGGTGTGGA
CTTTGACATGGTGGGTCATGGCACCGGCAATCCAAATGATGGTTATGAGGAGTTAA
ACCTGAAGTCCACCAAGGGTGCCCTCCAGTTCTCCCCCTGGATCCTGGTCCCTCA
AATCGGGTATGGCTTCCACCAGTACCTGCCCTTCCCCGACGGGATGTCGCCTTTT
CAGGCTGCCATGAAAGATGGCTCAGGATACCAAGTCCATCGCACAATGCAGTTTG
AAGACGGTGCCTCCCTGACTTCCAACTACCGCTACACCTACGAGGGAAGCCACAT
CAAAGGAGAGTTTCAGGTGAACGGGACTGGTTTCCCTGCTGACGGCCCTGTGATG
ACCAACTCGCTGACCACTGCGGACTGGTGCGTGACCAAGATGCTGTATCCTAACG
ACAAGACCATCATCAGCACCTTTGACTGGACCTACACCACTGGAAGTGGCAAGCG
CTACCAGAGCACAGTGCGGACCAACTACACCTTTGCCAAGCCAATGGCGGCCAAC
ATCCTGCAGAACCAGCCGATGTTCGTGTTCCGCAAGACGGAGCTCAAGCACTCCA
AGACCGAGCTCAACTTCAAGGAGTGGCAAAAGGCTTTTGCCGATGTGATG

>Branchiostoma_lanceolatum|FPY4|gi|169125798|gb|EU482390.1|
ATGCCTCTCCCAGCAACACATGAGTTACACATCTTTGGCTCCTTCAACGGTGTGGA
CTTTGACATGGTGGGTCGTGGCACCGGCAATCCAAATGATGGTTATGAGGAGTTA
AACCTGAAGTCCACCAAGGGTGCCCTCCAGTTCTCCCCCTGGATCCTGGTCCCGC
AAATCGGGTATGGCTTCCATCAGTATCTGCCCTTCCCCGACGGGATGTCACCTTTC
CAGGCTGCCATGAAAGATGGCTCCGGATACCAAGTCCATCGCACAATGCAGTTTG
AAGACGGTGCCTCCCTGACTTCCAACTACCGCTACACCTACGAGGGAAGCCACAT
CAAAGGAGAGTTTCAGGTGATCGGGACTGGTTTCCCTGCTGACGGTCCTGTGATG
ACCAACTCGCTGACCACTGCGGACTGGTGCGTGACCAAGATGCTGTACCCCAACG
ACAAAACCATCATCAGCACCTTTGACTGGACCTACAACACTGCAAGTGGCAAGCGC
TACCAGAGCACGGTGCGAACCAACTACACCTTTAACAAGCCAATGGCGGCCAACA
TCCTGAAGAACCAGCCGATGTTCGTGTTCCGCAAGACGGAGCTCAAGCACTCCAA
GACCGAGCTCAACTTCAAGGAGTGGCAAATGGCCTTTGCCGATGTGATG

>Branchiostoma_lanceolatum|FPY3|gi|169125796|gb|EU482389.1|
ATGTCTCTCCCAGCGACACATGAGTTACACATCTTTGGCTCCTTCAACGGTGTGGA
CTTTGACATGGTGGGTCGTGGCACCGGCAATCCAAATGATGGTTATGAGGAGTTA
AACCTGAAGTCCACCAAGGGTGCCCTCCAGTTCTCCCCCTGGATCCTGGTCCCTC
AAATCGGGTATGGCTTCCATCAGTACCTGCCCTTCCCCGACGGGATGTCGCCTTTC
CAGGCCGCCATGAAAGATGGCTCCGGATACCAAGTCCATCGCACAATGCAGTTTG
AAGACGGTGCCTCCCTGACTTCCAACTACCGCTACACCTACGAGGGAAGCCACAT
CAAAGGAGAGTTTCAGGTGATCGGGACTGGTTTCCCTGCTGACGGTCCTGTGATG
ACCAACTCGCTGACCGCTGCGGACTGGTGCGTGACCAAGATGCTGTACCCCAACG
ACAAAACCATCATCAGCACCTTTGACTGGACTTACACCACTGGAAGTGGCAAGCGC
TACCAGAGCACAGTGCGGACCAACTACACCTTTGCCAAGCCAATGGCGGCCAACA
TCCTGAAGAACCAGCCGATGTTCGTGTTCCGTAAGACGGAGCTCAAGCACTCCAA
GACCGAGCTCAACTTCAAGGAGTGGCAAAAGGCCTTTACCGATGTGATG

>Branchiostoma_lanceolatum|FPY2|gi|169125794|gb|EU482388.1|
ATGTCTCTCCCAGCGACCCACGAGTTACACATCTTTGGCTCCATCAATAGTTTGGA
GTTTGACCTGGTGGGTCGTGGCACCGGCAACCCAAGGGAAGGTTATGAGGAACTC
CACCTGAAGTCCACCAAGAGTGCCCTCCAGTTCTCCCCATGGATCCTGGTCCCTC

AAATCGGGTACGGCTTTTACCAGTACCTGCCCTTCCCCGATGGAGCGATGTCGCC
TTTTCAGGCCGCTATGAACGATGGCTCCGGATACCAAGTCCATCGCACAATGCAGT
TTGAACACGGTGCAACCCTGACTGGCATCTACCGCTATACCTACGAGGGAACCCA
CATCAAAGGAGAGTTTCAGGTGATCGGGACTGGTTTCCCTGCTGACGGCCCTGTG
ATGACCAACTCGCTGACCGCTGCGGACTGGTGCGTGACCAAGATTGTATACCCGA
ACGAGAATACCATCATCGACAAATTCGACTGGACCTACACCACTACAAGTGGCAAG
CGCTACCATAGCAATGTGCGGTCCAACTTCACCTTTGCCAAGCCGATCGCGGCCA
ACATCCTGCAAAAGCAGCCGATGTTCGTGTTCCGTAAGACGGAGCTAAAGCATTCC
AAGACCGAGCTCAACTTCAAGGAGTGGCAGACGGCCTTTGGCGATGTGATG

>Branchiostoma_lanceolatum|FPY1|gi|169125792|gb|EU482387.1|
ATGCCTCTCCCAGCAACCCACGAGTTACACATCTTTGGCTCCATCAATAGTTTGGA
GTTTGACCTGGTGGGTCGTGGCACCGGCAACCCAAAGGAAGGTTATGAGGAACTC
CACCTGAAGTCCACCAAGAGTGCCCTCCAGTTCTCCCCATGGATCCTGGTCCCTC
AAATCGGGTACGGCTTTTACCAGTACCTGCCCTTCCCCGATGGAGCGATGTCGCC
TTTTCAGGCCGCTATGAACGATGGCTCCGGATACCAAGTCCATCGCACAATGCAGT
TTGAAGACGGTGCAACCCTGACTGGCATCTACCGCTATACCTACGAGGGAACCCA
CATCAAAGGAGAGTTTCAGGTGATCGGGACTGGTTTCCCTGCTGACGGCCCTGTG
ATGACCAACTCGCTGACCGCTGCGGACTGGTGCGTGACCAAGATTGTATACCCGA
ACGAGAATACCATCATCGACAAATTCGACTGGACCTACACCACTACAAGTGGCAAG
CGCTACCAGAGCAATGTGCGGTCCAACTTCACCTTTGCCAAGCCGATCGCGGCCA
ACATCCTGCAGAAGCAGCCGATGTTCGTGTTCCGTAAGACGGAGCTAAAGCACTC
CAAGACCGAGCTCAACTTCAAGGAGTGGCAGACGGCCTTTAGCGATGTGATG

Supplementary file 2: The protein sequences of the 49 cephalochordate GFP-encoding genes used in this study (in FASTA format).

>Asymmetron_lucayanum|GFP2|asym20h_comp64813_c0_seq2_m.13123
DSQLPQLASDAQLSSIMAIPTSHDTHIFGSINGVEFDMVGGGTGNPKDGSVNSTVKST
KGGLPCSPLLIGPHLGYGLYQYLPFPDGPSPFQADMGYEVLRTMKFEDGAVLSANYR
YSYQGGKIKSEHKLVGSGFPADGPVLKNKLACLDRSVTKLKYVDDRNLVDTVDWAYS
TTDGKKYNAVVQTNYSWGNPSNAAFKDKMPAFVFRQIDVSGSKTEVHLVEKQKAFYD
LI

>Asymmetron_lucayanum|GFP1|asym20h_comp74545_c2_seq1_m.28460
MSLPTQHDCHIFGSINGIDFDMVGGGKGDPHKGILETTVRSTKGPLPFSPLILGPNLGY
GYHQYTPFPNGQSPYQNAVHDGGYKKHRTFTFEDGGILSITFNYTYEGNKIYGEFQCV
GTGFPDVGPVMTNALVGEHPNCEKLMISGANSIGSDNNWSFSTNQKGKYYKANVVTN
STFAKKLPADLEDIMPLFVFRKTQVKCTKTEITLHESEKSFSDL

>Branchiostoma_belcheri|GFPd2|005130R
MPTSHDLHIHGSINGLEFDMVGGGSGNPNDGSVETRVKSTKGGLQFSPLIVGPHLGY
GFYQYLPFPSGPSPYQAAMKDGGYKMHRTMQFEDGAVLTANYRYTYEGGKIKGDFH
LVGSGFPTNGPVMTNSLASQDRSVAKLTSVDDHTVVENIDWAYRTSDGGSYRAMVRT
NCTFGRPAAGVKGNMPMFVFRQLEISGSKSEISLQERQKVFSEV

>Branchiostoma_belcheri|GFPd3|005140R
MSLPTSHDLHIHGSINGLEFDMVGGGSGNPKDGSVETRVKSTKGGLQFSPLIVGPHLG
YGFYQYLPFPSGPSPYQAAMKDGGYKMHRTMQFEDGAVLTANYRYTYEGGKIKGEF
HLVGSGFPANGPVMTNSLASQDRSVAKLTSVDDHTVVENIDWAYRTSDGGSYRANVR
TNCTFGRPAAGVKGNMPMFVFRQLEISGSKSEISLQERQKVFSEV

>Branchiostoma_belcheri|GFPe1|144690R
MALPTNHDLHIFGSVNGLEFDMVGGGSGNPKAGTLETSVKSTRGALPFSPLLVAPNLG
YGFYQYLPFPDGPSPFQTAITDGGYEVNRVFKFEDGGVLSAHFRYTYEGGKIKGEFQL
VGSGFPASGPVMTSGLTSLDRSVAKLMCSDNRTLTGLNNWSFCTSDGQRHQAVVQT
NYTFAKPFPAGTAEKMPVFMGHQIEVNASKTEIALTEKAKAFCNTV

>Branchiostoma_belcheri|GFPf1|144780R
MMSLPTTHDCHIFGTINGHAFDMVGGGSGNPKDGSLQTTVRSTKGPLPFSGVILGPNL
GYGYHQYLPFPSGMSPYQNAIKNGGYEKHRTFQFEDGGVMSINFRYSYEGNKIKGEF
HVVGSGFPDDGPVMTNSLQAHDNNVERLQVLGDRTIGSDNVWTYTGKGKGGTYKAN
VMTNATFAQNLQPGLEAIMPLFVFRKVDIEASKTEITLVEREKVFSDLCHIEAAITGFYSD
RESEASVKVRHPVFSTIYKLATHQKPNTRSIQTSPANSAPIGRRPAADMGANQSRAMY
AASFAVAGAGLLYIFKDTLTGGLNASSRKNGKAEPVAPPVEKAKAPKPVAEAVVEKPA
APEVVAEVLEVAKPAAEVVPVAEAVVLEEVAAVAADVAAAAPEAVPEVAAAPEAVPEVI
AETAVPVAEVVEAAPEVAVEAPAPVVEAAVEEVAVVVEEVAAAPEAVPEVVAEEVAAA
PEAVQEVTVEVAAAPEAVQEVTVEVAAALEAVEEVVTEAAPEAVPEIKEEVAAAPEAV
QEVVTEAAPEAVTEVTVEVAAAPEAVTEVVTEAAPEAVTEVTDEVAAAPEVVKEVVAE
VAEAVEIAVAEPEPTAVVEETPAAAEDTPAAVAAEEPIPAEEPVAAVEDTPVVAATGPF

TVTTEFAEVPEPPLDATHCLVTAESSTPVVAETEAVAAEIPAAEESPAEATQPAAAAVD
VPAENGVPEEAPPTDEILPDKKAALADAVPSKATEGAAAS

>Branchiostoma_belcheri|GFPd4|145360F
MSFPTSHDLHIHGSINGLEFDMVGGGSGNPKDGSVETRVTSTKGDLPAFSPHIVAPHL
GYGFYQYLPFPGGPSPYQAAIKDGGKYNEHRTIQFEDGAVLTANYRYTYKGDKIKGDF
HLVGSGFPASGPVMTNSMASLDRTVAKITCVDEHYTSSGGSYRAMVLHHVIQHHRVF
ATAPVTIVTAIWFKSFTITTSSGGSYRAMVQTNCTFQKPAAGVQGNMPMFVFRQLEVK
ATKTEISLQEKQKAFTEVQ

>Branchiostoma_belcheri|GFPc1|199320R
MQIKMTIIHVFLQPVPATHELHVSGSINGREFDLAGRGTGNAKDGSEEIQVKSTKGALG
FSPVLLVPNLGYGFHQYLPYPDGMSPFQAAADDGSGYVVHRTLQFEDGGSVTGIYRY
SYDGSHIKGEFNVTGSGFPADGPVMTNSLTAVDPSVATVFCPNDTTVVSTIDWSCTTT
SGKRYHGTVRTNYTFAKPIAASFLQQQPMFVFRKTELKASDTELSLKESQKAFHGL

>Branchiostoma_belcheri|GFPf2|212280R
MPEALPLTHTVKALSLGLNAVLLLAINICIREAPSSTKAPEKVQEAAIDKGNDLSRVTPG
HPLTICHQWRDPGDVSRRAVSPPHLPPYTPAITINPQSSGKVHVQFPVLTFCSHLKPPE
GMAEYRYEIRYEWVITSLPTTHDCHIFGTINGHAFDMVGGGSGNPKDGSLQTTVRSTK
GPLPFSGVILGPNLGYGYHQYLPFPSGKSPYQNAIKNGGYEKHRTFHFEDGGVMSINF
RYTYEGNKIKGEFHVVGSGFPDDGPVMTNSVQAHDNNVERLQVLGDRTIGSDNVWTF
TGKGKGDTYNANVMTNATFAQNLQPGLESIMPLFVFRKVDIQASKTEITLVEREKVFSD
LCQHLLRGACREGLGEGVVRLDHRLVALPISGADKLGEIGKEFIITISIIAIITISSSISIISDS
VFSIIVFSTRASHLCRPVGQPYVNAPV

>Branchiostoma_belcheri|GFPb1|233630F
MECPTTRKPALFSPYVLAPHFGYGYDQYLPFPDQGVSPFQATMQGSSGYQVIRTVHF
EDGAILSAHFRYTYEGSHIKGEFQVIGGGFPADGPVMTKSLTAMDWSVAKLVSPNDNT
VQSTIDMAYTTTSGKRYQSTVRNIHTFAKPMTASILQQKPVFVFRKTDLKANKTEVTFK
EWQKAFTDVM

>Branchiostoma_belcheri|GFPd5|233640F
MGSTGGPQPYMSSIVRSALTFQSIPTSHDLHIHGSINGLEFDMVGGGSGNPKDGSVES
RVTSTKGDLPAFSPHIVAPHLGFGFYQYLPFPGGPSPYQAAIDVGGKYNEHRTIQFED
GAVLTANYRYTYKGDKIKGDFHLVGSGFPASGPVMTNSLASLDRTVAKITCVDEHSLV
DNIDWTYRTSSGGSYRAMVQTNCTFQKPVRGVQGNMPMFVFRQLEVKATKTEISLQE
KQKAFTEVQTLGPKMKKLNPFKKWQEKEKEKEKEKEKEKEKEKEKEKEKEKKHQKKQ
YGFTTPLRGVVNP

>Branchiostoma_belcheri|GFPb3|240030F
MLTWEVRADVNLGATHEIHVFGSLNGIEFDMVGRGSGNPKDGSQELQVKSTKGSLPF
SAQILIPHFGYGFYQYLPYPDGMSPFQAAMHDGKGYKVSRTMMFQDGAIMDAQYRYT
YEGSHIKGEMQVVGTGFPADGPVMTKSLKAADWNVTKMVYPDNKTIRATYDWTYTTT
SGKRYQSSVRTNHTFASPIAANILQQEPMFVFRKTELKGNAQDTEFTFRELQKAFSDLI
DLYTKKLESDNPFDLLTIYATHEIHIYGSFNGVEFDMVGRGTGNPKDGSEELHMKSTKG

PLPFSAQILIPHVGYGYHQYLPYPDGMSPFQAAMQDGSGYQVTRIMQFEDGANVTGH
YRYTYEGSHIKGDMQVIGTGFPADGPVMTKSLTAVDWCVAKNVHPDNKTIKATFDWTL
TTTSGKRYQASARTNYTFAKPMAANFLQQEPMFVFRKTAIQGNADDTEFAFKEEQKAF
ADLM

>Branchiostoma_belcheri|GFPb2|264830F
MSSRLLCFFGRKKKLASHELHIFGSINGVEFDMVGHGTGNPKDGSQELQVKSTKGPLP
FSPYVLAPHFAYGYDQYLPFPDQGVSPFQATMQGSSG

>Branchiostoma_belcheri|GFPx1|276530F
MAMTRCLTLVVVTAFMFARGGYANILHCLYSSQQTFHCMNGDCVPQEYVCDGWYDC
GDWSDELSGCRTRATLDITTRQLSPSALPHKSTKMAQHATQGDNIKYSTGLGIGGNAD
TQKEDEKAGARTKLPAAIGGTVGAVALLGLLPLPATHELHIYGSINGHEFDMVGGGDG
NPKDGHMQTNVKSTKGPLAFSPYIVTPHLGYGFYQYLPFPNGEMSPFQAAMQDGGY
EIHRTFRFEDGAMLSANYKYFFDGTHIKGEFCLTGSGFPANGPVMTSAITGVDDTVSR
MVPLDNNMLFDAFSWGYRTSSGKTYEAMVNTNYQFGKPIPAGIKSKLPMFVFRKLDIK
VSKTEVTLVEWQKAFSEPHLTSSLSAEMKTEEQAPLLRRDSAASPAGVAAVPRRSWR
QLLMNGSILLGREFCYALEAALVLPVLMTIGMPREMYRFFGLCYDSVHNILSNILGMNK
LSAYIVWLIPPVFGFIFVPLLGSVSDHCRCRWGRRRPFILALGLAIILGFALFLNGDALVT
LIAGDVSNSPKRADRNTMRTATLAVSMFGAILFDFAADFIESPIKAYLLDNCVERDRRR
GLDMQGALSGLGGFLGYATGAIDWIDLGIPPGSEYHVIFGISCSVFTLCLLLNLVSIREV
PLDDLTANSPDNGDRKPGREEPVPPQNPEAVVALDPKYGVMYMSDDPDLLQDVMTC
DDGLQKVLVTTVFGGGDQSDDGYGSIAQSEEGSIDTAEDTDTTELAQRLSITAYFRSIL
RMPKELACLCVSHFLGWASFLSVMLFFTDFMGRGVYKGNPSAPVDSPDRVLYEQGV
MIGCWGLTINAASCALYSSVCQSILRMPKELACLCVSHFLGWASFLSVMLFFTDFMGR
GVYKGNPSAPVDSPDRVLYEQGVMIGCWGLTINAASCALYSMLFGRILNHLSYRTMYI
FGYLAFGAGIGSMAIIAQLTEVRWEIIFLCPVMGIMYGTLNNIPYKLISRYHTSQTYIRTG
VDGSERRGMGIDCALVSSQNQLSQIVIGASMGSIVAAVGSVISVTVCSSVLAFTACIAAA
LLVHYGVDRREDGEPDVEDLLSMCFGRILNHLSYRTMYIFGYLAFGAGIGSMAIIAQLTE
VRWEIIFLCPVMGIMYGTLNNIPYKLISRYHTSQTYIRCGVDGSERRGMGIDCALVSSQ
NQLSQIVIGASMGSIVAAVGSVISVTVCSSVLAFTACIAAALLVHYDVDRREDGEPDVED
LLSSM

>Branchiostoma_belcheri|GFPd1|282900F
MSLPTSHDLHIHGSINGLEFDMVGGGSGNPKDGSVETRVKSTKGGLAFSPLIVGPHLG
YGFYQYLPFPSGPSPYQAAIKDGGYNEHRTMQFEDGAVLTANYRYTYEGGKIKGDFH
LVGSGFPASGPVMTNSLASLDRSVAKLTSVDDHTVVENIDWAYRTSSGDTYRAMVRT
NCTFGRPAAGVKGNMPMFVFRQLEVAGSKTEISLQERQKAFSEVQ

>Branchiostoma_floridae|GFPa1|63256
MPLPATHDIHLHGSINGHEFDMVGGGKGDPNAGSLVTTAKSTKGALKFSPYLMIPHLG
YGYYQYLPYPDGPSPFQTSMLEGSGYAVYRVFDFEDGGKLTTEFKYSYEGSHIKADM
KLMGSGFPDDGPVMTSQIVDQDGCVSKKTYLNNNTIVDSFDWSYNLQNGKRYRARVS
SHYIFDKPFSADLMKKQPVFVYRKCHVKASKTEVTLDEREKAFYELA

>Branchiostoma_floridae|GFPa2|63262
MPLPATHEIHLHGSVNGHEFDLVGSGKGDPKAGSLVTEVKSTMGPLKFSPHLMIPHLG
YGYYQYLPYPDGPSPFQTAMLDGSGYKVHRVFNFEDGGVLSIDYNYAYEGTHIKSDFK
LMGSGFPDDGPVMTSQIVDQDGCVSKKTYLNDNTIVDSFDWSYNLQNGKRYRARVTS
NYIFGKPLAADVMKKQPVFVYRKCYVKSTQTEITLDEREKAFYEVV

>Branchiostoma_floridae|GFPb1|75522
MPLPKTHELHIFGSFNGVEFDMVGRGIGNPNEGSEELNAKFTKGPLKFSPYILVPHLGY
AYYQYLPFPDGMSPFQAAMHDGSGYQVHRTIQYEDGASVTAHYRYTYEGSHIKGEFQ
VIGTGFPPDGPVMTNKLTAMDWSVTKMLYPNDKTILSTVDCSYTTTEGKRYQSKMRE
NNTFAKPMAADILQKQPMFVFRKTELQHSKTELTFKEWQKAFTDVM

>Branchiostoma_floridae|GFPb2|75521
MPLPKTHELHIFGSFNGVEFDMVGEGTGNPNEGSEELKLKSTNGPLKFSPYILVPHLG
YAFNQYLPFPDGMSPFQAAMQDESGYQVHRTLQYEDGAFVTANLRYTYEGSHIKGEF
QVIGTGFPPDGPVMTNKLTAMDWSVVKFVYPNDKTILSTFDKTYTTTEGKRYQCTFRE
NSTFAKPMAADILQKQPMFIFHKTELQHSNNAELTFKEKQTAFSDMK

>Branchiostoma_floridae|GFPb3|75519
MPLPTTHELHIFGTFNGVEYDMVGRGKGNPNDGYEELNLKSTKGPLKFSPWILVPQIG
YGFHQYLPYPDGMSPFQAAMHDGSGYQVHRTLDFEDGATLTADFRYTYEGSHIKGEF
KVIGTGFPADGPVMTNKLTAADWCVNKMLYPDDKTINSTFDWSYTTSEGKRYQSTVR
ENYTFAKPMAANILQKQPMFVFRKTELQHSKTELTFKEWQKAFTDVM

>Branchiostoma_floridae|GFPb4|75520
MPLPTTHELHIFGSFNGVEFDLVGRGEGNPKDGSQNLHLKSTKGPLQFSPWMLIPHIG
YGFYQYLPYPDGEMSPYQAAMYGGSGYLMHRTMQYEDGAKISGHYKYTYEGSHVKG
EFQLIGTGFPTDGPVMTNQLTAADWCVDKLLYPNDKTIISKFDWSYTTTDGKRYQAKV
QTNFDFAKPMAANYLQKQPMFVFRKVELEHSKTEVKFKQWQKAFHDIM

>Branchiostoma_floridae|GFPc1|75523
MPLPTTHEVHVYGSINGVEFDLVGSGKGNPKDGSEEIQVKSTKGPLGFSPYIVVPNIGY
GFHQYLPFPDGMSPFQAAADDGSGYVVHRNIQFEDGASLTGIYRYSYDAGHIKGEFR
VVGSGFPADGPVMTKSLTAVDWSVATMLFPNDTTVVSTIDWTCPTTSGKRYHATVRT
NYTFAKPIAGSILQKQPMFVFRKTEVKASDSEINLKESQKAFHDLV

>Branchiostoma_floridae|GFPd1|86184
MSVPTNLDLHIYGSINGMEFDMVGGGSGNPNDGSLSVNVKSTKGALRVSPLLVGPHL
GYGHYQYLPFPDGPSPFQAAVNNGGYQMHRSFNFEDGAVLTATYNYSYSGGKIQGE
FHLVGSCFPDDSPVMTNALTGLDRSVAKLMCVSDDKLAEFVDWTYRTSSGGRYRATV
QTNFTFAKPIAAGLKNNMPMFVFRQLEVTGSKTEISLQEQQKAFSTVL

>Branchiostoma_floridae|GFPd2|126982
MSVPTNLDLHIYGSINGMEFDMVGGGSGNPKDGSLAVNVKSTKGALCVSPLLVGPHL
GYGHYQYLPFPDGASPFQAAVNNGGYQMHRSFNFEDGAVLTATYNYSYSGGKIQGE

FHLVGSGFPDDSPVMTNALTGLDRSVSKLMCTSDDKLVESVHWSYRTSSGGRYRATV
QTNFTFAKPIEAGLKNNMPMFVFRQLEVTGSKTEIGLQEQQKAFSTVL

>Branchiostoma_floridae|GFPe1|63257
MSLPTTHDLHIFGSVNGAEFDLVGGGRGNPNDGTLETSVKSTRGALPCSPLLIGPNLG
YGFYQYLPFPGGASPFQTAITDGGYQVHRVFKFEDGGVLNCNFRYTYEGGKIKGEFQ
LIGSGFPAGGPVMSGGLTTLDRSVAKLQCSDDRTITGTNNWSFCTTDGKRHQADVQT
NYTFAKPLPAGLKEKMPIFLGHQIEVKASKTEITLSEKVKAFIDTV

>Branchiostoma_floridae|GFPe2|63260
MSLPTAHDLHIFGSVNGAEFDLVGGGKGNPNDGTLETSVKSTRGALPCSPLLIGPNLG
YGFYQYLPFPGGASPFQTAITDGGYQVHRVFKFEDGGVLNCNFRYTYEGGKIKGEFQ
LIGSGFPAGGPVMSGGLTTLDRSVAKLQCSDDCTITGTNNWSFCTTDGKRHQADVQT
NYIFAKPLPAGLKEKMPIFLGHQIEVKASKTEITLCEKVKAFIDTV

>Branchiostoma_floridae|GFPe3|63258
MSLPTTHDLHIFGSVNGAEFDLVGGGKGNPNDGTLETSVKSTRGALPCSPLLIGPNLG
YGFYQYLPFPGGASPFQTAITDGGYQVHRVFKFEDGGVLSCNFRYTYEGGKIKGEFQL
IGSGFPAGGPVMSGGLTTLDRSVAKLQCSDDCTITGTNNWSFCTTDGKRHQADVQTN
YTFAKPLPAGLKEKMPIFLGHQIEVQASKTEINLSEKVKAFIDTV

>Branchiostoma_floridae|GFPf1|63259
MSLPTAHDCHMFGSINGHEFDLVGGGNGNPNDGTLETKVRSTKGALPFSPVILAPNLG
YGYHQYLPFPAGTSPYQQAITNGVYQKHRTFKFEDGGVMTINFRYTYSGNKIKGEFHV
VGSGFPDDGPVMTNSLQQHDHNVERLMVLGDKTIGSDNMWTFTEKGGKDKRYKAEV
MTNATFAQNLQPGLKNVMPLFVFRKVDIDCSKTEVTLIEREKVFQDLLQ

>Branchiostoma_lanceolatum|FPX6|gi|169125832|gb|EU482407.1|
MSLPATHELHIFGKINGHEFDMRGKGTGNPNDGYEDLDLKSTKDDLPFSPWILVQNIG
YGFNQYLPYPDGAMSPFQAAMYNGSGYHVHREMEFEDGATLTGIYRYTYEGSHIKGE
FQVDGTGFPADGPVMTDSLTDLDWVVTKMVYPDDKTVFSTSDQTYTTTSGKGYQSTV
RTNNIFAEPIAADMMQSQPVFVSRK

>Branchiostoma_lanceolatum|FPX5|gi|169125830|gb|EU482406.1|
MSLPATHELHIFGKINGHEYDMRGKGTGNPNDGYEDLDLKSTKDDLPFSPWILVQNIG
YGFNQYLPYPDGAMSPFQAAMCDGSGYEVHREMEFEDGATVTGIYRYTYEGSHIKGE
FQVDGTGFPDDGPVMTDSLTDLDWVVTKMVYPDEKTVFSTSDQTYTTTSGKGYKSTV
RTNNIFAKPIAADMMQSQPVFVSRK

>Branchiostoma_lanceolatum|FPX4|gi|169125828|gb|EU482405.1|
MSLPKTHELHIFGKINGHEYDMRGKGTGNPNDGYEDLDLKSKDDLPFSPWILVQNIGY
GFNQYLPYPDGAMSPFQAAMCDGSGYEVHREMEFEDGATLTGIYRYTYEGSHIKGEF
QVDGTGFPDDGPVMTDSLTDLDWVVTKMVYPDEKTVFSTSDQTYTTASGKGYKSTV
RTNNIFAKPMAADMMQNQPIFVSRK

>Branchiostoma_lanceolatum|FPX3|gi|169125826|gb|EU482404.1|
MSLPATHELHIFDKINGHEFDMRGKGTGNPNDGYEDLDLKSTKDDLPFSPWILVQNIG
YGFNQYLPYPDGAMSPFQAAMYNGSGYHVHREMGFEDGATVTGIYRYTYEGSHIKG
EFQVDGTGFPADGPVMTNSLTDQDWSVTKMMYLDNKTVTSTADQTYTTASGKRYQG
TVRTNNTFAKPIAANILQKQPVFVSRK

>Branchiostoma_lanceolatum|FPR9|gi|169125824|gb|EU482403.1|
MSLPKTHDLHISGSVNGHEFDLEGSGKGDAKEGYQELHLKSNRGDLSFSPWILVPNIG
YGFYQYLPFPDGAMSPYQAAMHDGSGYVMHRAMRFEDGAMLHSDHRYTYNGNNIK
GEFRLTGSGFPADGPVMTNSLTAADWCVDKLLYPNENTIIGKFDWTYTTTSGKRYQSD
VQTNVTFGKPISADILKKQPMFVFRKVELKHSKTELNFKQWQKAFQDIV

>Branchiostoma_lanceolatum|FPR8|gi|169125822|gb|EU482402.1|
MSLPATHDLHISGSINGHEFDLEGSGKGNAKEGYQELHLKSNKGDLSFSPWILVPNIGY
GFYQYLPFPDGAMSPYQAAMHDGSGYVMHRAMRFEDGAMLHSDHRYTYNGNHIKG
EFRLTGSGFPADGPVMTNSLTAADWCVDKLLYPDENTIIGKFDWTYTTTSGKRYQSDV
QTNVTFAKPISADILKKQPMFVFRKVELKHSKTELNFKQWQKAFQDIV

>Branchiostoma_lanceolatum|FPR7|gi|169125820|gb|EU482401.1|
MPLPATHDLHISGSINGHEFDLEGSGKGNAKEGYQELHLKSNKGDLSFSPWILVPNIGY
GFYQYLPFPDGAMSPYQAAMHDGSGYVMHRAMRFEDGAMLHSDHRYTYNGNHIKG
EFRLTGSGFPADGPVMTNSLTAADWCVDKLLYPDENTIIGKFDWTYTTTSGKRYQSDV
QTNVTFAKPISADILKKQPMFVFRKVELKHSKTELNFKQWQKAFQDIV

>Branchiostoma_lanceolatum|FPR6|gi|169125818|gb|EU482400.1|
MSLPKTHDLHISGSVNGHEFDLEGSGKGNAKEGYQELHLKSNRGDLSFSPWILVPNIG
YGFYQYLPFPDGAMSPYQAAMHDGSGYVMHRAMRFEDGAMLHSDHRYTYNGNNIK
GEFRLTGSGFPADGPVMTNSLTAADWCVDKLLYPNENTIIGKFDWTYTTTSGKRYQSD
VQTNVTFGKPISADILKKQPMFVFRKVELKHSKTELNFKQWQKAFQDIV

>Branchiostoma_lanceolatum|FPR5|gi|169125816|gb|EU482399.1|
MPLPATHDLHISGSINGHEFDLEGSGKGNAKEGYQELHLKSNKGDLSFSPWILVPNIGY
GFYQYLPFPDGAMSPYQAAMHDGSGYVMHRSMQFEDGAMLHSDHRYIYKGNHIKGE
FRLTGSGFPADGPVMTNSLTAADWCVDKLLYPNDNTIIGKFDWTYTTTSGKRYQSDV
QTNVTFGKPIAADILKKQPMFVFRKVELKHTKTELNFKQWQKAFQDIA

>Branchiostoma_lanceolatum|FPR4|gi|169125814|gb|EU482398.1|
MSLPATHDLHISGSINGHEFDLEGSGKGNAKEGYQELHLKSNKGDLSFSPWILVPNIGY
GFYQYLPFPDGAMSPYQAAMHDGSGYVMHRTMQFEDGAMLHSDHRYTYKGNHIKG
EFRLTGSGFPADGPVMTNSLTAVDWCVDKLLYPNENTIIGKFDWTYTTTSGKRYQSDV
QTNVTFAKPIAADILKKQPMFVFRKVELKHSKTELNFKQWQKAFQDIV

>Branchiostoma_lanceolatum|FPR3|gi|169125812|gb|EU482397.1|
MPLPATHDLHISGSINGHEFDLEGSGKGNAKEGYQELHLKSNRGDLSFSPWILVPNIGY
GYYQYLPSPG

>Branchiostoma_lanceolatum|FPR2|gi|169125810|gb|EU482396.1|
MSLPATHDLHISGSINGHEFDLEGSGKGNAKEGYQELHLKSNKGDLSFSPWILVPNIGY
GFYQYLPFPDGAMSPYQAAMHDGSGYVMHRSMQFEDGAMLHSDHRYIYKGNHIKGE
FRLTGSGFPADGPVMTNSLTAADWCVDKLLYPNDNTIIGKFDWTYTTTSGKRYQSDV
QTNVTFGKPIAADILKKQPMFVFRKVELKHTKTELNFKQWQKAFQDIA

>Branchiostoma_lanceolatum|FPR1|gi|169125808|gb|EU482395.1|
MPLPATHDLHISGSINGHEFDLEGSGKGNAKEGYQELHLKSNRGDLSFSPWILVPNIGY
GFYQYLPFPDGAMSPYQAAMHDGSGYVMHRTMQFEDGAMLHSDHRYTYKGNHIKG
EFRLTGSGFPADGPVMTNSLTAADWCVDKLLYPTENTLIGKFDWTYTTTSGKRYQSD
VQTNVTFAKPMAADILKKQPMFVFRKVELKHTKTELNFKQWQKAFQDIV

>Branchiostoma_lanceolatum|FPO2|gi|169125806|gb|EU482394.1|
MPLPATHDLHISGSINGHEFDLEGSGKGNEKEGYQELHLKSNKGDLSFSPWILVPNTG
YGFYQYLPFPDGAMSPYQAAMHDGSGYVMHRSMQFEDGAMLHSDHRYIYKGNHIKG
EFRLTGSGFPADGPVMTNSLTAADWCVDKLLYPNDNTIIGKFDWTYTTTSGKRYQSDV
QTNVTFGKPIAADILKKQPMFVFRMMELKHTKTELNFKQWQKAFQDIA

>Branchiostoma_lanceolatum|FPO1|gi|169125804|gb|EU482393.1|
MPLPATHELHIFGSFNGVEFDMVGRGTGNPNDGSEDLHLKSTKGALQFSPWILIPHIGY
GFHQYLPFPDGMSPFQAAMQDGSGYQVHRTMQFEDGASLTANFRYTYEGSHIKGEF
QVIGSGFPADGPVMTNSLTAVDWCVAKMLYPNDKTIISTFDWTYTTGSGKRYQSTVRT
NYTFAKPMAANILKNQPMFVFRKTELKHSKTELNFKEWQKAFADVM

>Branchiostoma_lanceolatum|FPY6|gi|169125802|gb|EU482392.1|
MSLPATHELHIFGSINSLEFDLVGRGTGNPKEGYEELHLKSTKSALQFSPWILVPQIGY
GFYQYLPFPDGAMSPFQAAMNDGSGYQVHRTMQFEDGATLTGIYRYTYEGTHIKGEF
QVIGTGFPADGPVMTNSLTAADWCVTKIVYPNENTIIDKFDWTYTTTSGKRYQSNVRS
NFTFAKPIAANILQKQPMFVFRKTELKHSKTELNFKEWQTAFSDVM

>Branchiostoma_lanceolatum|FPY5|gi|169125800|gb|EU482391.1|
MPLPATHELHIFGSFNGVDFDMVGHGTGNPNDGYEELNLKSTKGALQFSPWILVPQIG
YGFHQYLPFPDGMSPFQAAMKDGSGYQVHRTMQFEDGASLTSNYRYTYEGSHIKGE
FQVNGTGFPADGPVMTNSLTTADWCVTKMLYPNDKTIISTFDWTYTTGSGKRYQSTV
RTNYTFAKPMAANILQNQPMFVFRKTELKHSKTELNFKEWQKAFADVM

>Branchiostoma_lanceolatum|FPY4|gi|169125798|gb|EU482390.1|
MPLPATHELHIFGSFNGVDFDMVGRGTGNPNDGYEELNLKSTKGALQFSPWILVPQIG
YGFHQYLPFPDGMSPFQAAMKDGSGYQVHRTMQFEDGASLTSNYRYTYEGSHIKGE
FQVIGTGFPADGPVMTNSLTTADWCVTKMLYPNDKTIISTFDWTYNTASGKRYQSTVR
TNYTFNKPMAANILKNQPMFVFRKTELKHSKTELNFKEWQMAFADVM

>Branchiostoma_lanceolatum|FPY3|gi|169125796|gb|EU482389.1|
MSLPATHELHIFGSFNGVDFDMVGRGTGNPNDGYEELNLKSTKGALQFSPWILVPQIG
YGFHQYLPFPDGMSPFQAAMKDGSGYQVHRTMQFEDGASLTSNYRYTYEGSHIKGE

FQVIGTGFPADGPVMTNSLTAADWCVTKMLYPNDKTIISTFDWTYTTGSGKRYQSTVR
TNYTFAKPMAANILKNQPMFVFRKTELKHSKTELNFKEWQKAFTDVM

>Branchiostoma_lanceolatum|FPY2|gi|169125794|gb|EU482388.1|
MSLPATHELHIFGSINSLEFDLVGRGTGNPREGYEELHLKSTKSALQFSPWILVPQIGY
GFYQYLPFPDGAMSPFQAAMNDGSGYQVHRTMQFEHGATLTGIYRYTYEGTHIKGEF
QVIGTGFPADGPVMTNSLTAADWCVTKIVYPNENTIIDKFDWTYTTTSGKRYHSNVRS
NFTFAKPIAANILQKQPMFVFRKTELKHSKTELNFKEWQTAFGDVM

>Branchiostoma_lanceolatum|FPY1|gi|169125792|gb|EU482387.1|
MPLPATHELHIFGSINSLEFDLVGRGTGNPKEGYEELHLKSTKSALQFSPWILVPQIGY
GFYQYLPFPDGAMSPFQAAMNDGSGYQVHRTMQFEDGATLTGIYRYTYEGTHIKGEF
QVIGTGFPADGPVMTNSLTAADWCVTKIVYPNENTIIDKFDWTYTTTSGKRYQSNVRS
NFTFAKPIAANILQKQPMFVFRKTELKHSKTELNFKEWQTAFSDVM

Supplementary file 3: The trimmed protein sequence alignment used in the phylogenetic analysis for Fig. 3 (in FASTA format).

>Asymmetron_lucayanum|GFP2|asym20h_comp64813_c0_seq2_m.13123
MAIPTSHDTHIFGSINGVEFDMVGGGTGNPKDGSVNSTVKSTKGGLPCSPLLIGPHLGYG
LYQYLPFPDGPSPF---QADMGYEVLRTMKFEDGAVLSANYRYSYQGGKIKSEHKLVGSG
FPADGPVLKNKLACLDRSVTKLKYVDDRNLVDTVDWAYSTTDGKKYNAVVQTNYSWGNPS
NAFKDKMPAFVFRQIDVSGSKTEVHLVEKQKAFYD
>Asymmetron_lucayanum|GFP1|asym20h_comp74545_c2_seq1_m.28460
MSLPTQHDCHIFGSINGIDFDMVGGGKGDPHKGILETTVRSTKGPLPFSPLILGPNLGYG
YHQYTPFPNGQSPYQNAVHDGGYKKHRTFTFEDGGILSITFNYTYEGNKIYGEFQCVGTG
FPDVGPVMTNALVGEHPNCEKLMISGANSIGSDNNWSFSTNQKGKYYKANVVTNSTFAKK
LPLEDIMPLFVFRKTQVKCTKTLHESEKSFSDL--
>Branchiostoma_belcheri|GFPd2|005130R
--MPTSHDLHIHGSINGLEFDMVGGGSGNPNDGSVETRVKSTKGGLQFSPLIVGPHLGYG
FYQYLPFPSGPSPYQAAMKDGGYKMHRTMQFEDGAVLTANYRYTYEGGKIKGDFHLVGSG
FPTNGPVMTNSLASQDRSVAKLTSVDDHTVVENIDWAYRTSDGGSYRAMVRTNCTFGRPA
AGVKGNMPMFVFRQLEISGSKSEISLQERQKVFSE
>Branchiostoma_belcheri|GFPd3|005140R
MSLPTSHDLHIHGSINGLEFDMVGGGSGNPKDGSVETRVKSTKGGLQFSPLIVGPHLGYG
FYQYLPFPSGPSPYQAAMKDGGYKMHRTMQFEDGAVLTANYRYTYEGGKIKGEFHLVGSG
FPANGPVMTNSLASQDRSVAKLTSVDDHTVVENIDWAYRTSDGGSYRANVRTNCTFGRPA
AGVKGNMPMFVFRQLEISGSKSEISLQERQKVFSE
>Branchiostoma_belcheri|GFPe1|144690R
MALPTNHDLHIFGSVNGLEFDMVGGGSGNPKAGTLETSVKSTRGALPFSPLLVAPNLGYG
FYQYLPFPDGPSPFQTAITDGGYEVNRVFKFEDGGVLSAHFRYTYEGGKIKGEFQLVGSG
FPASGPVMTSGLTSLDRSVAKLMCSDNRTLTGLNNWSFCTSDGQRHQAVVQTNYTFAKPF
PATAEKMPVFMGHQIEVNASKTEIALTEKAKAFCN
>Branchiostoma_belcheri|GFPf1|144780R
MSLPTTHDCHIFGTINGHAFDMVGGGSGNPKDGSLQTTVRSTKGPLPFSGVILGPNLGYG
YHQYLPFPSGMSPYQNAIKNGGYEKHRTFQFEDGGVMSINFRYSYEGNKIKGEFHVVGSG
FPDDGPVMTNSLQAHDNNVERLQVLGDRTIGSDNVWTYTGKGKGGTYKANVMTNATFAQN
LQLEAIMPLFVFRKVDIEASKTMGANQSRAMYAAS
>Branchiostoma_belcheri|GFPd4|145360F
MSFPTSHDLHIHGSINGLEFDMVGGGSGNPKDGSVETRVTSTKGDLAFSPHIVAPHLGYG
FYQYLPFPGGPSPYQAAIKDGGYNEHRTIQFEDGAVLTANYRYTYKGDKIKGDFHLVGSG
FPASGPVMTNSMASLDRTVAKITCVDEHTIVTAISFTITTSSGGSYRAMVQTNCTFQKPA
AGVQGNMPMFVFRQLEVKATKTEISLQEKQKAFTE
>Branchiostoma_belcheri|GFPc1|199320R
QPVPATHELHVSGSINGREFDLAGRGTGNAKDGSEEIQVKSTKGALGFSPVLLVPNLGYG
FHQYLPYPDGMSPFAAADDGSGYVVHRTLQFEDGGSVTGIYRYSYDGSHIKGEFNVTGSG
FPADGPVMTNSLTAVDPSVATVFCPNDTTVVSTIDWSCTTTSGKRYHGTVRTNYTFAKPI
AAQQQPMFVFRKTELKASDTELSLKESQKAFHGL-
>Branchiostoma_belcheri|GFPf2|212280R
IRLPTTHDCHIFGTINGHAFDMVGGGSGNPKDGSLQTTVRSTKGPLPFSGVILGPNLGYG
YHQYLPFPSGKSPYQNAIKNGGYEKHRTFHFEDGGVMSINFRYTYEGNKIKGEFHVVGSG

FPDDGPVMTNSVQAHDNNVERLQVLGDRTIGSDNVWTFTGKGKGDTYNANVMTNATFAQN
LQLESIMPLFVFRKVDIQASKTIITISIIAIITIS
>Branchiostoma_belcheri|GFPb1|233630F
-----------------------------------MECPTTRKPALFSPYVLAPHFGYG
YDQYLPFPDQGVPFATMQGSSGYQVIRTVHFEDGAILSAHFRYTYEGSHIKGEFQVIGGG
FPADGPVMTKSLTAMDWSVAKLVSPNDNTVQSTIDMAYTTTSGKRYQSTVRNIHTFAKPM
TAQQKPVFVFRKTDLKANKTEVTFKEWQKAFTDVM
>Branchiostoma_belcheri|GFPd5|233640F
QSIPTSHDLHIHGSINGLEFDMVGGGSGNPKDGSVESRVTSTKGDLAFSPHIVAPHLGFG
FYQYLPFPGGPSPYQAAIDVGGYNEHRTIQFEDGAVLTANYRYTYKGDKIKGDFHLVGSG
FPASGPVMTNSLASLDRTVAKITCVDEHSLVDNIDWTYRTSSGGSYRAMVQTNCTFQKPV
RGVQGNMPMFVFRQLEVKATKTEISLQEKQKAFTE
>Branchiostoma_belcheri|GFPb3|240030F
GKIYATHEIHIYGSFNGVEFDMVGRGTGNPKDGSEELHMKSTKGPLPFSAQILIPHVGYG
YHQYLPYPDGMSPFAAMQDGSGYQVTRIMQFEDGANVTGHYRYTYEGSHIKGDMQVIGTG
FPADGPVMTKSLTAVDWCVAKNVHPDNKTIKATFDWTLTTTSGKRYQASARTNYTFAKPM
AAQQEPMFVFRKTAIQADDTEFAFKEEQKAFADLM
>Branchiostoma_belcheri|GFPb2|264830F
KKKLASHELHIFGSINGVEFDMVGHGTGNPKDGSQELQVKSTKGPLPFSPYVLAPHFAYG
YDQYLPFPDQGVPFATMQGSSG-------------------------------------

>Branchiostoma_belcheri|GFPx1|276530F
LPLPATHELHIYGSINGHEFDMVGGGDGNPKDGHMQTNVKSTKGPLAFSPYIVTPHLGYG
FYQYLPFPNGEMPFQAAMQDGGYEIHRTFRFEDGAMLSANYKYFFDGTHIKGEFCLTGSG
FPANGPVMTSAITGVDDTVSRMVPLDNNMLFDAFSWGYRTSSGKTYEAMVNTNYQFGKPI
PAIKSKLPMFVFRKLDIKVSKTEVTLVEWQKAFLS
>Branchiostoma_belcheri|GFPd1|282900F
MSLPTSHDLHIHGSINGLEFDMVGGGSGNPKDGSVETRVKSTKGGLAFSPLIVGPHLGYG
FYQYLPFPSGPSPYQAAIKDGGYNEHRTMQFEDGAVLTANYRYTYEGGKIKGDFHLVGSG
FPASGPVMTNSLASLDRSVAKLTSVDDHTVVENIDWAYRTSSGDTYRAMVRTNCTFGRPA
AGVKGNMPMFVFRQLEVAGSKTEISLQERQKAFSE
>Branchiostoma_floridae|GFPa1|63256
MPLPATHDIHLHGSINGHEFDMVGGGKGDPNAGSLVTTAKSTKGALKFSPYLMIPHLGYG
YYQYLPYPDGPSPFQTSMLEGGYAVYRVFDFEDGGKLTTEFKYSYEGSHIKADMKLMGSG
FPDDGPVMTSQIVDQDGCVSKKTYLNNNTIVDSFDWSYNLQNGKRYRARVSSHYIFDKPF
SAKKQPVFVYRKCHVKASKTEVTLDEREKAFYELA
>Branchiostoma_floridae|GFPa2|63262
MPLPATHEIHLHGSVNGHEFDLVGSGKGDPKAGSLVTEVKSTMGPLKFSPHLMIPHLGYG
YYQYLPYPDGPSPFQTAMLDGGYKVHRVFNFEDGGVLSIDYNYAYEGTHIKSDFKLMGSG
FPDDGPVMTSQIVDQDGCVSKKTYLNDNTIVDSFDWSYNLQNGKRYRARVTSNYIFGKPL
AAKKQPVFVYRKCYVKSTQTEITLDEREKAFYEVV
>Branchiostoma_floridae|GFPb1|75522
MPLPKTHELHIFGSFNGVEFDMVGRGIGNPNEGSEELNAKFTKGPLKFSPYILVPHLGYA
YYQYLPFPDGMSPFAAMHDGSGYQVHRTIQYEDGASVTAHYRYTYEGSHIKGEFQVIGTG
FPPDGPVMTNKLTAMDWSVTKMLYPNDKTILSTVDCSYTTTEGKRYQSKMRENNTFAKPM

AAQKQPMFVFRKTELQHSKTELTFKEWQKAFTDVM
>Branchiostoma_floridae|GFPb2|75521
MPLPKTHELHIFGSFNGVEFDMVGEGTGNPNEGSEELKLKSTNGPLKFSPYILVPHLGYA
FNQYLPFPDGMSPFAAMQDESGYQVHRTLQYEDGAFVTANLRYTYEGSHIKGEFQVIGTG
FPPDGPVMTNKLTAMDWSVVKFVYPNDKTILSTFDKTYTTTEGKRYQCTFRENSTFAKPM
AAQKQPMFIFHKTELQSNNAELTFKEKQTAFSDMK
>Branchiostoma_floridae|GFPb3|75519
MPLPTTHELHIFGTFNGVEYDMVGRGKGNPNDGYEELNLKSTKGPLKFSPWILVPQIGYG
FHQYLPYPDGMSPFAAMHDGSGYQVHRTLDFEDGATLTADFRYTYEGSHIKGEFKVIGTG
FPADGPVMTNKLTAADWCVNKMLYPDDKTINSTFDWSYTTSEGKRYQSTVRENYTFAKPM
AAQKQPMFVFRKTELQHSKTELTFKEWQKAFTDVM
>Branchiostoma_floridae|GFPb4|75520
MPLPTTHELHIFGSFNGVEFDLVGRGEGNPKDGSQNLHLKSTKGPLQFSPWMLIPHIGYG
FYQYLPYPDGEMPYAAMYGGSGYLMHRTMQYEDGAKISGHYKYTYEGSHVKGEFQLIGTG
FPTDGPVMTNQLTAADWCVDKLLYPNDKTIISKFDWSYTTTDGKRYQAKVQTNFDFAKPM
AAQKQPMFVFRKVELEHSKTEVKFKQWQKAFHDIM
>Branchiostoma_floridae|GFPc1|75523
MPLPTTHEVHVYGSINGVEFDLVGSGKGNPKDGSEEIQVKSTKGPLGFSPYIVVPNIGYG
FHQYLPFPDGMSPFAAADDGSGYVVHRNIQFEDGASLTGIYRYSYDAGHIKGEFRVVGSG
FPADGPVMTKSLTAVDWSVATMLFPNDTTVVSTIDWTCPTTSGKRYHATVRTNYTFAKPI
AGQKQPMFVFRKTEVKASDSEINLKESQKAFHDLV
>Branchiostoma_floridae|GFPd1|86184
MSVPTNLDLHIYGSINGMEFDMVGGGSGNPNDGSLSVNVKSTKGALRVSPLLVGPHLGYG
HYQYLPFPDGPSPFQAAVNNGGYQMHRSFNFEDGAVLTATYNYSYSGGKIQGEFHLVGSC
FPDDSPVMTNALTGLDRSVAKLMCVSDDKLAEFVDWTYRTSSGGRYRATVQTNFTFAKPI
AALKNNMPMFVFRQLEVTGSKTEISLQEQQKAFST
>Branchiostoma_floridae|GFPd2|126982
MSVPTNLDLHIYGSINGMEFDMVGGGSGNPKDGSLAVNVKSTKGALCVSPLLVGPHLGYG
HYQYLPFPDGASPFQAAVNNGGYQMHRSFNFEDGAVLTATYNYSYSGGKIQGEFHLVGSG
FPDDSPVMTNALTGLDRSVSKLMCTSDDKLVESVHWSYRTSSGGRYRATVQTNFTFAKPI
EALKNNMPMFVFRQLEVTGSKTEIGLQEQQKAFST
>Branchiostoma_floridae|GFPe1|63257
MSLPTTHDLHIFGSVNGAEFDLVGGGRGNPNDGTLETSVKSTRGALPCSPLLIGPNLGYG
FYQYLPFPGGASPFQTAITDGGYQVHRVFKFEDGGVLNCNFRYTYEGGKIKGEFQLIGSG
FPAGGPVMSGGLTTLDRSVAKLQCSDDRTITGTNNWSFCTTDGKRHQADVQTNYTFAKPL
PALKEKMPIFLGHQIEVKASKTEITLSEKVKAFID
>Branchiostoma_floridae|GFPe2|63260
MSLPTAHDLHIFGSVNGAEFDLVGGGKGNPNDGTLETSVKSTRGALPCSPLLIGPNLGYG
FYQYLPFPGGASPFQTAITDGGYQVHRVFKFEDGGVLNCNFRYTYEGGKIKGEFQLIGSG
FPAGGPVMSGGLTTLDRSVAKLQCSDDCTITGTNNWSFCTTDGKRHQADVQTNYIFAKPL
PALKEKMPIFLGHQIEVKASKTEITLCEKVKAFID
>Branchiostoma_floridae|GFPe3|63258
MSLPTTHDLHIFGSVNGAEFDLVGGGKGNPNDGTLETSVKSTRGALPCSPLLIGPNLGYG
FYQYLPFPGGASPFQTAITDGGYQVHRVFKFEDGGVLSCNFRYTYEGGKIKGEFQLIGSG
FPAGGPVMSGGLTTLDRSVAKLQCSDDCTITGTNNWSFCTTDGKRHQADVQTNYTFAKPL
PALKEKMPIFLGHQIEVQASKTEINLSEKVKAFID

>Branchiostoma_floridae|GFPf1|63259
MSLPTAHDCHMFGSINGHEFDLVGGGNGNPNDGTLETKVRSTKGALPFSPVILAPNLGYG
YHQYLPFPAGTSPYQQAITNGVYQKHRTFKFEDGGVMTINFRYTYSGNKIKGEFHVVGSG
FPDDGPVMTNSLQQHDHNVERLMVLGDKTIGSDNMWTFTEKGGKKRYKAEVMTNATFAQN
LQLKNVMPLFVFRKVDIDCSKTIEREKVFQDLLQ-
>Branchiostoma_lanceolatum|FP-X6|gi|169125832|gb|EU482407.1|
MSLPATHELHIFGKINGHEFDMRGKGTGNPNDGYEDLDLKSTKDDLPFSPWILVQNIGYG
FNQYLPYPDGAMSPFQAAMYNGYHVHREMEFEDGATLTGIYRYTYEGSHIKGEFQVDGTG
FPADGPVMTDSLTDLDWVVTKMVYPDDKTVFSTSDQTYTTTSGKGYQSTVRTNNIFAEPI
AAMMQSQPVFVSRK---------------------
>Branchiostoma_lanceolatum|FP-X5|gi|169125830|gb|EU482406.1|
MSLPATHELHIFGKINGHEYDMRGKGTGNPNDGYEDLDLKSTKDDLPFSPWILVQNIGYG
FNQYLPYPDGAMSPFQAAMCDGYEVHREMEFEDGATVTGIYRYTYEGSHIKGEFQVDGTG
FPDDGPVMTDSLTDLDWVVTKMVYPDEKTVFSTSDQTYTTTSGKGYKSTVRTNNIFAKPI
AAMMQSQPVFVSRK---------------------
>Branchiostoma_lanceolatum|FP-X4|gi|169125828|gb|EU482405.1|
MSLPKTHELHIFGKINGHEYDMRGKGTGNPNDGYEDLDLKS-KDDLPFSPWILVQNIGYG
FNQYLPYPDGAMSPFQAAMCDGYEVHREMEFEDGATLTGIYRYTYEGSHIKGEFQVDGTG
FPDDGPVMTDSLTDLDWVVTKMVYPDEKTVFSTSDQTYTTASGKGYKSTVRTNNIFAKPM
AAMMQNQPIFVSRK---------------------
>Branchiostoma_lanceolatum|FP-X3|gi|169125826|gb|EU482404.1|
MSLPATHELHIFDKINGHEFDMRGKGTGNPNDGYEDLDLKSTKDDLPFSPWILVQNIGYG
FNQYLPYPDGAMSPFQAAMYNGYHVHREMGFEDGATVTGIYRYTYEGSHIKGEFQVDGTG
FPADGPVMTNSLTDQDWSVTKMMYLDNKTVTSTADQTYTTASGKRYQGTVRTNNTFAKPI
AAILQKQPVFVSRK---------------------
>Branchiostoma_lanceolatum|FP-R9|gi|169125824|gb|EU482403.1|
MSLPKTHDLHISGSVNGHEFDLEGSGKGDAKEGYQELHLKSNRGDLSFSPWILVPNIGYG
FYQYLPFPDGAMSPYQAAMHDGYVMHRAMRFEDGAMLHSDHRYTYNGNNIKGEFRLTGSG
FPADGPVMTNSLTAADWCVDKLLYPNENTIIGKFDWTYTTTSGKRYQSDVQTNVTFGKPI
SAKKQPMFVFRKVELKHSKTELNFKQWQKAFQDIV
>Branchiostoma_lanceolatum|FP-R8|gi|169125822|gb|EU482402.1|
MSLPATHDLHISGSINGHEFDLEGSGKGNAKEGYQELHLKSNKGDLSFSPWILVPNIGYG
FYQYLPFPDGAMSPYQAAMHDGYVMHRAMRFEDGAMLHSDHRYTYNGNHIKGEFRLTGSG
FPADGPVMTNSLTAADWCVDKLLYPDENTIIGKFDWTYTTTSGKRYQSDVQTNVTFAKPI
SAKKQPMFVFRKVELKHSKTELNFKQWQKAFQDIV
>Branchiostoma_lanceolatum|FP-R7|gi|169125820|gb|EU482401.1|
MPLPATHDLHISGSINGHEFDLEGSGKGNAKEGYQELHLKSNKGDLSFSPWILVPNIGYG
FYQYLPFPDGAMSPYQAAMHDGYVMHRAMRFEDGAMLHSDHRYTYNGNHIKGEFRLTGSG
FPADGPVMTNSLTAADWCVDKLLYPDENTIIGKFDWTYTTTSGKRYQSDVQTNVTFAKPI
SAKKQPMFVFRKVELKHSKTELNFKQWQKAFQDIV
>Branchiostoma_lanceolatum|FP-R6|gi|169125818|gb|EU482400.1|
MSLPKTHDLHISGSVNGHEFDLEGSGKGNAKEGYQELHLKSNRGDLSFSPWILVPNIGYG
FYQYLPFPDGAMSPYQAAMHDGYVMHRAMRFEDGAMLHSDHRYTYNGNNIKGEFRLTGSG
FPADGPVMTNSLTAADWCVDKLLYPNENTIIGKFDWTYTTTSGKRYQSDVQTNVTFGKPI
SAKKQPMFVFRKVELKHSKTELNFKQWQKAFQDIV
>Branchiostoma_lanceolatum|FP-R5|gi|169125816|gb|EU482399.1|

MPLPATHDLHISGSINGHEFDLEGSGKGNAKEGYQELHLKSNKGDLSFSPWILVPNIGYG
FYQYLPFPDGAMSPYQAAMHDGYVMHRSMQFEDGAMLHSDHRYIYKGNHIKGEFRLTGSG
FPADGPVMTNSLTAADWCVDKLLYPNDNTIIGKFDWTYTTTSGKRYQSDVQTNVTFGKPI
AAKKQPMFVFRKVELKHTKTELNFKQWQKAFQDIA
>Branchiostoma_lanceolatum|FP-R4|gi|169125814|gb|EU482398.1|
MSLPATHDLHISGSINGHEFDLEGSGKGNAKEGYQELHLKSNKGDLSFSPWILVPNIGYG
FYQYLPFPDGAMSPYQAAMHDGYVMHRTMQFEDGAMLHSDHRYTYKGNHIKGEFRLTGSG
FPADGPVMTNSLTAVDWCVDKLLYPNENTIIGKFDWTYTTTSGKRYQSDVQTNVTFAKPI
AAKKQPMFVFRKVELKHSKTELNFKQWQKAFQDIV
>Branchiostoma_lanceolatum|FP-R3|gi|169125812|gb|EU482397.1|
MPLPATHDLHISGSINGHEFDLEGSGKGNAKEGYQELHLKSNRGDLSFSPWILVPNIGYG
YYQYLPSPG---

>Branchiostoma_lanceolatum|FP-R2|gi|169125810|gb|EU482396.1|
MSLPATHDLHISGSINGHEFDLEGSGKGNAKEGYQELHLKSNKGDLSFSPWILVPNIGYG
FYQYLPFPDGAMSPYQAAMHDGYVMHRSMQFEDGAMLHSDHRYIYKGNHIKGEFRLTGSG
FPADGPVMTNSLTAADWCVDKLLYPNDNTIIGKFDWTYTTTSGKRYQSDVQTNVTFGKPI
AAKKQPMFVFRKVELKHTKTELNFKQWQKAFQDIA
>Branchiostoma_lanceolatum|FP-R1|gi|169125808|gb|EU482395.1|
MPLPATHDLHISGSINGHEFDLEGSGKGNAKEGYQELHLKSNRGDLSFSPWILVPNIGYG
FYQYLPFPDGAMSPYQAAMHDGYVMHRTMQFEDGAMLHSDHRYTYKGNHIKGEFRLTGSG
FPADGPVMTNSLTAADWCVDKLLYPTENTLIGKFDWTYTTTSGKRYQSDVQTNVTFAKPM
AAKKQPMFVFRKVELKHTKTELNFKQWQKAFQDIV
>Branchiostoma_lanceolatum|FP-O2|gi|169125806|gb|EU482394.1|
MPLPATHDLHISGSINGHEFDLEGSGKGNEKEGYQELHLKSNKGDLSFSPWILVPNTGYG
FYQYLPFPDGAMSPYQAAMHDGYVMHRSMQFEDGAMLHSDHRYIYKGNHIKGEFRLTGSG
FPADGPVMTNSLTAADWCVDKLLYPNDNTIIGKFDWTYTTTSGKRYQSDVQTNVTFGKPI
AAKKQPMFVFRMMELKHTKTELNFKQWQKAFQDIA
>Branchiostoma_lanceolatum|FP-O1|gi|169125804|gb|EU482393.1|
MPLPATHELHIFGSFNGVEFDMVGRGTGNPNDGSEDLHLKSTKGALQFSPWILIPHIGYG
FHQYLPFPDGMSPFAAMQDGSGYQVHRTMQFEDGASLTANFRYTYEGSHIKGEFQVIGSG
FPADGPVMTNSLTAVDWCVAKMLYPNDKTIISTFDWTYTTGSGKRYQSTVRTNYTFAKPM
AAKNQPMFVFRKTELKHSKTELNFKEWQKAFADVM
>Branchiostoma_lanceolatum|FP-Y6|gi|169125802|gb|EU482392.1|
MSLPATHELHIFGSINSLEFDLVGRGTGNPKEGYEELHLKSTKSALQFSPWILVPQIGYG
FYQYLPFPDGAMPFAAMNDGSGYQVHRTMQFEDGATLTGIYRYTYEGTHIKGEFQVIGTG
FPADGPVMTNSLTAADWCVTKIVYPNENTIIDKFDWTYTTTSGKRYQSNVRSNFTFAKPI
AAQKQPMFVFRKTELKHSKTELNFKEWQTAFSDVM
>Branchiostoma_lanceolatum|FP-Y5|gi|169125800|gb|EU482391.1|
MPLPATHELHIFGSFNGVDFDMVGHGTGNPNDGYEELNLKSTKGALQFSPWILVPQIGYG
FHQYLPFPDGMSPFAAMKDGSGYQVHRTMQFEDGASLTSNYRYTYEGSHIKGEFQVNGTG
FPADGPVMTNSLTTADWCVTKMLYPNDKTIISTFDWTYTTGSGKRYQSTVRTNYTFAKPM
AAQNQPMFVFRKTELKHSKTELNFKEWQKAFADVM
>Branchiostoma_lanceolatum|FP-Y4|gi|169125798|gb|EU482390.1|
MPLPATHELHIFGSFNGVDFDMVGRGTGNPNDGYEELNLKSTKGALQFSPWILVPQIGYG

FHQYLPFPDGMSPFAAMKDGSGYQVHRTMQFEDGASLTSNYRYTYEGSHIKGEFQVIGTG
FPADGPVMTNSLTTADWCVTKMLYPNDKTIISTFDWTYNTASGKRYQSTVRTNYTFNKPM
AAKNQPMFVFRKTELKHSKTELNFKEWQMAFADVM
>Branchiostoma_lanceolatum|FP-Y3|gi|169125796|gb|EU482389.1|
MSLPATHELHIFGSFNGVDFDMVGRGTGNPNDGYEELNLKSTKGALQFSPWILVPQIGYG
FHQYLPFPDGMSPFAAMKDGSGYQVHRTMQFEDGASLTSNYRYTYEGSHIKGEFQVIGTG
FPADGPVMTNSLTAADWCVTKMLYPNDKTIISTFDWTYTTGSGKRYQSTVRTNYTFAKPM
AAKNQPMFVFRKTELKHSKTELNFKEWQKAFTDVM
>Branchiostoma_lanceolatum|FP-Y2|gi|169125794|gb|EU482388.1|
MSLPATHELHIFGSINSLEFDLVGRGTGNPREGYEELHLKSTKSALQFSPWILVPQIGYG
FYQYLPFPDGAMPFAAMNDGSGYQVHRTMQFEHGATLTGIYRYTYEGTHIKGEFQVIGTG
FPADGPVMTNSLTAADWCVTKIVYPNENTIIDKFDWTYTTTSGKRYHSNVRSNFTFAKPI
AAQKQPMFVFRKTELKHSKTELNFKEWQTAFGDVM
>Branchiostoma_lanceolatum|FP-Y1|gi|169125792|gb|EU482387.1|
MPLPATHELHIFGSINSLEFDLVGRGTGNPKEGYEELHLKSTKSALQFSPWILVPQIGYG
FYQYLPFPDGAMPFAAMNDGSGYQVHRTMQFEDGATLTGIYRYTYEGTHIKGEFQVIGTG
FPADGPVMTNSLTAADWCVTKIVYPNENTIIDKFDWTYTTTSGKRYQSNVRSNFTFAKPI
AAQKQPMFVFRKTELKHSKTELNFKEWQTAFSDVM
>Aequorea_victoria|GFP|gi|155662|gb|M62654.1|
FTGVVPILVELDGDVNGHKFSVSGEGEGDATYGKLTLKFICTTGKLPVPWPTLVTTFSYG
VQCFSRYPDHMKDFFKSAMPEGYVQERTIFYKDDGNYKSRAEVKFEGDTLVNRIELKGID
FKEDGNILGHK-MEYNYNSHNVYIMADKGIKVNFKIRHNIEDGSVQLADHYNTPIGDGPV
---LLPDNHYLSTQSALSKDPNHMILLEFVTAAGI
>Heteractis_magnifica|GFP|gi|28627997|gb|AY157666.1|
IKETMRSKVYMEGNVNNHAFKCTAEGEGKPYKGSQKLTITVTEGPLPFAFDILSHAFQYG
NKVFTKYPDDIPDFFKQSLSGGFTWKRVSNYEDGGVLTVDQKTSLEGDCIICNIKVHGTN
FPADGPVMQKQTNGWEPSTETVIPRGEGILLRDVPALKLRNNKGHLLCVMETTYKPNKRV
---NLPKLHFHHLRMEKDSISDTIKQHEDVRASYF
>Montastraea_cavernosa|GFP_G1|gi|51472050|gb|AY679109.1|
IKPDMKIKLRMEGAVNGHKFVIEGDGKGKPFEGKQTMDLTVIEGPLPFAYDILTTVFDYG
NRVFAKYPKDIPDYFKQTFPEGYSWERSMTYEDQGICIATNDITMMDDCFVYKIRFDGVN
FPANGPVMQRKTLKWEPSTEKMYVRDG-VLKGDVNMALLLEGGGHYRCDFKTTYKAKKVV
---QLPDYHFVDHRIEIVSPDKKVKLYEHAEAH-F
>Montastraea_cavernosa|GFP_G2|gi|51472054|gb|AAU04448.1|
IKPDMKIKLRMEGAVNGHNFVIEGEGKGKPFEGTQTINLTVKEGPLPFAYDILTAAFQYG
NRAFTKYPRDIADYFKQSFPEGYSWERSMTYEDQGICIIKSDIRMEGDCFIYEIRYDGVN
FPPSGPVMQKKTLKWEPSTEKMYVRDG-VLKGEVNMALLLEGGGHYRCDFRSTYKAKKRV
---QLPDYHFVDHRIEILSHDNTVKLSENAEARYS
>Phialidium_sp.SL-2003|YFP|gi|40365350|gb|AY485333.1|
FHGKIPYVVEMEGNVDGHTFSIRGKGYGDASVGKVDAQFICTTGDVPVPWSTLVTTLTYG
AQCFAKYGPELKDFYKSCMPEGYVQERTITFEGDGVFKTRAEVTFENGSVYNRVKLNGQG
FKKDGHVLGKNEFNFTPHCLYIWGDQANGLKSAFKIMHEITGSKVADHTQMNTPIGGGPV
---HVPEYHHITYHVTLSKDVTNMSLVETVRAVDC
>Renilla_muelleri|GFP|gi|12621059|gb|AY015996.1|
LQEVMSYKVNLEGIVNNHVFTMEGCGKGNILFGNQLVQIRVTKGPLPFAFDIVSPAFQYG
NRTFTKYPNDISDYFIQSFPAGFMYERTLRYEDGGLVEIRSDINLIEDKFVYRVEYKGSN

FPDDGPVMQKTILGIEPSFEAMYMNNG-VLVGEVILVYKLNSGKYYSCHMKTLMKSKGVV
--KEFPSYHFIQHRLEKTYVEDFVEQHETAIAQMT
>Anthomedusae_sp.SL-2003|GFP1|gi|40365352|gb|AY485334.1|
FQKKLPYKLELDGDVDGQTFKVIGEGVGDATTGVIEGKYVCTEGEVPISWVSLITSLSYG
AKCFVRYPNEINDFFKSTFPSGYHQERKITYENDGVLETAAKITMESGAIVNRINVKGTG
FDKDGHVCQKNLESSPPSTTYVVPEGE-GIRIIYRNIYPTKDGHYVVADTQQVNRPIRAQ
GT-AIPTYHHIKSKVDLSTDPEHIIIKETNCAFDA
>Anthomedusae_sp.SL-2003|GFP2|gi|40365354|gb|AY485335.1|
FEKPLPYKVELEGDVDGQKFTVIGEGQGDASTGRVEGKYVCTKGEVPISWVSLITSLSYG
GKCFVRYPNVIKDFFKSTFPTGYHQERKITYEDDGVLETAAKVTLESGAIYNRISVKGVG
FKKDGNVCKKRLHSSPPQVSYVVPYGE-GIRVLYSNIYPTKDGGYVVADTRQVNRPIKAE
GK-AIPKYHYIKSKIDLSTDPNHIIIKEVNVASGI
>Anthomedusae_sp.SL-2003|GFP-like_chromoprotein|gi|40365356|gb|AY485336.1|
FQSDMTFKIFIDGVVNDQKFTIIADGSSKFPHGDFNVHAVCETGKLPMSWKPICHLIQYG
EPFFAKYPNGISHFAQECFPEGLTIDRTVRFENDGTMTSHHTYELDGTCVISRITVNCDG
FQPDGPIMKDQLVDILPTETHMFPHGSNAVRQLCYIGFTTADGGLMMSHFDSKLTFNGSR
AI-KIPGPHFVTVIIKQMKDTSHVCQREVTYAHSV
>Clavularia_sp.|FP484|gi|6090876|gb|AF168424.1|
IKPDMKIKLKMEGNVNGHAFVIEGEGEGKPYDGTHTLNLEVKEGPLPFSYDILSNAFQYG
NRALTKYPDDIADYFKQSFPEGYSWERTMTFEDKGIVKVKSDISMEEDSFIYEIRFDGMN
FPPNGPVMQKKTLKWEPSTEIMYVRDG-VLVGDISHSLLLEGGGHYRCDFKSIYKAKKVV
---KLPDYHFVDHRIEILNHDKKVTLYENAVARYS
>Chiridius_poppei|GFP|gi|85658700|dbj|AB185173.1|
-MTTFKIESRIHGNLNGEKFELVGGGVGE--EGRLEIEMKTKDKPLAFSPFLLSHCMGYG
FYHFASFPKGTKNILHAATNGGYTNTRKEIYEDGGILEVNFRYTYEFNKIIGDVECIGHG
FPSQSPIFKDTIVKSCPTVDLMLPMSGNIIASSYARAFQLKDGSFYTAEVKNNIDFKNPI
HEFSKSGPMFTHRRVEETHTKEMVEYQQVFNSAPR
>Labidocera_aestiva|GFP|gi|33243029|gb|AY268073.1|
-MPVMKIECRISGTMNGEEFELVGAGDGNTDEGRMTNKMKSTKGPLSFSPYLLSHIMGYG
FYHYATFPAGYENVLHAAKNGGYTNTRTERYEDGGIISVNFTYRYEGNKVIGDFKVVGSG
FPANSVIFTDKIIKSNPTCEHIYPKGDNILVNAYTRTWMLRDGGYYSAQVNNHLHFKTAM
HPLQNGGSMFTYRKVEELHSQSGIVEYQHVFKTPT
>Pontella_meadi|GFP1|gi|33243031|gb|AY268074.1|
-MPDMKLECHISGTMNGEEFELIGAGDGNTDEGRMTNKMKSIKGPISFSPYLLSHILGYG
YYHFATFPAGYENILHAMKNGGYSNVRTERYEDGGIISITFNYRYEGNKIIGDFKVVGTG
FPTNSLIFTDKIIKSNPTCENMFPKADNTLVNAYTRTYLLKDGGYYSAQVNNHMHFKSAI
HTLQNGGSMFTYRVVEETHTQNAIVEYQNVFKTPT
>Pontella_meadi|GFP2|gi|33243033|gb|AY268075.1|
-MPDMKLECHISGTMNGEEFELIGSGDGNTDQGRMTNNMKSIKGPLSFSPYLLSHILGYG
YYHFATFPAGYENILHAMKNGGYSNVRTERYEDGGIISITFNYRYEGSKIIGDFKVIGTG
FPTDSLIFTDKIIKSNPTCENMFPKADNILVNAYTRTYLLKDGGYYSAQVNNHMHFKSAI
HPLQNGGSMFTHRVVEENHTKTAIVEYQNVFKTPT
>Pontellina_plumata|GFP1|gi|33243025|gb|AY268071.1|
-MPAMKIECRISGTLNGVVFELVGGGEGIPEQGRMTNKMKSTKGALTFSPYLLSHVMGYG
FYHFGTYPSGYENPLHAANNGGYTNTRIEKYEDGGVLHVSFSYRYEAGRVIGDFKVVGTG
FPEDSVIFTDKIIRSNATVEHLHPMGDNVLVGSFARTFSLRDGGYYSFVVDSHMHFKSAI

HPLQNGGSMFAFRRVEELHSNTGIVEYQHAFKTPT
>Pontellina_plumata|GFP2|gi|33243027|gb|AY268072.1|
-MPAMKIECRITGTLNGVEFELVGGGEGTPEQGRMTNKMKSTKGALTFSPYLLSHVMGYG
FYHFGTYPSGYENPLHAINNGGYTNTRIEKYEDGGVLHVSFSYRYEAGRVIGDFKVVGTG
FPEDSVIFTDKIIRSNATVEHLHPMGDNVLVGSFARTFSLRDGGYYSFVVDSHMHFKSAI
HPLQNGGPMFAFRRVEELHSNTGIVEYQHAFKTPI
>Pontellidae_sp.SL-2003|GFP|gi|33243035|gb|AY268076.1|
-MAAMKIECRITGTMNGVEFELVGGGEGNTDQGRMTNKMKSTKGPLSFSPYLLSHVMGYG
FYHFGTFPSGYENPVHAMTNGGYTNTRIESYEDGGVLYLTFNYRLDGNKIIGDFKCVGTG
FPEDSVIFTDKIIKSNPNCEHFYPMAENIMKNAYMRTLSLRDGGYYSGQVTSHIHFKNAI
HPLHNGGSMFTYRRVEELHTQTGIVEYQHVFKTPT
>Goniopora_tenuidens|GFP-like_chromoprotein|gi|16660127|gb|AF383156.1|
IAKQMTYKVYMSGTVNGHYFEVQGDGKGKPYEGEQTVKLTVTKGPLPFAWDILSPQSQYG
SIPFTKYPEDIPDYVKQSFPEGYTWERIMNFEDGAVCTVSNDSSIQGNCFIYNVKFSGLN
FPPNGPVMQKKTQGWEPNTERLFARDG-MLIGNNFMALKLEGGGHYLCEFKSTYKAKKPV
---KMPGYHYVDRKLDVTNHNISVEQCEISIARKP
>Condylactis_passiflora|GFP-like_chromoprotein|gi|16660124|gb|AF383155.1|
LKESMRIKIYMEGTVNGYHFKCEGEGDGNPYEGTQNMRIRVTEGPLPFAFDILSPCCAYG
SKTFIKHTSGIPDYFKQSFPEGFTWERTTIYEDGGVLTAHQDTSLEGNCLNYKVKVLGTN
FPADGPVMKNISGGWEPCTEIVYQDNG-VLRGRNVMALKVSGRPPLICHLHSTYRSKKAC
A-LTMPGFHFADLRIQMPKKKKYFELYEASVARYS
>Heteractis_crispa|GFP-like_chromoprotein|gi|16660115|gb|AF363776.1|
LKESMRIKMYMEGTVNGHYFKCEGEGDGNPFTGTQSMRIHVTEGPLPFAFDILAPCCEYG
SRTFVHHTAEIPDFFKQSFPEGFTWERTTTYEDGGILTAHQDTSLEGNCLIYKVKVLGTN
FPADGPVMKNKSGGWEPCTEVVYPENG-VLCGRNVMALKVGDR-RLICHLYTSYRSKKAV
RALTMPGFHFTDIRLQMPRKKKYFELYEASVARYS
>Galaxea_fascicularis|GFP-like_chromoprotein|gi|78059179|gb|DQ206394.1|
IAKQMTYKVYMSGTVNGHYFEVEGDGKGKPYEGEQTVKLTVTKGPLPFAWDILSPQSQYG
SIPFTKYPEDIPDYVKQSFPEGYTWERIMNFEDGAVCTVSNDSSIQGNCFIYHVKFSGLN
FPPNGPVMQKKTQGWEPNTERLFARDG-MLIGNNFMALKLEGGGHYLCEFKSTYKAKKPV
---KMPGYHYVDRKLDVTNHNKSVEQCEISIARKS
>Montipora_efflorescens|GFP-like_chromoprotein|gi|78059145|gb|DQ206377.1|
IAKQMTYKVYMSGTVNGHYFEVEGDGKGKPYEGEQTVKLTVTKGPLPFAWDILSPLSQYG
SIPFTKYPEDIPDYVKQSFPEGYTWERIMNFEDGAVCTVSNDSSIQGNCFIYNVKISGVN
FPPNGPVMQKKTQGWEPNTERLFARDG-MLIGNNFMALKLEGGGYYLCEFKSTYKAKKPV
---RMPGYHYVDRKLDVTSHNKFVEQCEISIARHS

Supplementary file 4: The trimmed protein sequence alignment used in the phylogenetic analysis for Fig. 5 (in FASTA format).

>gi|508123703|pdb|4HE4|A|Phialidium_sp._SL-2003|Cnidarian-Hydrozoa
FHGKIPYVVEMEGNVDGHTFSIRGKGYGDASVGKVDAQFICTTGDVPVPWSTLVTTLXAQ
CFAKYGPELKDFYKSCMPDGYVQERTITFEGDGNFKTRAEVTFENGSVYNRVKLNGQGFK
KDGHVLGKNLNFTPHCLYIWGDQAGLKSAFKICHEITKGDFIVADHTQMNTPIGPVHPEY
HHMSYHVKLSKDVRDNMSLKETVRAVDCRK
>gi|40365351|gb|AAR85349.1||Phialidium_sp._SL-2003|Cnidarian-Hydrozoa
FHGKIPYVVEMEGNVDGHTFSIRGKGYGDASVGKVDAQFICTTGDVPVPWSTLVTTLGAQ
CFAKYGPELKDFYKSCMPEGYVQERTITFEGDGVFKTRAEVTFENGSVYNRVKLNGQGFK
KDGHVLGKNLNFTPHCLYIWGDQAGLKSAFKIMHEITGSKEIVADHTQMNTPIGPVHPEY
HHITYHVTLSKDVRDNMSLVETVRAVDCRK
>gi|347726909|gb|AEP19814.1||Clytia_hemisphaerica|Cnidarian-Hydrozoa
LNQRVPFIMELDAEVNGIRFAVRGKGTGDATTGIIDTKFVCTTGKLPVPWASISSTMGAL
CFAKYPDSVKDFFKSAMPDGYIQEKTISFENDGAYKVRGVITYEHGSIYNRVTLKGEGFK
KDGLILQKQYCCPNSAVYVLPDKEGLRVVYNTIYKLKDGGHHLAAHEQQNTPLGVVDPNY
HHIHAGSIFSKDLRDHMCLVETVRAVNLET
>gi|298257355|gb|ADI71927.1||Clytia_gregaria|Cnidarian-Hydrozoa
FEKEIPYITELEGDVEGMKFIIKGEGTGDATTGTIKAKYICTTGDLPVPWATILSSLGVF
CFAKYPRHIADFFKSTQPDGYSQDRIISFDNDGQYDVKAKVTCENGTLYNRVTVKGTGFK
SNGNILGMRVHSPPHAVYILPDRKGMKIEYNKAFDVMGGGHQMARHAQFNKPLWEEDPLY
HHLTVWTSFGKDPTDHLNIVEVIKAVDLET
>gi|116667779|pdb|2HPW|A|Clytia_gregaria|Cnidarian-Hydrozoa
FEKEIPYITELEGDVEGMKFIIKGEGTGDATTGTIKAKYICTTGDLPVPWATILSSLXVF
CFAKYPRHIADFFKSTQPDGYSQDRIISFDNDGQYDVKAKVTYENGTLYNRVTVKGTGFK
SNGNILGMRVHSPPHAVYILPDRKGMKIEYNKAFDVMGGGHQMARHAQFNKPLWEEDPLY
HHLTVWTSFGKDPTDHLTIVEVIKAVDLET
>gi|298257357|gb|ADI71928.1||Clytia_gregaria|Cnidarian-Hydrozoa
FEKEIPYITELEGDVEGMKFIIKGEGTGDATTGTIKAKYICTTGDLPVPWATILSSLGVF
CFAKYPRHIADFFKSTQPDGYSQDRIISFDNDGQYDVKAKVTYENGTLYNRVTVKGTGFK
SNGNILGMRVHSPPHAVYILPDRKGMKIEYNKAFDVMGGGHQMARHAQFNKPLWEEDPLY
HHLTVWTSFGKDPTDHLTIVEVIKAVDLET
>gi|298257359|gb|ADI71929.1||Clytia_gregaria|Cnidarian-Hydrozoa
FEKEIPYITELEGDVEGMKFIIKGVGTGDATTGTIKAKYICTTGDLPVPWATILSSLGVF
CFAKYPRHIADFFKSTQPDGYSQDRIISFDNDGQYDVKAKVTYENGTLYNRVTVKGTGFK
SNGNILGMRVHSPPHAVYILPDRKGMKIEYNKAFDVMGGGHQMARHAQFNKPLWEEDPLY
HHLTVWTSFGKDPTDHLNIVEVIKAVDLET
>gi|298257367|gb|ADI71933.1||Clytia_gregaria|Cnidarian-Hydrozoa
FEKEIPYITELEGDVEGMKFIIKGEGTGDATTGTIKAKYICTTGDLPVPWATILSSLGVF
CFAKYPRHIADFFKSTQPDGYSQDRIISFDNDGQYDVKAKVTYENGTLYNRVAVKGTGFK
SNGNILGMRVHSPPHAVYILPDRKGMKIEYNKAFDVMGGGHQMARHAQFNKPLWEEDPLY
HHLTVWTSFGKDPTDHLNIVEVIKAVDLET
>gi|298257371|gb|ADI71935.1||Clytia_gregaria|Cnidarian-Hydrozoa
FEKEIPYITELEGDVEGMKFIIKGEGTGDATTGTIKAKYICTTGDLPVPWATILSSLGVF

CFAKYPRHIADFFKSTQPDGYSQDRIISFDNDGQYDVKAKVTYENGTLYNRVTVKGTGFK
SNGNILGMRVHSPPHAVYILPDRKGMKIEYNKAFDVMGGGHQMARHAQFNKPLWEEDPLY
HHLTVWTSFGKDPTDHLNIVEVIKAVDLET
>gi|342221049|gb|AEL17649.1||Obelia_sp._MH-2011|Cnidarian-Hydrozoa
FTNKIPYVTELEGDVNGMKFTIHGKGTGDASTGNIEAKYVCTSGEIPVPWATLVSTLGVQ
CFAKYPSHIKDFFKSAMPEGYTQERTISFEGDGVYKTRAMVTYERGSIYNRVTLTGENFK
KDGHILRKNVQCPPDMVYILPDTVGIRVEFNQAYDIEGVTEKLVTCSQMNRPLSAAVPRY
HHLSKHTKLSKDRRDHMCLVEVVKAVDLDT
>gi|342221051|gb|AEL17650.1||Obelia_sp._MH-2011|Cnidarian-Hydrozoa
FTNKIPYVTELEGDVNGMKFTIHGKGTGDASTGHIEAKYVCTSGEIPVPWATLVSTMGVQ
CFAKYPSHIKDFYKSAMPEGYIQERTISFEGDGVYKTRAMVTYERGSIYNRVTLTGENFK
KDGHILRKNVQCLPSILYILPDTVGIRVEFNQVYDIEGEIEKLVTCSQMNRPLSAAVPRY
HHISKHTKLSKDLRDHMCLVEVVKAVDLDT
>gi|342221053|gb|AEL17651.1||Obelia_sp._MH-2011|Cnidarian-Hydrozoa
FTNKIPYVTELEGDVNGMKFTIHGKGTGDASTGHIEAKYVCTSGEIPVPWATLVSTMGVQ
CFAKYPSHIKDFYKSAMPEGYIQERTISFEGDGVYKTRAMVTYERGSIYNRVTLTGENFK
KDGHILRKNVQCPPSILYILPDTVGIRVEFNQVFDIEGETEKLVSFSQINRPLSAAVPRY
HHITYHTKLSKDRRDHMCLVEVVKAVDLDT
>gi|347726911|gb|AEP19815.1||Clytia_hemisphaerica|Cnidarian-Hydrozoa
FEKEIPYITELEGDVEGMKFTIKGEGTGDGTTGLIKSKYICTTGDLPVPWATILSSIGVF
CFAKYPRHIADFFKSTQPDGYSQDRIISFDDDGQYDVKAKITYEDGTLYNRVVLDGTGFK
SNGNILGMRVHSPPHIIYILPDRKGMKIEYNKAFDVMGGGHQMTRHAQFNKPLWEEDPMY
HHLSVWTSFGKDPTDHLTIVEVIKAIDLDT
>gi|582045456|pdb|4LW5|A|Aequorea_victoria|Cnidarian-Hydrozoa
FTGVVPILVELDGDVNGHKFSVRGEGEGDATIGKLTLKFICTTGKLPVPWPTLVTTLXVQ
CFSRYPDHMHDFFKSAISGGFINERTISFKDDGKYKTRAVVKFEGDTLVNRIELKGTDFK
EDGNILGHKLNFNSHNVYITADKQGIKANFTVRHNVEDGSVQLADHYQQNTPIGPVLPDN
HYLSTQTVLSKDPRDHMVLLEFVTAAGITH
>gi|122920877|pdb|2JAD|A|Aequorea_victoria|Cnidarian-Hydrozoa
FTGVVPILVELDGDVNGHKFSVSGEGEGDATYGKLTLKFIVTTGKLPVPWPTLVTTFXLQ
CFARYPDHMHDFFKSAMPEGYVQERTIFFKDDGNYKTRAEVKFEGDTLVNRIELKGIDFK
EDGNILGHKLNYNSHCVYIVADKQGIKVNFKIRHNIEDGSVQLADHYQQNTPIGPVLPDN
HYLCYQSALSKDPRDHMVLLEFVTAAGITH
>gi|221046699|pdb|3ED8|A|Aequorea_victoria|Cnidarian-Hydrozoa
FTGVVPILVELDGDVNGHKFSVRGEGEGDATIGKLTLKFICTTGKLPVPWPTLVTTLXLQ
CFARYPDHMHDFFKSAMPEGYVQERTISFKDDGKYKTRAVVKFEGDTLVNRIELKGTDFK
EDGNILGHKLNFNSHNVYIVMNHDGIKANFTVRHNVEDGSVQLADHYQQNTPIGPVLPDN
HYLSYQTVLSKDPRDHMVLLEFVTAAGITL
>gi|353442081|gb|AER00326.1||Aequorea_victoria|Cnidarian-Hydrozoa
----

------------------------GIKVNFKIRHNIEDGSVQLADHYQQNTPIGPVLPDN
HYLS--------------------------
>gi|402550124|pdb|3U8P|A|Aequorea_victoria|Cnidarian-Hydrozoa
FTGVVPILVELDGDVNGHKFSVSGEGEGDATFGKLTLKFICTTGKLPVPWPTLVTTLXVQ
CFSRYPDHMHDFFKSAMPEGYVQERTIFFKDDGNYKTRAEVKFEGDTLVNRIELKGIDFK

EDGNILGHKLNYNSHNVYIMADKQGIKVNFKIRHNIEDGSVQLADHYQQNTPIGPVLPDN
HYLSTQSALSKDPRDHMVLLEFVTAAGITL
>gi|499142128|pdb|4J8A|A|Aequorea_victoria|Cnidarian-Hydrozoa
FTGVVPILVELDGDVNGHKFSVRGEGEGDATNGKLTLKFICTTGKLPVPWPTLVTTLXVQ
CFSRYPDHMHDFFKSAMPEGYVQERTISFKDDGTYKTRAEVKFEGDTLVNRIELKGIDFK
EDGNILGHKLNXNSHNVYITADKQGIKANFKIRHNVEDGSVQLADHYQQNTPIGPVLPDN
HYLSTQSVLSKDPRDHMVLLEFVTAAGITH
>gi|514829887|pdb|4J88|A|Aequorea_victoria|Cnidarian-Hydrozoa
FTGVVPILVELDGDVNGHKFSVRGEGEGDATNGKLTLKFICTTGKLPVPWPTLVTTLXVQ
CFSRYPDHMHDFFKSAMPEGYVQERTISFKDDGTYKTRAEVKFEGDTLVNRIELKGIDFK
EDGNILGHKLNFNSHNVYITADKQGIKANFKIRHNVEDGSVQLADHYQQNTPIGPVLPDN
HYLSTQSVLSKDPRDHMVLLEFVTAAGITH
>gi|550545192|pdb|4JFG|A|Aequorea_victoria|Cnidarian-Hydrozoa
FTGVVPILVELDGDVNGHKFSVRGEGEGDATNGKLTLKFICTTGKLPVPWPTLVTTLXVQ
CFSRYPDHMHDFFKSAMPEGYVQERTISFKDDGTYKTRAEVKFEGDTLVNRIELKGIDFK
EDGNILGHKLNFNSHNVYITADKQGIKANFKIRHNVEDGSVQLADHYQQNTPIGPVLPDN
HYLSTQSVLSKDPRDHMVLLEFVTAAGITH
>gi|83754213|pdb|2B3P|A|Aequorea_victoria|Cnidarian-Hydrozoa
FTGVVPILVELDGDVNGHKFSVRGEGEGDATNGKLTLKFICTTGKLPVPWPTLVTTLXVQ
CFSRYPDHMHDFFKSAMPEGYVQERTISFKDDGTYKTRAEVKFEGDTLVNRIELKGIDFK
EDGNILGHKLNFNSHNVYITADKQGIKANFKIRHNVEDGSVQLADHYQQNTPIGPVLPDN
HYLSTQSVLSKDPRDHMVLLEFVTAAGITH
>gi|83754214|pdb|2B3Q|A|Aequorea_victoria|Cnidarian-Hydrozoa
FTGVVPILVELDGDVNGHKFSVSGEGEGDATYGKLTLKFICTTGKLPVPWPTLVTTLXVQ
CFSRYPDHMHDFFKSAMPEGYVQERTISFKDDGNYKTRAEVKFEGDTLVNRIELKGIDFK
EDGNILGHKLNYNSHNVYITADKQGIKANFKIRHNIEDGSVQLADHYQQNTPIGPVLPDN
HYLSTQSALSKDPRDHMVLLEFVTAAGITH
>gi|506954255|gb|AGM20710.1||Aequorea_victoria|Cnidarian-Hydrozoa
FTGVVPILVELDGDVNGHKFSVSGEGEGDATNGKLTLKFICTTGKLPVPWPTLVTTLGVQ
CFARYPDHMHDFFKSAMPEGYVQERTISFKDDGTYKTRAEVKFEGDTLVNRIELKGIDFK
EDGNILGHKLNFNSHNVYITADKQGIKANFKIRHNVEDGSVQLADHYQQNTPIGPVLPDN
HYLSTQSALSKDPRDHMVLLEFVTAAGITH
>gi|506954257|gb|AGM20711.1||Aequorea_victoria|Cnidarian-Hydrozoa
FTGVVPILVELDGDVNGHKFSVSGEGEGDATNGKLTLKFICTTGKLPVPWPTLVTTLGVQ
CFARYPDHMHDFFKSAMPEGYVQERTISFKDDGTYKTRAEVKFEGDTLVNRIELKGIDFK
EDGNILGHKLNFNSHNVYITADKQGIKANFKIRHNVEDGSVQLADHYQQNTPIGPVLPDN
HYLSYQSALSKDPRDHMVLLEFVTAAGITH
>gi|506954259|gb|AGM20712.1||Aequorea_victoria|Cnidarian-Hydrozoa
FTGVVPILVELDGDVNGHKFSVSGEGEGDATNGKLTLKFICTTGKLPVPWPTLVTTFGVQ
CFARYPDHMHDFFKSAMPEGYVQERTISFKDDGTYKTRAEVKFEGDTLVNRIELKGIDFK
EDGNILGHKLNFNSHNVYITADKQGIKANFKIRHNVEDGSVQLADHYQQNTPIGPVLPDN
HYLSTQSALSKDPRDHMVLLEFVTAAGITH
>gi|567755457|pdb|4LQT|A|Aequorea_victoria|Cnidarian-Hydrozoa
FTGVVPILVELDGDVNGHKFSVRGEGEGDATNGKLTLKFICTTGKLPVPAPTLVTTLXVQ
CFSRYPDHMHDFFKSAMPEGYVQERTISFKDDGTYKTRAEVKFEGDTLVNRIELKGIDFK
EDGNILGHKLNFNSHNVYITADKQGIKANFKIRHNVEDGSVQLADHYQQNTPIGPVLPDN

HYLSTQSALSKDPRDHMVLLEFVTAAGITH
>gi|567755459|pdb|4LQU|A|Aequorea_victoria|Cnidarian-Hydrozoa
FTGVVPILVELDGDVNGHKFSVRGEGEGDATNGKLTLKFICTTGKLPVPGPTLVTTLXVQ
CFSRYPDHMHDFFKSAMPEGYVQERTISFKDDGTYKTRAEVKFEGDTLVNRIELKGIDFK
EDGNILGHKLNFNSHNVYITADKQGIKANFKIRHNVEDGSVQLADHYQQNTPIGPVLPDN
HYLSTQSALSKDPRDHMVLLEFVTAAGITH
>gi|634009|emb|CAA58789.1||Aequorea_victoria|Cnidarian-Hydrozoa
FTGVVPVLVELDGDVNGQKFSVSGEGEGDATYGKLTLNFICTTGKLPVPWPTLVTTFGVQ
CFSRYPDHMHDFFKSAMPEGYVQERTIFYKDDGNYKTRAEVKFEGDTLVNRIELKGIDFK
EDGNILGHKMNYNSHNVYIMGDKPGIKVNFKIRHNIKDGSVQLADHYQQNTPIGPVLPDN
HYLSTQSALSKDPRDHMILLEFVTAARITH
>gi|634011|emb|CAA58790.1||Aequorea_victoria|Cnidarian-Hydrozoa
FTGVVPILVELDGDVNGQKFSVRGEGEGDATYGKLTLKFICTTGKLPVPWPTLVTTFGVQ
CFSRYPDHMHDFLKSAMPEGYVQERTIFYKDDGNYKTRAEVKFEGDTLVNRIELKGIDFK
EDGNILGHKMNYNSHNVYIMGDKPGIKVNFKIRHNIKDGSVQLADHYQQNTPIGPVLPDN
HYLSTQSALSQDPRDHMVLLEFVTSAGITH
>gi|741846|prf||2008181A|Aequorea_victoria|Cnidarian-Hydrozoa
FTGVVPILVELDGDVNGQKFSVSGEGEGDATYGKLTLKFICTTGKLPVPWPTLVTTFGVQ
CFSRYPDHMHDFFKSAMPEGYVQERTIFFKDDGNYKTRAEVKFEGDTLVNRIELKGIDFK
EDGNILGHKMNYNSHNVYIMADKPGIKVNFKIRHNIKDGSVQLADHYQQNTPIGPVLPDN
HYLSTQSALSKDPRDHMILLEFVTAAGITH
>gi|704360445|pdb|4NDJ|A|Aequorea_victoria|Cnidarian-Hydrozoa
FTGVVPILVELDGDVNGHKFSVSGEGEGDATYGKLTLKLICTTGKLPVPWPTLVTTLXLQ
CFARYPDHMHDFFKSAMPEGYVQERTIFFKDDGNYKTRAEVKFEGDTLVNRIELKGIDFK
EDGNILGHKLNYNSHNVYITADKQGIKANFKIRHNIEDGGVQLADHYQQNTPIGPVLPDN
HYLSYQSKLSKDPRDHMVLLEFVTAAGITL
>gi|704360450|pdb|4NDK|A|Aequorea_victoria|Cnidarian-Hydrozoa
FTGVVPILVELDGDVNGHKFSVSGEGEGDATYGKLTLKLICTTGKLPVPFPTLVTTLXLQ
CFARYPDHMHDFFKSAMPEGYVQERTIFFKDDGNYKTRAEVKFEGDTLVNRIELKGIDFK
EDGNILGHKLNYNSHNVYITADKQGIKANFKIRHNIEDGGVQLADHYQQNTPIGPVLPDN
HYLSYQSKLSKDPRDHMVLLEFVTAAGITL
>gi|756142478|pdb|4ORN|A|Aequorea_victoria|Cnidarian-Hydrozoa
FTGVVPILVEMDGDVNGRKFSVRGVGEGDATHGKLTLKFICTSGKLPVPWPTLVTTLXVQ
XFSRYPDHMHDFFKSAMPEGYVQERTIFFKDDGSYKTRAEVKFEGDTLVNRIVLKGTDFK
EDGNILGHKLNMNVGNVYITADKQGIKANFEIRHNVEDGGVQLADHYQQNTPIGSVLPDN
HYLSVQVKLSKDPRDHMVLLEFRTAAGITP
>gi|99032371|pdb|2G2S|B|Aequorea_victoria|Cnidarian-Hydrozoa
---GVQ
CFSRYPDHMHDFFKSAMPEGYVQERTISFKDDGNYKTRAEVKFEGDTLVNRIELKGIDFK
EDGNILGHKLNYNSHNVYITADKQGIKANFKIRHNIEDGSVQLADHYQQNTPIGPVLPDN
HYLSTQSALSKDPRDHMVLLEFVTAAGITH
>gi|99032352|pdb|2G16|B|Aequorea_victoria|Cnidarian-Hydrozoa
---XVQ
CFSRYPDHMHDFFKSAMPEGYVQERTISFKDDGNYKTRAEVKFEGDTLVNRIELKGIDFK
EDGNILGHKLNYNSHNVYITADKQGIKANFKIRHNIEDGSVQLADHYQQNTPIGPVLPDN
HYLSTQSALSKDPRDHMVLLEFVTAAGITH

>gi|99032375|pdb|2G3D|B|Aequorea_victoria|Cnidarian-Hydrozoa
---GVQ
CFSRYPDHMHDFFKSAMPEGYVQERTISFKDDGNYKTRAEVKFEGDTLVNRIELKGIDFK
EDGNILGHKLNYNSHNVYITADKQGIKANFKIRHNIEDGSVQLADHYQQNTPIGPVLPDN
HYLSTQSALSKDPRDHMVLLEFVTAAGITH
>gi|150261557|pdb|2Q6P|A|Aequorea_victoria|Cnidarian-Hydrozoa
FTGVVXILVELDGDVNGHKFSVSGEGEGDATYGKLTLKFICTTGKLXVXWXTLVTTLXVQ
CFSRYXDHMHDFFKSAMXEGYVQERTIFFKDDGNYKTRAEVKFEGDTLVNRIELKGIDFK
EDGNILGHKLNYNSHNVYIMADKQGIKVNFKIRHNIEDGSVQLADHYQQNTXIGXVLXDN
HYLSTQSALSKDXRDHMVLLEFVTAAGITH
>gi|2392316|pdb|1EMB||Aequorea_victoria|Cnidarian-Hydrozoa
FTGVVPILVELDGDVNGHKFSVSGEGEGDATYGKLTLKFICTTGKLPVPWPTLVTTFXVQ
CFSRYPDHMHDFFKSAMPEGYVQERTIFFKDDGNYKTRAEVKFEGDTLVNRIELKGIDFK
EDGNILGHKLNYNSHNVYIMADKQGIKVNFKIRHNIEDGSVQLADHYQQNTPIGPVLPDN
HYLSTQSALSKDPRDHMVLLEFVTAAGITH
>gi|48425578|pdb|1S6Z|A|Aequorea_victoria|Cnidarian-Hydrozoa
FTGVVPILVELDGDVNGHKFSVSGEGEGDATYGKLTLKFICTTGKLPVPWPTLVTTLXVQ
CFSRYPDHMHDFFKSAMPEGYVQERTIFFKDDGNYKTRAEVKFEGDTLVNRIELKGIDFK
EDGNILGHKLNYNSHNVYIMADKQGIKVNFKIRHNIEDGSVQLADHYQQNTPIGPVLPDN
HYLSTQSALSKDPRDHMVLLEFVTAAGITH
>gi|149241869|pdb|2H6V|A|Aequorea_victoria|Cnidarian-Hydrozoa
FTGVVPILVELDGDVNGHKFSVSGEGEGDATYGKLTLKFICTTGKLPVPWPTLVTTLXVQ
CFSRYPDHMHDFFKSAMPEGYVQERTIFFKDDGNYKTRAEVKFEGDTLVNRIELKGIDFK
EDGNILGHKLNYNSHNVYIMADKQGIKVNFKIRHNIEDGSVQLADHYQQNTPIGPVLPDN
HYLSYQSALSKDPRDHMVLLEFVTAAGITL
>gi|11513672|pdb|1F09|A|Aequorea_victoria|Cnidarian-Hydrozoa
FTGVVPILVELDGDVNGHKFSVSGEGEGDATYGKLTLKFICTTGKLPVPWPTLVTTFXLQ
CFARYPDHMHDFFKSAMPEGYVQERTIFFKDDGNYKTRAEVKFEGDTLVNRIELKGIDFK
EDGNILGHKLNYNSQNVYIMADKQGIKVNFKIRHNIEDGSVQLADHYQQNTPIGPVLPDN
HYLSYQSALSKDPRDHMVLLEFVTAAGITH
>gi|1169893|sp|P42212.1|GFP_AEQVI|Aequorea_victoria|Cnidarian-Hydrozoa
FTGVVPILVELDGDVNGHKFSVSGEGEGDATYGKLTLKFICTTGKLPVPWPTLVTTFGVQ
CFSRYPDHMHDFFKSAMPEGYVQERTIFFKDDGNYKTRAEVKFEGDTLVNRIELKGIDFK
EDGNILGHKLNYNSHNVYIMADKQGIKVNFKIRHNIEDGSVQLADHYQQNTPIGPVLPDN
HYLSTQSALSKDPRDHMVLLEFVTAAGITH
>gi|145579497|pdb|2HFC|A|Aequorea_victoria|Cnidarian-Hydrozoa
FTGVVPILVELDGDVNGHKFSVSGEGEGDATYGKLTLKFICTTGKLPVPWPTLVTTLGVQ
CFSRYPDHMHDFFKSAMPEGYVQEATISFKDDGNYKTRAEVKFEGDTLVNRIELKGIDFK
EDGNILGHKLNYNSHNVYITADKQGIKANFKIRHNIEDGSVQLADHYQQNTPIGPVLPDN
HYLSTQSALSKDPRDHMVLLEFVTAAGITH
>gi|146386837|pdb|2H9W|A|Aequorea_victoria|Cnidarian-Hydrozoa
FTGVVPILVELDGDVNGHKFSVSGEGEGDATYGKLTLKFICTTGKLPVPWPTLVTTLXVQ
XFSRYPDHMHDFFKSAMPEGYVQERTIFFKDDGNYKTRAEVKFEGDTLVNRIELKGIDFK
EDGNILGHKLNYNSHNVYIMADKQGIKVNFKIRHNIEDGSVQLADHYQQNTPIGPVLPDN
HYLSYQSALSKDPRDHMVLLEFVTAAGITL
>gi|14719453|pdb|1HUY|A|Aequorea_victoria|Cnidarian-Hydrozoa

FTGVVPILVELDGDVNGHKFSVSGEGEGDATYGKLTLKFICTTGKLPVPWPTLVTTFXLM
CFARYPDHMHDFFKSAMPEGYVQERTIFFKDDGNYKTRAEVKFEGDTLVNRIELKGIDFK
EDGNILGHKLNYNSHNVYIMADKQGIKVNFKIRHNIEDGSVQLADHYQQNTPIGPVLPDN
HYLSYQSALSKDPRDHMVLLEFVTAAGITH
>gi|155663|gb|AAA27722.1||Aequorea_victoria|Cnidarian-Hydrozoa
FTGVVPILVELDGDVNGHKFSVSGEGEGDATYGKLTLKFICTTGKLPVPWPTLVTTFGVQ
CFSRYPDHMHDFFKSAMPEGYVQERTIFYKDDGNYKSRAEVKFEGDTLVNRIELKGIDFK
EDGNILGHKMNYNSHNVYIMADKQGIKVNFKIRHNIEDGSVQLADHYQQNTPIGPVLPDN
HYLSTQSALSKDPRDHMILLEFVTAAGITH
>gi|1942689|pdb|1GFL|A|Aequorea_victoria|Cnidarian-Hydrozoa
FTGVVPILVELDGDVNGHKFSVSGEGEGDATYGKLTLKFICTTGKLPVPWPTLVTTFGVQ
CFSRYPDHMHDFFKSAMPEGYVQERTIFFKDDGNYKTRAEVKFEGDTLVNRIELKGIDFK
EDGNILGHKLNYNSHNVYIMADKQGIKVNFKIRHNIEDGSVQLADHYQQNTPIGPVLPDN
HYLSTQSALSKDPRDHMVLLEFVTAAGITH
>gi|206582017|pdb|3DPW|A|Aequorea_victoria|Cnidarian-Hydrozoa
FTGVVPILVELDGDVNGHKFSVSGEGEGDATYGKLTLKFICTTGKLPVPWPTLVTTFXLM
CFARYPDHMHDFFKSAMPEGYVQERTIFFKDDGNYKTRAEVKFEGDTLVNRIELKGIDFK
EDGNILGHKLNYNSHNVYIMADKQGIKVNFKIRHNIEDGSVQLADHYQQNTPIGPVLPDN
HYLSYQSALSKDPRDHMVLLEFVTAAGITL
>gi|253723015|pdb|1MYW|A|Aequorea_victoria|Cnidarian-Hydrozoa
FTGVVPILVELDGDVNGHKFSVSGEGEGDATYGKLTLKLICTTGKLPVPWPTLVTTLXLQ
CFARYPDHMHDFFKSAMPEGYVQERTIFFKDDGNYKTRAEVKFEGDTLVNRIELKGIDFK
EDGNILGHKLNYNSHNVYITADKQGIKANFKIRHNIEDGGVQLADHYQQNTPIGPVLPDN
HYLSYQSALSKDPRDHMVLLEFVTAAGITH
>gi|28373440|pdb|1JBY|A|Aequorea_victoria|Cnidarian-Hydrozoa
FTGVVPILVELDGDVNGHKFSVSGEGEGDATYGKLTLKFICTTGKLPVPWPTLVTTFXVQ
CFSRYPDHMHDFFKSAMPEGYVQERTIFFKDDGNYKTRAEVKFEGDTLVNRIELKGIDFK
EDGNILGHKLNYNSGNVYIMADKQGIKVNFKIRHNIEDGSVQLADHYQQNTPIGPVLPDN
HYLSCQSALSKDPRDHMVLLEFVTAAGITH
>gi|34809574|pdb|1JC0|A|Aequorea_victoria|Cnidarian-Hydrozoa
FTGVVPILVELDGDVNGHKFSVSGEGEGDATYGKLTLKFISTTGKLPVPWPTLVTTLXVQ
CFSRYPDHMHDFFKSAMPEGYVQERTIFFKDDGNYKTRAEVKFEGDTLVNRIELKGIDFK
EDGNILGHKLNYNCHNVYIMADKQGIKVNFKIRHNIEDGSVQLADHYQQNTPIGPVLPDN
HYLSTCSALSKDPRDHMVLLEFVTAAGITH
>gi|34811710|pdb|1H6R|A|Aequorea_victoria|Cnidarian-Hydrozoa
FTGVVPILVELDGDVNGHKFSVSGEGEGDATYGKLTLKFIVTTGKLPVPWPTLVTTFXLQ
CFARYPDHMHDFFKSAMPEGYVQERTIFFKDDGNYKTRAEVKFEGDTLVNRIELKGIDFK
EDGNILGHKLNYNSHCVYIVADKQGIKVNFKIRHNIEDGSVQLADHYQQNTPIGPVLPDN
HYLCYQSALSKDPRDHMVLLEFVTAAGITH
>gi|42543412|pdb|1Q73|A|Aequorea_victoria|Cnidarian-Hydrozoa
FTGVVPILVELDGDVNGHKFSVSGEGEGDATYGKLTLKFICTTGKLPVPWPTLVTTFXVQ
CFSRYPDHMHDFFKSAMPEGYVQERTIFFKDDGNYKTRAEVKFEGDTLVNRIELKGIDFK
EDGNILGHKLNCNSHNVYIMADKQGIKVNFKIRHNIEDGSVQLADHYQQNTPIGPVLPDN
HYLSCQSALSKDPRDHMVLLEFVTAAGITH
>gi|550545392|pdb|3W1D|A|Aequorea_victoria|Cnidarian-Hydrozoa
FTGVVPILVELDGDVNGHKFSVSGEGEGDATYGKLTLKFICTTGKLPVPWPTLVTTFXLQ

CFARYPDHMHDFFKSAMPEGYVQERTIFFKDDGNYKTRAEVKFEGDTLVNRIELKGIDFK
EDGNILGHKLNYNSHNVYIMADKQGIKVNFKIRHNIEDGSVQLADHYQQNTPIGPVLPDN
HYLSYQSALSKDPRDHMVLLEFVTAAGITL
>gi|635575543|pdb|4OGS|A|Aequorea_victoria|Cnidarian-Hydrozoa
FTGVVPILVELDGDVNGHKFSVSGEGEGDATYGKLTLKFICTTGKLPVPWPTLVTTFXVQ
CFSRYPDHMHDFFKSAMPEGYVQERTIFFKDDGNYKTRAEVKFEGDTLVNRIELKGIDFK
EDGNILGHKLNYNSHNVYIMADKQGIKVNFKIRHNIEDGSVQLADHYQQNTPIGPVLPDN
HYLSVQAALSKDPRDHMVLLEFVTAAGITH
>gi|635575819|pdb|4KA9|A|Aequorea_victoria|Cnidarian-Hydrozoa
FTGVVPILVELDGDVNGHKFSVSGEGEGDATYGKLTLKFICTTGKLPVPWPTLVTTLXVQ
CFSRYPDHMHDFFKSAMPEGYVQERTIFFKDDGNYKTRAEVKFEGDTLVNRIELKGIDFK
EDGNILGHKLNYNSHNVYIMADKQGIKVNFKIRHNIEDGSVQLADHYQQNTPIGPVLPDN
HYLSTQSALSKDPRDHMVLLEFVTAAGITL
>gi|6730026|pdb|2YFP|A|Aequorea_victoria|Cnidarian-Hydrozoa
FTGVVPILVELDGDVNGHKFSVSGEGEGDATYGKLTLKFICTTGKLPVPWPTLVTTFXLQ
CFARYPDHMHDFFKSAMPEGYVQERTIFFKDDGNYKTRAEVKFEGDTLVNRIELKGIDFK
EDGNILGHKLNYNSGNVYIMADKQGIKVNFKIRHNIEDGSVQLADHYQQNTPIGPVLPDN
HYLSYQSALSKDPRDHMVLLEFVTAAGITH
>gi|93278673|pdb|2AH8|A|Aequorea_victoria|Cnidarian-Hydrozoa
FTGVVPILVELDGDVNGHKFSVSGEGEGDATYGKLTLKFISTTGKLPVPWPTLVTTFXVQ
CFSRYPDHMHDFFKSAMPEGYVQERTIFFKDDGNYKTRAEVKFEGDTLVNRIELKGIDFK
EDGNILGHKLNYNCHNVYIMADKQGIKVNFKIRHNIEDGSVQLADHYQQNTPIGPVLPDN
HYLKTCSALSKDPRDHMVLLERVTAAGITH
>gi|93278674|pdb|2AH8|B|Aequorea_victoria|Cnidarian-Hydrozoa
FTGVVPILVELDGDVNGHKFSVSGEGEGDATYGKLTLKFISTTGKLPVPWPTLVTTFXVQ
CFSRYPDHMHDFFKSAMPEGYVQERTIFFKDDGNYKTRAEVKFEGDTLVNRIELKGIDFK
EDGNILGHKLNYNCHNVYIMADKQGIKVNFKIRHNIEDGSVQLADHYQQNTPIGPVLPDN
HYLKTCSALSKDPRDHMVLLERVTAAGITH
>gi|93278675|pdb|2AHA|A|Aequorea_victoria|Cnidarian-Hydrozoa
FTGVVPILVELDGDVNGHKFSVSGEGEGDATYGDLTLKFISTTGKLPVPWPTLVTTFXVQ
CFSRYPDHMHDFFKSAMPEGYVQERTIFFKDDGNYKTRAEVKFEGDTLVNRIELKGIDFK
EDGNILGHKLNYNCHNVYIMADKQGIKVNFKIRHNIEDGSVQLADHYQQNTPIGPVLPDN
HYLSTCSALSKDPRDHMVLLERVTAAGITH
>gi|158430429|pdb|2QU1|A|Aequorea_victoria|Cnidarian-Hydrozoa
FTGVVPILVELDGDVNGHKFSVSGEGEGDATYGKLTLKFICTTGKLPVPWPTLVTTLXVQ
CFSRYPDHXHDFFKSAXPEGYVQERTISFKDDGNYKTRAEVKFEGDTLVNRIELKGIDFK
EDGNILGHKLNYNSHNVYITADKQGIKANFKIRHNIEDGSVQLADHYQQNTPIGPVLPDN
HYLSTQSALSKDPRDHXVLLEFVTAAGITH
>gi|11513641|pdb|1B9C|A|Aequorea_victoria|Cnidarian-Hydrozoa
FTGVVPILVELDGDVNGHKFSVSGEGEGDATYGKLTLKFICTTGKLPVPWPTLVTTFXVQ
CFSRYPDHMHDFFKSAMPEGYVQERTISFKDDGNYKTRAEVKFEGDTLVNRIELKGIDFK
EDGNILGHKLNYNSHNVYITADKQGIKANFKIRHNIEDGSVQLADHYQQNTPIGPVLPDN
HYLSTQSALSKDPRDHMVLLEFVTAAGITH
>gi|145579505|pdb|2HGY|A|Aequorea_victoria|Cnidarian-Hydrozoa
FTGVVPILVELDGDVNGHKFSVSGEGEGDATYGKLTLKFICTTGKLPVPWPTLVTTFXVQ
CFSRYPDHMHDFFKSAMPEGYVQERTISFKDDGNYKTRAEVKFEGDTLVNRIELKGIDFK

EDGNILGHKLNYNSHNVYITADKQGIKANFKIRHNIEDGSVQLADHYQQNTPIGPVLPDN
HYLSTQSALSKDPRDHMVLLAFVTAAGITH
>gi|152148991|pdb|2DUE|A|Aequorea_victoria|Cnidarian-Hydrozoa
FTGVVPILVELDGDVNGHKFSVSGEGEGDATYGKLTLKFICTTGKLPVPWPTLVTTFXVQ
CFSRYPDHMHDFFKSAMPEGYVQERTIFFKDDGNYKTRAEVKFEGDTLVNRIELKGIDFK
EDGNILGHKLNYNSDNVYIMADKQGIKVNFKIRHNIEDGSVQLADHYQQNTPIGPVLPDN
HYLSTQSALSKDPRDHMVLLEFVTAAGITH
>gi|152148993|pdb|2DUG|A|Aequorea_victoria|Cnidarian-Hydrozoa
FTGVVPILVELDGDVNGHKFSVSGEGEGDATYGKLTLKFICTTGKLPVPWPTLVTTFXVQ
CFSRYPDHMHDFFKSAMPEGYVQERTIFFKDDGNYKTRAEVKFEGDTLVNRIELKGIDFK
EDGNILGHKLNYNSNNVYIMADKQGIKVNFKIRHNIEDGSVQLADHYQQNTPIGPVLPDN
HYLSTQSALSKDPRDHMVLLEFVTAAGITH
>gi|157830280|pdb|1BFP|A|Aequorea_victoria|Cnidarian-Hydrozoa
FTGVVPILVELDGDVNGHKFSVSGEGEGDATYGKLTLKFICTTGKLPVPWPTLVTTFXVQ
CFSRYPDHMHDFFKSAMPEGYVQERTIFFKDDGNYKTRAEVKFEGDTLVNRIELKGIDFK
EDGNILGHKLNFNSHNVYIMADKQGIKVNFKIRHNIEDGSVQLADHYQQNTPIGPVLPDN
HYLSTQSALSKDPRDHMVLLEFVTAAGITH
>gi|157830960|pdb|1EMA|A|Aequorea_victoria|Cnidarian-Hydrozoa
FTGVVPILVELDGDVNGHKFSVSGEGEGDATYGKLTLKFICTTGKLPVPWPTLVTTFXVQ
CFSRYPDHXHDFFKSAXPEGYVQERTIFFKDDGNYKTRAEVKFEGDTLVNRIELKGIDFK
EDGNILGHKLNYNSHNVYIXADKQGIKVNFKIRHNIEDGSVQLADHYQQNTPIGPVLPDN
HYLSTQSALSKDPRDHXVLLEFVTAAGITH
>gi|157830964|pdb|1EMF|A|Aequorea_victoria|Cnidarian-Hydrozoa
FTGVVPILVELDGDVNGHKFSVSGEGEGDATYGKLTLKFICTTGKLPVPWPTLVTTLXVQ
CFSRYPDHMHDFFKSAMPEGYVQERTIFFKDDGNYKTRAEVKFEGDTLVNRIELKGIDFK
EDGNILGHKLNYNSHNVYIMADKQGIKANFKIRHNIEDGSVQLADHYQQNTPIGPVLPDN
HYLSTQSALSKDPRDHMVLLEFVTAAGITH
>gi|157879831|pdb|1QYO|A|Aequorea_victoria|Cnidarian-Hydrozoa
FTGVVPILVELDGDVNGHKFSVSGEGEGDATYGKLTLKFICTTGKLPVPWPTLVTTLGVQ
CFSRYPDHMHDFFKSAMPEGYVQERTISFKDDGNYKTRAEVKFEGDTLVNRIELKGIDFK
EDGNILGHKLNYNSHNVYITADKQGIKANFKIRHNIEDGSVQLADHYQQNTPIGPVLPDN
HYLSTQSALSKDPRDHMVLLEFVTAAGITH
>gi|178847038|pdb|2HRS|A|Aequorea_victoria|Cnidarian-Hydrozoa
FTGVVPILVELDGDVNGHKFSVSGEGEGDATYGKHTLKFICTTGKLPVPWPTLVTTLXVQ
CFSRYPDHMHDFFKSAMPEGYVQERTISFKDDGNYKTRAEVKFEGDTLVNRIELKGIDFK
EDGNILGHKLNYNSHNVYITADKQGIKANFKIRHNIEDGSVQLADHYQQNTPIGPVLPDN
HYLSTQSALSKDPRDHMVLLEFHTAAGITH
>gi|20664224|pdb|1KYP|A|Aequorea_victoria|Cnidarian-Hydrozoa
FTGVVPILVELDGDVNGHKFSVSGEGEGDATYGKLTLKFICTTGKLPVPWPTLVTTLXVQ
CFSRYPDHMHDFFKSAMPEGYVQERTISFKDDGNYKTRAEVKFEGDTLVNRIELKGIDFK
EDGNILGHKLNFNSGNVYITADKQGIKANFKIRHNIEDGSVQLADHYQQNTPIGPVLPDN
HYLSTQSALSKDPRDHMVLLEFVTAAGITH
>gi|227343654|pdb|2QT2|A|Aequorea_victoria|Cnidarian-Hydrozoa
FTGVVPILVELDGDVNGHKFSVSGEGEGDATYGKLTLKFICTTGKLPVPWPTLVTTLXVQ
CFSRYPDHMHDFFKSAMPEGYVQERTISFKDDGNYKTRAEVKFEGDTLVNRIELKGIDFK
EDGNILGHKLNYNSHNVYITADKQGIKANFKIRHNIEDGSVQLADHYEQNTPIGPVLPDN

HYLSTQSALSKDPRDHMVLLEFVTAAGITH
>gi|270346500|pdb|3G9A|A|Aequorea_victoria|Cnidarian-Hydrozoa
FTGVVPILVELDGDVNGHKFSVSGEGEGDATYGKLTLKFICTTGKLPVPWPTLVTTFXVQ
CFSRYPDHMHDFFKSAMPEGYVQERTISFKDDGNYKTRAEVKFEGDTLVNRIELKGIDFK
EDGNILGHKLNYNSHNVYITADKQGIKANFKIRHNIEDGSVQLADHYQQNTPIGPVLPDN
HYLSTQSALSKDPRDHMVLLEFVTAAGITH
>gi|270346695|pdb|3K1K|A|Aequorea_victoria|Cnidarian-Hydrozoa
FTGVVPILVELDGDVNGHKFSVSGEGEGDATYGKLTLKFICTTGKLPVPWPTLVTTFXVQ
CFSRYPDHXHDFFKSAXPEGYVQERTISFKDDGNYKTRAEVKFEGDTLVNRIELKGIDFK
EDGNILGHKLNYNSHNVYITADKQGIKANFKIRHNIEDGSVQLADHYQQNTPIGPVLPDN
HYLSTQSALSKDPRDHXVLLEFVTAAGITH
>gi|42543379|pdb|1Q4C|A|Aequorea_victoria|Cnidarian-Hydrozoa
FTGVVPILVELDGDVNGHKFSVSGEGEGDATYGKLTLKFICTTGKLPVPWPTLVTTFXVQ
CFSRYPDHMHDFFKSAMPEGYVQERTIFFKDDGNYKTRAEVKFEGDTLVNRIELKGIDFK
EDGNILGHKLNYNSHNVYIMADKQGIKVNFKIRHNIEDGSVQLADHYQQNTPIGPVLPDN
HYLSCQSALSKDPRDHMVLLEFVTAAGITH
>gi|42543381|pdb|1Q4E|A|Aequorea_victoria|Cnidarian-Hydrozoa
FTGVVPILVELDGDVNGHKFSVSGEGEGDATYGKLTLKFICTTGKLPVPWPTLVTTFXVQ
CFSRYPDHMHDFFKSAMPEGYVQERTIFFKDDGNYKTRAEVKFEGDTLVNRIELKGIDFK
EDGNILGHKLNCNSHNVYIMADKQGIKVNFKIRHNIEDGSVQLADHYQQNTPIGPVLPDN
HYLSTQSALSKDPRDHMVLLEFVTAAGITH
>gi|589911345|pdb|1HCJ|A|Aequorea_victoria|Cnidarian-Hydrozoa
FTGVVPILVELDGDVNGHKFSVSGEGEGDATYGKLTLKFICTTGKLPVPWPTLVTTFXVQ
CFSRYPDHMHDFFKSAMPEGYVQERTIFFKDDGNYKTRAEVKFEGDTLVNRIELKGIDFK
EDGNILGHKLNYNSHNVYIMADKQGIKVNFKIRHNIEDGSVQLADHYQQNTPIGPVLPDN
HYLSTQSALSKDPRDHMVLLXFVTAAGITH
>gi|61680646|pdb|1YHG|A|Aequorea_victoria|Cnidarian-Hydrozoa
FTGVVPILVELDGDVNGHKFSVSGEGEGDATYGKLTLKFICTTGKLPVPWPTLVTTLGGQ
CFSRYPDHMHDFFKSAMPEGYVQERTISFKDDGNYKTRAEVKFEGDTLVNRIELKGIDFK
EDGNILGHKLNYNSHNVYITADKQGIKANFKIRHNIEDGSVQLADHYQQNTPIGPVLPDN
HYLSTQSALSKDPRDHMVLLEFVTAAGITH
>gi|61680648|pdb|1YHH|A|Aequorea_victoria|Cnidarian-Hydrozoa
FTGVVPILVELDGDVNGHKFSVSGEGEGDATYGKLTLKFICTTGKLPVPWPTLVTTLAVQ
CFSRYPDHMHDFFKSAMPEGYVQERTISFKDDGNYKTRAEVKFEGDTLVNRIELKGIDFK
EDGNILGHKLNYNSHNVYITADKQGIKANFKIRHNIEDGSVQLADHYQQNTPIGPVLPDN
HYLSTQSALSKDPRDHMVLLEFVTAAGITH
>gi|61680649|pdb|1YHI|A|Aequorea_victoria|Cnidarian-Hydrozoa
FTGVVPILVELDGDVNGHKFSVSGEGEGDATYGKLTLKFICTTGKLPVPWPTLVTTFGVQ
CFSRYPDHMHDFFKSAMPEGYVQEATISFKDDGNYKTRAEVKFEGDTLVNRIELKGIDFK
EDGNILGHKLNYNSHNVYITADKQGIKANFKIRHNIEDGSVQLADHYQQNTPIGPVLPDN
HYLSTQSALSKDPRDHMVLLEFVTAAGITH
>gi|61680686|pdb|1YJ2|A|Aequorea_victoria|Cnidarian-Hydrozoa
FTGVVPILVELDGDVNGHKFSVSGEGEGDATYGKLTLKFICTTGKLPVPWPTLVTTFXVQ
CFSRYPDHMHDFFKSAMPEGYVQERTISFKDDGNYKTRAEVKFEGDTLVNRIELKGIDFK
EDGNILGHKLNYNSGNVYITADKQGIKANFKIRHNIEDGSVQLADHYQQNTPIGPVLPDN
HYLSTQSALSKDPRDHMVLLEFVTAAGITH

>gi|61680691|pdb|1YJF|A|Aequorea_victoria|Cnidarian-Hydrozoa
FTGVVPILVELDGDVNGHKFSVSGEGEGDATYGKLTLKFICTTGKLPVPWPTLVTTFXVQ
CFSRYPDHMHDFFKSAMPEGYVQERTISFKDDGNYKTRAEVKFEGDTLVNRIELKGIDFK
EDGNILGHKLNYNSHNVYITADKQGIKANFKIRHNIEDGSVQLADHYQQNTPIGPVLPDN
HYLSTQSALSKDPRDHMVLLEFVTAAGITH
>gi|93279786|pdb|2FWQ|A|Aequorea_victoria|Cnidarian-Hydrozoa
FTGVVPILVELDGDVNGHKFSVSGEGEGDATYGKLTLKFICTTGKLPVPWPTLVTTLXVQ
CFSRYPDHMHDFFKSAMPEGYVQERTISFKDDGNYKTRAEVKFEGDTLVNRIELKGIDFK
EDGNILGHKLNYNSGNVYITADKQGIKANFKIRHNIEDGSVQLADHYQQNTPIGPVLPDN
HYLSTQSALSKDPRDHMVLLEFVTAAGITH
>gi|17026380|gb|AAL33912.1|AF435427_1|Aequorea_macrodactyla|Cnidarian-Hydrozoa
FTGIVPVLIELDGDVHGHKFSVRGEGEGDADYGKLEIKFICTTGKLPVPWPTLVTTLGIQ
CFARYPEHMNDFFKSAMPEGYIQERTIFFQDDGKYKTRGEVKFEGDTLVNRIELKGMDFK
EDGNILGHKLNFNSHNVYIMPDKAGLKVNFKIRHNIEGGGVQLADHYQTNVPLGPVLPIN
HYLSLQTAISKDRRDHMVFLEFFSACGHTH
>gi|17026382|gb|AAL33913.1|AF435428_1|Aequorea_macrodactyla|Cnidarian-Hydrozoa
FTGIVPVLIELDGDVHGHKFSVRGEGEGDADYGKLEIKFICTTGKLPVPWPTLVTTLGIQ
CFARYPEHMNDFFKSAMPEGYIQERTIFFQDDGKYKTRGEVKFEGDTLVNRIELKGMDFK
EDGNILGHKLNFNSHNVYIMPDKAGLKVNFKIRHNIEGGGVQLADHYQTNVPLGPVLPIN
HYLSFQTAISKDRRDHMVFLEFFSACGHTH
>gi|17026384|gb|AAL33914.1|AF435429_1|Aequorea_macrodactyla|Cnidarian-Hydrozoa
FTGIVPVLIELDGDVHGHKFSVRGEGEGDADYGKLEIKFICTTGKLPVPWPTLVTTLGIQ
CFARYPEHMNDFFKSAMPEGYIQERTIFFQDDGKYKTRGEVKFEGDTLVNRIELKGMDFK
EDGNILGHKLNFNSHNVYIMPDKAGLKVNFKIRHNIEGGGVQLADHYQTNVPLGPVLPIN
HYLSYQTAISKDRRDHMVFLEFFSACGHTH
>gi|17026386|gb|AAL33915.1|AF435430_1|Aequorea_macrodactyla|Cnidarian-Hydrozoa
FTGIVPVLIELDGDVHGHKFSVRGEGEGDADYGKLEIKFICTTGKLPVPWPTLVTTLGIL
CFARYPEHMNDFFKSAMPEGYIQERTIFFQDDGKYKTRGEVKFEGDTLVNRIELKGMDFK
EDGNILGHKLNFNSHNVYIMPDKAGLKVNFKIRHNIEGGGVQLADHYQTNVPLGPVLPIN
HYLSCQTAISKDRRDHMVFLEFFSACGHTH
>gi|17026388|gb|AAL33916.1|AF435431_1|Aequorea_macrodactyla|Cnidarian-Hydrozoa
FTGIVPVLIELDGDVHGHKFSVRGEGEGDADYGKLEIKFICTTGKLPVPWPTLVTTLGIQ
CFARYPEHMNDFFKSAMPEGYIQERTIFFQDDGKYKTRGEVKFEGDTLVNRIELKGMDFK
EDGNILGHKLNFNSHNVYIMPDKAGLKVNFKIRHNIEGGGVQLADHYQTNVPLGPVLPIN
HYLSTQTAISKDRRDHMVFLEFFSACGHTH
>gi|17026390|gb|AAL33917.1|AF435432_1|Aequorea_macrodactyla|Cnidarian-Hydrozoa
FTGVVPILVELDGDVHGHKFSVRGEGEGDADYGKLEIKFICTTGKLPVPWPTLVTTLGIL
CFARYPEHMNDFFKSAMPEGYIQERTIFFQDDGKYKTRGEVKFEGDTLVNRIELKGMDFK
EDGNILGHKLNFNSHNVYIMPDKAGLKVNFKIRHNIEGGGVQLADHYQTNVPLGPVLPIN
HYLSYQTAISKDRRDHMVFLEFFSACGHTH
>gi|17026392|gb|AAL33918.1|AF435433_1|Aequorea_macrodactyla|Cnidarian-Hydrozoa
FTGVVPILVELDGDVHGHKFSVRGEGEGDADYGKLEIKFICTTGKLPVPWPTLVTTLGIL
CFARYPEHMNDFFKSAMPEGYIQERTIFFQDDGKYKTRGEVKFEGDTLVNRIELKGMDFK
EDGNILGHKLNFNSHNVYIMPDKAGLKVNFKIRHNIEGGGVQLADHYQTNVPLGPVLPIN
HYLSTQTAISKDRRDHMVFLEFFSACGHTH
>gi|18175238|gb|AAK02059.1||Aequorea_macrodactyla|Cnidarian-Hydrozoa

FTGIVPVLIELDGDVHGHKFSVRGEGEGDADYGKLEIKFICTTGKLPVPWPTLVTTFGIQ
CFARYPEHMNDFFKSAMPEGYIQERTIFFQDDGKYKTRGEVKFEGDTLVNRIELKGMDFK
EDGNILGHKLNFNSHNVYIMPDKAGLKVNFKIRHNIEGGGVQLADHYQTNVPLGPVLPIN
HYLSTQTAISKDRRDHMVFLEFFSACGHTH
>gi|183186849|gb|ACC54354.1||Aldersladia_magnificus|Cnidarian-Hydrozoa
FTGIVPILIELNGDVHGHKFSVKGEGEGDATYGKLEIKFVCTTGKLPVPWPTLVTTFGVQ
CFARYPEHMHDFFKSAMPEGYIQERTIFFQDDGKYKTRAEVKFEGDTLVNRIELKGMDFK
EDGNILGHKLSYNSHNVYVMADKPGLKVNFKIRHNIEGGGVQLADHYQTNVPLGPVLPIN
HYLSTQTAITKDPRDHMVFLEFFTACGITH
>gi|197107230|pdb|3CB9|A|Aequorea_victoria|Cnidarian-Hydrozoa
FTGVVPILVELDGDVNGHKFSVSGEGEGDATYGKLTLKFISTTGKLPVPWPTLVTTLXVQ
CFSRYPDHMHDFFKSAMPEGYVQERTISFKDDGNYKTRAEVKFEGDTLVNRIELKGIDFK
EDGNILGHKLNYNCRNVYITADKQGIKANFKTRHNIEDGSVQLADHYQQNTPIGPVLPDN
HYLSTCSALSKDPRDHMVLLEFVTAAGITH
>gi|197107232|pdb|3CD1|A|Aequorea_victoria|Cnidarian-Hydrozoa
FTGVVPILVELDGDVNGHKFSVSGEGEGDATYGKLTLKFISTTGKLPVPWPTLVTTLXVQ
CFSRYPDHMHDFFKSAMPEGYVQERTISFKDDGNYKTRAEVKFEGDTLVNRIELKGIDFK
EDGNILGHKLNYNCENVYIMADKQGIKANFKTRHNIEDGSVQLADHYQQNTPIGPVLPDN
HYLSTCSALSKDPRDHMVLLEFVTAAGITH
>gi|675082447|pdb|4N3D|A|Aequorea_victoria|Cnidarian-Hydrozoa
FTGVVPILVELDGDVNGHKFSVSGEGEGDATYGKLTLKFICTTGKLPVPWPTLVTTLXVQ
CFSRYPDHMHDFFKSAMPEGYVQERTIFFKDDGNYKTRAEVKFEGDTLVNRIELKGIDFK
EDGNILGHKLNYNSHNVYIMADKQGIQVNFKIRHNIEDGSVQLADHYQQNTPIGPVLPDN
HYLSTQSALSKDPRDHMVLLEFVTAAGITL
>gi|217035443|pdb|3EVP|A|Aequorea_victoria|Cnidarian-Hydrozoa
FTGVVPILVELDGDVNGHKFSVSGEGEGDATYGKLTLKFICTTGKLPVPWPTLVTTLXVQ
CFSRYPDHMHDFFKSAMPEGYIQERTIFFKDDGNYKTRAEVKFEGDTLVNRIELKGIDFK
EDGNILGHKLYN--

>gi|217035444|pdb|3EVR|A|Aequorea_victoria|Cnidarian-Hydrozoa
FTGVVPILVELDGDVNGHKFSVSGEGEGDATYGKLTLKFICTTGKLPVPWPTLVTTLXVQ
CFSRYPDHMHDFFKSAMPEGYIQERTIFFKDDGNYKTRAEVKFEGDTLVNRIELKGIDFK
EDGNILGHKLNTRDQ----LTEEQEFKEAFSLFDKDGDGTIELQDMINEVDADGTIDP--
EFLTMMARKMKDTEEIREAFRVFDKDGNGY
>gi|217035445|pdb|3EVU|A|Aequorea_victoria|Cnidarian-Hydrozoa
FTGVVPILVELDGDVNGHKFSVSGEGEGDATYGKLTLKFICTTGKLPVPWPTLVTTLXVQ
CFSRYPDHMHDFFKSAMPEGYIQERTIFFKDDGNYKTRAEVKFEGDTLVNRIELKGIDFK
EDGNILGHKLNTRDQ----LTEEQEFKEAFSLFDKDGDGTIELQDMINEVDADGTIDP--
EFLTMMARKMKDTEEIREAFRVFDKDGNGY
>gi|23200274|pdb|1KP5|A|Aequorea_victoria|Cnidarian-Hydrozoa
FTGVVPILVELDGDVNGHKFSVSGEGEGDATYGKLTLKFICTTGKLPVPWPTLVTTFXVQ
CFSRYPDHMHDFFKSAMPEGYVQERTISFKDDGNYKTRAEVKFEGDTLVNRIELKGIDFK
EDGNILGHKLNYNSHNVYITADKQGIKANFKIRHNIEDGSVQLADHYQQNTPIGPVLPDN
HYLSTQSALSKDPRDHMVLLEFVTAAGLVP
>gi|290789997|pdb|3GEX|A|Aequorea_victoria|Cnidarian-Hydrozoa
FGGIVPILVELEGDVNGHKFSVSGEGEGDATYGKLTLKFICTTGKLPVPWPTLVTTLXVQ

CFSRYPDHMHDFFKSVMPEGYVQERTIFFKDDGNYKTRAEVKFEGDTLVNRIELKGIDFK
EDGNILGHKLNYISHNVYITADKQGIKANFKARHNITDGSVQLADHYQQNTPIGPVIPDN
HYLSTQSALSKDPRDHMVLLEFVTAAGITH
>gi|317455165|pdb|3LA1|A|Aequorea_victoria|Cnidarian-Hydrozoa
FGGIVPILVELEGDVNGHKFSVSGEGEGDATYGKLTLKFICTTGKLPVPWPTLVTTLXVQ
CFSRYPDHMHDFFKSVMPEGYVQERTIFFKDDGNYKTRAEVKFEGDTLVNRIELKGIDFK
EDGNILGHKLNYISHNVYITADKQGIKANFKIRHNITDGSVQLADHYQQNTPIGPVIPDN
HYLSTQSALSKDPRDHMVLLEFVTAAGITH
>gi|544191935|pdb|4H47|A|Aequorea_victoria|Cnidarian-Hydrozoa
FGGIVPILVELEGDVNGHKFSVSGEGEGDATYGKLTLKFICTTGKLPVPWPTLVTTLXVQ
CFSRYPDHMHDFFKSVMPEGYVQERTIFFKDDGNYKTRAEVKFEGDTLVNRIELKGIDFK
EDGNILGHKLNYISHNVYITADKQGIKANFKARHNITDGSVQLADHYQQNTPIGPVIPDN
HYLSTQSALSKDPRDHMVLLEFVTAAGITH
>gi|34421676|gb|AAN41637.1||Aequorea_coerulescens|Cnidarian-Hydrozoa
FTGVVPILIELNGDVNGHKFSVSGEGEGDATYGKLTLKFICTTGKLPVPWPTLVTTFGVQ
CFSRYPDHMHDFFKSAMPEGYIQERTIFFKDDGNYKSRAEVKFEGDTLVNRIELTGTDFK
EDGNILGNKMNYNAHNVYIMTDKAGIKVNFKIRHNIEDGSVQLADHYQQNTPIGPVLPDN
HYLSTQSTLSKDPRDHMIYFEFVTAAAITH
>gi|290790310|pdb|3LVA|A|Aequorea_coerulescens|Cnidarian-Hydrozoa
FTGIVPILIELNGDVNGHKFSVSGEGEGDATYGKLTLKFICTTGKLPVPWPTLVTTLXVQ
CFSRYPDHMHDFFKSAMPEGYIQERTIFFEDDGNYKSRAEVKFEGDTLVNRIELTGTDFK
EDGNILGNKMNYNAHNVYIMTDKAGIKVNFKIRHNIEDGSVQLADHYQQNTPIGPVLPDN
HYLSTQSALSKDPRDHMIYFGFVTAAAITH
>gi|290790312|pdb|3LVC|A|Aequorea_coerulescens|Cnidarian-Hydrozoa
FTGIVPILIELNGDVNGHKFSVSGEGEGDATYGKLTLKFICTTGKLPVPWPTLVTTLXVQ
CFSRYPDHMHDFFKSAMPEGYIQERTIFFEDDGNYKSRAEVKFEGDTLVNRIELTGTDFK
EDGNILGNKMNYNAHNVYIMTDKAGIKVNFKIRHNIEDGSVQLADHYQQNTPIGPVLPDN
HYLSTQSALSKDPRDHMIYFEFVTAAAITH
>gi|347582958|gb|AEP13896.1||Anthoathecata_sp._MH-2011|Cnidarian-Hydrozoa
FSKPMTCQTEIDGEINGNKFKVVGNGDSPGG-GDFSIHAYCTTGELPMSWVVLGSPLGFH
MFSGYPDDIIHYFQECFPEGYILTRSLRFEYDGTLTTTHHYSLEGNCVKAKVTLKGEGFD
PNGPTMTKEF-EEQHPSQVQIFPHGIRLLSNVVFKKKDGTTQLALQDCSVKPLRDVPPNV
HFLRTQIIQKKDDRDHVVQREIAIA-----
>gi|349587609|pdb|3P28|A|Aequorea_victoria|Cnidarian-Hydrozoa
---TGKLPVPWPTLVTTFXVQ
CFSRYPDHMHDFFKSAMPEGYVQERTISFKDDGNYKTRAEVKFEGDTLVNRIELKGIDFK
EDGNILGHKLNYNSHNVYITADKQGIKANFKIRHNIEDGSVQLADHYQQNTPIGPVLPDN
HYLSTQSKLSKDPRDHMVLLEFVTAAGIGT
>gi|383875275|pdb|2YE1|A|Aequorea_victoria|Cnidarian-Hydrozoa
FTGVVPILVELDGDVNGHKFSVSGEGEGDATYGKLTLKFICTTGKLPVPWPTLVTTLXVQ
CFARYPDHMHDFFKSAMPEGYVQERTIFFKDDGNYKTRAEVKFEGDTLVNRIELKGIDFK
EDGNILGHKLNYISGNVYITADKQGIKANFKIRHNIEDGGVQLADHYQQNTPIGPVLPDN
HYLSTQSALSKDPRDHMVLLEFLTAAGITL
>gi|400260715|pdb|4AR7|A|Aequorea_victoria|Cnidarian-Hydrozoa
FTGVVPILVELDGDVNGHKFSVSGEGEGDATYGKLTLKFICTTGKLPVPWPTLVTTLXVQ
CFARYPDHMHDFFKSAMPEGYVQERTIFFKDDGNYKTRAEVKFEGDTLVNRIELKGIDFK

EDGNILGHKLNYISDNVYITADKQGIKANFKIRHNIEDGGVQLADHYQQNTPIGPVLPDN
HYLSTQSKLSKDPRDHMVLLEFVTAAGITL
>gi|410562687|pdb|4AS8|A|Aequorea_victoria|Cnidarian-Hydrozoa
FTGVVPILVELDGDVNGHKFSVSGEGEGDATYGKLTLKFICTTGKLPVPWPTLVTTLXVQ
CFARYPDHMHDFFKSAMPEGYVQERTIFFKDDGNYKTRAEVKFEGDTLVNRIELKGIDFK
EDGNILGHKLNAISDNVYITADKQGIKANFKIRHNIEDGSVQLADHYQQNTPIGPVLPDN
HYLSTQSALSKDPRDHMVLLEFVTAAGITL
>gi|394985973|pdb|3SRY|A|Aequorea_victoria|Cnidarian-Hydrozoa
FTGVVPILVELDGDVNGHKFSVSGEGEGDATYGKLTLKFICTTGKLPVPWPTLVTTFXVQ
CFARYPDHMHDFFKSAMPEGYVQERTIFFKDDGNYKTRAEVKFEGDTLVNRIELKGIDFK
EDGNILGHKLNYNSHNVYIMADKQGIKVNFKIRHNIEDGSVQLADHYAQNTPIGPVLPDN
HYLSYQSALSKDPRDHMVLLEFVTAAGITL
>gi|394985978|pdb|3SSP|A|Aequorea_victoria|Cnidarian-Hydrozoa
FTGVVPILVELDGDVNGHKFSVSGEGEGDATYGKLTLKFICTTGKLPVPWPTLVTTFXVQ
CFARYPDHMHDFFKSAMPEGYVQERTIFFKDDGNYKTRAEVKFEGDTLVNRIELKGIDFK
EDGNILGHKLNYNSHNVYIMADKQGIKVNFKIRHNIEDGSVQLADHYQQNTPIGPVLPDN
HYLSYQSALSKDPRDHMVLLEFVTAAGITL
>gi|394985996|pdb|3ST0|A|Aequorea_victoria|Cnidarian-Hydrozoa
FTGVVPILVELDGDVNGHKFSVSGEGEGDATYGKLTLKFICTTGKLPVPWPTLVTTFXVT
CFARYPDHMHDFFKSAMPEGYVQERTIFFKDDGNYKTRAEVKFEGDTLVNRIELKGIDFK
EDGNILGHKLNYNSHNVYIMADKQGIKANFKIRHNIEDGSVQLADHYQQNTPIGPVLPDN
HYLSYQSALSKDPRDHMVLLEFVTAAGITL
>gi|402550703|pdb|4GES|B|Aequorea_victoria|Cnidarian-Hydrozoa
FTGVVPILVELDGDVNGHKFSVSGEGEGDATYGKLTLKFICTTGKLPVPWPTLVTTFGVQ
CFSRYPDHMHDFFKSAMPEGYVQERTISFKDDGNYKTRAEVKFEGDTLVNRIELKGIDFK
EDGNILGHKLNYNSHNVXITADKQGIKANFKIRHNIEDGSVQLADHYQQNTPIGPVLPDN
HYLSTQSALSKDPRDHMVLLEFVTAAGITH
>gi|380763700|pdb|2YDZ|A|Aequorea_victoria|Cnidarian-Hydrozoa
FTGVVPILVELDGDVNGHKFSVSGEGEGDATYGKLTLKFICTTGKLPVPWPTLVTTLXVQ
CFARYPDHMHDFFKSAMPEGYVQERTIFFKDDGNYKTRAEVKFEGDTLVNRIELKGIDFK
EDGNILGHKLNYISDNVYITADKQGIKANFKIRHNIEDGGVQLADHYQQNTPIGPVLPDN
HYLSTQSALSKDPRDHMVLLEFVTAAGITL
>gi|380764941|pdb|3ZTF|A|Aequorea_victoria|Cnidarian-Hydrozoa
FTGVVPILVELDGDVNGHKFSVSGEGEGDATYGKLTLKFICTTGKLPVPWPTLVTTLXVQ
CFARYPDHMHDFFKSAMPEGYVQERTIFFKDDGNYKTRAEVKFEGDTLVNRIELKGIDFK
EDGNILGHKLNYFSDNVYITADKQGIKANFKIRHNIEDGGVQLADHYQQNTPIGPVLPDN
HYLSTQSALSKDPRDHMVLLEFVTAAGITL
>gi|402550708|pdb|4GF6|B|Aequorea_victoria|Cnidarian-Hydrozoa
FTGVVPILVELDGDVNGHKFSVSGEGEGDATYGKLTLKFICTTGKLPVPWPTLVTTFGVQ
CFSRYPDHMHDFFKSAMPEGYVQERTISFKDDGNYKTRAEVKFEGDTLVNRIALKGIDFK
EAGNILGHKLNYNSHNVXITADKQGIKANFKIRHNIADGSVQLADHYQQNTPIGPVLPDN
HYLSTQSALSKDPRDHMVLLEFVTAAGITH
>gi|606384|gb|AAA58246.1||Aequorea_victoria|Cnidarian-Hydrozoa
FTGVVPILVELDGDVNGQKFSVSGEGEGDATYGKLTLKFICTTGKLPVPWPTLVTTFGVQ
CFSRYPDHMHDFFKSAMPEGYVQERTIFYKDDGNYKTRAEVKFEGDTLVNRIELKGIDFK
EDGNILGHKMNYNSHNVYIMADKPGIKVNFKIRHNIKDGSVQLADHYQQNTPIGPVLPDN

HYLSTQSALSKDPRDHMILLEFVTAAGITH
>gi|408535774|pdb|3VHT|A|Aequorea_victoria|Cnidarian-Hydrozoa
FTGVVPILVELDGDVNGHKFSVSGEGEGDATYGKLTLKFICTTGKLPVPWPTLVTTFXVQ
CFARYPDHMHDFFKSAMPEGYVQERTIFFKDDGNYKTRAEVKFEGDTLVNRIELKGIDFK
EDGNILGHKLNYNSHNVYIMADKQGIKVNFKIRHNIEDGSVQLADHYQQNTPIGPVLPDN
HYLSTQSALSKDPRDHMVLLEFVTAAGITG
>gi|225734277|pdb|3GJ1|A|Aequorea_victoria|Cnidarian-Hydrozoa
FTGVVPILVELDGDVNGHKFSVSGEGEGDATYGKLTLKFICTTGKLPVPWPTLVTTFXVQ
CFSRYPDHMHDFFKSAMPEGYVQERTISFKDDGNYKTRAEVKFEGDTLVNRIELKGIDFK
EDGNILGHKLNYNSHNVYITADKQGIKANFKIRHNIEDGSVQLADHYQQNTPIGPVLPDN
HYLSHQSALSKDPRDHMVLLEFVTAAGIT-
>gi|227343653|pdb|2QRF|A|Aequorea_victoria|Cnidarian-Hydrozoa
FTGVVPILVELDGDVNGHKFSVSGEGEGDATYGKLTLKFICTTGKLPVPWPTLVTTLXVQ
CFSRYPDHMHDFFKSAMPEGYVQERTISFKDDGNYKTRAEVKFEGDTLVNRIELKGIDFK
EDGNILGHKLNYNSHNVYITADKQGIKANFKIRHNIEDGSVQLADHYEQNTPIGPVLPDN
HYLSTQSALSKDPRDHMVLLEFVTAAGIT-
>gi|227343655|pdb|2QZ0|A|Aequorea_victoria|Cnidarian-Hydrozoa
FTGVVPILVELDGDVNGHKFSVSGEGEGDATYGKLTLKFICTTGKLPVPWPTLVTTLXVQ
CFSRYPDHMHDFFKSAMPEGYVQERTISFKDDGNYKTRAEVKFEGDTLVNRIELKGIDFK
EDGNILGHKLNYNSHNVYITADKQGIKANFKIRHNIEDGSVQLADHYEQNTPIGPVLPDN
HYLSTQSALSKDPRDHMVLLEFVTAAGI--
>gi|37928101|pdb|1QXT|A|Aequorea_victoria|Cnidarian-Hydrozoa
FTGVVPILVELDGDVNGHKFSVSGEGEGDATYGKLTLKFICTTGKLPVPWPTLVTTLGVQ
CFSRYPDHMHDFFKSAMPEGYVQEATISFKDDGNYKTRAEVKFEGDTLVNRIELKGIDFK
EDGNILGHKLNYNSHNVYITADKQGIKANFKIRHNIEDGSVQLADHYQQNTPIGPVLPDN
HYLSTQSALSKDPRDHMVLLEFVTAAGI--
>gi|37928105|pdb|1QY3|A|Aequorea_victoria|Cnidarian-Hydrozoa
FTGVVPILVELDGDVNGHKFSVSGEGEGDATYGKLTLKFICTTGKLPVPWPTLVTTLGVQ
CFSRYPDHMHDFFKSAMPEGYVQEATISFKDDGNYKTRAEVKFEGDTLVNRIELKGIDFK
EDGNILGHKLNYNSHNVYITADKQGIKANFKIRHNIEDGSVQLADHYQQNTPIGPVLPDN
HYLSTQSALSKDPRDHMVLLEFVTAAGI--
>gi|37928112|pdb|1QYF|A|Aequorea_victoria|Cnidarian-Hydrozoa
FTGVVPILVELDGDVNGHKFSVSGEGEGDATYGKLTLKFICTTGKLPVPWPTLVTTLXVQ
CFSRYPDHMHDFFKSAMPEGYVQEATISFKDDGNYKTRAEVKFEGDTLVNRIELKGIDFK
EDGNILGHKLNYNSHNVYITADKQGIKANFKIRHNIEDGSVQLADHYQQNTPIGPVLPDN
HYLSTQSALSKDPRDHMVLLEFVTAAGI--
>gi|3891568|pdb|1YFP|A|Aequorea_victoria|Cnidarian-Hydrozoa
FTGVVPILVELDGDVNGHKFSVSGEGEGDATYGKLTLKFICTTGKLPVPWPTLVTTFXLQ
CFARYPDHMHDFFKSAMPEGYVQERTIFFKDDGNYKTRAEVKFEGDTLVNRIELKGIDFK
EDGNILGHKLNYNSHNVYIMADKQGIKVNFKIRHNIEDGSVQLADHYQQNTPIGPVLPDN
HYLSYQSALSKDPRDHMVLLEFVTAAGI--
>gi|49258783|pdb|1RRX|A|Aequorea_victoria|Cnidarian-Hydrozoa
FTGVVPILVELDGDVNGHKFSVSGEGEGDATXGKLTLKFICTTGKLPVPWPTLVTTLXVQ
CFSRXPDHMHDFFKSAMPEGXVQERTIFFKDDGNXKTRAEVKFEGDTLVNRIELKGIDFK
EDGNILGHKLNXNSHNVXIMADKQGIKVNFKIRHNIEDGSVQLADHXQQNTPIGPVLPDN
HXLSTQSALSKDPRDHMVLLEFVTAAGI--

>gi|665764546|pdb|4P1Q|A|Aequorea_victoria|Cnidarian-Hydrozoa
FTGVVPILVELDGDVNGHKFSVSGEGEGDATYGKLTLKFICTTGKLPVPWPTLVTTFXVQ
CFSRYPDHMHDFFKSAMPEGYVQERTISFKDDGNYKTRAEVKFEGDTLVNRIELKGIDFK
EDGNILGHKLNYNSHNVYITADKQGIKANFKIRHNIEDGSVQLADHYQQNTPIGPVLPDN
HYLSTQSALSKDPRDHMVLLHFVTAAGITH
>gi|99031842|pdb|2AWJ|A|Aequorea_victoria|Cnidarian-Hydrozoa
FTGVVPILVELDGDVNGHKFSVSGEGEGDATYGKLTLKFICTTGKLPVPWPTLVTTLGVQ
CFSRYPDHMHDFFKSAMPEGYVQEMTISFKDDGNYKTRAEVKFEGDTLVNRIELKGIDFK
EDGNILGHKLNYNSHNVYITADKQGIKANFKIRHNIEDGSVQLADHYQQNTPIGPVLPDN
HYLSTQSALSKDPRDHMVLLEFVTAAGI--
>gi|99031843|pdb|2AWK|A|Aequorea_victoria|Cnidarian-Hydrozoa
FTGVVPILVELDGDVNGHKFSVSGEGEGDATYGKLTLKFICTTGKLPVPWPTLVTTLXVQ
CFSRYPDHMHDFFKSAMPEGYVQEMTISFKDDGNYKTRAEVKFEGDTLVNRIELKGIDFK
EDGNILGHKLNYNSHNVYITADKQGIKANFKIRHNIEDGSVQLADHYQQNTPIGPVLPDN
HYLSTQSALSKDPRDHMVLLEFVTAAGI--
>gi|99031844|pdb|2AWL|A|Aequorea_victoria|Cnidarian-Hydrozoa
FTGVVPILVELDGDVNGHKFSVSGEGEGDATYGKLTLKFICTTGKLPVPWPTLVTTLXVQ
CFSRYPDHMHDFFKSAMPEGYVQEKTISFKDDGNYKTRAEVKFEGDTLVNRIELKGIDFK
EDGNILGHKLNYNSHNVYITADKQGIKANFKIRHNIEDGSVQLADHYQQNTPIGPVLPDN
HYLSTQSALSKDPRDHMVLLEFVTAAGI--
>gi|99031845|pdb|2AWM|A|Aequorea_victoria|Cnidarian-Hydrozoa
FTGVVPILVELDGDVNGHKFSVSGEGEGDATYGKLTLKFICTTGKLPVPWPTLVTTLXVQ
CFSRYPDHMHDFFKSAMPEGYVQEATISFKDDGNYKTRAEVKFEGDTLVNRIELKGIDFK
EDGNILGHKLNYNSHNVYITADKQGIKANFKIRHNIEDGSVQLADHYRQNTPIGPVLPDN
HYLSTQSALSKDPRDHMVLLEFVTAAGI--
>gi|408535775|pdb|3VHT|B|Aequorea_victoria|Cnidarian-Hydrozoa
FTGVVPILVELDGDVNGHKFSVSGEGEGDATYGKLTLKFICTTGKLPVPWPTLVTTFXVQ
CFARYPDHMHDFFKSAMPEGYVQERTIFFKDDGNYKTRAEVKFEGDTLVNRIELKGIDFK
EDGNILGHKLNYNSHNVYIMADKQGIKVNFKIRHNIEDGSVQLADHYQQNTPIGPVLPDN
HYLSTQSALSKDPRDHMVLLEFVTAAGITG
>gi|409106990|pdb|3UFZ|A|Aequorea_victoria|Cnidarian-Hydrozoa
FAGVVPILVELDGDVNGHKFSVSGEGEGDATYGKLTLKFICTTGKLPVPFPTLVTTLXVQ
CFSRYPDHMHDFFKSAMPEGYVQERTISFKDDGNYKTRAEVKFEGDTLVNRIELKGIDFK
EDGNILGHKLNYNSHNVYITADKQGIKANFKIRHNIEDGSVQLADHYQQNTPIGPVLPDN
HCLSTQTVLSKDPRDHMVLLEFVTAAGI--
>gi|37926353|pdb|1CV7|A|Aequorea_victoria|Cnidarian-Hydrozoa
FTGVVPILVELDGDVNGHRFSVSGEGEGDATYGKLTLKFICTTGKLPVPWPTLVTTLXVQ
CFSRYPDHMHDFFKSAMPEGYVQERTIFFKDDGNYKTRAEVKFEGDTLVNRIELKGIDFK
EDGNILGHKLNYISHNVYITADKQGIKAHFKIRHNIEDGSVQLADHYQQNTPIGPVLPDN
HYLSTQSALSKDPRDHMVLLEFVTAAG---
>gi|49258765|pdb|1RMM|A|Aequorea_victoria|Cnidarian-Hydrozoa
FTGVVPILVELDGDVNGHKFSVSGEGEGDATYGKLTLKFICTTGKLPVPXPTLVTTLXVQ
CFSRYPDHMHDFFKSAMPEGYVQERTIFFKDDGNYKTRAEVKFEGDTLVNRIELKGIDFK
EDGNILGHKLNYNSHNVYIMADKQGIKVNFKIRHNIEDGSVQLADHYQQNTPIGPVLPDN
HYLSTQSALSKDPRDHMVLLEFVTAAGI--
>gi|433286590|pdb|3V3D|A|Aequorea_victoria|Cnidarian-Hydrozoa

FTGVVPILVELDGDVNGHKFSVSGEGEGDATYGKLTLKFICTTGKLPVPWPTLVTTFXLQ
CFARYPDHMHDFFKSAMPEGYVQERTIFFKDDGNYKTRAEVKFEGDTLVNRIELKGIDFK
EDGNILGHKLNYNSHNVYIMADKQGIKVNFKIRHNIEDGSVQLADHYQQNTPIGPVLPDN
HYLSFQSALSKDPRDHMVLLEFVTAAGITL
>gi|304446102|pdb|3OGO|A|Aequorea_victoria|Cnidarian-Hydrozoa
FTGVVPILVELDGDVNGHKFSVSGEGEGDATYGKLTLKFICTTGKLPVPWPTLVTTLXVQ
CFSRYPDHMHDFFKSAMPEGYVQERTIFFKDDGNYKTRAEVKFEGDTLVNRIELKGIDFK
EDGNILGHKLNYNSHNVYIMADKQGIKVNFKIRHNIEDGSVQLADHYQQNTPIGPVLPDN
HYLSTQSALSKDPRDHMVLLEFVTAAGITL
>gi|330689315|pdb|2Y0G|A|Aequorea_victoria|Cnidarian-Hydrozoa
FTGVVPILVELDGDVNGHKFSVSGEGEGDATYGKLTLKFICTTGKLPVPWPTLVTTLXVQ
CFSRYPDHMHDFFKSAMPEGYVQERTIFFKDDGNYKTRAEVKFEGDTLVNRIELKGIDFK
EDGNILGHKLNYNSHNVYIMADKQGIKVNFKIRHNIEDGSVQLADHYQQNTPIGPVLPDN
HYLSTQSALSKDPRDHMVLLEFVTAAGITL
>gi|346651994|pdb|3ST2|A|Aequorea_victoria|Cnidarian-Hydrozoa
FTGVVPILVELDGDVNGHKFSVSGEGEGDATYGKLTLKFICTTGKLPVPWPTLLTTIXLM
CFARYPDHMHDFFKSAMPEGYVQERTIFFKDDGNYKTRAEVKFEGDTLVNRIELKGIDFK
EDGNILGHKLNHDSHNVYIMADKQGIKVNFKIRHNIEDGSVQLADHYQQNTPIGPVLPDN
HYLSYQSALSKDPRDHMVLLEFVTAAGITL
>gi|482677107|pdb|4EN1|A|Aequorea_victoria|Cnidarian-Hydrozoa
FTGVVPILVELDGDVNGHKFSVSGEGEGDATYGKLTLKFICTTGKLPVPWPTLVTTLXVQ
CFARYPDHMHDFFKSAMPEGYVQERTIFFKDDGNYKTRAEVKFEGDTLVNRIELKGIDFK
EDGNILGHKLNAIHGNVYITADKQGIKANFGLNCNIEDGSVQLADHYQQNTPIGPVLPDN
HYLSTQSKLSKDPRDHMVLLEFVTAAGITL
>gi|161172181|pdb|2Q57|A|Aequorea_victoria|Cnidarian-Hydrozoa
FTGVVPILVELDGDVNGHKFSVSGEGEGDATYGKLTLKFICTTGKLPVPWPTLVTTLXVQ
CFARYPDHMHDFFKSAMPEGYVQERTIFFKDDGNYKTRAEVKFEGDTLVNRIELKGIDFK
EDGNILGHKLNAISDNVYITADKQGIKANFKIRHNIEDGSVQLADHYQQNTPIGPVLPDN
HYLSTQSALSKDPRDHMVLLEFVTAAGITL
>gi|609412314|pdb|4KW4|A|Aequorea_victoria|Cnidarian-Hydrozoa
FTGVVPILVELDGDVNGHKFSVSGEGEGDATYGKLTLKFICTTGKLPVPWPTLVTTLXVQ
CFARYPDHMHDFFKSAMPEGYVQERTIFFKDDGNYKTRAEVKFEGDTLVNRIELKGIDFK
EDGNILGHKLNYNHHKVYITADKQGIKVNFKTRHNIEDGSVQLADHYQQNTPIGPVLPDN
HYLHTHSKLSKDPRDHMVLLEFVTAAGITL
>gi|157830967|pdb|1EMM|A|Aequorea_victoria|Cnidarian-Hydrozoa
FTGVVPILVELDGDVNGHKFSVSGEGEGDATYGKLTLKFICTTGKLPVPWPTLVTTLXVQ
CFSRYPDHMHDFFKSAMPEGYVQERTIFFKDDGNYKTRAEVKFEGDTLVNRIELKGIDFK
EDGNILGHKLNYNSHNVYIMADKQGIKVNFKIRHNIEDGSVQLADHYQQNTPIGPVLPDN
HYLSTQSALSKDPRDHMVLLEFVTAAGITH
>gi|161760919|pdb|2OKW|A|Aequorea_victoria|Cnidarian-Hydrozoa
FTGVVPILVELDGDVNGHKFSVSGEGEGDATYGKLTLKFICTTGKLPVPWPTLVTTLXVQ
CFSRYPDHMHDFFKSAMPEGYVQERTIFFKDDGNYKTRAEVKFEGDTLVNRIELKGIDFK
EDGNILGHKLNYNSHNVYIMADKQGIKVNFKIRHNIEDGSVQLADHYQQNTPIGPVLPDN
HYLSTQCALSKDPRDHMVLLEFVTAAGITH
>gi|178847036|pdb|2HJO|A|Aequorea_victoria|Cnidarian-Hydrozoa
FTGVVPILVELDGDVNGHKFSVSGEGEGDATYGKLTLKFICTTGKLPVPWPTLVTTLXVQ

CFSRYPDHMHDFFKSAMPEGYVQERTIFFKDDGNYKTRAEVKFEGDTLVNRIELKGIDFK
EDGNILGHKLNYNSHNVYIMADKQGIKVNFKIRHNIEDGSVQLADHYQQNTPIGPVLPDN
HYLSTQSALSKDPRDHMVLLEFHTAAGITH
>gi|178847037|pdb|2HQZ|A|Aequorea_victoria|Cnidarian-Hydrozoa
FTGVVPILVELDGDVNGHKFSVSGEGEGDATYGKHTLKFICTTGKLPVPWPTLVTTLXVQ
CFSRYPDHMHDFFKSAMPEGYVQERTIFFKDDGNYKTRAEVKFEGDTLVNRIELKGIDFK
EDGNILGHKLNYNSHNVYIMADKQGIKVNFKIRHNIEDGSVQLADHYQQNTPIGPVLPDN
HYLSTQSALSKDPRDHMVLLEFVTAAGITH
>gi|2392317|pdb|1EMC|A|Aequorea_victoria|Cnidarian-Hydrozoa
FTGVVPILVELDGDVNGHKFSVSGEGEGDATYGKLTLKFICTTGKLPVPWPTLVTTLXVQ
CFSRYPDHMHDFFKSAMPEGYVQERTIFFKDDGNYKTRAEVKFEGDTLVNRIELKGIDFK
EDGNILGHKLNYNSHNVYIMADKQGIKVNFKTRHNIEDGSVQLADHYQQNTPIGPVLPDN
HYLSTQSALSKDPRDHMVLLEFVTAAGITH
>gi|260099862|pdb|2WSN|A|Aequorea_victoria|Cnidarian-Hydrozoa
FTGVVPILVELDGDVNGHKFSVSGEGEGDATYGKLTLKFICTTGKLPVPWPTLVTTLXVQ
CFSRYPDHMHDFFKSAMPEGYVQERTIFFKDDGNYKTRAEVKFEGDTLVNRIELKGIDFK
EDGNILGHKLNYISHNVYITADKQGIKANFKIRHNIEDGSVQLADHYQQNTPIGPVLPDN
HYLSTQSALSKDPRDHMVLLEFVTAAGITL
>gi|260099863|pdb|2WSO|A|Aequorea_victoria|Cnidarian-Hydrozoa
FTGVVPILVELDGDVNGHKFSVSGEGEGDATYGKLTLKFICTTGKLPVPWPTLVTTLXVQ
CFARYPDHMHDFFKSAMPEGYVQERTIFFKDDGNYKTRAEVKFEGDTLVNRIELKGIDFK
EDGNILGHKLNAISDNVYITADKQGIKANFKIRHNIEDGSVQLADHYQQNTPIGPVLPDN
HYLSTQSALSKDPRDHMVLLEFVTAAGITL
>gi|295982061|pdb|2WUR|A|Aequorea_victoria|Cnidarian-Hydrozoa
FTGVVPILVELDGDVNGHKFSVSGEGEGDATYGKLTLKFICTTGKLPVPWPTLVTTLXVQ
CFSRYPDHMHDFFKSAMPEGYVQERTIFFKDDGNYKTRAEVKFEGDTLVNRIELKGIDFK
EDGNILGHKLNYNSHNVYIMADKQGIKVNFKTRHNIEDGSVQLADHYQQNTPIGPVLPDN
HYLSTQSALSKDPRDHMVLLEFVTAAGITH
>gi|37928121|pdb|1QYQ|A|Aequorea_victoria|Cnidarian-Hydrozoa
FTGVVPILVELDGDVNGHKFSVSGEGEGDATYGKLTLKFICTTGKLPVPWPTLVTTLXVQ
CFSRYPDHMHDFFKSAMPEGYVQERTISFKDDGNYKTRAEVKFEGDTLVNRIELKGIDFK
EDGNILGHKLNYNSHNVYITADKQGIKANFKIRHNIEDGSVQLADHYQQNTPIGPVLPDN
HYLSTQSALSKDPRDHMVLLEFVTAAGITH
>gi|407943868|pdb|4EUL|A|Aequorea_victoria|Cnidarian-Hydrozoa
FTGVVPILVELDGDVNGHKFSVSGEGEGDATYGKLTLKFICTTGKLPVPWPTLVTTLXVQ
CFSRYPDHMHDFFKSAMPEGYVQERTIFFKDDGNYKTRAEVKFEGDTLVNRIELKGIDFK
EDGNILGHKLNYNSHNVYIMADKQGIKVNFKIRHNIEDGSVQLADHYQQNTPIGPVLPDN
HYLSTQSALSKDPRDHMVLLEFVTAAGITL
>gi|49258764|pdb|1RM9|A|Aequorea_victoria|Cnidarian-Hydrozoa
FTGVVPILVELDGDVNGHKFSVSGEGEGDATYGKLTLKFICTTGKLPVPXPTLVTTLXVQ
CFSRYPDHMHDFFKSAMPEGYVQERTIFFKDDGNYKTRAEVKFEGDTLVNRIELKGIDFK
EDGNILGHKLNYISHNVYITADKQGIKANFKIRHNIEDGSVQLADHYQQNTPIGPVLPDN
HYLSTQSALSKDPRDHMVLLEFVTAAGITL
>gi|49258766|pdb|1RMO|A|Aequorea_victoria|Cnidarian-Hydrozoa
FTGVVPILVELDGDVNGHKFSVSGEGEGDATYGKLTLKFICTTGKLPVPXPTLVTTLXVQ
CFSRYPDHMHDFFKSAMPEGYVQERTIFFKDDGNYKTRAEVKFEGDTLVNRIELKGIDFK

EDGNILGHKLNYNSHNVYIMADKQGIKVNFKIRHNIEDGSVQLADHYQQNTPIGPVLPDN
HYLSTQSALSKDPRDHMVLLEFVTAAGITL
>gi|550545393|pdb|3W1C|A|Aequorea_victoria|Cnidarian-Hydrozoa
FTGVVPILVELDGDVNGHKFSVSGEGEGDATYGKLTLKFICTTGKLPVPWPTLVTTFXLQ
CFARYPDHMHDFFKSAMPEGYVQERTIFFKDDGNYKTRAEVKFEGDTLVNRIELKGIDFK
EDGNILGHKLNYNSHNVYIMADKQGIKVNFKIRHNIEDGSVQLADHYQQNTPIGPVLPDN
HYLSYQSALSKDPRDHMVLLEFVTAAGITL
>gi|564731093|pdb|4L12|A|Aequorea_victoria|Cnidarian-Hydrozoa
FTGVVPILVELDGDVNGHKFSVSGEGEGDATYGKLTLKFICTTGKLPVPWPTLVTTLXVQ
CFSRYPDHMHDFFKSAMPEGYVQERTIFFKDDGNYKTRAEVKFEGDTLVNRIELKGIDFK
EDGNILGHKLNYNEHNVYITADKQGIKANFKIRHNIEDGSVQLADHYQQNTPIGPVLPDN
HYLDTESALSKDPRDHMVLLEEVEAAGITL
>gi|672885647|pdb|4KAG|A|Aequorea_victoria|Cnidarian-Hydrozoa
FTGVVPILVELDGDVNGHKFSVSGEGEGDATYGKLTLKFICTTGKLPVPWPTLVTTLXVQ
CFSRYPDHMHDFFKSAMPEGYVQERTIFFKDDGNYKTRAEVKFEGDTLVNRIELKGIDFK
EDGNILGHKLNYNSHNVYIMADKQGIKVNFKIRHNIEDGSVQLADHYQQNTPIGPVLPDN
HYLSTQSALSKDPRDHMVLLEFVTAAGITL
>gi|672885648|pdb|4KEX|A|Aequorea_victoria|Cnidarian-Hydrozoa
FTGVVPILVELDGDVNGHKFSVSGEGEGDATYGKLTLKFICTTGKLPVPWPTLVTTLXVQ
CFSRYPDHMHDFFKSAMPEGYVQERTIFFKDDGNYKTRAEVKFEGDTLVNRIELKGIDFK
EDGNILGHKLNYNSHNVYIMADKQGIKVNFKIRHNIEDGSVQLADHYQQNTPIGPVLPDN
HYLSTQSALSKDPRDHMVLLEFVT-AGITL
>gi|99032351|pdb|2G16|A|Aequorea_victoria|Cnidarian-Hydrozoa
FTGVVPILVELDGDVNGHKFSVSGEGEGDATYGKLTLKFICTTGKLPVPWPTLVTTF---

>gi|99032370|pdb|2G2S|A|Aequorea_victoria|Cnidarian-Hydrozoa
FTGVVPILVELDGDVNGHKFSVSGEGEGDATYGKLTLKFICTTGKLPVPWPTLVTTL---

>gi|40365353|gb|AAR85350.1||Anthomedusae_sp._SL-2003|Cnidarian-Hydrozoa
FQKKLPYKLELDGDVDGQTFKVIGEGVGDATTGVIEGKYVCTEGEVPISWVSLITSLGAK
CFVRYPNEINDFFKSTFPSGYHQERKITYENDGVLETAAKITMESGAIVNRINVKGTGFD
KDGHVCQKNLSSPPSTTYVVPEGEGIRIIYRNIYPTKDGHYVVADTQQVNRPIQGTSPTY
HHIKSKVDLSTDPKDHIIIKETNCAFDADF
>gi|40365355|gb|AAR85351.1||Anthomedusae_sp._SL-2003|Cnidarian-Hydrozoa
FEKPLPYKVELEGDVDGQKFTVIGEGQGDASTGRVEGKYVCTKGEVPISWVSLITSLGGK
CFVRYPNVIKDFFKSTFPTGYHQERKITYEDDGVLETAAKVTLESGAIYNRISVKGVGFK
KDGNVCKKRLSSPPQVSYVVPYGEGIRVLYSNIYPTKDGGYVVADTRQVNRPIEGKSPKY
HYIKSKIDLSTDPKDHIIIKEVNVASGIDF
>gi|347726913|gb|AEP19816.1||Clytia_hemisphaerica|Cnidarian-Hydrozoa
FNKELPFVCELDGEINGHKFSVRGKGIGNSAQGLTHGIHVCTSGELPVSWTAITHNLGLL
CFSQYPHDINDYIKSTFPEGYTKERTSNFDGDGKYTSRHVITYENGCIYNRVTINGSGFS
DDGNVLGKNLEKPVCSMYFPGKD-GLRAEICKLTETKDGGYQSCREDQVIRPIGPTVMTH

HYNYSSVEYSKDARDHIVMKEITKVSHFI-
>gi|347726915|gb|AEP19817.1||Clytia_hemisphaerica|Cnidarian-Hydrozoa
FNKELPFVVEVDFDIDDHKFSIRGKGVGNAEKGLMVGKYVVSEGELPCSWSAITHNFGQV
CFTRYPKDIPDHIKSLFPEGYVQTRHSHFVDDGEYTSVHTLTYENGVIYNRVKVNGGGFK
PDGNVFGKRLEPDICSVYFPGKD-GYNCEFVKLSETVDGDYQAIRIDQVIRPLGPSLMTK
LYHHYKFEYSKDAREHIIMKEQVHASHHTS
>gi|294662355|pdb|3A8S|A|Anthomedusae_sp._DC-2005|Cnidarian-Hydrozoa
FQSDMTFKIFIDGEVNGQKFTIVADGSSKFPHGDFNVHAVCETGKLPMSWKPICHLIXEP
FFARYPDGISHFAQECFPEGLSIDRTVRFENDGTMTSHHTYELDDTCVVSRITVNCDGFQ
PDGPIMRDQLDILPNETHMFPHGPAVRQLAFIGFTTADGGLMMGHFDSKMTFNRAIEPGP
HFVTIITKQMRDTRDHVCQREVAYAHSVPR
>gi|257471790|pdb|3GB3|A|Anthomedusae_sp._DC-2005|Cnidarian-Hydrozoa
FQSDMTFKIFIDGEVNGQKFTIVADGSSKFPHGDFNVHAVCETGKLPMSWKPICHLIXEP
FFARYPDGISHFAQECFPEGLSIDRTVRFENDGTMTSHHTYELDDTCVVSRITVNCDGFQ
PDGPIMRDQLDILPNETHMFPHGPAVRQLAFIGFTTADGGLMMGHFDSKMTFNRAIEPGP
HFVTIITKQMRDTRDHVCQREVAYAHSVPR
>gi|442570522|pdb|4B30|A|Anthomedusae_sp._DC-2005|Cnidarian-Hydrozoa
FQSDMTFKIFIDGEVNGQKFTIVADGSSKFPHGDFNAHAVCETGKLPMSWKPICHLIXEP
FFARYPDGISHFAQECFPEGLSIDRTVRFENDGTMTSHHTYELDDTXVVSRITVNCDGFQ
PDGPIMRDQLDILPNETHMFPHGPAVRQLAFIGFTTADGGLMMGHFDSKMTFNRAIEPGP
HFVTIITKQMRDTRDHVXQREVAYAHSVPR
>gi|442570523|pdb|4B30|B|Anthomedusae_sp._DC-2005|Cnidarian-Hydrozoa
FQSDMTFKIFIDGEVNGQKFTIVADGSSKFPHGDFNAHAVCETGKLPMSWKPICHLIXEP
FFARYPDGISHFAQECFPEGLSIDRTVRFENDGTMTSHHTYELDDTXVVSRITVNCDGFQ
PDGPIMRDQLDILPNETHMFPHGPAVRQLAFIGFTTADGGLMMGHFDSKMTFNRAIEPGP
HFVTIITKQMRDTRDHVCQREVAYAHSVPR
>gi|63253819|gb|AAY40168.1||Anthomedusae_sp._DC-2005|Cnidarian-Hydrozoa
FQSDMTFKIFIDGEVNGQKFTIVADGSSKFPHGDFNVHAVCETGKLPMSWKPICHLIGEP
FFARYPDGISHFAQECFPEGLSIDRTVRFENDGTMTSHHTYELDDTCVVSRITVNCDGFQ
PDGPIMRDQLDILPNETHMFPHGPAVRQLAFIGFTTADGGLMMGHFDSKMTFNRAIEPGP
HFVTIITKQMRDTRDHVCQREVAYAHSVPR
>gi|40365357|gb|AAR85352.1||Anthomedusae_sp._SL-2003|Cnidarian-Hydrozoa
FQSDMTFKIFIDGVVNDQKFTIIADGSSKFPHGDFNVHAVCETGKLPMSWKPICHLIGEP
FFAKYPNGISHFAQECFPEGLTIDRTVRFENDGTMTSHHTYELDGTCVISRITVNCDGFQ
PDGPIMKDQLDILPTETHMFPHGSAVRQLCYIGFTTADGGLMMSHFDSKLTFNRAIKPGP
HFVTVIIKQMKDTRDHVCQREVTYAHSVPR
>gi|255917805|pdb|2WIQ|A|Anthomedusae_sp._DC-2005|Cnidarian-Hydrozoa
FQSDMTFKIFIDGEVNGQKFTIVADGSSKFPHGDFNVHAVCETGKLPMSWKPICHLIXEP
FFARYPDGISHFAQECFPEGLSIDRTVRFENDGTMTSHHTYELDDTCVVSRITVNCDGFQ
PDGPIMRDQLDILPNETHMFPHGPAVRQLAFIGFTTADGGLMMGHFDSKMTFNRAIEPGP
HFVTIITKQMRDTRDHVCQREVAYAHSVPR
>Branchiostoma_belcheri|GFPe1|144690R
MALPTNHDLHIFGSVNGLEFDMVGGGSGNPKAGTLETSVKSTRGALPFSPLLVAPNLGFY
QYLPFPDG-PSPFQTAITGGYEVNRVFKFEDGGVLSAHFRYTYEGGKIKGEFQLVGSGFP
ASGPVMTSGLSLDRSVAKLMCSDNTLTGLNNWSFCTSDGQRHQAVVQTNYTFAKPFPTMP
VFMGHQIEV-NASKTEIALTEKAKAFCNTV

>Branchiostoma_floridae|GFPe1|63257
MSLPTTHDLHIFGSVNGAEFDLVGGGRGNPNDGTLETSVKSTRGALPCSPLLIGPNLGFY
QYLPFPGG-ASPFQTAITGGYQVHRVFKFEDGGVLNCNFRYTYEGGKIKGEFQLIGSGFP
AGGPVMSGGLTLDRSVAKLQCSDDTITGTNNWSFCTTDGKRHQADVQTNYTFAKPLPLMP
IFLGHQIEV-KASKTEITLSEKVKAFIDTV
>Branchiostoma_floridae|GFPe2|63260
MSLPTAHDLHIFGSVNGAEFDLVGGGKGNPNDGTLETSVKSTRGALPCSPLLIGPNLGFY
QYLPFPGG-ASPFQTAITGGYQVHRVFKFEDGGVLNCNFRYTYEGGKIKGEFQLIGSGFP
AGGPVMSGGLTLDRSVAKLQCSDDTITGTNNWSFCTTDGKRHQADVQTNYIFAKPLPLMP
IFLGHQIEV-KASKTEITLCEKVKAFIDTV
>Branchiostoma_floridae|GFPe3|63258
MSLPTTHDLHIFGSVNGAEFDLVGGGKGNPNDGTLETSVKSTRGALPCSPLLIGPNLGFY
QYLPFPGG-ASPFQTAITGGYQVHRVFKFEDGGVLSCNFRYTYEGGKIKGEFQLIGSGFP
AGGPVMSGGLTLDRSVAKLQCSDDTITGTNNWSFCTTDGKRHQADVQTNYTFAKPLPLMP
IFLGHQIEV-QASKTEINLSEKVKAFIDTV
>Branchiostoma_belcheri|GFPd1|282900F
MSLPTSHDLHIHGSINGLEFDMVGGGSGNPKDGSVETRVKSTKGGLAFSPLIVGPHLGFY
QYLPFPSG-PSPYQAAIKGGYNEHRTMQFEDGAVLTANYRYTYEGGKIKGDFHLVGSGFP
ASGPVMTNSLSLDRSVAKLTSVDDTVVENIDWAYRTSSGDTYRAMVRTNCTFGRPA-VMP
MFVFRQLEV-AGSKTEISLQERQKAFSEVQ
>Asymmetron_lucayanum|GFP1|asym20h_comp64813_c0_seq2_m.13123
MAIPTSHDTHIFGSINGVEFDMVGGGTGNPKDGSVNSTVKSTKGGLPCSPLLIGPHLGLY
QYLPFPDG-PSPFQADM-G-YEVLRTMKFEDGAVLSANYRYSYQGGKIKSEHKLVGSGFP
ADGPVLKNKLCLDRSVTKLKYVDDNLVDTVDWAYSTTDGKKYNAVVQTNYSWGNPSNFMP
AFVFRQIDV-SGSKTEVHLVEKQKAFYDLI
>Branchiostoma_belcheri|GFPd3|005140R
MSLPTSHDLHIHGSINGLEFDMVGGGSGNPKDGSVETRVKSTKGGLQFSPLIVGPHLGFY
QYLPFPSG-PSPYQAAMKGGYKMHRTMQFEDGAVLTANYRYTYEGGKIKGEFHLVGSGFP
ANGPVMTNSLSQDRSVAKLTSVDDTVVENIDWAYRTSDGGSYRANVRTNCTFGRPA-VMP
MFVFRQLEI-SGSKSEISLQERQKVFSEV-
>Branchiostoma_belcheri|GFPd2|005130R
--MPTSHDLHIHGSINGLEFDMVGGGSGNPNDGSVETRVKSTKGGLQFSPLIVGPHLGFY
QYLPFPSG-PSPYQAAMKGGYKMHRTMQFEDGAVLTANYRYTYEGGKIKGDFHLVGSGFP
TNGPVMTNSLSQDRSVAKLTSVDDTVVENIDWAYRTSDGGSYRAMVRTNCTFGRPA-VMP
MFVFRQLEI-SGSKSEISLQERQKVFSEV-
>Branchiostoma_belcheri|GFPd4|145360F
MSFPTSHDLHIHGSINGLEFDMVGGGSGNPKDGSVETRVTSTKGDLAFSPHIVAPHLGFY
QYLPFPGG-PSPYQAAIKGKYNEHRTIQFEDGAVLTANYRYTYKGDKIKGDFHLVGSGFP
ASGPVMTNSMSLDRTVAKITCVDEIVTAIWSFTITTSSGGSYRAMVQTNCTFQKPA-VMP
MFVFRQLEV-KATKTEISLQEKQKAFTEVQ
>Branchiostoma_belcheri|GFPd5|233640F
QSIPTSHDLHIHGSINGLEFDMVGGGSGNPKDGSVESRVTSTKGDLAFSPHIVAPHLGFY
QYLPFPGG-PSPYQAAIDGKYNEHRTIQFEDGAVLTANYRYTYKGDKIKGDFHLVGSGFP
ASGPVMTNSLSLDRTVAKITCVDESLVDNIDWTYRTSSGGSYRAMVQTNCTFQKPV-VMP
MFVFRQLEV-KATKTEISLQEKQKAFTEVQ
>Branchiostoma_floridae|GFPd1|86184

MSVPTNLDLHIYGSINGMEFDMVGGGSGNPNDGSLSVNVKSTKGALRVSPLLVGPHLGHY
QYLPFPDG-PSPFQAAVNGGYQMHRSFNFEDGAVLTATYNYSYSGGKIQGEFHLVGSCFP
DDSPVMTNALGLDRSVAKLMCVSDKLAEFVDWTYRTSSGGRYRATVQTNFTFAKPIALMP
MFVFRQLEV-TGSKTEISLQEQQKAFSTVL
>Branchiostoma_floridae|GFPd2|126982
MSVPTNLDLHIYGSINGMEFDMVGGGSGNPKDGSLAVNVKSTKGALCVSPLLVGPHLGHY
QYLPFPDG-ASPFQAAVNGGYQMHRSFNFEDGAVLTATYNYSYSGGKIQGEFHLVGSGFP
DDSPVMTNALGLDRSVSKLMCTSDKLVESVHWSYRTSSGGRYRATVQTNFTFAKPIELMP
MFVFRQLEV-TGSKTEIGLQEQQKAFSTVL
>Branchiostoma_belcheri|GFPf2|212280R
TSLPTTHDCHIFGTINGHAFDMVGGGSGNPKDGSLQTTVRSTKGPLPFSGVILGPNLGYH
QYLPFPSG-KSPYQNAIKGGYEKHRTFHFEDGGVMSINFRYTYEGNKIKGEFHVVGSGFP
DDGPVMTNSVAHDNNVERLQVLGDTIGSDNVWTFTGKKGDTYNANVMTNATFAQNLQLMP
LFVFRKVDI-QASKTEITLVEREKVFSDLC
>Asymmetron_lucayanum|GFP2|asym20h_comp74545_c2_seq1_m.28460
MSLPTQHDCHIFGSINGIDFDMVGGGKGDPHKGILETTVRSTKGPLPFSPLILGPNLGYH
QYTPFPNG-QSPYQNAVHGGYKKHRTFTFEDGGILSITFNYTYEGNKIYGEFQCVGTGFP
DVGPVMTNALGEHPNCEKLMISGASIGSDNNWSFSTNKGKYYKANVVTNSTFAKKLPLMP
LFVFRKTQV-KCTKTEITLHESEKSFSDL-
>Branchiostoma_belcheri|GFPf1|144780R
MSLPTTHDCHIFGTINGHAFDMVGGGSGNPKDGSLQTTVRSTKGPLPFSGVILGPNLGYH
QYLPFPSG-MSPYQNAIKGGYEKHRTFQFEDGGVMSINFRYSYEGNKIKGEFHVVGSGFP
DDGPVMTNSLAHDNNVERLQVLGDTIGSDNVWTYTGKKGGTYKANVMTNATFAQNLQLMP
LFVFRKVDI-EASKTEITLVEREKVFSDLC
>Branchiostoma_floridae|GFPf1|63259
MSLPTAHDCHMFGSINGHEFDLVGGGNGNPNDGTLETKVRSTKGALPFSPVILAPNLGYH
QYLPFPAG-TSPYQQAITGVYQKHRTFKFEDGGVMTINFRYTYSGNKIKGEFHVVGSGFP
DDGPVMTNSLQHDHNVERLMVLGDTIGSDNMWTFTEKGGKDYKAEVMTNATFAQNLQLMP
LFVFRKVDI-DCSKTEVTLIEREKVFQDLL
>Branchiostoma_floridae|GFPa1|63256
MPLPATHDIHLHGSINGHEFDMVGGGKGDPNAGSLVTTAKSTKGALKFSPYLMIPHLGYY
QYLPYPDGPSPFQTSMLESGYAVYRVFDFEDGGKLTTEFKYSYEGSHIKADMKLMGSGFP
DDGPVMTSQIDQDGCVSKKTYLNNTIVDSFDWSYNLQNGKRYRARVSSHYIFDKPFSLQP
VFVYRKCHV-KASKTEVTLDEREKAFYELA
>Branchiostoma_floridae|GFPa2|63262
MPLPATHEIHLHGSVNGHEFDLVGSGKGDPKAGSLVTEVKSTMGPLKFSPHLMIPHLGYY
QYLPYPDGPSPFQTAMLDSGYKVHRVFNFEDGGVLSIDYNYAYEGTHIKSDFKLMGSGFP
DDGPVMTSQIDQDGCVSKKTYLNDTIVDSFDWSYNLQNGKRYRARVTSNYIFGKPLAVQP
VFVYRKCYV-KSTQTEITLDEREKAFYEVV
>Branchiostoma_lanceolatum|FPO1|gi|169125804|gb|EU482393.1|
MPLPATHELHIFGSFNGVEFDMVGRGTGNPNDGSEDLHLKSTKGALQFSPWILIPHIGFH
QYLPFPDGMSPFQAAMQDSGYQVHRTMQFEDGASLTANFRYTYEGSHIKGEFQVIGSGFP
ADGPVMTNSLAVDWCVAKMLYPNDTIISTFDWTYTTGSGKRYQSTVRTNYTFAKPMAIQP
MFVFRKTEL-KHSKTELNFKEWQKAFADVM
>Branchiostoma_belcheri|GFPb3|240030F
LTIYATHEIHIYGSFNGVEFDMVGRGTGNPKDGSEELHMKSTKGPLPFSAQILIPHVGYH

QYLPYPDGMSPFQAAMQDSGYQVTRIMQFEDGANVTGHYRYTYEGSHIKGDMQVIGTGFP
ADGPVMTKSLAVDWCVAKNVHPDNTIKATFDWTLTTTSGKRYQASARTNYTFAKPMAFEP
MFVFRKTAI-QGNDTEFAFKEEQKAFADLM
>Branchiostoma_floridae|GFPb1|75522
MPLPKTHELHIFGSFNGVEFDMVGRGIGNPNEGSEELNAKFTKGPLKFSPYILVPHLAYY
QYLPFPDGMSPFQAAMHDSGYQVHRTIQYEDGASVTAHYRYTYEGSHIKGEFQVIGTGFP
PDGPVMTNKLAMDWSVTKMLYPNDTILSTVDCSYTTTEGKRYQSKMRENNTFAKPMAIQP
MFVFRKTEL-QHSKTELTFKEWQKAFTDVM
>Branchiostoma_floridae|GFPb2|75521
MPLPKTHELHIFGSFNGVEFDMVGEGTGNPNEGSEELKLKSTNGPLKFSPYILVPHLAFN
QYLPFPDGMSPFQAAMQDSGYQVHRTLQYEDGAFVTANLRYTYEGSHIKGEFQVIGTGFP
PDGPVMTNKLAMDWSVVKFVYPNDTILSTFDKTYTTTEGKRYQCTFRENSTFAKPMAIQP
MFIFHKTEL-QHSNAELTFKEKQTAFSDMK
>Branchiostoma_floridae|GFPb3|75519
MPLPTTHELHIFGTFNGVEYDMVGRGKGNPNDGYEELNLKSTKGPLKFSPWILVPQIGFH
QYLPYPDGMSPFQAAMHDSGYQVHRTLDFEDGATLTADFRYTYEGSHIKGEFKVIGTGFP
ADGPVMTNKLAADWCVNKMLYPDDTINSTFDWSYTTSEGKRYQSTVRENYTFAKPMAIQP
MFVFRKTEL-QHSKTELTFKEWQKAFTDVM
>Branchiostoma_floridae|GFPb4|75520
MPLPTTHELHIFGSFNGVEFDLVGRGEGNPKDGSQNLHLKSTKGPLQFSPWMLIPHIGFY
QYLPYPDGESPYQAAMYGSGYLMHRTMQYEDGAKISGHYKYTYEGSHVKGEFQLIGTGFP
TDGPVMTNQLAADWCVDKLLYPNDTIISKFDWSYTTTDGKRYQAKVQTNFDFAKPMAYQP
MFVFRKVEL-EHSKTEVKFKQWQKAFHDIM
>Branchiostoma_lanceolatum|FPX6_partial|gi|169125832|gb|EU482407.1|
MSLPATHELHIFGKINGHEFDMRGKGTGNPNDGYEDLDLKSTKDDLPFSPWILVQNIGFN
QYLPYPDGASPFQAAMYNSGYHVHREMEFEDGATLTGIYRYTYEGSHIKGEFQVDGTGFP
ADGPVMTDSLDLDWVVTKMVYPDDTVFSTSDQTYTTTSGKGYQSTVRTNNIFAEPIAMQP
VFVSRK------------------------
>Branchiostoma_lanceolatum|FPX5_partial|gi|169125830|gb|EU482406.1|
MSLPATHELHIFGKINGHEYDMRGKGTGNPNDGYEDLDLKSTKDDLPFSPWILVQNIGFN
QYLPYPDGASPFQAAMCDSGYEVHREMEFEDGATVTGIYRYTYEGSHIKGEFQVDGTGFP
DDGPVMTDSLDLDWVVTKMVYPDETVFSTSDQTYTTTSGKGYKSTVRTNNIFAKPIAMQP
VFVSRK------------------------
>Branchiostoma_lanceolatum|FPX4_partial|gi|169125828|gb|EU482405.1|
MSLPKTHELHIFGKINGHEYDMRGKGTGNPNDGYEDLDLKSK-DDLPFSPWILVQNIGFN
QYLPYPDGASPFQAAMCDSGYEVHREMEFEDGATLTGIYRYTYEGSHIKGEFQVDGTGFP
DDGPVMTDSLDLDWVVTKMVYPDETVFSTSDQTYTTASGKGYKSTVRTNNIFAKPMAMQP
IFVSRK------------------------
>Branchiostoma_lanceolatum|FPX3_partial|gi|169125826|gb|EU482404.1|
MSLPATHELHIFDKINGHEFDMRGKGTGNPNDGYEDLDLKSTKDDLPFSPWILVQNIGFN
QYLPYPDGASPFQAAMYNSGYHVHREMGFEDGATVTGIYRYTYEGSHIKGEFQVDGTGFP
ADGPVMTNSLDQDWSVTKMMYLDNTVTSTADQTYTTASGKRYQGTVRTNNTFAKPIAIQP
VFVSRK------------------------
>Branchiostoma_lanceolatum|FPY6|gi|169125802|gb|EU482392.1|
MSLPATHELHIFGSINSLEFDLVGRGTGNPKEGYEELHLKSTKSALQFSPWILVPQIGFY
QYLPFPDGASPFQAAMNDSGYQVHRTMQFEDGATLTGIYRYTYEGTHIKGEFQVIGTGFP

ADGPVMTNSLAADWCVTKIVYPNETIIDKFDWTYTTTSGKRYQSNVRSNFTFAKPIAIQP
MFVFRKTEL-KHSKTELNFKEWQTAFSDVM
>Branchiostoma_lanceolatum|FPY2|gi|169125794|gb|EU482388.1|
MSLPATHELHIFGSINSLEFDLVGRGTGNPREGYEELHLKSTKSALQFSPWILVPQIGFY
QYLPFPDGASPFQAAMNDSGYQVHRTMQFEHGATLTGIYRYTYEGTHIKGEFQVIGTGFP
ADGPVMTNSLAADWCVTKIVYPNETIIDKFDWTYTTTSGKRYHSNVRSNFTFAKPIAIQP
MFVFRKTEL-KHSKTELNFKEWQTAFGDVM
>Branchiostoma_lanceolatum|FPY1|gi|169125792|gb|EU482387.1|
MPLPATHELHIFGSINSLEFDLVGRGTGNPKEGYEELHLKSTKSALQFSPWILVPQIGFY
QYLPFPDGASPFQAAMNDSGYQVHRTMQFEDGATLTGIYRYTYEGTHIKGEFQVIGTGFP
ADGPVMTNSLAADWCVTKIVYPNETIIDKFDWTYTTTSGKRYQSNVRSNFTFAKPIAIQP
MFVFRKTEL-KHSKTELNFKEWQTAFSDVM
>Branchiostoma_lanceolatum|FPY5|gi|169125800|gb|EU482391.1|
MPLPATHELHIFGSFNGVDFDMVGHGTGNPNDGYEELNLKSTKGALQFSPWILVPQIGFH
QYLPFPDGMSPFQAAMKDSGYQVHRTMQFEDGASLTSNYRYTYEGSHIKGEFQVNGTGFP
ADGPVMTNSLTADWCVTKMLYPNDTIISTFDWTYTTGSGKRYQSTVRTNYTFAKPMAIQP
MFVFRKTEL-KHSKTELNFKEWQKAFADVM
>Branchiostoma_lanceolatum|FPY4|gi|169125798|gb|EU482390.1|
MPLPATHELHIFGSFNGVDFDMVGRGTGNPNDGYEELNLKSTKGALQFSPWILVPQIGFH
QYLPFPDGMSPFQAAMKDSGYQVHRTMQFEDGASLTSNYRYTYEGSHIKGEFQVIGTGFP
ADGPVMTNSLTADWCVTKMLYPNDTIISTFDWTYNTASGKRYQSTVRTNYTFNKPMAIQP
MFVFRKTEL-KHSKTELNFKEWQMAFADVM
>Branchiostoma_lanceolatum|FPY3|gi|169125796|gb|EU482389.1|
MSLPATHELHIFGSFNGVDFDMVGRGTGNPNDGYEELNLKSTKGALQFSPWILVPQIGFH
QYLPFPDGMSPFQAAMKDSGYQVHRTMQFEDGASLTSNYRYTYEGSHIKGEFQVIGTGFP
ADGPVMTNSLAADWCVTKMLYPNDTIISTFDWTYTTGSGKRYQSTVRTNYTFAKPMAIQP
MFVFRKTEL-KHSKTELNFKEWQKAFTDVM
>Branchiostoma_belcheri|GFPc1|199320R
QPVPATHELHVSGSINGREFDLAGRGTGNAKDGSEEIQVKSTKGALGFSPVLLVPNLGFH
QYLPYPD-GMSPFQAAADGGYVVHRTLQFEDGGSVTGIYRYSYDGSHIKGEFNVTGSGFP
ADGPVMTNSLAVDPSVATVFCPNDTVVSTIDWSCTTTSGKRYHGTVRTNYTFAKPIAFQP
MFVFRKTEL-KASDTELSLKESQKAFHGL-
>Branchiostoma_belcheri|GFPb1|233630F
----------------------------------MECPTTRKPALFSPYVLAPHFGYD
QYLPFPDQGVSPFQATMQSGYQVIRTVHFEDGAILSAHFRYTYEGSHIKGEFQVIGGGFP
ADGPVMTKSLAMDWSVAKLVSPNDTVQSTIDMAYTTTSGKRYQSTVRNIHTFAKPMTIKP
VFVFRKTDL-KANKTEVTFKEWQKAFTDVM
>Branchiostoma_belcheri|GFPb2|264830F
KKKLASHELHIFGSINGVEFDMVGHGTGNPKDGSQELQVKSTKGPLPFSPYVLAPHFGYD
QYLPFPDQGVSPFQATMQSSG--------------------------------------

>Branchiostoma_belcheri|GFPx1|276530F_m
YPLPATHELHIYGSINGHEFDMVGGGDGNPKDGHMQTNVKSTKGPLAFSPYIVTPHLGFY
QYLPFPNGEMSPFQAAMQGGYEIHRTFRFEDGAMLSANYKYFFDGTHIKGEFCLTGSGFP
ANGPVMTSAIGVDDTVSRMVPLDNMLFDAFSWGYRTSSGKTYEAMVNTNYQFGKPIPILP

MFVFRKLDI-KVSKTEVTLVEWQKAFSEPH
>Branchiostoma_floridae|GFPc1|75523
MPLPTTHEVHVYGSINGVEFDLVGSGKGNPKDGSEEIQVKSTKGPLGFSPYIVVPNIGFH
QYLPFPDG-MSPFQAAADGGYVVHRNIQFEDGASLTGIYRYSYDAGHIKGEFRVVGSGFP
ADGPVMTKSLAVDWSVATMLFPNDTVVSTIDWTCPTTSGKRYHATVRTNYTFAKPIAIQP
MFVFRKTEV-KASDSEINLKESQKAFHDLV
>Branchiostoma_lanceolatum|FPR5|gi|169125816|gb|EU482399.1|
MPLPATHDLHISGSINGHEFDLEGSGKGNAKEGYQELHLKSNKGDLSFSPWILVPNIGFY
QYLPFPDGAMSPYQAAMHGGYVMHRSMQFEDGAMLHSDHRYIYKGNHIKGEFRLTGSGFP
ADGPVMTNSLAADWCVDKLLYPNDTIIGKFDWTYTTTSGKRYQSDVQTNVTFGKPIAIQP
MFVFRKVEL-KHTKTELNFKQWQKAFQDIA
>Branchiostoma_lanceolatum|FPR4|gi|169125814|gb|EU482398.1|
MSLPATHDLHISGSINGHEFDLEGSGKGNAKEGYQELHLKSNKGDLSFSPWILVPNIGFY
QYLPFPDGAMSPYQAAMHGGYVMHRTMQFEDGAMLHSDHRYTYKGNHIKGEFRLTGSGFP
ADGPVMTNSLAVDWCVDKLLYPNETIIGKFDWTYTTTSGKRYQSDVQTNVTFAKPIAIQP
MFVFRKVEL-KHSKTELNFKQWQKAFQDIV
>Branchiostoma_lanceolatum|FPR2|gi|169125810|gb|EU482396.1|
MSLPATHDLHISGSINGHEFDLEGSGKGNAKEGYQELHLKSNKGDLSFSPWILVPNIGFY
QYLPFPDGAMSPYQAAMHGGYVMHRSMQFEDGAMLHSDHRYIYKGNHIKGEFRLTGSGFP
ADGPVMTNSLAADWCVDKLLYPNDTIIGKFDWTYTTTSGKRYQSDVQTNVTFGKPIAIQP
MFVFRKVEL-KHTKTELNFKQWQKAFQDIA
>Branchiostoma_lanceolatum|FPR1|gi|169125808|gb|EU482395.1|
MPLPATHDLHISGSINGHEFDLEGSGKGNAKEGYQELHLKSNRGDLSFSPWILVPNIGFY
QYLPFPDGAMSPYQAAMHGGYVMHRTMQFEDGAMLHSDHRYTYKGNHIKGEFRLTGSGFP
ADGPVMTNSLAADWCVDKLLYPTETLIGKFDWTYTTTSGKRYQSDVQTNVTFAKPMAIQP
MFVFRKVEL-KHTKTELNFKQWQKAFQDIV
>Branchiostoma_lanceolatum|FPO2|gi|169125806|gb|EU482394.1|
MPLPATHDLHISGSINGHEFDLEGSGKGNEKEGYQELHLKSNKGDLSFSPWILVPNTGFY
QYLPFPDGAMSPYQAAMHGGYVMHRSMQFEDGAMLHSDHRYIYKGNHIKGEFRLTGSGFP
ADGPVMTNSLAADWCVDKLLYPNDTIIGKFDWTYTTTSGKRYQSDVQTNVTFGKPIAIQP
MFVFRMMEL-KHTKTELNFKQWQKAFQDIA
>Branchiostoma_lanceolatum|FPR9|gi|169125824|gb|EU482403.1|
MSLPKTHDLHISGSVNGHEFDLEGSGKGDAKEGYQELHLKSNRGDLSFSPWILVPNIGFY
QYLPFPDGAMSPYQAAMHGGYVMHRAMRFEDGAMLHSDHRYTYNGNNIKGEFRLTGSGFP
ADGPVMTNSLAADWCVDKLLYPNETIIGKFDWTYTTTSGKRYQSDVQTNVTFGKPISIQP
MFVFRKVEL-KHSKTELNFKQWQKAFQDIV
>Branchiostoma_lanceolatum|FPR8|FPR8|gi|169125822|gb|EU482402.1|
MSLPATHDLHISGSINGHEFDLEGSGKGNAKEGYQELHLKSNKGDLSFSPWILVPNIGFY
QYLPFPDGAMSPYQAAMHGGYVMHRAMRFEDGAMLHSDHRYTYNGNHIKGEFRLTGSGFP
ADGPVMTNSLAADWCVDKLLYPDETIIGKFDWTYTTTSGKRYQSDVQTNVTFAKPISIQP
MFVFRKVEL-KHSKTELNFKQWQKAFQDIV
>Branchiostoma_lanceolatum|FPR7|gi|169125820|gb|EU482401.1|
MPLPATHDLHISGSINGHEFDLEGSGKGNAKEGYQELHLKSNKGDLSFSPWILVPNIGFY
QYLPFPDGAMSPYQAAMHGGYVMHRAMRFEDGAMLHSDHRYTYNGNHIKGEFRLTGSGFP
ADGPVMTNSLAADWCVDKLLYPDETIIGKFDWTYTTTSGKRYQSDVQTNVTFAKPISIQP
MFVFRKVEL-KHSKTELNFKQWQKAFQDIV

>Branchiostoma_lanceolatum|FPR6|gi|169125818|gb|EU482400.1|
MSLPKTHDLHISGSVNGHEFDLEGSGKGNAKEGYQELHLKSNRGDLSFSPWILVPNIGFY
QYLPFPDGAMSPYQAAMHGGYVMHRAMRFEDGAMLHSDHRYTYNGNNIKGEFRLTGSGFP
ADGPVMTNSLAADWCVDKLLYPNETIIGKFDWTYTTTSGKRYQSDVQTNVTFGKPISIQP
MFVFRKVEL-KHSKTELNFKQWQKAFQDIV
>Branchiostoma_lanceolatum|FPR3_partial|gi|169125812|gb|EU482397.1|
MPLPATHDLHISGSINGHEFDLEGSGKGNAKEGYQELHLKSNRGDLSFSPWILVPNIGYY
QYLPSPG---

>gi|114793939|pdb|2G3O|A|Pontellina_plumata|Copepod
-MPAMKIECRITGTLNGVEFELVGGGEGTPEQGRMTNKMKSTKGALTFSPYLLSHVMXFY
HFGTYPSGYENPFLHAINGGYTNTRIEKYEDGGVLHVSFSYRYEAGRVIGDFKVVGTGFP
EDSVIFTDKIRSNATVEHLHPMGDVLVGSFARTFSLRDGGYYSFVVDSHMHFKAIHPLGP
MFAFRRVEE-LHSNTELGIVEYQHAFKTPI
>gi|33243026|gb|AAQ01183.1||Pontellina_plumata|Copepod
-MPAMKIECRISGTLNGVVFELVGGGEGIPEQGRMTNKMKSTKGALTFSPYLLSHVMGFY
HFGTYPSGYENPFLHAANGGYTNTRIEKYEDGGVLHVSFSYRYEAGRVIGDFKVVGTGFP
EDSVIFTDKIRSNATVEHLHPMGDVLVGSFARTFSLRDGGYYSFVVDSHMHFKAIHPLGS
MFAFRRVEE-LHSNTELGIVEYQHAFKTPT
>gi|33243028|gb|AAQ01184.1||Pontellina_plumata|Copepod
-MPAMKIECRITGTLNGVEFELVGGGEGTPEQGRMTNKMKSTKGALTFSPYLLSHVMGFY
HFGTYPSGYENPFLHAINGGYTNTRIEKYEDGGVLHVSFSYRYEAGRVIGDFKVVGTGFP
EDSVIFTDKIRSNATVEHLHPMGDVLVGSFARTFSLRDGGYYSFVVDSHMHFKAIHPLGP
MFAFRRVEE-LHSNTELGIVEYQHAFKTPI
>gi|33243036|gb|AAQ01188.1||Pontellidae_sp._SL-2003|Copepod
-MAAMKIECRITGTMNGVEFELVGGGEGNTDQGRMTNKMKSTKGPLSFSPYLLSHVMGFY
HFGTFPSGYENPYVHAMTGGYTNTRIESYEDGGVLYLTFNYRLDGNKIIGDFKCVGTGFP
EDSVIFTDKIKSNPNCEHFYPMAEIMKNAYMRTLSLRDGGYYSGQVTSHIHFKAIHPLGS
MFTYRRVEE-LHTQTDLGIVEYQHVFKTPT
>gi|93279890|pdb|2G6X|A|Pontellina_plumata|Copepod
-LPAMEIECRITGTLNGVEFELVGGGEGTPEQGRMTNKMKSTKGALTFSPYLLSHVMXFY
HFGTYPSGYENPFLHAINGGYTNTRIEKYEDGGVLHVSFSYRYEAGRVIGDFKVMGTGFP
EDSVIFTDKIRSNATVEHLHPMGDDLDGSFTRTFSLRDGGYYSSVVDSHMHFKAIHPLGP
MFAFRRVEE-DHSNTELGIVEYQHAFKTPD
>gi|33243032|gb|AAQ01186.1||Pontella_meadi|Copepod
-MPDMKLECHISGTMNGEEFELIGAGDGNTDEGRMTNKMKSIKGPISFSPYLLSHILGYY
HFATFPAGYENIYLHAMKGGYSNVRTERYEDGGIISITFNYRYEGNKIIGDFKVVGTGFP
TNSLIFTDKIKSNPTCENMFPKADTLVNAYTRTYLLKDGGYYSAQVNNHMHFKAIHTLGS
MFTYRVVEE-THTQNEVAIVEYQNVFKTPT
>gi|126030215|pdb|2DD7|A|Chiridius_poppei|Copepod
--TTFKIESRIHGNLNGEKFELVGGGVGEE--GRLEIEMKTKDKPLAFSPFLLSHCMXFY
HFASFPKGTKNIYLHAATGGYTNTRKEIYEDGGILEVNFRYTYEFNKIIGDVECIGHGFP
SQSPIFKDTIKSCPTVDLMLPMSGIIASSYARAFQLKDGSFYTAEVKNNIDFKNPIHFGP
MFTHRRVEE-THTKENLAMVEYQQVFNSAP
>gi|126030217|pdb|2DD9|A|Chiridius_poppei|Copepod

--TTFKIESRIHGNLNGEKFELVGGGVGEE--GRLEIEMKTKDKPLAFSPFLLSTCMXFY
HFASFPKGTKNIYLHAATGGYTNTRKEIYEDGGILEVNFRYTYEFNKIIGDVECIGHGFP
SQSPIFKDTIKSCPTVDLMLPMSGIIASSYARAFQLKDGSFYTAEVKNNIDFKNPIHFGP
MFTHRRVEE-THTKENLAMVEYQQVFNSAP
>gi|85658701|dbj|BAE78442.1||Chiridius_poppei|Copepod
-MTTFKIESRIHGNLNGEKFELVGGGVGEE--GRLEIEMKTKDKPLAFSPFLLSHCMGFY
HFASFPKGTKNIYLHAATGGYTNTRKEIYEDGGILEVNFRYTYEFNKIIGDVECIGHGFP
SQSPIFKDTIKSCPTVDLMLPMSGIIASSYARAFQLKDGSFYTAEVKNNIDFKNPIHFGP
MFTHRRVEE-THTKENLAMVEYQQVFNSAP
>gi|33243030|gb|AAQ01185.1||Labidocera_aestiva|Copepod
-MPVMKIECRISGTMNGEEFELVGAGDGNTDEGRMTNKMKSTKGPLSFSPYLLSHIMGFY
HYATFPAGYENVYLHAAKGGYTNTRTERYEDGGIISVNFTYRYEGNKVIGDFKVVGSGFP
ANSVIFTDKIKSNPTCEHIYPKGDILVNAYTRTWMLRDGGYYSAQVNNHLHFKAMHPLGS
MFTYRKVEE-LHSQSDVGIVEYQHVFKTPT
>gi|33243034|gb|AAQ01187.1||Pontella_meadi|Copepod
-MPDMKLECHISGTMNGEEFELIGSGDGNTDQGRMTNNMKSIKGPLSFSPYLLSHILGYY
HFATFPAGYENIYLHAMKGGYSNVRTERYEDGGIISITFNYRYEGSKIIGDFKVIGTGFP
TDSLIFTDKIKSNPTCENMFPKADILVNAYTRTYLLKDGGYYSAQVNNHMHFKAIHPLGS
MFTHRVVEE-NHTKTNVAIVEYQNVFKTPT
>gi|255032500|gb|ACT99046.1||Pontella_mimocerami|Copepod
-MPNMKLECRISGTMNGEEFELVGNGDGNTDEGRMTNKMKSTKGPLSFSPYLLSHVLGYY
HYATFPAGYENVYLHAMKGGYSNTRTERYEDGGIISATFNYRYEGDKIIGDFKVVGTGFP
TNSIIFTDKIKSNPTCEHIYPKADILVNAYTRTWMLRDGGYYSAQVNNHMHFKAIHPLGS
MFTYRKVEE-LHTQTEVGIVEYQHVFKTPT
>gi|255032502|gb|ACT99047.1||Pontella_mimocerami|Copepod
-MPNMKLECRISGTMNGEEFKLVGAGEGNTDEGRMTNKVKSTKGPLPFSPYLLSHVLGYY
HYATFPAGYENVYLHAMKGGYSNTRTERYEDGGIISATFNYRYEGDKIIGDFKVVGTGFP
TNSIIFTDKIKSNPTCEHIYPKADILVNAYTRTWMLRDGGYYSAQVNNHMHFKAIHPLGS
MFTYRKVEE-LHTQTEVGIVEYQHVFKRPT
>gi|255032504|gb|ACT99048.1||Pontella_mimocerami|Copepod
-MPNMTLECRISGTMNGEEFELVGNGDGNTDEGRMTNKMKSTKGPLSFSPYLLSHVLGYY
HYATFPAGYENVYLHAMKGGYSNTRTERYEDGGIISATFNYRYEGDKIIGDFKVVGTGFP
TNSIIFTDKIKSNPTCEHIYPKADILVNAYTRTWMLRDGGYYSAQVNNHMHFKAIHPLGS
MFTYRKVEE-LHTQTEVGIVEYQHVFKTPT
>gi|255032506|gb|ACT99049.1||Pontella_mimocerami|Copepod
-MPNMELECRISGTMNGEEFELVGNGDGNTDEGRMTNKMKSTKGPLSFSPYLLSHVLGYY
HYATFPAGYENVYLHAMKGGYSNTRTERYEDGGIISATFNYRYEGDKIIGDFKVVGTGFP
TNSIIFTDKIKSNPTCEHIYPKADILVNAYTRTWMLRDGGYYSAQVNNHMHFKAIHPLGS
MFTYRKVEE-LHTQTEVGIVEYQHVFKTPT
>gi|255032508|gb|ACT99050.1||Pontella_mimocerami|Copepod
-MPNMTLESAISGTMNGEEFELVGNGDGNTDEGRMTNKMKSTKGPLSFSPYLLSHVLGYY
HYATFPAGYENVYLHAMKGGYSNTRTERYEDGGIISATFNYRYEGDKIIGDFKVVGTGFP
TNSIIFTDKIKSNPTCEHIYPKADILVNAYTRTWMLRDGGYYSAQVNNHMHFKAIHPLGS
MFTYRKVEE-LHTQTEVGIVEYQHVFKTPT
>gi|125395854|gb|ABN41776.1||Lobophyllia_hemprichii|Cnidarian-Anthozoa
IKSVMNVKLRLEGAVNGHPFVIEGKGNGHPFEGTQDIKLTVKGGPLPFAYDILTTVFGNR

VFVKYPDDIVDYFKLSFPEGYSWERSMVYEDGGVCLATSDIKLLKDEFFHKIRFDGVNFP
ANSPVMLKTTKWEPSTEKMYARDGVVKGDVNMALLLKGGGHYRCDFKTTYKAKKYVPPDY
HFVDHRIEITSHDYNIVNVFEDAVAHSGLT
>gi|154814320|gb|ABS87211.1||Montastraea_cavernosa|Cnidarian-Anthozoa
IKTDMKIKLRMEGAVNGHKFVIEGEGEGKPFDGKQTMDLTVIGAPLPFAYDILTTVFGNR
VFAKYPKDIPDYFKQTFPEGYSWERSMTFEDQGICTVTSDIKLEGDCFFYEIRFYGVNFP
SSGPVMQKKTKWEPSTEIMYVRDGVLKGDVNMALLLEGGGHYRCDFKTTYKAKKFVQPDY
HFVDHRIEIVSHDYNKVKLYEHAEAHSGLP
>gi|154814324|gb|ABS87213.1||Montastraea_cavernosa|Cnidarian-Anthozoa
IKPDMKIKLRMEGAVNGHKFVIEGDGKGKPFEGKQTMDLTVIGAPLPFAYDILTTVFGNR
VFAKYPKDIPDYFKQSFPEGYSWERSMTFEDQGVCTVTSDIKLEGDCFFYEIRFYGVNFP
SSGPVMQKKTKWEPSTENMYVRDGVLLGDVNMALLLEGGGHYRCDFKTTYKAKKVVQPDY
HFVDHRIEIVSHDYNKVKLYEHAEAHSGLP
>gi|15081472|gb|AAK83923.1|AF401282_1|Montastraea_faveolata|Cnidarian-Anthozoa
IKPDMKIKLRMEGAVNGHKFVIEGDGKGKPFEGTQSMDLTVKGAPLPFAYDILTTVFGNR
VFAKYPQDIPDYFKQTFPEGYSWERSMTYEDQGICVATNDITLMKGVFVYKIRFDGVNFP
ANGPVMQKKTKWEPSTEKMYVRDGVLKGDVNMALLLEGGGHYRCDFKTTYKAKKFVQPDY
HFVDHRIEILSHDYNKVKLYEHAEAHSGLP
>gi|154814314|gb|ABS87208.1||Montastraea_cavernosa|Cnidarian-Anthozoa
IKTDMKIKLRMEGAVNGHKFVIEGEGEGKPFDGKQTMDLTVIGAPLPFAYDILTTVFGNR
VFAKYPKDIPDYFKQTFPEGYSWERSMTYEDQGICIATNDITMMKGVFVYKIRFDGVNFP
ANGPVMQRKTKWEPSTEIMYVRDGVLKGDVNMALLLEGGGHYRCDFKTTYKAKKVVRPDY
HFVDHRIEIVSHDYNKVKLHEHAEAHSGLS
>gi|154814322|gb|ABS87212.1||Montastraea_cavernosa|Cnidarian-Anthozoa
IKPDMKIKLRMEGAVNGHKFVIEGDGKGKPFEGKQTMDLTVIGAPLPFAYDILTTVFGNR
VFAKYPKDIPDYFKQTFPEGYSWERSMTYEDQGICIATNDITMMKGVFVYKIRFDGVNFP
ANGPVMQRKTKWEPSTEKMYVRDGVLKGDVNMALLLEGGGHYRCDFKTTYKAKKVVQPDY
HFVDHRIEIVSHDYNKVKLYEHAEAHSGLP
>gi|154814326|gb|ABS87214.1||Montastraea_cavernosa|Cnidarian-Anthozoa
IKPDMKIKLRMEGAVNGHKFVIEGDGKGKPFDGKQTMDLTVIGAPLPFAYDILTTVFGNR
VFAKYPKDIPDYFKQTFPEGYSWERSMTYEDQGICIATNDITMMKGVFLYKIRFDGVNFP
SSGPVMQKKTKWEPSTENMYVRDGVLLGDVNMALLLEGGGHYRCDFKTTYKAKKVVQPDY
HFVDHRIEIVSHDYNKVKLYEHAEAHSGLP
>gi|32188172|gb|AAO61601.1||Montastraea_cavernosa|Cnidarian-Anthozoa
IKPDMKIKLRMEGAVNGHKFVIEGDGKGKPFEGKQTMDLTVIGAPLPFAYDILTTVFGNR
VFAKYPKDIPDYFKQTFPEGYSWERSMTYEDQGICIATNDITMMKGVFLYKIRFDGVNFP
ANGPVMQRKTKWEPSTEKMYVRDGVLKGDVNMALLLEGGGHYRCDFKTTYKAKKVVQPDY
HFVDHRIEIVSHDYNKVKLYEHAEAHSGLP
>gi|51472051|gb|AAU04446.1||Montastraea_cavernosa|Cnidarian-Anthozoa
IKPDMKIKLRMEGAVNGHKFVIEGDGKGKPFEGKQTMDLTVIGAPLPFAYDILTTVFGNR
VFAKYPKDIPDYFKQTFPEGYSWERSMTYEDQGICIATNDITMMKGVFVYKIRFDGVNFP
ANGPVMQRKTKWEPSTEKMYVRDGVLKGDVNMALLLEGGGHYRCDFKTTYKAKKVVQPDY
HFVDHRIEIVSPDYNKVKLYEHAEAHFGLP
>gi|51472053|gb|AAU04447.1||Montastraea_cavernosa|Cnidarian-Anthozoa
IKPDMKIKLRMEGAVNGHKFVIEGDGKGKPFEGKQTMDLTVIGAPLPFAYDILTTVFGNR
VFAKYPKDIPDYFKQTFPEGYSWERSMTYEDQGICIATNDITMMKGVFVYKIRFDGVNFP

ANGPVMQRKTKWEPSTEKMYVRDGVLKGDVNMALLLEGGGHYRCDSKTTYKAKKVVQPDY
HFVDHRIEIGSPDYNKVKLYEHAEAHFGLP
>gi|51472057|gb|AAU04449.1||Montastraea_faveolata|Cnidarian-Anthozoa
IKPDMKIKLRMEGAVNGHKFVIEGDGKGKPFEGKQTMDLTVIGAPLPFAYDILTTVFGNR
VFAKYPKDIPDYFKQTFPEGYSWERSMTYEDQGICIATNDITMMKGVFVYKIRFDGVNFP
ANGPVMQRKTKWEPSTEKMYVRDGVLKGDVNMALLLEGGGHYRCDSKTTYKAKKVVQPDY
HFVDHRIEIVSHDYNKVKLYEHAEAHSGLP
>gi|32188176|gb|AAO61603.1||Montastraea_cavernosa|Cnidarian-Anthozoa
IKPDMKIKLRMEGAVNGHNFVIEGEGKGKPFEGTQTINLTVKGGPLPFAYDILTAAFGNR
AFTKYPRDIADYFKQSFPEGYSWERSMTYEDQGICIIKSDIRMEGDCFIYEIRYDGVNFP
PSGPVMQKKTKWEPSTEKMYVRDGVLKGDVNMALLLEGGGHYRCDFRSTYKAKKRVQPDY
HFVDHRIEILSHDYNTVKLSENAEARYSML
>gi|51472055|gb|AAU04448.1||Montastraea_cavernosa|Cnidarian-Anthozoa
IKPDMKIKLRMEGAVNGHNFVIEGEGKGKPFEGTQTINLTVKGGPLPFAYDILTAAFGNR
AFTKYPRDIADYFKQSFPEGYSWERSMTYEDQGICIIKSDIRMEGDCFIYEIRYDGVNFP
PSGPVMQKKTKWEPSTEKMYVRDGVLKGEVNMALLLEGGGHYRCDFRSTYKAKKRVQPDY
HFVDHRIEILSHDYNTVKLSENAEARYSML
>gi|149241732|pdb|2GW4|A|Trachyphyllia_geoffroyi|Cnidarian-Anthozoa
IKPEMKIKLLMEGNVNGHQFVIEGDGKGHPFEGKQSMDLVVKGAPLPFAYDILTTAX---

>gi|154814328|gb|ABS87215.1||Montastraea_cavernosa|Cnidarian-Anthozoa
IKSVMKIKLRMEGAVNGHNFTIVGEGEGKPYEGTQSMDLTVVGGPLPFAYDILTTVFGNR
VFAKYPKDIPDYFKQTFPEGYSWERSMTYEDGGICTATNAIKMEGDCFIYKIRFDGVNFP
HKSPVMQKKTKWEPSTEKMYVRDGVLKGDISMALLLEGGGHYRCDFKTSYKAKEGVEPDY
HFVDHRIEILRHDYTEVKLCEHAEAHSGLP
>gi|257221237|gb|ACV52384.1||Paragoniastrea_australensis|Cnidarian-Anthozoa
---GTRLKYLWFANV-------------KDSMGKQTMDLTVIGAPLPFAFDILTTVFGNR
VFAKYPKEIPDYFKQSFPEGYSWQRSMTYEDGGVCQASNDIKIKEDDFVYEIRFDGVNFP
XXXPVMQKKTKWEPSTEKMYVRDGXLKGDVNMALLLQGGGHYRCDFKTTYKAKKAVQP--

>gi|62732002|dbj|BAD95669.1||Favia_favus|Cnidarian-Anthozoa
ITSEMKIELRMEGAVNGHKFVITGKGSGQPFEGIQNVDLTVIGGPLPFAFDILTTAFGNR
VFVEYPEEIVDYFKQSFPEGYSWERSMSYEDGGICLATNNITMKKDGFVNEIRFDGVNFP
ANGPVMQRKTKWEPSTEKMYVRDGVLKGDVNMALLLQGGGHYRCDFRTTYKAKKVVQPDY
HFVDHQMEITSHDYNKVKLYEHAKAHSGLP
>gi|62732004|dbj|BAD95670.1||Favia_favus|Cnidarian-Anthozoa
ITSEMKMELRMEGAVNGHKFVITGKGSGQPFEGIQNMDLTVIGGPLPFAFDILTTVFGNR
VFVKYPEEIVDYFKQSFPEGYSWERSMSYEDGGICLATNNITMKKDGFVYEIRFDGVNFP
ANGPVMQRKTKWEPSTEKMYVRDGVLKGDVNMALLLQGGGHYRCDFRTTYKAKKVVQPDY
HFVDHRIEITSHDYNKVKLYEHAKAHSGLP
>gi|62738512|pdb|1XSS|A|Favia_favus|Cnidarian-Anthozoa
ITSEMKMELRMEGAVNGHKFVITGKGSGQPFEGIQNMDLTVIGGPLPFAFDILTTVFXNR
VFVKYPEEIVDYFKQSFPEGYSWERSMSYEDGGICLATNNITMKKDGFVYEIRFDGVNFP
ANGPVMQRKTKWEPSTEKMYVRDGVLKGDVNMALLLQGGGHYRCDFRTTYKAKKVVQPDY

HFVDHRIEITSHDYNKVKLYEHAKAHSGLP
>gi|93279225|pdb|2DDC|A|Favia_favus|Cnidarian-Anthozoa
ITSEMKIEVRMEGAVNGHKFVITGKGSGQPFEGIQNVDLTVIGGPLPFAFDILTTAFXNR
VFVKYPEEIVDYFKQSFPEGYSWERSMSYEDGGICLATNNITMKKDGFVNEIRFDGVNFP
ANGPVMQRKTKWESSTEKMYVRDGVLKGDVNMALLLQGGGHYRCDFRTTYKAKKVVQPDY
HFVDHLMEITSHDYNKVKLYEHAKAHSGLP
>gi|154814312|gb|ABS87207.1||Montastraea_cavernosa|Cnidarian-Anthozoa
IKSVMKIKLRMDGIVNGHKFMITGEGEGKPFEGTHTIILKVKGGPLPFAYDILTTAFGNR
VFTKYPKDIPDYFKQSFPEGYSWERSMTFEDQGVCTVTSDIKLEGDCFFYEIRFYGVNFP
SSGPVMQKKTKWEPSTENMYVRDGVLLGDVNMALLLEGGGHYRCDFKTTYKAKKVVQPDY
HFVDHRIEIVSHDYNKVKLYEHAEA-HSGL
>gi|12621060|gb|AAG54098.1||Renilla_muelleri|Cnidarian-Anthozoa
LQEVMSYKVNLEGIVNNHVFTMEGCGKGNILFGNQLVQIRVTGAPLPFAFDIVSPAFGNR
TFTKYPNDISDYFIQSFPAGFMYERTLRYEDGGLVEIRSDINLIEDKFVYRVEYKGSNFP
DDGPVMQKTIGIEPSFEAMYMNNGVLVGEVILVYKLNSGKYYSCHMKTLMKSKGVVKPSY
HFIQHRLE-KTYVGGFVEQHETAIAQMTSI
>gi|122920453|pdb|2HQK|A|Clavularia_sp.|Cnidarian-Anthozoa
IKPDMKIKLKMEGNVNGHAFVIEGEGEGKPYDGTNTINLEVKGAPLPFSYDILTTAFXNR
AFTKYPDDIPNYFKQSFPEGYSWERTMTFEDKGIVKVKSDISMEEDSFIYEIHLKGENFP
PNGPVMQKKTGWDASTERMYVRDGVLKGDVKHKLLLEGGGHHRVDFKTIYRAKKAVKPDY
HFVDHRIEILNHDYNKVTVYESAVARNST-
>gi|145580167|pdb|2OTB|A|Clavularia_sp.|Cnidarian-Anthozoa
IKPDMKIKLKMEGNVNGHAFVIEGEGEGKPYDGTNTINLEVKGAPLPFSYDILTNAFXNR
AFTKYPDDIPNYFKQSFPEGYSWERTMTFEDKGIVKVKSDISMEEDSFIYEIHLKGENFP
PNGPVMQKKTKWEPSTEILYVRDGVLVGDIKHKLLLEGGGHHRVDFKTIYRAKKAVKPDY
HFVDHRIEILNHDYNKVTVYESAVARY---
>gi|636666639|pdb|4Q9W|A|Clavularia_sp.|Cnidarian-Anthozoa
IKPDMKIKLKMEGNVNGYAFVIEGEGEGKPYDGTNTINLEVKGAPLPFSYDILTTAFXNR
AFTKYPDDIPNYFKQSFPEGYSWERTMTFEDKGIVKVKSDISLEEDSFIYEIYLKGENFP
PNGPVMQKKTGWDASTERMYVRDGVLKGDVKHKLLLEGGGYYRVDFKTIYRAKKAVKPDY
HFVDHRIEILNYDYNKVTVYESAVARNST-
>gi|720062878|pdb|4R6D|A|Clavularia_sp.|Cnidarian-Anthozoa
IKPDMKIKLKMEGNVNGYAFVIEGEGEGKPYDGTNTINLEVKGAPLPFSYDILTTAFXNR
AFTKYPDDIPNYFKQSFPEGYSWERTMTFEDKGIVKVKSDISLEEDSFIYEIYLKGENFP
PNGPVMQKKTGWDASTERMYVRDGVLKGDVKHKLLLEGGGYYRVDFKTIYRAKKAVKPDY
HFVDHRIECLNHDHNKVTVYESAVARN---
>gi|12621058|gb|AAG54097.1||Ptilosarcus_sp._CSG-2001|Cnidarian-Anthozoa
LKEIMSAKASVEGIVNNHVFSMEGFGKGNVLFGNQLMQIRVTGGPLPFAFDIVSIAFGNR
TFTKYPDDIADYFVQSFPAGFFYERNLRFEDGAIVDIRSDISLEDDKFHYKVEYRGNGFP
SNGPVMQKAIGMEPSFEVVYMNSGVLVGEVDLVYKLESGNYYSCHMKTFYRSKGGVKPEY
HFIHHRLE-KTYVGSFVEQHETAIAQLTTI
>gi|15298096|gb|AAK62982.2|AF384683_1|Montastraea_cavernosa|Cnidarian-Anthozoa
IKPIMEIKLRMQGVVNGHKFVIKGEGEGKPFEGTQTINLTVKGAPLPFAYDILTSAFGNR
VFTKYPDDIPDYFKQTFPEGYSWERIMAYEDQSICTATSDIKMEGDCFIYEIQFHGVNFP
PNGPVMQKKTKWEPSTEKMYVRDGVLKGDVNMALLLEGGGHYRCDFRSTYKAKKRVQPDY
HFVDHRIEILSHDYNTVKLSEDAEARYSML

>gi|154814310|gb|ABS87206.1||Montastraea_cavernosa|Cnidarian-Anthozoa
IKSVMKIKLRMDGIVNGHKFMITGEGEGKPFEGTHTIILKVKGGPLPFAYDILTTAFGNR
VFTKYPKDIPDYFKQSFPEGYSWERSMTFEDQGVCTVTSDIKLEGDCFFYEIRFYGVNFP
SSGPVMQKKTKWEPSTENMYVRDGVLLGDVSRTLLLEGNKHHRCNFRSTYRAKKGVVPEY
HFVDHRIEILSHDYNTVEVYENAVARPSML
>gi|16508125|gb|AAL17905.1||Montastraea_cavernosa|Cnidarian-Anthozoa
IKSVMKIKLRMDGIVNGHKFMITGEGEGKPFEGTHTIILKVKGGPLPFAYDILTTAFGNR
VFTKYPKDIPDYFKQSFPEGYSWERSMTFEDQGVCTVTSDIKLEGDCFFYEIRFYGVNFP
SSGPVMQKKTKWEPSTENMYVRDGVLLGDVSRTLLLEGDKHHRCNFRSTYGAKKGVVPEY
HFVDHRIEILSHDYNTVEVYENAVARPSML
>gi|32188174|gb|AAO61602.1||Montastraea_cavernosa|Cnidarian-Anthozoa
IKSVMKIKLHMDGIVNGHKFMITGEGEGKPFEGTHTIILKVKGGPLPFAYDILTTAFGNR
VFTKYPKDIPDYFKQSFPEGYSWERSMTFEDQGVCTVTSDIKLEGDCFFYEIRFYGVNFP
SSGPVMQKKTKWEPSTENMYVRDGVLLGDVSRTLLLEGDKHHRCNFRSTYRAKKGVVPEY
HFVDHRIEILSHDYNTVEVYENAVARPSML
>gi|154814318|gb|ABS87210.1||Montastraea_cavernosa|Cnidarian-Anthozoa
IKPIMEIKLRMQGVVNGHKFVIKGEGEGKPFEGTQTINLTVKGAPLPFAYDILTSAFGNR
VFTKYPDDIPDYFKQTFPEGYSWERIMAYEDQSICTATSDIKMEGDCFIYEIQFHGVNFP
PNGPVMQKKTKWEPSTEKMYVRDGVLKGDVNMALLLEGGGHYRCDFKTTYKAKKVVRPDY
HFVDHRIEIVSHDYNKVKLYEHAEA-HSGL
>gi|239781655|pdb|2VZX|A|Dendronephthya_sp._SSAL-2002|Cnidarian-Anthozoa
IKEDMRVKVHMEGNVNGHAFVIEGEGKGKPYEGTQTANLTVKGAPLPFSYDILTTAVXNR
VFTKYPEDIPDYFKQSFPEGYSWERTMTFEDKGICTIRSDISLEGDCFFQNVRFKGTNFP
PNGPVMQKKTKWEPSTEKLHVRDGLLVGNINMALLLEGGGHYLCDFKTTYKAKKVVQPDA
HFVDHRIEILGNDYNKVKLYEHAVARYSPL
>gi|269993106|emb|CBI12485.1||Cyphastrea_microphthalma|Cnidarian-Anthozoa
IKPDMRIKLRMEGAVNGHKFVILGDGNGKPYEGTQTIDVTVKGGPLPFAYDILTSAFGNR
VFTKYPDDIADYFKQSFPVGYSWERSMTYEDGGICTVSSDIKMEGNSFIYEIRFHGLNFP
SDGPVMQKKTKWEPSTEKMYVRDGVLKGDVNMTLLLEGGGHYRCDFKSTYKAKRAVQPDY
HYIDHRIEILSHDYNKVKLCENAAARCSML
>gi|28974113|gb|AAO00732.1||Meandrina_meandrites|Cnidarian-Anthozoa
VPTQVKMKYSMDGNFNGQSFTVVGEGTGNPYEGHQSLKLTV-GEPLPFAFDILSATFGNR
VFTKYPEGKTDYFKEAFPGGLTWERTMTFEDGGICTVAAEISLTGSVFEHKSKFVGVNFP
ANGPVIQKKTGWETSTEKMAANDGSVQGYDTMFLKLEGGGRHKCYFVTNHKAKRAVKPDN
HFVW---HRLVRNGNTVELEETAEARYDS-
>gi|28974115|gb|AAO00733.1||Meandrina_meandrites|Cnidarian-Anthozoa
VPTQVKMKYSMDGNFNGQSFTVVGEGTGNPYEGHQSLKLTV-GEPLPFAFDILSATFGNR
VFTKYPEGKTDYFKEAFPGGLTWERTMTFEDGGICTVAAEISLTGNVFEHKSKFVGVNFP
ANGPVIQKKTGWETSTEKMAANDGSVQGYDTMFLKLEGGGRHKCYFVTNHKAKRAVKPDN
HFVW---HRLVRNGNTVELEETAEARYDS-
>gi|296278350|pdb|3ADF|A|Galaxea_fascicularis|Cnidarian-Anthozoa
IKPEMKIKLCMRGTVNGHNFVIEGEGKGNPYEGTQILDLNVTGAPLPFAYDILTTVFXNR
AFTKYPADIQDYFKQTFPEGYHWERSMTYEDQGICTATSNISMRGDCFFYDIRFDGTNFP
PNGPVMQKKTKWEPSTEKMYVEDGVLKGDVNMRLLLEGGGHYRCDFKTTYKAKKEVRPDA
HKIDHRIEILKHDYNKVKLYENAVARYSML
>gi|52839539|dbj|BAD52001.1||Galaxea_fascicularis|Cnidarian-Anthozoa

IKPEMKIKLCMRGTVNGHNFVIEGEGKGNPYEGTQILDLNVTGAPLPFAYDILTTVFGNR
AFTKYPADIQDYFKQTFPEGYHWERSMTYEDQGICTATSNISMRGDCFFYDIRFDGVNFP
PNGPVMQKKTKWEPSTEKMYVRDGVLKGDVNMALLLEGGGHYRCDFKTTYKAKKDVRPDY
HFVDHRIEILKHDYNKVKLYENAVARYSML
>gi|52839541|dbj|BAD52002.1||Galaxea_fascicularis|Cnidarian-Anthozoa
IKPEMKIKLCMRGTVNGHNFVIEGEGKGNPYEGTQILDLNVTGAPLPFAYDILTTVFGNR
AFTKYPADIQDYFKQTFPEGYHWERSMTYEDQGICTATSNISMRGDCFFYDIRFDGTNFP
PNGPVMQKKTKWEPSTEKMYVRDGVLKGDVNMALLLEGGGHYRCDFKTTYKAKKDVRPDA
HKVDHRIEILKHDYNKVKLYENAVARYSML
>gi|33333768|gb|AAQ11989.1||Cerianthus_membranaceus|Cnidarian-Anthozoa
LDNNLSVSVYMKGNVNNHEFEYDGIGGGDPNSGQFSLKTKLRGKPLPFSYDIITMGFGFR
AFTKYPEGIADYFKGSFPEAFQWNRRIEFEDGGVINMSSDITYKDKVLHGDVWALGVNFP
PNGPVMKNEIMEEPAEETLTAKNGVLVGFCPKAYLLKDGSYYYGHMTTFYRSKKSGQPGF
HFIKHRLVKTKVEFKMVEQAEYATAHVCDL
>gi|119389059|pdb|2C9J|A|Cerianthus_membranaceus|Cnidarian-Anthozoa
LDNNLSVSVYMKGNVNNHEFEYDGIGGGDPNSGQFSLKTKLRGKPLPFSYDIITMGFXFR
AFTKYPEGIADYFKGSFPEAFQWNRRIEFEDGGVINMSSDITYKDKVLHGDVWALGVNFP
PNGPVMKNEIMEEPAEETLTAKNGVLVGFCPKAYLLKDGSYYYGHMTTFYRSKKSGQPGF
HFIKHRLVKTKVEFKMVEQAEYATAHVCDL
>gi|42521552|gb|AAS18270.1||Astrangia_lajollaensis|Cnidarian-Anthozoa
IGKEMNMTYLMDGSVNGHNFTVEGEGDGKPYEGHQCLKLRITGEPLPFAFDILTAAFGNR
CFVNYPAEIADYFKQAFPEGLTWERSMTFEDGGFAAVSADISLNGNCFVHQSKFVGVNFP
ADGPVMQKKTGWEPSTEKMIVRDGILEGDVTMYLKLEGGGNYRCEYRTIYKAKKDVKPKS
HFVEHTLVRNNIKGNTIELIEDASARY---
>gi|42521554|gb|AAS18271.1||Astrangia_lajollaensis|Cnidarian-Anthozoa
LSKQMNMTYLMDGSVNGHNFTVEGEGDGKPYEGHQCLKLRITGEPLPFAFDILTAAFGNR
CFVNYPAEIADYFKQAFPEGLTWERSMTFEDGGFAAVSADLSLNGNWFVHQSKFVGVNFP
ADGPVMQKKTGWEPSTEKMIVRDGTLEGDVTMYLKLEGGGNYRCDYRTIYKAKKDVKPKS
HFVEHTLVRNNIKGNTIELIEDASARY---
>gi|471179648|gb|AGI04830.1||Agaricia_agaricites|Cnidarian-Anthozoa
---LPFSFDLLTPAFGNR
VITKYPEDIPDYFKQAFPEGYHWERSITFEDQAVCTATSHI-------------------

>gi|471179803|gb|AGI04903.1||Agaricia_lamarcki|Cnidarian-Anthozoa
---LPFSFDLLTPAFGNR
VITKYPEDIPDYFKQAFPEGYHWERSINFEDQAVCTATSHI-------------------

>gi|471179740|gb|AGI04872.1||Agaricia_agaricites|Cnidarian-Anthozoa
---LPFSFDLLTPAFGNR
VFTKYPEDMPDYFKQAFPEGYHWERSITFEDHAVCTATSHI-------------------

>gi|471179783|gb|AGI04893.1||Agaricia_fragilis|Cnidarian-Anthozoa
---LPFSFDLLTPAFGNR

VFTKYPEDIPDYFKQAFPEGYQWERSITFEDQVGPELTIEF-------------------

>gi|471179785|gb|AGI04894.1||Agaricia_fragilis|Cnidarian-Anthozoa
---LPFSFDLLTPAFGNR
VFTKYPEDIPDYFKQAFPEGYQWDRSITFEDQVGPELTIEF-------------------

>gi|471179826|gb|AGI04914.1||Agaricia_lamarcki|Cnidarian-Anthozoa
---LPFSFXXLXXAFGNR
VFTXXPENIPDYFKQTFPEGYHWERSVPFEDQAVCTVTSHI-------------------

>gi|471179851|gb|AGI04926.1||Agaricia_lamarcki|Cnidarian-Anthozoa
---LPFSFDILTPAFGNR
VFTKYPENIPDYFKQTFPEGYHWERSVPFEDQAVCTVTSHI-------------------

>gi|78059162|gb|ABB17958.1||Eusmilia_fastigiata|Cnidarian-Anthozoa
IQTDMKMTYSMEGCVNGHNFTVKGGGDGNPYEGHQELRLCITGEPVPFAFDILSAAFGNR
CFTKYPDEIRDYFKQAFPGGLSWERSMAFEDGASAAVTAEISLEGDCFEHECEFVGVNFP
ANGPVMQKKTGWETSTEKMTAHGKVVQGNVPMFLKLEGGGRHRCDFRTTYKAKKDVKPNS
HFIT---HCLVRKGNNTELIEDAEARN---
>gi|19982569|gb|AAK71335.1||Montastraea_cavernosa|Cnidarian-Anthozoa
IKPDMKMKLRMEGAVNGHKFVVEGDGKGKPFDGTQTMDLTVIGAPLPFAYDILTTVFGNR
VFAKYPEDIADYFKQTFPEGYFWERSMTYEDQGICIATNDITMMEGVFAYKIRFDGVNFP
ANGPVMQRKTKWEPSTEIMYARDGVLKGDVNMALLLEGGGHYRCDFKTTYKAKKVVRPDY
HFVDHRIEIVSHDYNKVKLHEHAEARHGLS
>gi|32188170|gb|AAO61600.1||Montastraea_cavernosa|Cnidarian-Anthozoa
IKPDMKMKLRMEGAVNGHKFVIEGDGKGKPFDGTQTMDLTVIGAPLPFAYDILTTVFGNR
VFAKYPEDIADYFKQTFPEGYFWERSMTYEDQGICIATNDITMMKGVFVYKIRFDGVNFP
ANGPVMQRKTKWEPSTEIMYVRDGVLKGDVNMALLLEGGGHYRCDFKTTYKAKKVVRPDY
HFVDHRIEIVSHDYNKVKLHEHAEARHGLS
>gi|15425965|gb|AAK97633.1|AF406766_1|Montastraea_cavernosa|Cnidarian-Anthozoa
IKPDMKIKLRMEGAVNGHKFVIEGDGKGKPFEGTQSMDLTVIGAPLPFAYDILTTVFGNR
VFAKYPQDITDYFKQTFPEGYFWERSMTYEDQGICIATNDITMMKGVFVYKIRFDGVNFP
ANGPVMQRKTKWEPSTEIMYARDGVLKGDVNMALLLEGGGHYRCDFKTTYKAKKVVRPDY
HFVDHRIEIVSHDYNKVKLYEHAEAXXGLS
>gi|21303778|gb|AAK71334.1||Montastraea_cavernosa|Cnidarian-Anthozoa
IKPDMKMKLRMEGAVNGHKFVIEGDGKGKPFDGTQTMDLTVIGAPLPFAYAILTTVFGNR
VFAKYPEDIADYFKQTFPEGYFWERSMTYEDQGICIATNDITMMKGVFVYKIRFDGVNFP
ANGPVMQRKTKWEPSTEKMYXRDGVLKGDVNMALLLEGGGHYRXDXKTTYRAKKVVQPDY
HFVDHRIEIVSHDYNKVKLYEHAEAHSGLP
>gi|85002150|gb|ABC68476.1||Montastraea_faveolata|Cnidarian-Anthozoa
MKSEMKIKLRMEGSVNGHTFEIEGEGKGKPFE----------------------------

>gi|187564291|gb|ACD13194.1||Platygyra_lamellina|Cnidarian-Anthozoa
IKRDMKIKLRMEGAVNGHKFVIIGNGDGKPYEGTQTIDLEVIGAPLPFAFDILTTVFGNR
VFAKYPNEIVDYFKQSFPEGYSWERSMTYEDGGICIATNNITLS---FHYNIRFDGVNFP
PKSPVLQKTTKWEPSTEIMYVRDGVLKGDVNMALLIEGGGHYRCDFKTIYKAKKVVEPDY
HFVDHRITIKSHDYNKVLLHEHAKARYGLQ
>gi|78059184|gb|ABB17969.1||Echinophyllia_echinata|Cnidarian-Anthozoa
IKPDMKIRLRMEGAVNGHKFVIIGKGDGKPYEGTQTMDLEVIGGPLPFAFDILTTVFGNR
AFVKYPTDIADYFKQSFPEGFSWERSMTYEDGGICIATNDITLSKDIFDYNIRFDGVNFP
PNSPVLQKTTKWEPSTENMYVRDGVLKGDINMSLLLEGGAGYRCDFKTTYKAKKAVKPDY
HFVDHRITIVSHDYNKVKLREHAEAHSGLQ
>gi|187564277|gb|ACD13193.1||Favites_abdita|Cnidarian-Anthozoa
IKPDMKIKLRMEGAVNGHKFAIEGEGKGQPFQGKQTMNLKVKGGPLPFAYDILTTIFGNR
VFAKYPDDIVDYFKQSFPEGYSWERSMTYEDGGICIATNDITLDGDCFVYKIRFDGVNFP
AKSPVLQKKTKWEPSTEKLYVRDGLLKGDVNMALLLEGGGHYRCDFKTTYKAKKVVQPDY
HFVDHCIEIVSHDYNNVKLCEHAEAHSGLP
>gi|56749100|sp|Q9U6Y3.1|GFPL_CLASP|Clavularia_sp.|Cnidarian-Anthozoa
IKPDMKIKLKMEGNVNGHAFVIEGEGEGKPYDGTHTLNLEVKGAPLPFSYDILSNAFGNR
ALTKYPDDIADYFKQSFPEGYSWERTMTFEDKGIVKVKSDISMEEDSFIYEIRFDGMNFP
PNGPVMQKKTKWEPSTEIMYVRDGVLVGDISHSLLLEGGGHYRCDFKSIYKAKKVVKPDY
HFVDHRIEILNHDYNKVTLYENAVARYSLL
>gi|149241738|pdb|2GX0|A|Echinophyllia_sp._SC22|Cnidarian-Anthozoa
IKPDMKIKLRMEGAVNGHPFAIEGVGLGKPFEGKQSMDLKVKGGPLPFAYDILTTVFXNR
VFAKYPENIVDYFKQSFPEGYSWERSMNYEDGGICNATNDITLDGDCYIYEIRFDGVNFP
ANGPVMQKRTKWEPSTEKLYVRDGVLKGDVNMALSLEGGGHYRCDFKTTYKAKKVVQPDY
HFVDHHIEIKSHDYSNVNLHEHAEAHSELP
>gi|154814316|gb|ABS87209.1||Montastraea_cavernosa|Cnidarian-Anthozoa
IKPDMKIKLRMEGAVNGHYFVIEGEGKGKPFEGTQSMDLTVKGAPLPFAYDIMTTAFGNR
VFAKYPKDIPDYFKQMFPEGYSWERSMTFEDGGICTARNEITMEGDCFFNKVRFDGVNFP
PNGPVMQKKTKWEPSTEKMYVRDGVLTGDINMALLLQGGVHYRCDFRTTYKAKEGVEPGY
HFVDHCIEILSHDYNKVKLYEYAEAHSGLP
>gi|188036188|pdb|2Z1O|A|Echinophyllia_sp._SC22|Cnidarian-Anthozoa
IKPDMKIKLRMEGAVNGHPFAIEGVGLGKPFEGKQSMDLKVKGGPLPFAYDILTTVFXNR
VFAKYPENIVDYFKQSFPEGYSWERSMNYEDGGICNATNDITLDGDCYIYEIRFDGVNFP
ANGPVMQKRTKWEPSTEKLYVRDGVLKGDVNMALSLEGGGHYRCDFKTTYKAKKVVQPDY
HFVDHHIEIKSHDYSNVNLHEHAEAHSELP
>gi|119390114|pdb|2IE2|A|Echinophyllia_sp._SC22|Cnidarian-Anthozoa
IKPDMKIKLRMEGAVNGHPFAIEGVGLGKPFEGKQSMDLKVKGGPLPFAYDILTTVFXNR
VFAKYPENIVDYFKQSFPEGYSWERSMNYEDGGICNATNDITLDGDCYIYEIRFDGVNFP
ANGPVMQKRTKWEPSTEKLYVRDGVLKGDVNMALSLEGGGHYRCDFKTTYKAKKVVQPDY
HFVDHHIEIKSHDYSNVNLHEHAEAHSELP
>gi|343197768|pdb|3ZUF|A|Echinophyllia_sp._SC22|Cnidarian-Anthozoa
IKPDMKIKLRMEGAVNGHPFAIEGVGLGKPFEGKQSMDLKVKGGPLPFAYDILTMAFXNR
VFAKYPENIVDYFKQSFPEGYSWERSMIYEDGGICNATNDITLDGDCYIYEIRFDGVNFP
ANGPVMQKRTKWELSTEKLYVRDGVLKSDGNYALSLEGGGHYRCDFKTTYKAKKVVQPDY

HSVDHHIEIKSHDYSNVNLHEHAEAHSE--
>gi|343197774|pdb|3ZUJ|A|Echinophyllia_sp._SC22|Cnidarian-Anthozoa
IKPDMKIKLRMEGAVNGHPFAIEGVGLGKPFEGKQSMDLKVKGGPLPFAYDILTMAFXNR
VFAKYPENIVDYFKQSFPEGYSWERSMIYEDGGICNATNDITLDGDCYIYEIRFDGVNFP
ANGPVMQKRTKWELSTEKLYVRDGVLKSDGNYALSLEGGGHYRCDFKTTYKAKKVVQPDY
HSVDHHIEIKSHDYSNVNLHEHAEAHS---
>gi|356624727|pdb|3ZUL|A|Echinophyllia_sp._SC22|Cnidarian-Anthozoa
IKPDMKIKLRMEGAVNGHPFAIEGVGLGKPFEGKQSMDLKVKGGPLPFAYDILTMAFXNR
VFAKYPENIVDYFKQSFPEGYSWERSMIYEDGGICNATNDITLDGDCYIYEIRFDGVNFP
ANGPVMQKRTKWELSTEKLYVRDGVLKSDGNYALSLEGGGHYRCDFKTTYKAKKVVQPDY
HSVDHHIEIKSHDYSNVNLHEHAEAHSELP
>gi|559778650|pdb|4HQC|A|Echinophyllia_sp._SC22|Cnidarian-Anthozoa
IKPDMKIKLRMEGAVNGHPFAIEGVGLGKPFEGKQSMDLKVKGGPLPFAYDILTTAXXNR
VFAKYPENIVDYFKQSFPEGYSWERSMSYEDGGICIATNDITLDGDCYIYEIRFDGVNFP
ANGPVMQKRTKWEPSTEKLYVRDGVLKGDVNMALSLEGGGHYRCDFKTTYKAKKVVQPDY
HFVDHHIEIKSHDYSNVNLHEHAEAHSGLP
>gi|559778651|pdb|4HQC|B|Echinophyllia_sp._SC22|Cnidarian-Anthozoa
IKPDMKIKLRMEGAVNGHPFAIEGVGLGKPFEGKQSMDLKVKGGPLPFAYDILTTAXXNR
VFAKYPENIVDYFKQSFPEGYSWERSMSYEDGGICIATNDITLDGDCYIYEIRFDGVNFP
ANGPVMQKRTKWEPSTEKLYVRDGVLKGDVNMALSLEGGGHYRCDFKTTYKAKKVVQPDY
HFVDHHIEIKSHDYSNVNLHEHAEAHSGLP
>gi|559778653|pdb|4HQC|D|Echinophyllia_sp._SC22|Cnidarian-Anthozoa
IKPDMKIKLRMEGAVNGHPFAIEGVGLGKPFEGKQSMDLKVKGGPLPFAYDILTTAXXNR
VFAKYPENIVDYFKQSFPEGYSWERSMSYEDGGICIATNDITLDGDCYIYEIRFDGVNFP
ANGPVMQKRTKWEPSTEKLYVRDGVLKGDVNMALSLEGGGHYRCDFKTTYKAKKVVQPDY
HFVDHHIEIKSHDYSNVNLHEHAEAHSGLP
>gi|56201403|dbj|BAD72874.1||Echinophyllia_sp._SC22|Cnidarian-Anthozoa
IKPDMKIKLRMEGAVNGHPFAIEGVGLGKPFEGKQSMDLKVKGGPLPFAYDILTTVFGNR
VFAKYPENIVDYFKQSFPEGYSWERSMNYEDGGICNATNDITLDGDCYIYEIRFDGVNFP
ANGPVMQKRTKWEPSTEKLYVRDGVLKGDVNMALSLEGGGHYRCDFKTTYKAKKVVQPDY
HFVDHHIEIKSHDYSNVNLHEHAEAHSELP
>gi|19982597|gb|AAK71336.1||Montastraea_cavernosa|Cnidarian-Anthozoa
IKSVMKIKLRMEGSVNGHNFVIVGEGEGKPYEGTQSMDLTVKGAPLPFAYDIMTTVFGNR
VFAKYPKHIPDYFKQMFPEEYSWERSMNFEGGGICTARNEITMEGDCFFNKVRFDGVNFP
PNGPVMQKKTKWEPSTEKMYVRDGVLTGDINMALLLEGGGHYRCDFRTTYRAKKGVKPDY
HFEDHSIEILRHDYTEVKLYEHAEAHSGLP
>gi|32188166|gb|AAO61598.1||Montastraea_cavernosa|Cnidarian-Anthozoa
IKSVMKIKLRMEGSVNGHNFVIVGEGEGKPYEGTQSMDLTVKGAPLPFAYDIMTTVFGNR
VFAKYPKHIPDYFKQMFPEGYSWERSMNFEDGGICTARNEITMEGDCFFNKVRFDGVNFP
PNGPVMQKKTKWEPSTEKMYVRDGVLTGDINMALLLEGGGHYRCDFRTTYRAKKGVKPDY
HFVDHSIEILRHDYTEVKLYEHAEAHSGLP
>gi|37287363|gb|AAQ90465.1||Montastraea_cavernosa|Cnidarian-Anthozoa
IKSVMKIKRRMEGSVNGHNFVIVGEGEGKPYEGTQSMDLTVKGAPLPFAYDIMTTVFGNR
VFAKYPKHIPDYFKQMFPEGYSWERSMNFEDGGICIARNEITMEGDCFFNKVRFDGVNFP
PNGPVMQKKTKWEPSTEKMYVRDGVLTGDINMALLLEGGGHYRCDFRTTYRAKKGVKPDY
HFVDHSIEILRHDYTEVKLYEHAEAHSGLP

>gi|19982609|gb|AAK71337.1||Scolymia_cubensis|Cnidarian-Anthozoa
VKDFMKITLRMDGAVNGKPFAVNGTGDGNPYGGIQSLKLTVDNKPLPFAFDILSAAFGNR
AFTEYPKEISDYFKQSFEGGFTWERSFTFEDGAICVATNDIKMVGDEFQYNIRFDGVNFP
EDGPVMQKKTKWEPSTEIMRVQGGVLKGEVNMALLLKDKSHYRCDFKTTYKAKNPVPPXY
HYVDHCIEITEEN--YVKLQEYAKARSGLH
>gi|257221221|gb|ACV52376.1||Scleractinia_sp._Lizard_Island_35|Cnidarian-Anthozoa
IKPDMKIKLHMKGAVNGHIFEIDGEGNGKPFEGKQTIELKVVGGPLPFAFDILTTVFGNR
VFAKYPPEIVDYFKQSFPEGYSWERSMMYEDGGICIATNNITLLK--FYYNIRFDGVNFP
TNGPVMQKKTKWEPSTEKMYVRDGVLKGDVNMALLIEGGGHNRCDFKTTYKARKAVPPNY
HFVDHRIEIVSHDYNHVKLCEHAEAHSGLP
>gi|257221243|gb|ACV52387.1||Scleractinia_sp._Lizard_Island_39|Cnidarian-Anthozoa
IKPDMKIKLCMEGAVNGHPFMIEGEGKGKPFDGKQNMELKVKGGPLPFAYDILTTVFGNR
VFAKYPRDIADYFKQSFPEGFSWERSMAYEDGGICIATNNITLMKGD-XYXIRFDGVNFP
ANXPVMQKKXKWEPSTEKLYVRDGVLKGDINMALLLEGGGHYRCDFKTT-----------

>gi|291191300|pdb|3LS3|A|Pectiniidae|Cnidarian-Anthozoa
IKPDMKIKLRMEGAVNGHPFAIEGVGLGKPFEGKQSMDLKVKGGPLPFAYDILTMAFXNR
VFAKYPENIVDYFKQSFPEGYSWERSMIYEDGGICNATNDITLDGDCYICEIRFDGVNFP
ANGPVMQKRTKWELSTEKLYVRDGVLKSDGNYALSLEGGGHYRCDFKTTYKAKKVVQPDY
HSVDHHIEIISHDYSNVNLHEHAEAHSELP
>gi|557804866|pdb|4HQ8|A|Echinophyllia_sp._SC22|Cnidarian-Anthozoa
IKPDMKIKLRMEGAVNGHPFAIEGVGLGKPFEGKQSMDLKVKGGPLPFAYDILTTAFXNR
VFAKYPENIVDYFKQSFPEGYSWERSMSYEDGGICIATNDITLDGDCYIYEIRFDGVNFP
ANGPVMQKRTKWEPSTEKLYVRDGVLKGDVNMALSLEGGGHYRCDFKTTYKAKKVVQPDY
HFVDHHIEIKSHDYSNVNLHEHAEAHSGLP
>gi|119390252|pdb|2IOV|A|Echinophyllia_sp._SC22|Cnidarian-Anthozoa
IKPDMKIKLRMEGAVNGHPFAIEGVGLGKPFEGKQSMDLKVKGGPLPFAYDILTTVFXNR
VFAKYPENIVDYFKQSFPEGYSWERSMNYEDGGICNATNDITLDGDCYIYEIRFDGVNFP
ANGPVMQKRTKWEPSTEKLYVRDGVLKGDVNMALSLEGGGHYRCDFKTTYKAKKVVQPDY
HFVDHHIEIKSHDYSNVNLHEHAEAHSELP
>gi|194368617|pdb|2Z6X|A|Pectiniidae|Cnidarian-Anthozoa
IKPDMKIKLRMEGAVNGHPFAIEGVGLGKPFEGKQSMDLKVKGGPLPFAYDILTTVFXNR
VFAKYPENIVDYFKQSFPEGYSWERSMNYEDGGICIATNDITLDGDCFIYEIRFDGVNFP
ANGPVMQKRTKWEPSTEKLYVRDGVLKGDVNMALLLEGGGHYRCDFKTTYKAKKVVQPDY
HFVDHRIEIKSHDYNNVNLHEHAEAHSGLP
>gi|422919356|pdb|4EMQ|A|Echinophyllia_sp._SC22|Cnidarian-Anthozoa
IKPDMKIKLRMEGAVNGHPFAIEGVGLGKPFEGKQSMDLKVKGGPLPFAYDILTTVFXNR
VFAKYPENIVDYFKQSFPEGYSWERSMNYEDGGICNATNDITLDGDCYIYEIRFDGVNFP
ANGPVMQKRTKWEPSTENLYVRDGVLKGDVNMALSLEGGGHYRCDFKTTYKAKKVVQPDY
HFVDHHIEIKSHDYSNVNLHEHAEAHSELP
>gi|559778652|pdb|4HQC|C|Echinophyllia_sp._SC22|Cnidarian-Anthozoa
IKPDMKIKLRMEGAVNGHPFAIEGVGLGKPFEGKQSMDLKVKGGPLPFAYDILTTAXXNR
VFAKYPENIVDYFKQSFPEGYSWERSMSYEDGGICIATNDITLDGDCYIYEIRFDGVNFP
ANGPVMQKRTKWEPSTEKLYVRDGVLKGDVNMALSLEGGGHYRCDFKTTYKAKKVVQPDY
HFVDHHIEIKSHDYSNVNLHEHAEAHSGLP
>gi|582045224|pdb|4IZN|A|Echinophyllia_sp._SC22|Cnidarian-Anthozoa

IKPDMKIKLRMEGAVNGHPFAIEGVGLGKPFEGKQSMDLKVKGGPLPFAYDILTTAFXNR
VFTKYPENIVDYFKQSFPEGYSWERSMSYEDGGICIATNDITLDGDCYIYEIRFDGVNFP
ANGPVMQKRTKWEPSTEKLYVRDGVLKGDVNMALSLEGGGHYRCDFKTTYKAKKVVQPDY
HFVDHHIEIKSHDYSNVNLHEHAEAHSGLP
>gi|78059158|gb|ABB17956.1||Echinophyllia_echinata|Cnidarian-Anthozoa
INPDMKIKLYMEGAVNGHKFEIKGEGNGKPFEGKQTMDLAVVGGPLPFAFDILTTSFGNR
VFTKYPDTIVDYFKPSFPEGYSWERSMTYEDGGICIATNDITLLK--FYYKIRFDGVNFA
ANGPVMQKKTKWEPSTEKMYVRDGVLKGEVNMALLLEGGGHYRCDFKTTYKAKKVVRPSY
HFVDHRIEILSHSYNQVRLHEHAEAHSGLP
>gi|78059170|gb|ABB17962.1||Galaxea_fascicularis|Cnidarian-Anthozoa
IKPDMKIKLCMRGTINGHNFVIEGEGKGNPYEGTQILDLNVTGAPLPFAYDILTTVFGNR
AFTKYPADIQDYFKQTFPEGYHWERSMTYEDQGICTATSNISMRGDCFFYDIRFDGVNFP
PNGPVMQKKTKWEPSTEKMYVRDGVLKGDVNMALLLEGGGHYRCDFKTTYKAKKDVRPDY
HFVDHRIEILKHDYNKVELYENAVARYSML
>gi|85002148|gb|ABC68475.1||Montastraea_faveolata|Cnidarian-Anthozoa
IKPDMKIKLRMKGAVNGHTFEIEGEGKGKPFEGKQNMDLKVVGGPLPFAYDILTTVFGNR
VFAKYPGDIADYFKQSFPEGYSWERSMTYEDGGVCIATNDITLVGDV-RSEIRFEGVNFP
ANGPVMQKKTKWEPSTEKMYVRDGVLKGDVNMALLLEGGGHYRCDFKTTYKAKKVVQPEY
HFVDHRIEIVSHDYNIVKLREHAEAHFGLP
>gi|78059182|gb|ABB17968.1||Echinophyllia_echinata|Cnidarian-Anthozoa
IKPDMKIKLRMEGAVNGHKFAIEGEGNGQPFEGKQTMNLKVKGGPLPFAYDILTTIFGNR
VFVKYPDDIVDYFKQSFPEGYSWERSMIYEDGGICIATNDITLEGDCFVYKIRFDGVNFP
AKSPVLQKMTKWEPSTEKLYVRDGVLKGDVNMALLLEGGGHFRCDFKTTYKAKKVVQPDY
HFVDHRIEIMSHDYNNVKLCEHAEAHSGLP
>gi|21303776|gb|AAK71332.1||Montastraea_annularis|Cnidarian-Anthozoa
IKPEMKIKMRMDGAVNGHKFVITGEGSGEPFEGKQTMNLTVIGGPLPFAFDILTTAFGXR
VFAKYPEDIPDYFKQSFPEGFSWERSMTYEDGGICIATNDIKMEGDCFSYEIRFDGVNFP
ANSPVMQKKTKWEPCTXEMYVRDGVLKGGLNMALLLEGGGHFRCDLKTTYKAKKVVQPDY
HFVNHRLEITWHDYNNVKLSEHAEAHSGLP
>gi|257221215|gb|ACV52373.1||Scleractinia_sp._Lizard_Island_28|Cnidarian-Anthozoa
IKTDMKMKLRMVGAVNGHKFEIAGEGKGKPFEGKQTMNLEVLGGPLPFAFDILTTVFGNR
VFVKYPKDIXBYFKQSFPEGFSWERSMAYEDGGICIXTNBITLMKGD-LYEIRFDGVNFP
ANSPVXQKXTKWEPSTEKLYVRDGVLKGDVNMALLLEEGGHYRCDFKTTYKSKKVVQPDY
HFVDHRIEIESHDYNKVKLCEHAEAHSGLP
>gi|257221219|gb|ACV52375.1||Lobophyllia_hemprichii|Cnidarian-Anthozoa
IKTDMKMKLRMXGAVNGHKFVIAGEGRGQPFEGKQTMDLXVXGGPLPFAFDILTTVFGNR
VFVKYPRDIADYFKQSFPEGFSWERSMAYEDGGICIATNNITLMKGD-LYEIRFDGVNFP
ANSPVMQKRTKWEPSTEKLYVRDGVLKGDVNMALLLEEGGHYRCDFKTTYKSKKVVQPDY
HFVDHRIEIESHDYNKVKLCEHAEAHSGLP
>gi|257221239|gb|ACV52385.1||Scleractinia_sp._Lizard_Island_38|Cnidarian-Anthozoa
IKPDMKMKLRMEGAVNGHKFVIAGEGRGQPFEGKQTMDLTVKGGPLPFAFDILTTVFGNR
VFVKYPRDIADYFKQSFPEGFSWERSMAYEDGGICIATNNITLMKGD-LYEIRFDGVNFP
ANSPVMQKRTKWEPSTEKL--

>gi|257221241|gb|ACV52386.1||Scleractinia_sp._Lizard_Island_38|Cnidarian-Anthozoa
IKPDMKMKLRMEGAVNGHKFVIAGEGRGQPFEGKQTMNLEVLGGPLPFAFDILTTVFGNR

VFVKYPNDIADYFKQSFPEGFSWERSMAYEDGGICLATNDITLNGDCFLYEIRFDGVNFP
ANSPVMQKRTKWEPSTEKL--

>gi|257221229|gb|ACV52380.1||Lobophyllia_hemprichii|Cnidarian-Anthozoa
ITLKGDCKLRMVGAVNGHKFEIAGEGKGKPFEGKQTMDLKVLGGPLPFAYDILTTVFGNR
VFVKYPBBIEGHFKQSFPEGFSWQRSMAYEDGGICLATNDITLKGDCFLYEIRFDGVNFP
ANSPVMQKKTKWEPSTEKLYVR-------------------------------------

>gi|257221231|gb|ACV52381.1||Lobophyllia_hemprichii|Cnidarian-Anthozoa
-KATVKMKLRMVGAVNGHKFEIAGEGKGKPFEGKQTMNLEVLGGPLPFAFDILTTVFGNR
VFVKYPNDIADYFKQSFPEGFSWERSMAYEDGGICIATNDITLKGDCFLYEIRFDGVNFP
ANSPVMQKRTKWEPSTEKL--

>gi|19982586|gb|AAK71333.1||Scolymia_cubensis|Cnidarian-Anthozoa
VKEHMKIKLRMGGTVNGKHFAVNGTGDGYPYQGKQILKLIVESEPLPFAFDILSAAFGNR
AFTEYPTEIADYFKQSFEGGFSWERSFTFEDGAICVATNDITMVGGEFQYDIRFDGLNFP
XDGPVMQKKTKWEPSTEIMLKTTYREHKARLSPAKERKA---------------------

>gi|257221223|gb|ACV52377.1||Scleractinia_sp._Lizard_Island_37|Cnidarian-Anthozoa
IKADMKMKLRMVGAVNGHKFEIAGEGKGKPFEGKQTMDLKVLGGPLPFAYDILTTVFGNR
VFVKYPXDIXXYFKQSFPEGFSWQRSMAYEDGGICLATNDITLNGDCFLYEIRFDGVNFP
XNXPVMQKKTKWEPSTEKXFKTTYLYEAEAGLPRQAK-----------------------

>gi|257221233|gb|ACV52382.1||Lobophyllia_hemprichii|Cnidarian-Anthozoa
IKTDMKMKLRMVGAVNGHKFEIAGEGKGKPFEGKQTMNLEVLGGPLPFAFDILTTVFGNR
VFVKYPRDIADYFKQSFPEGFSWERSMAYEDGGICLATNDITLKGDCFLYEIRFDGVNFP
ANSPVLQKKT---

>gi|257221235|gb|ACV52383.1||Lobophyllia_hemprichii|Cnidarian-Anthozoa
IKTDMKMKLRMVGAVNGHKFEIAGEGKGKPFEGKQTMNLEVLGGPLPFAFDILTTVFGNR
VFVKYPRDIADYFKQSFPEGFSWERSMAYEDGGICLATNNITLMKGD-LYEIRFDGVNFP
ANSPVLQKKT---

>gi|595588282|pdb|4JC2|A|Alcyonium|Cnidarian-Anthozoa
IKQEMKIKLHMEGNVNGHAFVIEGDGKGKPYDGTQTLNLTVKGAPLPFSYDILTAAFXNR
AFTRYPADIPDYFKQTFPEGYSWERTMSYEDNAICNVRSEISMEGDCFTYKIRFDGKNFP
PNGPVMQKKTKWEPSTEKMYVRDGFLMGDVNMALLLDGGGHHRCDFKTSYKAKKVVQPDY
HFVDHRNEILSHDYSKVKLYENAVARYSLL
>gi|187564305|gb|ACD13195.1||Sarcophyton_sp._NA-2008|Cnidarian-Anthozoa
IKQEMKIKLHMDGNVNGHAFVIEGDGKGKPYDGTQTLNLSVKGAPLPFSYDILTNAFGNR
AFTKYPADIPDYFKQTFPEGYSWERTMSYEDNAICNVRSEISMEGDCFIYKIRFDGKNFP
PNGPVMQKKTKWEPSTEMMYARDGFLMGDVNMALLLEGGGHHRCDFKTSYKAAKNVQPDY
HYVDHRIEILSHDYNKVKLYENAVARYSLL
>gi|197209733|dbj|BAG68908.1||Sarcophyton_sp._sesoko01|Cnidarian-Anthozoa
IKQEMKIKLHMEGNVNGHAFVIEGDGKGKPYDGTQTLNLTVKGAPLPFSYDILTNAFGNR
AFTKYPADIPDYFKQTFPEGYSWERTMSYEDNAICNVRSEISMEGDCFIYKIRFDGKNFP

PNGPVMQKKTKWEPSTEMMYVRDGFLMGDVNMALLLEGGGHHRCDFKTSYK-AKVVQPDY
HYVDHRIEILSHDYSKVKLYENAVARYSLL
>gi|257221217|gb|ACV52374.1||Scleractinia_sp._Lizard_Island_30|Cnidarian-Anthozoa
IKQEMKIKLRMEGTVNGHSFVIEGEGKGKPYEGTQTINLTVKGAPLPFAYDILTSGFGNR
AFTKYPSDIPDYFKQTFPEGYSWERSMTYEDQGICTITSDIRMEDDCFMYEIRFKGENFS
PNGPVMQKKTKWEPSTEKMYVRDGVLKGDVNMALLLDGGGHYRCDFRSTYKAKKVVNPDY
HFVDHRIEILSHDYSKVKLYENAVARYSML
>gi|71318038|gb|AAM10625.3|AF420591_1|Dendronephthya_sp._SSAL-2002|Cnidarian-Anthozoa
IKEDMRVKVHMEGNVNGHAFVIEGEGKGKPYEGTQTLNLTVKGAPLPFSYDILTTALGNR
VFTKYPEDIPDYFKQSFPEGYSWERTMTYEDKGICTIRSDISLEGDCFFQNVRFNGMNFP
PNGPVMQKKTKWEPSTEKLHVRDGLLVGNINMALLLEGGGHYLCDFKTTYKAKKVVQPDY
HFVDHRIEILSNDYNKVKLYEHGVARYSPL
>gi|257221225|gb|ACV52378.1||Lobophyllia_hemprichii|Cnidarian-Anthozoa
IKPDMKIKLRMEGTVNGHSFVIDGDGKGKPFEGKQSMSLEVKGGPLPFSYDILTTAFGNR
VFAEYPDHIQDYFKQSFPKGYSWERSLTFEDGGICIARNDITLEGDTFYNKVRFYGVNFP
PNGPVMQKKTKWEPSTEKMYVRDGVLTGDITMALLLIGDVHYRCDFRTTYKARKGVKPGY
HFVKHRIEISSHDYNKVKLCEHAVAHSGLP
>gi|125395879|gb|ABN41777.1||Catalaphyllia_jardinei|Cnidarian-Anthozoa
IKEEMKIRLRMEGIVNGHAFVIEGNGNGHPFEGKQSMDLEVKGAPLPFAYDILTTAFGNR
VFAKYPGNIPDYFKQSFPKGFSWERSLIFEDGGICIATNDITMEGDTFVNKVRFYGVNFP
PNGPVMQKRTKWEASTEKMYVRDGVLTGDITMALLLKGDVHYRCDFRTTYKTNEDVKPGY
HFVDHCISILSHDYTKVKLYEHAVAHSGLP
>gi|149241730|pdb|2GW3|A|Trachyphyllia_geoffroyi|Cnidarian-Anthozoa
IKPEMKIKLLMEGNVNGHQFVIEGDGKGHPFEGKQSMDLVVKGAPLPFAYDILTTAFXNR
VFAKYPDHIPDYFKQSFPKGFSWERSLMFEDGGVCIATNDITLKGDTFFNKVRFDGVNFP
PNGPVMQKKTKWEASTEKMYLRDGVLTGDITMALLLKGDVHYRCDFRTTYKSREGVKPGY
HFVDHCISILRHDYNEVKLYEHAVAHSGLP
>gi|23503508|dbj|BAC20344.1||Trachyphyllia_geoffroyi|Cnidarian-Anthozoa
IKPEMKIKLLMEGNVNGHQFVIEGDGKGHPFEGKQSMDLVVKGAPLPFAYDILTTAFGNR
VFAKYPDHIPDYFKQSFPKGFSWERSLMFEDGGVCIATNDITLKGDTFFNKVRFDGVNFP
PNGPVMQKKTKWEASTEKMYLRDGVLTGDITMALLLKGDVHYRCDFRTTYKSREGVKPGY
HFVDHCISILRHDYNEVKLYEHAVAHSGLP
>gi|149241733|pdb|2GW4|B|Trachyphyllia_geoffroyi|Cnidarian-Anthozoa
---XNR
VFAKYPDHIPDYFKQSFPKGFSWERSLMFEDGGVCIATNDITLKGDTFFNKVRFDGVNFP
PNGPVMQKKTKWEASTEKMYLRDGVLTGDITMALLLKGDVHYRCDFRTTYKSREGVKPGY
HFVDHCISILRHDYNEVKLYEHAVAHSGLP
>gi|353251678|pdb|3P8U|A|Lobophyllia_hemprichii|Cnidarian-Anthozoa
IKPDMKINLRMEGNVNGHHFVIDGDGTGKPFEGKQSMDLEVKGGPLPFAFDILTTAFXNR
VFAEYPDHIQDYFKQSFPKGYSWERSLTFEDGGICIARNDITMEGDTFYNKVRFHGTNFP
ANGPVMQKKTKWEPSTEKMYVRDGVLTGDIHMALLLEGNAHYRCDFRTTYKAKKGVKPGY
HFVDHCIEILSHDYNKVKLYEHAVAHSGLP
>gi|196049670|pdb|2VVJ|A|Lobophyllia_hemprichii|Cnidarian-Anthozoa
IKPDMKINLRMEGNVNGHHFVIDGDGTGKPFEGKQSMDLEVKGGPLPFAFDILTTAXXNR
VFAEYPDHIQDYFKQSFPKGYSWERSLTFEDGGICIARNDITMEGDTFYNKVRFHGVNFP

ANGPVMQKKTKWEPSTEKMYVRDGVLTGDITMALLLEGNAHYRCDSRTTYKAKKGVKPGY
HLVDHCIEILSHDYNKVKLYEHAVAHSGLP
>gi|212374902|pdb|2VVH|A|Lobophyllia_hemprichii|Cnidarian-Anthozoa
IKPDMKINLRMEGNVNGHHFVIDGDGTGKPFEGKQSMDLEVKGGPLPFAFDILTTAFXNR
VFAEYPDHIQDYFKQSFPKGYSWERSLTFEDGGICIARNDITMEGDTFYNKVRFHGVNFP
ANGPVMQKKTKWEPSTEKMYVRDGVLTGDITMALLLEGNAHYRCDSRTTYKAKKGVKPGY
HLVDHCIEILSHDYNKVKLYEHAVAHSGLP
>gi|357380612|pdb|3S05|A|Lobophyllia_hemprichii|Cnidarian-Anthozoa
IKPDMKIKLRMEGNVNGHHFVIDGDGTGKPFEGKQSMDLEVKGGPLPFAFDILTTAFXNR
VFAKYPDNIQDYFKQSFPKGYSWERSLTFEDGGICIARNDITMEGDTFYNKVRFYGTNFP
ANGPVMQKKTKWEPSTEKMYVRDGVLTGDIHMALLLEGNAHYRCDFRTTYKAKKGVKPGY
HFVDHCIEILSHDYNKVKLYEHAVAHSGLP
>gi|357380701|pdb|3TMR|A|Lobophyllia_hemprichii|Cnidarian-Anthozoa
IKPDMKINLRMEGNVNGHHFVIDGDGTGKPFEGKQSMDLEVKGGPLPFAFDILTTAFXNR
VFAEYPDHIQDYFKQSFPKGYSWERSLTFEDGGICIARNDITMEGDTFYNKVRFHGVNFP
ANGPVMQKKTKWEPSTEKMYVRDGVLTGDITMALLLEGNAHYRCDSRTTYKAKKGVKPGY
HLVDHCIEILSHDYNKVKLYEHAVAHSGLP
>gi|529482534|pdb|2BTJ|A|Lobophyllia_hemprichii|Cnidarian-Anthozoa
IKPDMKINLRMEGNVNGHHFVIDGDGTGKPFEGKQSMDLEVKGGPLPFAFDILTTAXXNR
VFAEYPDHIQDYFKQSFPKGYSWERSLTFEDGGICIARNDITMEGDTFYNKVRFHGVNFP
ANGPVMQKKTKWEPSTEKMYVRDGVLTGDITMALLLEGNAHYRCDFRTTYKAKKGVKPGY
HFVDHCIEILSHDYNKVKLYEHAVAHSGLP
>gi|550545535|pdb|4LJD|A|Lobophyllia_hemprichii|Cnidarian-Anthozoa
IKPDMKINLRMEGNVNGHHFVIDGDGTGKPFEGKQSMDLEVKGGPLPFAFDILTTAFXNR
VFAEYPDHIQDYFKQSFPKGYSWERSLTFEDGGICIARNDITMEGDTFYNKVRFHGVNFP
ANGPVMQKKTKWEPSTEKMYVRDGVLTGDITXALLLEGNAHYRXDSRTTYKAKKGVKPGY
HLVDHCIEILSHDYNKVKLYEHAVAHSGLP
>gi|550545537|pdb|4LJD|C|Lobophyllia_hemprichii|Cnidarian-Anthozoa
IKPDMKINLRMEGNVNGHHFVIDGDGTGKPFEGKQSMDLEVKGGPLPFAFDILTTAFXNR
VFAEYPDHIQDYFKQSFPKGYSWERSLTFEDGGICIARNDITMEGDTFYNKVRFHGVNFP
ANGPVMQKKTKWEPSTEKMYVRDGVLTGDITMALLLEGNAHYRXDSRTTYKAKKGVKPGY
HLVDHCIEILSHDYNKVKLYEHAVAHSGLP
>gi|550545539|pdb|4LJC|A|Lobophyllia_hemprichii|Cnidarian-Anthozoa
IKPDMKINLRMEGNVNGHHFVIDGDGTGKPFEGKQSMDLEVKGGPLPFAFDILTTAFXNR
VFAEYPDHIQDYFKQSFPKGYSWERSLTFEDGGICIARNDITMEGDTFYNKVRFHGVNFP
ANGPVMQKKTKWEPSTEKMYVRDGVLTGDITMALLLEGNAHYRCDSRTTYKAKKGVKPGY
HLVDHCIEILSHDYNKVKLYEHAVAHSGLP
>gi|55667942|gb|AAV54099.1||Lobophyllia_hemprichii|Cnidarian-Anthozoa
IKPDMKINLRMEGNVNGHHFVIDGDGTGKPFEGKQSMDLEVKGGPLPFAFDILTTAFGNR
VFAEYPDHIQDYFKQSFPKGYSWERSLTFEDGGICIARNDITMEGDTFYNKVRFHGVNFP
ANGPVMQKKTKWEPSTEKMYVRDGVLTGDITMALLLEGNAHYRCDFRTTYKAKKGVKPGY
HFVDHCIEILSHDYNKVKLYEHAVAHSGLP
>gi|71042513|pdb|1ZUX|A|Lobophyllia_hemprichii|Cnidarian-Anthozoa
IKPDMKINLRMEGNVNGHHFVIDGDGTGKPFEGKQSMDLEVKGGPLPFAFDILTTAFXNR
VFAEYPDHIQDYFKQSFPKGYSWERSLTFEDGGICIARNDITMEGDTFYNKVRFHGVNFP
ANGPVMQKKTKWEPSTEKMYVRDGVLTGDITMALLLEGNAHYRCDFRTTYKAKKGVKPGY

HFVDHCIEILSHDYNKVKLYEHAVAHSGLP
>gi|753534389|pdb|4R6B|A|Lobophyllia_hemprichii|Cnidarian-Anthozoa
IKPDMKINLRMEGNVNGHHFVIDGDGTGKPFEGKQSMDLEVKGGPLPFAFDILTTAFXNR
VFAEYPDHIQDYFKQSFPKGYSWERSLTFEDGGICIARNDITMEGDTFYNKVRFHGVNFP
ANGPVMQKKTKWEPSTEKMYVRDGVLTGDITAALLLEGNAHYRCDSRTTYKAKKGVKPGY
HLVDHCIEILSHDYNKVKLYEHAVAHSGLP
>gi|51593112|gb|AAU06843.1||Scolymia_cubensis|Cnidarian-Anthozoa
VKQFMKIKMSLEGTVNGHYFKIVGEGDGTPFEGKQTLHLKVKGAPLPFAYDILTTALGNR
VFVEYPENIPDYFKQSFPKGYSWERSLTFEDGGICIARSDIKMVGDTFHNEVQFYGVNFP
ANGPVMQRHTKWEPSTEKIYVRDGVLTGDITMALLLKGGTHYRCDFRTTYKAKKGPKPGY
HLVDHCIEITSHDYNVVELYEHAVAHSGLP
>gi|78059164|gb|ABB17959.1||Mycedium_elephantotus|Cnidarian-Anthozoa
IKPDMKLKLRMEGTVNGHYFVIEGDGKGRPFEGKQSMDLDVKGGPLPFAYDILTTAFGNR
VFAEYPDHIPDYFKQSFPGGYSWERSLTFEDGGICIARNDIKMVGDTFYNTVRFDGVNFP
PNGPVMQRRTKWEPSTEKIYVRDGVLTGDITMALLLEGGVHYRCDFRTTYKAKKGVQPGY
HFVDHCIEILSHDYNKVKLYEHAVAHSGLP
>gi|78059166|gb|ABB17960.1||Echinophyllia_echinata|Cnidarian-Anthozoa
INPEMKIKLLMEGNVNGHPFVIEGDGKGHPFEGKQSMDLVVKGAPLPFAYDILTTAFGNR
VFAKYPDHIPDYFKQSFPNGFSWERSLMFEDGGVCIATNDITLEGDTFFNKVRFYGVNFP
PNGPVMQKKTKWEASTEKMYLRDGVLTGDITMALLLKGDVHYRCDFRTTYKSREGVKPGY
HFVDHCISIVSHDYTKVKLYEHAVAHLGLP
>gi|78059156|gb|ABB17955.1||Mycedium_elephantotus|Cnidarian-Anthozoa
IKTDMKIRLQMQGAVNGHPFVITGEGEGKPYEGKHTINLTVQGGPLPFAFDILTTAFGNR
VFTKYPKDIPDYFKQSFPAGYSWERCMTFEDGGLCTVSSHIKIEGDYFTYDIRFHGVNFP
AGGPVMQKKTRWEPSTENMYVRDGVLVGEVERTLLLEGNKHHRCNFRTTYKAKKEVVPEY
HFVDHRIEILGHDYNNVVVYENAQQASTLP
>gi|11890751|gb|AAG41205.1|AF322221_1|Anemonia_sulcata|Cnidarian-Anthozoa
IKETMRVQLSMEGSVNYHAFKCTGKGEGKPYEGTQSLNITITGGPLPFAFDILSHAFGIK
VFAKYPKEIPDFFKQSLPGGFSWERVSTYEDGGVLSATQETSLQGDCIICKVKVLGTNFP
ANGPVMQKKTGWEPSTETVIPRDGGLLLRDTPALMLADGGHLSCFMETTYKSKKEVKPEL
HFHHLRMEKLNISWKTVEQHESVY--SQVP
>gi|126030169|pdb|2C9I|A|Anemonia_sulcata|Cnidarian-Anthozoa
IKETMRVQLSMEGSVNYHAFKCTGKGEGKPYEGTQSLNITITGGPLPFAFDILSHAFXIK
VFAKYPKEIPDFFKQSLPGGFSWERVSTYEDGGVLSATQETSLQGDCIICKVKVLGTNFP
ANGPVMQKKTGWEPSTETVIPRDGGLLLRDTPALMLADGGHLSCFMETTYKSKKEVKPEL
HFHHLRMEKLNISWKTVEQHESVY--SQVP
>gi|42521556|gb|AAS18272.1||Astrangia_lajollaensis|Cnidarian-Anthozoa
IKKEMTMDYVMDGCVNGHSFTVKGDGAGKPYEGHQRLSLHVTGQPLPFAFDILSAAFGNR
CFTKYPKEIPDMFKQAFPGGMSWERTMTFEDGGVASVSADISIEKDG-KHRSKFVGVNFP
VNGPVMQNKTGWEPSIEKMTVRDGTLKGHVTMFLKLEGGGNYRCDFETTYKAKKAVKLDS
HFIEHRLVTTIR-GNNVELQEAR--NSGLG
>gi|471179751|gb|AGI04877.1||Agaricia_fragilis|Cnidarian-Anthozoa
--LPFSFDILTPAFGNR
VFTKYPKEIPDYFKQTFPEGYHWERSIPFQDQASCTVTSHI------------------

>gi|471179771|gb|AGI04887.1||Agaricia_fragilis|Cnidarian-Anthozoa
---LPFSFXXXTPAFGNR
VFTKYPKEIPDYFKQTFPEGYHWKRSIPFQDQASCTVTSHI-------------------

>gi|471179818|gb|AGI04910.1||Agaricia_lamarcki|Cnidarian-Anthozoa
---LPFSFXXLTPAFGNR
VFTKYPKEIPDYFKQTFPEGYHWERSIPFQDQASCTVTSHI-------------------

>gi|78059186|gb|ABB17970.1||Eusmilia_fastigiata|Cnidarian-Anthozoa
VLNDVTMTYFMEGSVNGHDFTIEGEGTGKPYEGHQRFNLRVTGAPLPFAVDILSAAFGNR
CFTKYPKEIPDFFKQSLPEDMSWERTMTFEDGGIVAISAHIRLIGNRFEHKSKFVGVNFP
ADGPVMQRKTGWEPSSEKMTPRDGILKGYVPSFLVLQGGGNYRCDYDTSYRAMKPVEPGG
HFIQHRIVRRDIKGNTWQIQEDAN--SEVP
>gi|471179686|gb|AGI04847.1||Agaricia_agaricites|Cnidarian-Anthozoa
---LPFSFXXXXXXFGNR
VFTXYPPNIPDYFKQTFPEGYHWERNIPFEDQAACTVTSHI-------------------

>gi|471179656|gb|AGI04832.1||Agaricia_agaricites|Cnidarian-Anthozoa
---LPFSFXXXTXAFGNR
VFTXXPPNIPDYFKQTFPEGYHWERNIPFEDQAACSVTSHI-------------------

>gi|471179664|gb|AGI04836.1||Agaricia_agaricites|Cnidarian-Anthozoa
---LPFSFXXXXXAFGNR
VFTXXPPNIPDYFKQTFPEGYHWERNIPFEDQAACTVTSHI-------------------

>gi|471179666|gb|AGI04837.1||Agaricia_agaricites|Cnidarian-Anthozoa
---LPFSFXXXTPAFGNR
VFTXXPPNIPDYFKQTFPEGYHWERNIPFEDQAACSVTSHI-------------------

>gi|471179678|gb|AGI04843.1||Agaricia_agaricites|Cnidarian-Anthozoa
---LPFSFXXXXXXFGNR
VFTXXPPNIPDYFKQTFPEGYHWERNIPFEDQAACSVTSHI-------------------

>gi|471179682|gb|AGI04845.1||Agaricia_agaricites|Cnidarian-Anthozoa
---LPFSFXXXTXAFGNR
VFTXXPPNIPDYFKQTFPEGYHWERNIPFEDQAACTVTSQI-------------------

>gi|471179660|gb|AGI04834.1||Agaricia_agaricites|Cnidarian-Anthozoa

--LPFSFXXXTPAFGNR
VFTKYPPNIPDYFKQTFPEGYHWERNIPFEDQAACTVTSHI-------------------

>gi|471179668|gb|AGI04838.1||Agaricia_agaricites|Cnidarian-Anthozoa
--LPFSFXXLTPAFGNR
VFTKYPPNIPDYFKQTFPEGYHWERNIPFEYQAACTVTSHI-------------------

>gi|471179688|gb|AGI04848.1||Agaricia_agaricites|Cnidarian-Anthozoa
--LPFSFXXLTPAFGNR
VFTKYPPNIPDYFKQTFPEGYHWERNIPFEDQAACTVTSHI-------------------

>gi|471179694|gb|AGI04851.1||Agaricia_agaricites|Cnidarian-Anthozoa
--LPFSFXXLTPAFGNR
VFTKYPPNIPDYFKQTFPEGYHWERNIPFEDQAACSVTSHI-------------------

>gi|471179730|gb|AGI04867.1||Agaricia_agaricites|Cnidarian-Anthozoa
--LPFSFXXXTPAFGNR
VFTKYPPNIPDYFKQTFPEGYHWERNIPFEDQAACSVTSHI-------------------

>gi|471179736|gb|AGI04870.1||Agaricia_agaricites|Cnidarian-Anthozoa
--LPFSFXXXTPAFGNR
VFTKYPPNIPDYFKQTFPEGYYWERNIPFEDQAACTVTSQI-------------------

>gi|471179662|gb|AGI04835.1||Agaricia_agaricites|Cnidarian-Anthozoa
--LPFSFXXXTPAFGNR
AFTXYPPNVPDYFKQTFPEGYHWERNIPFEDQAACTVTSHI-------------------

>gi|471179674|gb|AGI04841.1||Agaricia_agaricites|Cnidarian-Anthozoa
--LPFSFXXXXPAFGNR
VFTXYPPNIPDYFKQTFPEGYHWERNIPFEDQAACTVTSQI-------------------

>gi|471179684|gb|AGI04846.1||Agaricia_agaricites|Cnidarian-Anthozoa
--LPFSFXXXXXAFGNR
VFTXXPPNTPDYFKQTFPEGYHWERNIPFEDQAACTVTSHI-------------------

>gi|471179718|gb|AGI04861.1||Agaricia_agaricites|Cnidarian-Anthozoa
--LPFSFXXXXXAFGNR

VFTXXPPNIPDYFKQTFPEGYHWERNIPFEDQAACSVTSHI------------------

>gi|471179720|gb|AGI04862.1||Agaricia_agaricites|Cnidarian-Anthozoa
--LPFSFXXXXXAFGSK
VFTXXPPNIPDYFKQTFPEGYHWERNIPFEDQAACSVTSHI------------------

>gi|471179732|gb|AGI04868.1||Agaricia_agaricites|Cnidarian-Anthozoa
--LPFSFXXXXXAFGNR
VFTXXPTDIPDYFKQTFPEGYRWERSIPFEDQASCTVTSHT------------------

>gi|471179769|gb|AGI04886.1||Agaricia_fragilis|Cnidarian-Anthozoa
--LPFSFXXXTPAFGNR
VFTXXPKEIPDYFKQTFPEGYHWERSIPFQDQASCTVTSHI------------------

>gi|471179777|gb|AGI04890.1||Agaricia_fragilis|Cnidarian-Anthozoa
--LPFSFXXXXXAFGNR
VFTXXPSNIPDFFKRTFPEGYHWERIMPFEDQAVCTVTSHI------------------

>gi|471179816|gb|AGI04909.1||Agaricia_lamarcki|Cnidarian-Anthozoa
--LPFSFXXXXXALGNR
VFTXXPSNIPDFFKQTFPEGYHWERIMPFEDQAVCTVTSHI------------------

>gi|471179865|gb|AGI04933.1||Helioseris_cucullata|Cnidarian-Anthozoa
--LPFSFDXXXXXFGNR
AFTXXPADIPDFFKQSFPEGYYWERNITFEDQAACTVTSQI------------------

>gi|471179742|gb|AGI04873.1||Agaricia_agaricites|Cnidarian-Anthozoa
--LPFSFDILTPAFGNR
VFTKYPPNIPDYFKQTFPEGYHWERNIPFEDQAACTVTSHI------------------

>gi|21303774|gb|AAK71331.1||Agaricia_fragilis|Cnidarian-Anthozoa
P---LPFSFDILTPAFGNR
VFTKYPPNIPDYFKQTFPEGYHWERNIPFEDQAACTVTSHIRLENVRRILKWEPSTENYP
RDGFLEGHVDEGGGYYRAETEHDENVEHDVARYMLPTM---------------------

>gi|471179670|gb|AGI04839.1||Agaricia_agaricites|Cnidarian-Anthozoa
---DILTPAFGNR
VFTKYPTDIPDYFKQTFPEGYRWERSIPFQDQASCTVTSHI------------------

>gi|471179692|gb|AGI04850.1||Agaricia_agaricites|Cnidarian-Anthozoa
--DILTPAFGXX
XFTKYPPNIPDYLKQTFPEGYHWERNIPFEDQAACTVTSHI-------------------

>gi|471179714|gb|AGI04859.1||Agaricia_agaricites|Cnidarian-Anthozoa
---ILTPAFGXX
XFTKYPPNIPDYFKQTFPEGYHWERNIPFEDQAACSVTSHI-------------------

>gi|471179746|gb|AGI04875.1||Agaricia_agaricites|Cnidarian-Anthozoa
---LPFSFXXXTPAFGNR
VFTKYPTDIPDYFKQTFPEGYRWERSIPFQDQASCTVTSHI-------------------

>gi|471179748|gb|AGI04876.1||Agaricia_agaricites|Cnidarian-Anthozoa
---XDILTPAFGNR
VFTKYPPNIPDYFKQTFPEGYHWERNIPFEDQAACTVTSQI-------------------

>gi|471179799|gb|AGI04901.1||Agaricia_lamarcki|Cnidarian-Anthozoa
---LPFSFDILTPAFGNR
VFTKYPTDIPDYFKQTFPEGYRWERSIPFQDQASCTVTSHI-------------------

>gi|471179824|gb|AGI04913.1||Agaricia_lamarcki|Cnidarian-Anthozoa
---LPFSFXXXXXAFGNR
VFTKYPENIPDYFKQTFPEGYHWERSVPFEDQAACTVTSHI-------------------

>gi|75238521|gb|AAU04450.2||Pocillopora_damicornis|Cnidarian-Anthozoa
P--LPFSFDILSSAFGNR
CFTKYPADMPDYFKEAFPAGMSFERTFTFEDGGVATASGHICLEMFHRTIGWDPSFEKTV
SNNILRGDVTKGGGYHSCQDIEDKLLETAVAHVNPL------------------------

>gi|471179654|gb|AGI04831.1||Agaricia_agaricites|Cnidarian-Anthozoa
---LPFSFDXLTTAFGNR
VFTKYPSNIPDFFKQTFPEGYHWERIMPFEDQAVCTVTSHI-------------------

>gi|471179698|gb|AGI04853.1||Agaricia_agaricites|Cnidarian-Anthozoa
---LPFSFXXXTTAFGNR
VFTKYPSNIPDFFKQTFPEGYHWERIMPFEDQAVCTVTSHI-------------------

>gi|471179701|gb|AGI04854.1||Agaricia_agaricites|Cnidarian-Anthozoa
---LPFSFXXLTTAFGNR
VFTKYPSNIPDFFKQTFPEGYHWERIMPFEDQAVCTVTSHI-------------------

>gi|471179781|gb|AGI04892.1||Agaricia_fragilis|Cnidarian-Anthozoa
---LPFSFDILTTAFGNR
VFTKYPSTIPDFFKQTFPEGYHWERIMPFEDQAVCTVTSHI-------------------

>gi|471179836|gb|AGI04919.1||Agaricia_lamarcki|Cnidarian-Anthozoa
---LPFSFDILTTAFGNR
VSTKYPSNIPDFFKQTFPEGYHWERIMPFEDQAVCTVTSHI-------------------

>gi|471179840|gb|AGI04921.1||Agaricia_lamarcki|Cnidarian-Anthozoa
---LPFSFDILTTAFGNR
VFTKYPSNIPDFFKQTFPEGYHWERIMPFEDQAVCTVTSHI-------------------

>gi|471179849|gb|AGI04925.1||Agaricia_lamarcki|Cnidarian-Anthozoa
---LPFSFDILTTAFGNR
VFTKYPSNIPDFFKQTFPEGYHWERIMPFEDQAACTVTSHI-------------------

>gi|21303782|gb|AAK71341.1||Agaricia_agaricites|Cnidarian-Anthozoa
P--LPFSFDILTTAFGNR
VFTKYPSNIPDFFKQTFPEGYHWERIMPFEDQAVCTVTSHIRLEFVDMQRKIMRWEPSEN
MYPRNGLLEGFRLKGGGYYMITNHYTNELRALAYSTLPPI--------------------

>gi|471179672|gb|AGI04840.1||Agaricia_agaricites|Cnidarian-Anthozoa
---ILTTAFGXX
XFTKYPSNIPDFFKQTFPEGYHWERIMPFEDQAVCTVTSHI-------------------

>gi|471179690|gb|AGI04849.1||Agaricia_agaricites|Cnidarian-Anthozoa
---LPFSFXXLTTAFGNX
XFTKYPSNIPDFFKQTFPEGYHWERXXPFEDQAVCTVTSHI-------------------

>gi|471179707|gb|AGI04857.1||Agaricia_agaricites|Cnidarian-Anthozoa
---LPFSFXXXTTAFGNR
VFTKYPSNIPDFFKQTFPEGYHGERIMPFEDQAVCTVTSHI-------------------

>gi|471179710|gb|AGI04858.1||Agaricia_agaricites|Cnidarian-Anthozoa
---LPFSFDXXXXAFGNR
VFTKYPSNIPDFFKQTFPEGYHWERIMPFEDQAVCTVTSHI------------------

>gi|471179734|gb|AGI04869.1||Agaricia_agaricites|Cnidarian-Anthozoa
---LPFSFXXXXTAFGNR
VFTKYPSNIPDFFKQTFPEGYHWERIMPFEDQAVCTVTSHI------------------

>gi|471179753|gb|AGI04878.1||Agaricia_fragilis|Cnidarian-Anthozoa
---LPFSFDILTTAFGNR
VFTKYPSNIPDFFKQTFPEGYHWERVMPFEDQAVCTVTSYI------------------

>gi|471179755|gb|AGI04879.1||Agaricia_fragilis|Cnidarian-Anthozoa
---LPFSFDILTTAFGNR
VFTKYPSNIPDFFKQTFPEGYHWERIMPFEDQAVCTVTSYI------------------

>gi|471179757|gb|AGI04880.1||Agaricia_fragilis|Cnidarian-Anthozoa
---LPFSFDILTTAFGNR
VFTKYPSNIPDFFKQTFPEGYHWERIMPFEDQAVCTVTSHI------------------

>gi|471179822|gb|AGI04912.1||Agaricia_lamarcki|Cnidarian-Anthozoa
---LPFSFDXLTTAFGNR
VFTKYPSNIPDYFKQTFPEGYHWERSVPFEDQAVCTVTSHI------------------

>gi|51593140|gb|AAU06857.1||Agaricia_fragilis|Cnidarian-Anthozoa
IVKEMMTKLHMEGTVNGHAFTIEGKGKGDPYNGVQSMNLDVKGAPLPFSFDLLTPAFGNR
VFTKYPEDIPDFFKQVFPEGYHWERSITFEDQAVCTATSHIRLDQKE-IYDVRFHGVNFP
ANGPIMQKKIGWEPSTEKMYARDGVLKGDVNMTLRVEGGGHYRADFRTTYKAKKPVNPGY
HFIDHRIEITKHSYTNVALYEAAVARHSPL
>gi|187564264|gb|ACD13192.1||Acropora_eurystoma|Cnidarian-Anthozoa
IVQEMKTKYHMEGSVNGHEFTIEGVATGYPYEGKQMSELVIIGKPLPFSFDILSSVFGNR
CFTKYPADIPDYFKQAFPDGMSYERSFLFEDGAVATASWNIRLEGNCFIHNSIFHGVNFP
ADGPVMKKQTGWDKSFEKMTVSKEVLRGDVTMFLMLEGGGYHRCQFHSTYKTEKPVTPPN
HVVEHQIVRTDLGGFTVKLEALAAAHVNPL
>gi|199581453|gb|ACH89428.1||Acropora_millepora|Cnidarian-Anthozoa
IVQEMKTKYHMEGSVNGHEFTIEGVGTGYPYEGKQMSELVIIGKPLPFSFDILSSVFGNR
CFTKYPADMPDYFKQAFPDGMSYERSFLFEDGAVATASWKIRLEGNCFIHNSIFHGVNFP
ADGPVMKKKTGWDKSFEKMTVSKEVLRGDVTMFLMLEGGGSHRCQFHSTYKTEKPVTPPN
HVVEHQIVRTDLGGFTVKLEAHAAAHVNPL
>gi|383872166|tpd|FAA00739.1||Acropora_digitifera|Cnidarian-Anthozoa

IVQEMKTKYHMEGTVNGHEFTIEGVATGYPYEGKQMSELVIIGKPLPFSFDILSSVFGNR
CFTKYPADMPDYFKQAFPDGMSYERSFLFEDGAVATASWNIRLEGNCFIHKSIFHGVNFP
ADGPVMKKKTDWDKSFEKMTVSKEVLRGDVTMFLMLEGGGSHRCQFHSTYKTEKPVTPPN
HVVEHQIVRTDLGGFTVKLEAHAAAHVNPL
>gi|51593116|gb|AAU06845.1||Acropora_aculeus|Cnidarian-Anthozoa
IVQEMKTKYRMEGSVNGHEFTIEGVGTGYPYEGKQMSELVIIGKPLPFSFDILSSVFGNR
CFTKYPADMPDYFKQAFPDGMSYERSFLFEDGAVATASWNIRLEGNCFIHNSIFHGVNFP
ADGPVMKKKTGWDKSFEKMTVSKEVLRGDVTMFLMLEGGGYHRCQFHSTYKTEKPVEPPN
HVVEHQIVRTDLGGFTVKLEAHAAAHVNPL
>gi|51593118|gb|AAU06846.1||Acropora_millepora|Cnidarian-Anthozoa
IVQEMKTKYHMEGSVNGHEFTIEGVGTGYPYEGKQMSELVIIGKPLPFSFDILSSVFGNR
CFTKYPADMPDYFKQAFPDGMSYERSFLFEDGAVATASWNIRLEGNCFIHKSIFHGVNFP
ADGPVMKKKTDWDKSFEKMTVSKEVLRGDVTMFLMLEGGGSHRCQFHSTYKTEKPVTPPN
HVVEHQIVRTDLGGFTVKLEAHAAAHVNPL
>gi|51593120|gb|AAU06847.1||Acropora_nobilis|Cnidarian-Anthozoa
IAQEMKTKYHMEGSVNGHEFTIEGVGTGYPYEGKQMSELVIIGKPLPFSFDILSSVFGNR
CFTKYPADMPDYFKQAFPDGMSYERSFLFEDGAVATASWKIRLEGNCFIHNSIFNGVNFP
ADGPVMEKKTGWDKSFEKMTVSKEVLRGDVTMFLMLEGGGSHRCQFHSTYKTEKPVTPPN
HVVEHQIVRTDLGGFTVKLEAHAAAHVNPL
>gi|78059190|gb|ABB17972.1||Danafungia_horrida|Cnidarian-Anthozoa
IVQEMKTKYRMEGSVNGHEFTIEGVGTGYPYEGKQMSELVIIGKPLPFSFDILSSVFGNR
CFTKYPADMPDYFKQAFPDGMSYERSFLFEDGAVATASWNIRLEGNCFIHNSIFHGVNFP
ADGPVMKKKTGWDKSFEKMTVSKEVLTGDVTMFLMLEGGGYHRCQFHSTYKTEKPVEPPN
HVVEHQIVRTDLGGFTVKLEAHAAAHVNPL
>gi|199581445|gb|ACH89424.1||Acropora_pulchra|Cnidarian-Anthozoa
IVQVMKTKYHMEGSVNGHEFTIEGVGTGNPYEGTQMSELVITGKPLPFSFDILSTVFGNR
CFTKYPEGMTDYFKQAFPDGMSYERSFLYEDGGVATASWNIRLERDCFIHKSIYHGVNFP
ADGPVMKKKTGWDKAFEKMTVSKDVLRGDVTEFLMLEGGGYHSCQFHSTYKPEKPAAPPN
HVVEHHIVRTDLGGFTVKLEEHAAAHVNPL
>gi|197253915|gb|ACH53606.1||Acropora_millepora|Cnidarian-Anthozoa
IAQVMKTKYHMEGSVNGHEFTIEGVGTGNPYEGTQMSELVIIGKPLPFSFDILSTVFGNR
CFTKYPEGMTDYFKQAFPDGMSYERSFLYEDGGVATASWNIRLERDCFVHKSIYHGVNFP
ADGPVMKKKTGWDKAFEKMTVSKDVLRGDVTEFLMLEGGGYHSCQFHSTYKPEKPGTPPN
HVVEHHIVRTDLGGFAVKLEEHAAAHVNP-
>gi|199581449|gb|ACH89426.1||Acropora_millepora|Cnidarian-Anthozoa
IVQEMKTKYHMEGSVNGHEFTIEGVGTGNPYEGTQMSELVITGKPLPFSFDILSTVFGNR
CFTKYPEGMTDYFKQAFPDGMSYERSFLYEDGGVATASWNIRLERDCFIHKSIYHGVNFP
ADGPVMKKKTGWDKAFEKMTVSKDVLRGDVTEFLMLEGGGYHSCQFHSTYKPEKPVTPPN
HVVEHHIVRTDLGGFTVKLEEHAAAHVNPL
>gi|199581451|gb|ACH89427.1||Acropora_millepora|Cnidarian-Anthozoa
IVQAMKTKYHMEGSVNGHEFTIEGVGTGNPYEGTQMSELVITGKPLPFSFDILSTVFGNR
CFTKYPEGMTDYFKQAFPDGMSYERSFLFEDGGVATASWNIRLEGDCFIHKSIYHGVNFP
ADGPVMKKKTGWEKSFEKMTVSKDVLRGDVTMFLMLEGGGYHRCQFHSTYKPEKPGTPPN
HVVEHHIVRTDLGGFTVKLEEHAAAHVNPL
>gi|51593124|gb|AAU06849.1||Acropora_millepora|Cnidarian-Anthozoa
IAQVMKTKYHMEGSVNGHEFTIEGVGTGNPYEGTQMSELVITGKPLPFSFDILSTVFGNR

CFTKYPEGMTDYFKQAFPDGMSFERSFLYEDGGVATASWNIRLERDCFIHKSIYHGVNFP
ADGPVMKKKTGWDKAFEKMTVSKDVLRGDVTEFLMLEGGGYHSCQFHSTYKPEKPVTPPN
HVVEHHIVRTDLGGFTVKLEEHAAAHVNPL
>gi|51593126|gb|AAU06850.1||Acropora_nobilis|Cnidarian-Anthozoa
IAQVMKTKYHMEGSVNGHEFTIEGVGTGNPYEGTQMSELVITGKPLPFSFDILSTVFGNR
CFTKYPEGMTDYFKQAFPDGMSYERSFLYEDGGVATAGWNIGLERDCFIHKSIYHGVNFP
ADGPVMKKKTGWDKAFEKMTVSKDVLRGDVTGFLMLEGGGYHSCQFHSTYKPEKPAAPPN
HVVEHHIVRTDLGGFTVKLEEHAAAHVNPL
>gi|51593128|gb|AAU06851.1||Acropora_nobilis|Cnidarian-Anthozoa
IAQVMKTKYHMEGSVNGHEFTIEGVGTGNPYEGTQMSELVITGKPLPFSFDILSTVFGNR
CFTKYPEGMTDYFKQAFPDGMSYERSFLYEDGGVATASWNIRLERDCFIHKSIYHGVNFP
ADGPVMKKKTGWDKAFEKMTVSKDVLRGDVTEFLMLEGGGYHSCQFHSTYKPEKPAAPPN
HVVEHHIVRTDLGGFTVKLEEHAAAHVNPL
>gi|383212107|dbj|BAM08940.1||Acropora_tenuis|Cnidarian-Anthozoa
----MRTKYHMEGSVNGHEFTIEGVGTGNPYEGTQTAELVIIGKPLPFSFDILSSVFGNL
CFTKYPADMTDYFKQAFPAGMSYERSFLFEDGAVATASWNIRLEGNCFIHNSIFHGVNFP
ADGPVMKKKTGWDKSFQKMTVSKEVLRGDLAMFLLLEGGGYHRCQFHSTYKTERPVTPPN
HVVEHQIVRTDLGGFTVKLEEHAAAHVNPL
>gi|383872164|tpd|FAA00738.1||Acropora_digitifera|Cnidarian-Anthozoa
----MKTKYHMEGSVNGHEFTIEGVGTGNPYEGTQMSELVITGKPLPFSFDILSTVFGNR
CFTKYPEGMTDYFKQAFPDGMSYESLE-----------------RDCFIHKSIYHGVNFP
ADGPVMKKKTGWDKAFEKMTVSKDVLRGDVTEFLMLEGGGYHSCQFHSTYKPEKPATPPN
HVVEHHIVRTDLGGFTVKLEEHAAAHVNPL
>gi|383872168|tpd|FAA00740.1||Acropora_digitifera|Cnidarian-Anthozoa
----MKTKYHMEGSVNGHEFTIEGVGTGNPYEGTQLSELVIIGKPLPFSFDILSTAFGNR
CFTKYPEGMTDYFKQAFPEGMSYERSFVFEDGGVATASWTIRLEGNCFIHNSIYHGVNFP
ADGPVMKKKTGWDKSFEKMTVSKEVLRGDVTQFLQLEGGGYHKCQFRSTYKTEKPVTPPN
HVVEHHIVRTDLGGFMVKLEEQAAAHVSPL
>gi|383872170|tpd|FAA00741.1||Acropora_digitifera|Cnidarian-Anthozoa
----MRTKYHMEGSVNGHEFTIEGVGTGNPYEGKQMSELVIIGKPLPFSFDILSTAFGNL
CFTKYPADMPDYFKQGFPDGMSYERSFLFEDGGVATASWNIRLEGNCFIHNSIFHGVNFP
ADGPVMKKKTGWDKSFQKMTVSKELLRGDVTQFLLLEGGGYHRCQFHSTYKTEKPVTPPN
HVIEHHIVRTDLGGFTVKLEEHAAAHVNPL
>gi|383872172|tpd|FAA00742.1||Acropora_digitifera|Cnidarian-Anthozoa
---------------------------------MSELVIIGKPLPFSFEILSSVFGNR
CFTKYPEGMPDYFKQGFPDGMSYERSFMFEDGGVATASWTIRLEGNCFIHKSIFHGVNFP
ADGPVMKKKTGWDKSFEKMTVSKGVLRGDVTMFLLLEGGGYHSCQFDSTFKTEKPVTPPN
HVIEHHIVRTDLGGFTVKLEERAAAHVNPL
>gi|383872174|tpd|FAA00743.1||Acropora_digitifera|Cnidarian-Anthozoa
----
--------MPDYFKQGFPDGMSYERSFMFEDGGVATASWTIRLEGNCFIHKSIFHGVNFP
ADGPIMQKKTGWDESFEKMTVSKEVLRGDVTMFLMLEGGGYHRCQFDSTFKTEKPVTPPN
HVIEHHIARTDLGGFAVKLDGQAAAHVNPL
>gi|49257065|dbj|BAD24723.1||Acropora_sp._#30|Cnidarian-Anthozoa
IAQEMRTKYRMEGSVNGHEFTIEGVGTGNPYEGKQMSELVIIGKPLPFSFDILSTAFGNR
CFTKYPADMPDYFKQAFPDGMSYERSFLFEDGGVATASWSIRLEGNCFIHNSIYHGVNFP

ADGPVMKKQTGWDKSFEKMSVAKEVLRGDVTQFLLLEGGGYQRCRFHSTYKTEKPVAPPS
HVVEHQIVRTDLGGFKVKLEEHAEAHVNPL
>gi|51593122|gb|AAU06848.1||Acropora_aculeus|Cnidarian-Anthozoa
ITQEMPTKYHMKGSVNGHEFEIEGVGTGHPYEGTHMAELVIIGKPLPFSFDILSTVIGNR
CFTKYPADLPDYFKQAYPGGMSYERSFVYQDGGIATASWNVGLEGNCFIHKSTYLGVNFP
ADGPVMTKKTGWDKAFEKMTGFNEVLRGDVTEFLMLEGGGYHSCQFHSTYKPEKPVEPPN
HVIEHHIVRTDLGGFMVKLVQHAAAHVNPL
>gi|75766021|pdb|2A46|A|Anemonia_majano|Cnidarian-Anthozoa
IGDDMKMTYHMDGCVNGHYFTVKGEGNGKPYEGTQTSTFKVTGGPLAFSFDILSTVFXNR
CFTAYPTSMPDYFKQAFPDGMSYERTFTYEDGGVATASWEISLKGNCFEHKSTFHGVNFP
ADGPVMAKKTGWDPSFEKMTVCDGILKGDVTAFLMLQGGGNYRCQFHTSYKTKKPVTPPN
HVVEHRIARTDLDGNSVQLTEHAVAHITSV
>gi|56749103|sp|Q9U6Y6.1|GFPL_ANEMA|Anemonia_majano|Cnidarian-Anthozoa
IGDDMKMTYHMDGCVNGHYFTVKGEGNGKPYEGTQTSTFKVTGGPLAFSFDILSTVFGNR
CFTAYPTSMPDYFKQAFPDGMSYERTFTYEDGGVATASWEISLKGNCFEHKSTFHGVNFP
ADGPVMAKKTGWDPSFEKMTVCDGILKGDVTAFLMLQGGGNYRCQFHTSYKTKKPVTPPN
HVVEHRIARTDLDGNSVQLTEHAVAHITSV
>gi|75766022|pdb|2A47|A|Anemonia_majano|Cnidarian-Anthozoa
IGDDMKMTYHMDGCVNGHYFTVKGEGNGKPYEGTQTSTFKVTGGPLAFSFDILSTVFXNR
CFTAYPTSMPDYFKQAFPDGMSYERTFTYEDGGVATASWEISLKGNCFEHKSTFHGVNFP
ADGPVMAKKTGWDPSFEKMTVCDGILKGDVTAFLMLQGGGNYRCQFHTSYKTKKPVTPPN
HVVETRIARTDLDGNSVQLTEHAVAHITSV
>gi|75766023|pdb|2A48|A|Anemonia_majano|Cnidarian-Anthozoa
IGDDMKMTYHMDGCVNGHYFTVKGEGNGKPYEGTQTSTFKVTGGPLAFSFDILSTVFXNR
CFTAYPTSMPDYFKQAFPDGMSYERTFTYEDGGVATASWEISLKGNCFEHKSTFHGVNFP
ADGPVMAKKTGWDPSFQKMTVCDGILKGDVTAFLMLQGGGNYRCQFHTSYKTKKPVTPPN
HVVEHRIARTDLDGNSVQLTEHAVAHITSV
>gi|78059172|gb|ABB17963.1||Stylocoeniella_sp._NOA-2005|Cnidarian-Anthozoa
IANEMTMTFHMDGCVNGHYFTIEGEGSGRPYEGKQMSKFKVTGGPLPFSFDILSSAFGNR
CFTAYPAGMHDYFKQAFPEGMSYERTFTFEDGGVATASGDISLKGNCFVHKSMFHGVNFP
ADGPVMKKKTGWDPSFEKMTVCNGILKGDVTMFLMLEDGKNYKCQFHTSYKTKKPVTPSN
HVVEHRIVRTNLDGNHVQLDEHAVAHVNPL
>gi|78059192|gb|ABB17973.1||Acropora_millepora|Cnidarian-Anthozoa
IVQEMKTKYHMEGSVNGHEFTIEGVGTGYPYEGKQISELVIIGKPLPFSFDILSSVFGNR
CFTKYPADMPDYFKQAFPDGMSYERSFLFEDGAVATASWNIRLEGNCFIHKSIFHGVNFP
ADGPVMKKKTDWDKSFEKMTVSKEVLRGDVTMFLMLEGGGSHRCQFHSTYKTEKPVTPPN
HVVEHQIVRTDLGGFTVKLEEHAAAHVSL-
>gi|257221247|gb|ACV52389.1||Scleractinia_sp._Lizard_Island_36|Cnidarian-Anthozoa
ISSVMNIKLRMEGEVNGHKFTVEGEGSGKPYEGTQTIDLEVTGGPLPFAFDILTTAFGNR
AFTKYPADIPDYFKESFPEGHSWVRTMVFDDGGVCDVTNDIRMYGNVFEYEITFHCNNFE
LSGPIMQKETKWEPSCEGMYAGKNVLLGDEFRHLLLVGGGHFRCDCSPSYSAKKRVEPKY
HFIDHRIEIVSHDYQKVKVYEIAEAKYSPL
>gi|257221245|gb|ACV52388.1||Scleractinia_sp._Lizard_Island_39|Cnidarian-Anthozoa
----MNIKLRMEGEVNGHKFTVEGEGSGKPYEGTQTIDLEVTGGPLPFAFDILTTAFGNR
AFTKYPADIPDYFKESFPEGHSWVRTMVFDDGGVCDVTNDIRMYGNVFEYETTFHCNNFD
PSGPVMQKETKWEPSCEGMYAGKNVLLGDEFRHLLLVGGGHFRCDFRSTYSARKRVEPEY

>gi|118138297|pdb|2IB5|A|Epiactis_japonica|Cnidarian-Anthozoa
ISDNVRIKLYXEGTVNNHHFXCEAEGEGKPYEGTQXENIKVTGGPLPFSFDILTPNCXSV
AITKYTSGIPDYFKQSFPEGFTWERTTIYEDGAYLTTQQETKLDGNCLVYNIKILGCNFP
PNGPVXQKKTGWEPCCEXRYTRDGVLCGQTLXALKCADGNHLTCHLRTTYRSKKAAKPPF
HFSDHRPEIVKVSGTLFEQHESSVARYCQT
>gi|117413686|emb|CAL49526.1||Epiactis_japonica|Cnidarian-Anthozoa
ISDNVRIKLYMEGTVNNHHFMCEAEGEGKPYEGTQMENIKVTGGPLPFSFDILTPNCGSV
AITKYTSGIPDYFKQSFPEGFTWERTTIYEDGAYLTTQQETKLDGNCLVYNIKILGCNFP
PNGPVMQKKTGWEPCCEMRYTRDGVLCGQTLMALKCADGNHLTCHLRTTYRSKKAAKPPF
HFSDHRPEIVKVSGTLFEQHESSVARYCQT
>gi|117413688|emb|CAL49527.1||Epiactis_japonica|Cnidarian-Anthozoa
ISDNVRIKLYMEGTVNNHHFMCEAEGEGKPYEGTQMENIKVTGGPLPFSFDILTPNCGSV
AITKYTSGIPDYFKQSFPEGFTWERTTIYEDGAYLTTQQETKLDGNCLVYNIKILGCNFP
PNGPVMQKKTGWEPCCEMRYTRDGVLCGQTLMALKCADGNHLTCHLRTTYRSKKAAKPPF
HFSDHRPEIVKVSGTLFEQHESSVARYCQT
>gi|159795488|pdb|2RH7|A|Renilla_reniformis|Cnidarian-Anthozoa
LKEVMPTKINLEGLVGDHAFSMEGVGEGNILEGTQEVKISVTGAPLPFAFDIVSVAFXNR
AYTGYPEEISDYFLQSFPEGFTYERNIRYQDGGTAIVKSDISLEDGKFIVNVDFKAKDLR
RMGPVMQQDIGMQPSYESMYTNVTSVIGECIIAFKLQTGKHFTYHMRTVYKSKKPVEPLY
HFIQHRLVKTNVDSGYVVQHETAIAAHSTI
>gi|14161475|gb|AAK54757.1|AF372525_1|Renilla_reniformis|Cnidarian-Anthozoa
LKEVMPTKINLEGLVGDHAFSMEGVGEGNILEGTQEVKISVTGAPLPFAFDIVSVAFGNR
AYTGYPEEISDYFLQSFPEGFTYERNIRYQDGGTAIVKSDISLEDGKFIVNVDFKAKDLR
RMGPVMQQDIGMQPSYESMYTNVTSVIGECIIAFKLQTGKHFTYHMRTVYKSKKPVEPLY
HFIQHRLVKTNVDSGYVVQHETAIAAHSTI
>gi|471179767|gb|AGI04885.1||Helioseris_cucullata|Cnidarian-Anthozoa
---LPFSFDLLTPAFGNR
AFCEYPADIQDFFKQSFPEGYSWERNITFEDQAACKITSKI-------------------

>gi|471179863|gb|AGI04932.1||Helioseris_cucullata|Cnidarian-Anthozoa
---LPFSFDXLTPAFGNR
AFCEYPADIQDFFKQSFPEGYSWERNITFEDQAACKITSKI-------------------

>gi|471179853|gb|AGI04927.1||Helioseris_cucullata|Cnidarian-Anthozoa
---LPFSFXXXXXXFGNR
AFXXXPADIQDFFKQSFPEGYSWERNITFEDQAACKITSKI-------------------

>gi|471179857|gb|AGI04929.1||Helioseris_cucullata|Cnidarian-Anthozoa
---LPFSFXXXXXAFGNR
AFXXXPADIQDFFKQSFPEGYSWERNITFEDQAACKITSKI-------------------

>gi|471179859|gb|AGI04930.1||Helioseris_cucullata|Cnidarian-Anthozoa
--LPFSFXXXXXXFGNR
AFXXXPADIQDFFKQSFPEGYSWERNITFEDQAACKITSKI-------------------

>gi|635576945|pdb|4OHS|A|Actinia_equina|Cnidarian-Anthozoa
VTEDMCIKMTMEGTINGHHFKCVGEGEGKPFEGTQVEKIRITGGPLPFAYDILAPCCXSK
TFIKHVSGIPDYFKESFPEGFTWERTQIFEDGGSLTIHQDTSLQGNNFIFKVNVIGANFP
ANGPVMQKKTGWEPSVEILYPRDGVLCGQALMALKCTDGDHLTSHLRTTYRSRKPSNPEF
HFGDHRIEILKAEGKFYEQYESAVARYCE-
>gi|635576948|pdb|4OHS|D|Actinia_equina|Cnidarian-Anthozoa
VTEDMCIKMTMEGTINGHHFKCVGEGEGKPFEGTQVEKIRITGGPLPFAYDILAPCCXSK
TFIKHVSGIPDYFKESFPEGFTWERTQIFEDGGSLTIHQDTSLQGNNFIFKVNVIGANFP
ANGPVMQKKTGWEPSVEILYPRDGVLCGQALMALKCTDGDHLTSHLRTTYRSRKPSNPEF
HFGDHRIEILKAEGKFYEQYESAVARYCE-
>gi|635576951|pdb|4OHS|G|Actinia_equina|Cnidarian-Anthozoa
VTEDMCIKMTMEGTINGHHFKCVGEGEGKPFEGTQVEKIRITGGPLPFAYDILAPCCXSK
TFIKHVSGIPDYFKESFPEGFTWERTQIFEDGGSLTIHQDTSLQGNNFIFKVNVIGANFP
ANGPVMQKKTGWEPSVEILYPRDGVLCGQALMALKCTDGDHLTSHLRTTYRSRKPSNPEF
HFGDHRIEILKA-GKFYEQYESAVARYC--
>gi|635576952|pdb|4OHS|H|Actinia_equina|Cnidarian-Anthozoa
VTEDMCIKMTMEGTINGHHFKCVGEGEGKPFEGTQVEKIRITGGPLPFAYDILAPCCXSK
TFIKHVSGIPDYFKESFPEGFTWERTQIFEDGGSLTIHQDTSLQGNNFIFKVNVIGANFP
ANGPVMQKKTGWEPSVEILYPRDGVLCGQALMALKCTDGDHLTSHLRTTYRSRKPSNPEF
HFGDHRIEILKAEGKFYEQYESAVARYC--
>gi|76262441|gb|ABA41382.1||Actinia_equina|Cnidarian-Anthozoa
VKKDMCIKMTMEGTVNGHHFKCVGEGEGKPFEGTQVEKIRITGGPLPFAYDILAPCCGSK
TFIKHVSGIPDYFKESFPEGFTWERTQIFEDGGYLTIHQDTSLQGNNFIFKVNVIGANFP
ANGPVMQKKTGWEPCVEMLYPRDGVLCGQSLMALKCTDGNHLTSHLRTTYRSRKPSNPEF
HFGDHRIEILKAEGKFYEQYESAVARYCEA
>gi|635576946|pdb|4OHS|B|Actinia_equina|Cnidarian-Anthozoa
VTEDMCIKMTMEGTINGHHFKCVGEGEGKPFEGTQVEKIRITGGPLPFAYDILAPCCXSK
TFIKHVSGIPDYFKESFPEGFTWERTQIFEDGGSLTIHQDTSLQGNNFIFKVNVIGANFP
ANGPVMQKKTGWEPSVEILYPRDGVLCGQALMALKCTDGDHLTSHLRTTYRSRKPSNPEF
HFGDHRIEILKAEGKFYEQYESAVARYCEA
>gi|635576947|pdb|4OHS|C|Actinia_equina|Cnidarian-Anthozoa
VTEDMCIKMTMEGTINGHHFKCVGEGEGKPFEGTQVEKIRITGGPLPFAYDILAPCCXSK
TFIKHVSGIPDYFKESFPEGFTWERTQIFEDGGSLTIHQDTSLQGNNFIFKVNVIGANFP
ANGPVMQKKTGWEPSVEILYPRDGVLCGQALMALKCTDGDHLTSHLRTTYRSRKPSNPEF
HFGDHRIEILKAEGKFYEQYESAVARYCEA
>gi|224983429|pdb|3CFA|A|Anemonia_sulcata|Cnidarian-Anthozoa
---XSK
TFIKYVSGIPDYFKQSFPEGFTWERTTTYEDGGFLTAHQDTSLDGDXLVYKVKILGNNFP
ADGPVMQNKAGWEPSCEILYEVDGVLCGQSLMALKCPGGRHLNCRLHTTYRSKKPASPEF
HFEDHRIEVKEVQGKHYEQYEAAVARYXDA
>gi|224983437|pdb|3CFF|A|Anemonia_sulcata|Cnidarian-Anthozoa

---XSK
TFIKYVSGIPDYFKQSFPEGFTWERTTTYEDGGFLTAHQDTSLDGDXLVYKVKILGNNFP
ADGPVMQNKAGWEPGCEILYEVDGVLCGQSLMALKCPGGRHLNCRLHTTYRSKKPASPEF
HFEDHRIEVKEVQGKHYEQYEAAVARYXDA
>gi|66360543|pdb|1XMZ|A|Anemonia_sulcata|Cnidarian-Anthozoa
LKKTMPFKTTIEGTVNGHYFKCTGKGEGNPFEGTQEMKIEVIGGPLPFAFHILSTSCXSK
TFIKYVSGIPDYFKQSFPEGFTWERTTTYEDGGFLTAHQDTSLDGDCLVYKVKILGNNFP
ADGPVMQNKARWEPGTEIVYEVDGVLRGQSLMALKCPGGRHLTCHLHTTYRSKKPASPGF
HFEDHRIEIMEEV-KCYKQYEAAVGRYCDA
>gi|18999346|gb|AAG41206.2|AF322222_1|Anemonia_sulcata|Cnidarian-Anthozoa
LKKTMPFKTTIEGTVNGHYFKCTGKGEGNPFEGTQEMKIEVIGGPLPFAFHILSTSCGSK
TFIKYVSGIPDYFKQSFPEGFTWERTTTYEDGGFLTAHQDTSLDGDCLVYKVKILGNNFP
ADGPVMQNKARWEPATEILYEVDGVLRGQSLMALKCPGGRHLTCHLHSTYRSKKPASPGF
HFGDHRIEIMEEV-KCYKQYEAAVARYCDA
>gi|56967219|pdb|1XQM|A|Anemonia_sulcata|Cnidarian-Anthozoa
LTETMPFKTTIEGTVNGHCFKCIGKGEGNPFEGTQEMKIEVIGGPLPFAFHILSTSCXSK
TFIKYVSGIPDYFKQSFPEGFTWERTTTYEDGGFLTAHQDTSLDGDCLVYKVKILGNNFP
ADGPVMQNKVRWEPGTEIVYEVDGVLRGQSLMALKCPGGRHLTCHLHTTYRSKKPASPGF
HFEDHRIEIMEEV-KCYKQYEAAVGRYCDA
>gi|57012972|sp|Q9GZ28.1|NFCP_ANESU|Anemonia_sulcata|Cnidarian-Anthozoa
LKKTMPFKTTIEGTVNGHYFKCTGKGEGNPFEGTQEMKIEVIGGPLPFAFHILSTSCGSK
TFIKYVSGIPDYFKQSFPEGFTWERTTTYEDGGFLTAHQDTSLDGDCLVYKVKILGNNFP
ADGPVMQNKARWEPATEIVYEVDGVLRGQSLMALKCPGGRHLTCHLHTTYRSKKPASPGF
HFEDHRIEIMEEV-KCYKQYEAAVGRYCDA
>gi|75766027|pdb|2A50|B|Anemonia_sulcata|Cnidarian-Anthozoa
---XSK
TFIKYVSGIPDYFKQSFPEGFTWERTTTYEDGGFLTAHQDTSLDGDCLVYKVKILGNNFP
ADGPVMQNKARWEPATEIVYEVDGVLRGQSLMALKCPGGRHLTCHLHTTYRSKKPASPGF
HFEDHRIEIMEEV-KCYKQYEAAVGRYCDA
>gi|75766031|pdb|2A52|B|Anemonia_sulcata|Cnidarian-Anthozoa
---XSK
TFIKYVSGIPDYFKQSFPEGFTWERTTTYEDGGFLTAHQDTSLDGDCLVYKVKILGNNFP
ADGPVMQNKARWEPATEIVYEVDGVLRGQVLMALKCPGGRHLTCHLHTTYRSKKPASPGF
HFEDHRIEIMEEV-KCYKQYEAAVGRYCDA
>gi|75766035|pdb|2A53|B|Anemonia_sulcata|Cnidarian-Anthozoa
---XSK
TFIKYVSGIPDYFKQSFPEGFTWERTTTYEDGGFLTAHQDTSLDGDCLVYKVKILGNNFP
ADGPVMQNKARWEPSTEIVYEVDGVLRGQSLMALKCPGGRHLTCHLHTTYRSKKPASPGF
HFEDHRIEIMEEV-KCYKQYEAAVGRYCDA
>gi|403071994|pdb|4EDO|A|Entacmaea_quadricolor|Cnidarian-Anthozoa
ISENMHMKLYMEGTVNGHHFKCTSEGEGKPYEGTQTAKIKVVGGPLPFAFDILATSFXSK
TFINHTQGIPDFFKQSFPEGFTWERITTYEDGGVLTATQDTSLQNGCLIYNVKINGVNFP
SNGPVMQKKTGWEASTEMLYPADSGLRGHSQMALKLVGGGYLHCSLKTTYRSKKPAKPGF
YFVDRKLERIKEAETYVEQHEMAVARYCDL
>gi|333944387|pdb|3PJ7|A|Entacmaea_quadricolor|Cnidarian-Anthozoa
ISENMHMKLYMEGTVNDHHFKCTSEGEGKPYEGTQTMKIKVVGGPLPFAFDILATSFXSK

TFINHTQGIPDFFKQSFPEGFTWERITTYEDGGVLTATQDTSLQNGCLIYNVKINGVNFP
SNGPVMQKKTGWEASTEMLYPADSGLRGHSQMALKLVGGGYLHCSLKTTYRSKKPAKPGF
YFVDRKLERIKEAETYVEQHEMAVARYCDL
>gi|403071996|pdb|4EDS|A|Entacmaea_quadricolor|Cnidarian-Anthozoa
ISENMHTKLYMEGTVNGHHFKCTSEGEGKPYEGTQTCKIKVVGGPLPFAFDILATSFXSK
TFINHTQGIPDFFKQSFPEGFTWERITTYEDGGVLTATQDTSLQNGCLIYNVKINGVNFP
SNGPVMQKKTGWEANTEMLYPADSGLRGHNQMALKLVGGGYLHCSLKTTYRSKKPAKPGF
YFVDRKLERIKEAETYVEQHEMAVARYCDL
>gi|194368662|pdb|3BX9|A|Entacmaea_quadricolor|Cnidarian-Anthozoa
ITENMHMKLYMEGTVNNHHFKCTSEGEGKPYEGTQTMRIKVVGGPLPFAFDILATSFXSK
TFINHTQGIPDFFKQSFPEGFTWERVTTYEDGGVLTATQDTSLQDGCLIYNVKIRGVNFP
SNGPVMQKKTGWEASTEMLYPADGGLEGRSDMALKLVGGGHLICNLKTTYRSKKPAKPGV
YYVDRRLERIKEAETYVEQHEVAVARYCDL
>gi|257471886|pdb|3H1O|A|Entacmaea_quadricolor|Cnidarian-Anthozoa
ITENMHMKLYMEGTVNNHHFKCTSEGEGKPYEGTQTQRIKVVGGPLPFAFDILATSFXSH
TFINHTQGIPDFWKQSFPEGFTWERVTTYEDGGVLTATQDTSLQDGCLIYNVKIRGVNFP
SNGPVMQKKTGWEAHTEMLYPADGGLEGRADLALKLVGGGHLICNFKTTYRSKKPAKPGV
YYVDYRLERIKEAETYVEQHEVAVARYCDL
>gi|209870646|pdb|3E5T|A|Entacmaea_quadricolor|Cnidarian-Anthozoa
IKENMRMMVVMEGSVNGYQFKCTGEGDGNPYMGTQTMRIKVVGGPLPFAFDILATSFXSK
TFIKHTKGIPDFFKQSFPEGFTWERVTRYEDGGVFTVMQDTSLEDGCLVYHAKVTGTNFP
SNGAVMQKKTGWEPNTEMLYPADGGLRGYSQMALNVDGGGYLSCSFETTYRSKKTVEPGF
HFVDHRLERLEESEMFVVQHEHAVAKFCDL
>gi|209870647|pdb|3E5V|A|Entacmaea_quadricolor|Cnidarian-Anthozoa
IKENMRMMVVMEGSVNGYQFKCTGEGDGNPYMGTQTMRIKVVGGPLPFAFDILATSFXSK
TFIKHTKGIPDFFKQSFPEGFTWERVTRYEDGGVFTVMQDTSLEDGCLVYHAKVRGVNFP
SNGAVMQKKTGWEPSTEMLYPADGGLRGYSQMALNVDGGGYLSCSFETTYRSKKTVEPGF
HFVDHRLERLEESEMFVVQHEHAVAKFCDL
>gi|209870648|pdb|3E5W|A|Entacmaea_quadricolor|Cnidarian-Anthozoa
IKENMRMMVVMEGSVNGYQFKCTGEGDGNPYMGTQTMRIKVVGGPLPFAFDILATSFXSK
TFIKHTKGIPDFFKQSFPEGFTWERVTRYEDGGVFTVMQDTSLEDGCLVYHAKVTGVNFP
SNGAVMQKKTGWEPSTEMLYPADGGLRGYCQMALNVDGGGYLFCSFETTYRSKKTDEPGF
HFVDHRLERLEESEMFVVQHEHAVAKFCDL
>gi|38493050|pdb|1UIS|A|Entacmaea_quadricolor|Cnidarian-Anthozoa
IKENMRMMVVMEGSVNGYQFKCTGEGDGNPYMGTQTMRIKVVGGPLPFAFDILATSFXSK
TFIKHTKGIPDFFKQSFPEGFTWERVTRYEDGGVFTVMQDTSLEDGCLVYHAKVTGVNFP
SNGAVMQKKTGWEPNTEMLYPADGGLRGYSQMALNVDGGGYLSCSFETTYRSKKTVEPGF
HFVDHRLERLEESEMFVVQHEHAVAKFCDL
>gi|56748804|sp|Q8ISF8.1|RFP_ENTQU|Entacmaea_quadricolor|Cnidarian-Anthozoa
IKENMRMMVVMEGSVNGYQFKCTGEGDGNPYMGTQTMRIKVVGGPLPFAFDILATSFGSK
TFIKHTKGIPDFFKQSFPEGFTWERVTRYEDGGVFTVMQDTSLEDGCLVYHAKVTGVNFP
SNGAVMQKKTGWEPNTEMLYPADGGLRGYSQMALNVDGGGYLSCSFETTYRSKKTVEPGF
HFVDHRLERLEESEMFVVQHEHAVAKFCDL
>gi|333944381|pdb|3PIB|A|Entacmaea_quadricolor|Cnidarian-Anthozoa
IKENMHMKLYMEGTVNNHHFKCTSEGEGKPYEGTQTMKIKVVGGPLPFAFDILATSFXSK
TFINHTQGIPDFFKQSFPEGFTWERITTYEDGGVLTATQDTSLQNGCIIYNVKINGVNFP

SNGSVMQKKTGWEANTEMLYPADGGLRGHSQMALKLVGGGYLHCSFKTTYRSKKPAKPGF
HFVDHRLERIKEAETYVEQHEMAVAKYCDL
>gi|333944391|pdb|3PJB|A|Entacmaea_quadricolor|Cnidarian-Anthozoa
IKENMHMKLYMEGTVNNHHFKCTSEGEGKPYEGTQTMKIKVVGGPLPFAFDILATSFXSK
TFINHTQGIPDFFKQSFPEGFTWERITTYEDGGVLTATQDTSLQNGCIIYNVKINGVNFP
SNGSVMQKKTGWEANTEMLYPADGGLRGHSQMALKLVGGGYLHCSFKTTYRSKKPAKPGF
HFVDHRLERIKEAETYVEQHEMAVAKYCDL
>gi|333944385|pdb|3PJ5|A|Entacmaea_quadricolor|Cnidarian-Anthozoa
ISENMHMKLYMEGTVNDHHFKCTSEGEGKPYEGTQTMKIKVVGGPLPFAFDILATSFXSK
TFINHTQGIPDFFKQSFPEGFTWERITTYEDGGVLTATQDTSLQNGCLIYNVKINGVNFP
SNGPVMQKKTGWEASTEMLYPADSGLRGHSQMALKLVGGGYLHCSLKTTYRSKKPAKPGF
YFVDRKLERIKEAETYVEQHEMAVARYCDL
>gi|340708159|pdb|3RWA|A|Entacmaea_quadricolor|Cnidarian-Anthozoa
ITENMHMKLYMEGTVNNHHFKCTSEGEGKPYEGTQTMRIKVVGGPLPFAFDILATSFXSK
TFINHTQGIPDFFKQSFPEGFTWERVTTYEDGGVLTATQDTSLQDGCLIYNVKIRGVNFP
SNGPVMQKKTGWEASTEMLYPADGGLEGRSDMALKLV-----------------------

>gi|340708167|pdb|3RWT|A|Entacmaea_quadricolor|Cnidarian-Anthozoa
ITENMHMKLYMEGTVNNHHFKCTSEGEGKPYEGTQTMRIKVVGGPLPFAFDILATSFXSK
TFINHTQGIPDFFKQSFPEGFTWERVTTYEDGGVLTATQDTSLQDGCLIYNVKIRGVNFP
SNGPVMQKKTGWEASTEMLYPAD------------------------------------

>gi|407943597|pdb|3U0K|A|Entacmaea_quadricolor|Cnidarian-Anthozoa
IKENMRMKVVLEGSVNGHQFKCTGEGEGNPYMGTQTMRIKVIGGPLPFAFDILATSXXSR
TFIKYPKGIPDFFKQSFPEGFTWERVTRYEDGGVITVMQDTSLEDGCLVYHAQVRGVNFP
SNGAVMQKKTGWEPTRDQLTEEQIAEFKEAFSLFDKDGDGTITTKLGTVMRSLNPT-QMI
NEVDMMARKMKDTEEEIREAGNGAAELRHV
>gi|407943598|pdb|3U0L|A|Entacmaea_quadricolor|Cnidarian-Anthozoa
IKENMRMKVVLEGSVNGHQFKCTGEGEGNPYMGTQTMRIKVIGGPLPFAFDILATSFXSR
TFIKYPKGIPDFFKQSFPEGFTWERVTRYEDGGVITVMQDTSLEDGCLVYHAQVRGVNFP
SNGAVMQKKTGWEPNTEMMYPADGGLRGYTHMALKVDGGGHLSCSFVTTYRSKKTVGPGI
HAVSHRLERLEESEMFVVQREHAVAKFAGL
>gi|281307105|pdb|3IP2|A|Entacmaea_quadricolor|Cnidarian-Anthozoa
IKEDMHMKLYMEGTVNNHHFKCTSEGEGKPYEGTQTGRIKVVGGPLPFAFDILATCFXSK
TFINHTQGIPDFFKQSFPEGFTWERVTTYEDGGVLTATQDTSLQDGCLIYNVKIRGVNFP
SNGPVMQKKTGWEASTEMLYPADGGLEGRCDMALKLVGGGHLICNLKTTYRSKKPAKPGV
YFVDRRLERIKEAETYVEQHEVAVARYCDL
>gi|594149474|pdb|4OQW|A|Entacmaea_quadricolor|Cnidarian-Anthozoa
IKENMHMKLYMEGTVNNHHFKCTTEGEGKPYEGTQTQRIKVVGGPLPFAFDILATCFXSK
TFINHTQGIPDFFKQSFPEGFTWERVTTYEDGGVLTVTQDTSLQDGCLIYNVKLRGVNFP
SNGPVMQKKTGWEATTETLYPADGGLEGRCDMALXLVGGGHLHCNLKTTYRSXKPAKPGV
YFVDRRLERIKEAETYVEQHEVAVARYCDL
>gi|595588492|pdb|4OJ0|A|Entacmaea_quadricolor|Cnidarian-Anthozoa
IKENMPMKLYMEGTVNNHHFKCTTEGEGKPYEGTQTQRIKVVGGPLPFAFDILATCFXSK
TFIKHPKGIPDFFKQSFPEGFTWERVTTYEDGGVLTVTQDTSLQDGCLIYNVKLRGVNFP
SNGPVMQKKTGWEATTETLYPADGGLEGRCDMALKLDGGGHLHCNLKTTYRSKKPAGPGV

YFVDRRLERIKEAETYVEQHEVAEARYCDL
>gi|291291622|pdb|3LF3|A|Discosoma_sp.|Cnidarian-Anthozoa
IKEFMRFKVHVEGSVNGHEFEIEGEGKGRPYEGTQTAKLKVTGGPLPFAWDILSPQXXSR
AYVKHPADIPDYWKLSFPEGFKWERVMNFEDGGVVTVTQDSSLQDGEFIYKVKLRGTNFP
SDGPVMQKKTGWEASTERMYPEDGALKGEIKQRLKLKDGGHYDAEVKTTYKAKKPVQPGA
YNVNIKLDITSHNYTIVEQYERSEGRHSTG
>gi|268612455|pdb|3KCS|A|Discosoma_sp.|Cnidarian-Anthozoa
IKEFMRFKVHMEGSVNGHVFEIEGEGEGRPYEGTQTAKLKVTGGPLPFTWDILSPQ-XSN
AYVKHPADIPDYFKLSFPEGFKWERVMKFEDGGVVTVTQDSSLQDGEFIYKVKLRGTNFP
SDGPVMQKKTGWEALSERMYPEDGALKGEVKPRVKLKDGGHYDAEVKTTYKAKKPVQPGA
YNVNRKLDITSHNYTIVEQYERAEGRHSTG
>gi|290790215|pdb|3LF4|B|Discosoma_sp.|Cnidarian-Anthozoa
---XSR
AYVKHPADIPDYWKLSFPEGFKWERVMNFEDGGVVTVTQDSSLQDGEFIYKVKLRGTNFP
SDGPVMQKKTGWEAISERMYPEDGALKGEIKQRLKLKDGGHYDAEVKTTYKAKKPVQPGA
YNVNIKLDITSHNYTIVEQYERAEGRHSTG
>gi|662702316|pdb|4Q7R|A|Discosoma_sp.|Cnidarian-Anthozoa
IKEFMRFKVHMEGSVNGHEFEIEGEGEGRPYEGFQTVKLKVTGGPLPFAWDILSPQX-SK
AYVKHPADIPDYLKLSFPEGFKWERVMNFEDGGVVTVTQDSSLQDGEFIYKVKLRGTNFP
SDGPVMQKKTGMEASSERMYPEDGALKGEDKLRLKLKDGGHYTSEVKTTYKAKKPVQPGA
YIVDIKLDITSHNYTIVEQYERAEGRHSTG
>gi|662702318|pdb|4Q7T|A|Discosoma_sp.|Cnidarian-Anthozoa
IKEFMRFKVRMEGTVNGHEFEIEGEGEGRPYEGFQTAKLKVTGGPLPFAWDILSPLX-SK
AYVKHPADIPDYFKLSFPEGFKWERVMNYEDGGVVTVTQDSSLQDGEFIYKVKMRGTNFP
SDGPVMQKKTGWEASSERMYPEDGALKGEIRMRLKLKDGGHYTSEVKTTYKAKKSVQPGA
YIVGIKLDITSHNYTIVEQYERAEGRHSTG
>gi|114794214|pdb|2H5O|A|Discosoma_sp.|Cnidarian-Anthozoa
IKEFMRFKVRMEGSVNGHEFEIEGEGEGRPYEGFQTAKLKVTGGPLPFAWDILSPFX-SK
AYVKHPADIPDYFKLSFPEGFKWERVMNFEDGGVVTVTQDSSLQDGEFIYKVKLRGTNFP
SDGPVMQKKTGWEASSERMYPEDGALKGEIKMRLKLKDGGHYTSEVKTTYKAKKPVQPGA
YIVGIKLDITSHNYTIVEQYERAEGRHSTG
>gi|662702320|pdb|4Q7U|A|Discosoma_sp.|Cnidarian-Anthozoa
IKEFMRFKVHMEGTVNGHEFEIEGEGEGHPYEGFQTAKLKVTGGPLPFAWDILSPLX-SK
AYVKHPADIPDYFKLSFPEGFKWERVMNYEDGGVVTVTQDSSLQDGEFIYKVKMRGTNFP
SDGPVMQKKTGWEASSERMYPEDGALKGEIRMRLKLKDGGHYTSEVKTTYKAKKSVLPGA
YIVGIKLDITSHNYTIVEQYERSEARHSTG
>gi|315022044|pdb|3NEZ|A|Discosoma_sp.|Cnidarian-Anthozoa
IKEFMRFKTHMEGSVNGHEFEIEGEGEGRPYEGTQTAKLKVTGGPLPFAWDILSPQFXSK
AYVKHPADIPDYLKLSFPEGFKWERVMNFEDGGVVTVTQDSSLQDGEFIYKVKLHGTNFP
SDGPVMQKKTGWEASSERMYPEDGALKGEIKLRLKLKDGGHYDAEVKTTYKAKKPVQPGA
YNANYKLDITSHNYTIVEQYERCEGRHS--
>gi|315022045|pdb|3NEZ|B|Discosoma_sp.|Cnidarian-Anthozoa
IKEFMRFKTHMEGSVNGHEFEIEGEGEGRPYEGTQTAKLKVTGGPLPFAWDILSPQFXSK
AYVKHPADIPDYLKLSFPEGFKWERVMNFEDGGVVTVTQDSSLQDGEFIYKVKLHGTNFP
SDGPVMQKKTGWEASSERMYPEDGALKGEIKLRLKLKDGGHYDAEVKTTYKAKKPVQPGA
YNANYKLDITSHNYTIVEQYERCEGRHS--

>gi|114794216|pdb|2H5P|A|Discosoma_sp.|Cnidarian-Anthozoa
IKEFMRFKVRMEGSVNGHEFEIEGEGEGRPYEGTQTAKLKVTGGPLPFAWDILTPNFXSK
AYVKHPADIPDYLKLSFPEGFKWERVMNFEDGGVVTVTQDSSLQDGEFIYKVKLRGTMFP
SDGPVMQKKTGWEASSERMYPEDGALKGEIKMRLKLKDGGHYDAEVKTTYKAKKPVQPGA
YIVGIKLDITSHNYTIVELYERAEGRHSTG
>gi|114794217|pdb|2H5Q|A|Discosoma_sp.|Cnidarian-Anthozoa
IKEFMRFKVHMEGSVNGHEFEIEGEGEGRPYEGTQTAKLKVTGGPLPFAWDILSPQFXSK
AYVKHPADIPDYLKLSFPEGFKWERVMNFEDGGVVTVTQDSSLQDGEFIYKVKLRGTNFP
SDGPVMQKKTGWEASSERMYPEDGALKGEIKQRLKLKDGGHYDAEVKTTYKAKKPVQPGA
YNVNIKLDITSHNYTIVEQYERAEGRHSTG
>gi|308387883|pdb|3NF0|A|Discosoma_sp._LW-2004|Cnidarian-Anthozoa
IKEFMRFKEHMEGSVNGHEFEIEGEGEGRPYEGTQTARLKVTGGPLPFAWDILSPQIXSK
AYVKHPADIPDYLKLSFPEGFKWERVMNFEDGGVVTVTQDSSLQDGEFIYKVKVRGTNFP
SDGPVMQKKTGWEASSERMYPEDGALKGEMKMRLRLKDGGHYDAEVKTTYMAKKPVQPGA
YKTDTKLDITSHNYTIVEQYERNEGRHSTG
>gi|409974060|pdb|4H3M|A|Discosoma_sp._LW-2004|Cnidarian-Anthozoa
IKEFMRFKAHMEGSVNGHEFEIEGEGEGRPYEGTQTARLKVTGGPLPFAWDILSPQIXSK
AYVKHPADIPDYLKLSFPEGFKWERVMNFEDGGVVTVTQDSSLQDGEFIYKVKVRGTNFP
SDGPVMQKKTGWEASSERMYPEDGALKGEMKMRLRLKDGGHYDAEVKTTYMAKKPVQPGA
YKADYKLDITSHNYTIVEQYERCEGRHSTG
>gi|409974062|pdb|4H3N|A|Discosoma_sp._LW-2004|Cnidarian-Anthozoa
IKEFMRFKEHMEGSVNGHEFEIEGEGEGRPYEGTQTARLKVTGGPLPFAWDILSPQIXSK
AYVKHPADIPDYLKLSFPEGFKWERVMNFEDGGVVTVTQDSSLQDGEFIYKVKVRGTNFP
SDGPVMQKKTGWEASSERMYPEDGALKGEMKMRLRLKDGGHYDAEVKTTYMAKKPVQPGA
YKADYKLDITSHNYTIVEQYERCEGRHSTG
>gi|410563140|pdb|4H3L|A|Discosoma_sp._LW-2004|Cnidarian-Anthozoa
IKEFMRFKPHMEGSVNGHEFEIEGEGEGRPYEGTQTARLKVTGGPLPFAWDILSPQIXSK
AYVKHPADIPDYLKLSFPEGFKWERVMNFEDGGVVTVTQDSSLQDGEFIYKVKVRGTNFP
SDGPVMQKKTGWEASSERMYPEDGALKGEMKMRLRLKDGGHYDAEVKTTYMAKKPVQPGA
YKTDIKLDITSHNYTIVEQYERAEGRHSTG
>gi|315022058|pdb|3NED|A|Discosoma_sp.|Cnidarian-Anthozoa
IKEFMRFKTHMEGSVNGHEFEIEGEGEGRPYEGTQTAKLKVTGGPLPFAWDILSPQFXSK
AYVKHPADIPDYLKLSFPEGFKWERVMNFEDGGVVTVTQDSSLQDGEFIYKVKLRGTNFP
SDGPVMQKKTGWEACSERMYPEDGALKGEMKMRLKLKDGGHYDAEVKTTYKAKKPVQPGA
YNTNTKLDITSHNYTIVEQYERNEGRHSTG
>gi|55792807|gb|AAV65486.1||Discosoma_sp._LW-2004|Cnidarian-Anthozoa
IKEFMRFKVRMEGSVNGHEFEIEGEGEGRPYEGTQTAKLKVTGGPLPFAWDILSPQCGSK
GYVKHPADIPDYLKLSFPEGFKWERVMNFEDGGVVTVTQDSSLQDGEFIYKVKLRGTNFP
SDGPVMQKKTGWEASSERMYPEDGALKGEMKMRLKLKDGGHYDAEVKTTYMAKKPVQPGA
YKTDIKLDITSHNYTIVEQYERAEGRHSTG
>gi|194368491|pdb|2QLG|A|Discosoma_sp._LW-2004|Cnidarian-Anthozoa
IKEFMRFKEHMEGSVNGHEFEIEGEGEGRPYEGTQTARLKVTGGPLPFAWDILSPQIXSK
AYVKHPADIPDYLKLSFPEGFKWERVMNFEDGGVVTVTQDSSLQDGEFIYKVKVRGTNFP
SDGPVMQKKTGWEASSERMYPEDGALKGEMKMRLRLKDGGHYDAEVKTTYMAKKPVQPGA
YKTDIKLDITSHNYTIVEQYERAEGRHSTG
>gi|194368493|pdb|2QLH|A|Discosoma_sp._LW-2004|Cnidarian-Anthozoa

IKEFMRFKEHMEGSVNGHEFEIEGEGEGRPYEGTQTARLKVTGGPLPFAWDILSPQLXSK
AYVKHPADIPDYLKLSFPEGFKWERVMNFEDGGVVTVTQDSSLQDGEFIYKVKVRGTNFP
SDGPVMQKKTGWEASSERMYPEDGALKGEMKMRLRLKDGGHYDAEVKTTYMAKKPVQPGA
YKTDIKLDITSHNYTIVEQYERAEGRHSTG
>gi|194368495|pdb|2QLI|A|Discosoma_sp._LW-2004|Cnidarian-Anthozoa
IKEFMRFKQHMEGSVNGHEFEIEGEGEGRPYEGTQTARLKVTGGPLPFAWDILSPQIXSK
AYVKHPADIPDYLKLSFPEGFKWERVMNFEDGGVVTVTQDSSLQDGEFIYKVKVRGTNFP
SDGPVMQKKTGWEASSERMYPEDGALKGEMKMRLRLKDGGHYDAEVKTTYMAKKPVQPGA
YKTDIKLDITSHNYTIVEQYERAEGRHSTG
>gi|55792809|gb|AAV65487.1||Discosoma_sp._LW-2004|Cnidarian-Anthozoa
IKEFMRFKEHMEGSVNGHEFEIEGEGEGRPYEGTQTARLKVTGGPLPFAWDILSPQIGSK
AYVKHPADIPDYLKLSFPEGFKWERVMNFEDGGVVTVTQDSSLQDGEFIYKVKVRGTNFP
SDGPVMQKKTGWEASSERMYPEDGALKGEMKMRLRLKDGGHYDAEVKTTYMAKKPVQPGA
YKTDIKLDITSHNYTIVEQYERAEGRHSTG
>gi|160286149|pdb|2VAE|A|Discosoma_sp.|Cnidarian-Anthozoa
IKEFMRFKVRMEGSVNGHEFEIEGEGEGRPYEGTQTAKLKVTGGPLPFAWDILSPQFXSK
VYVKHPADIPDYKKLSFPEGFKWERVMNFEDGGVVTVTQDSSLQDGCFIYKVKFIGVNFP
SDGPVMQKKTGWEPSTERLYPRDGVLKGEIHKALKLKDGGHYLVEFKSIYMAKKPVQPGY
YYVDSKLDITSHNYTIVEQYERAEGRHHLF
>gi|114794265|pdb|2H8Q|A|Discosoma_sp.|Cnidarian-Anthozoa
IKEFMRFKVRMEGTVNGHEFEIEGEGEGRPYEGHNTVKLKVTGGPLPFAWDILSPQFXSK
VYVKHPADIPDYMKLSFPEGFKWERVMNFEDGGVVTVTQDSSLQDGCFIYKVKFIGVNFP
SDGPVMQKKTGWEASTERLYPRDGVLKGEIHKALKLKDGGHYLVEFKSIYMAKKPVQPGY
YYVDSKLDITSHNYTIVEQYERTEGRHHLF
>gi|73535927|pdb|1ZGP|A|Discosoma_sp.|Cnidarian-Anthozoa
IKEFMRFKVRMEGTVNGHEFEIEGEGEGRPYEGHNTVKLKVTGGPLPFAWDILSPQFXSM
VYVKHPADIPDYKKLSFPEGFKWERVMNFEDGGVVTVTQDSSLQDGCFIYKVKFIGVNFP
SDGPVMQKKTGWEASTERLYPRDGVLKGEIHKALKLKDGGHYLVEFKSIYMAKKPVQPGY
YYVDSKLDITSHNYTIVEQYERTEGRHHLF
>gi|10304307|gb|AAG16224.1|AF272711_1|Discosoma_sp._SSAL-2000|Cnidarian-Anthozoa
IKEFMRFKVRMEGTVNGHEFEIKGEGEGRPYEGHCSVKLMVTGGPLPFAFDILSPQFGSK
VYVKHPADIPDYKKLSFPEGFKWERVMNFEDGGVVTVSQDSSLKDGCFIYEVKFIGVNFP
SDGPVMQRRTGWEASSERLYPRDGVLKGDIHMALRLEGGGHYLVEFKSIYMVKPSVQPGY
YYVDSKLDMTSHNYTVVEQYEKTQGRHHPF
>gi|12084491|pdb|1G7K|A|Discosoma_sp.|Cnidarian-Anthozoa
IKEFXRFKVRXEGTVNGHEFEIEGEGEGRPYEGHNTVKLKVTGGPLPFAWDILSPQFXSK
VYVKHPADIPDYKKLSFPEGFKWERVXNFEDGGVVTVTQDSSLQDGCFIYKVKFIGVNFP
SDGPVXQKKTGWEASTERLYPRDGVLKGEIHKALKLKDGGHYLVEFKSIYXAKKPVQPGY
YYVDSKLDITSHNYTIVEQYERTEGRHHLF
>gi|12084722|pdb|1GGX|A|Discosoma_sp.|Cnidarian-Anthozoa
IKEFMRFKVRMEGTVNGHEFEIEGEGEGRPYEGHNTVKLKVTGGPLPFAWDILSPQFXSK
VYVKHPADIPDYKKLSFPEGFKWERVMNFEDGGVVTVTQDSSLQDGCFIYKVKFIGVNFP
SDGPVMQKKTGWEASTERLYPRDGVLKGEIHKALKLKDGGHYLVEFKSIYMAKKPVQPGY
YYVDSKLDITSHNYTIVEQYERTEGRHHLF
>gi|55976617|sp|Q9U6Y8.1|RFP_DISSP|Discosoma_sp.|Cnidarian-Anthozoa

IKEFMRFKVRMEGTVNGHEFEIEGEGEGRPYEGHNTVKLKVTGGPLPFAWDILSPQFGSK
VYVKHPADIPDYKKLSFPEGFKWERVMNFEDGGVVTVTQDSSLQDGCFIYKVKFIGVNFP
SDGPVMQKKTGWEASTERLYPRDGVLKGEIHKALKLKDGGHYLVEFKSIYMAKKPVQPGY
YYVDSKLDITSHNYTIVEQYERTEGRHHLF
>gi|160286147|pdb|2VAD|A|Discosoma_sp.|Cnidarian-Anthozoa
IKEFMQFKVRMEGSVNGHYFEIEGEGEGKPYEGTQTAKLQVTGGPLPFAWDILSPQFXSK
AYVKHPADIPDYMKLSFPEGFTWERSMNFEDGGVVEVQQDSSLQDGTFIYKVKFKGVNFP
ADGPVMQKKTGWEPSTEKLYPQDGVLKGEISHALKLKDGGHYTCDFKTVYKAKKPVQPGN
HYVDSKLDITNHNYTVVEQYEHAEARHSGS
>gi|211938970|pdb|2V4E|A|Discosoma_sp.|Cnidarian-Anthozoa
IKPFMRFKVHMEGSVNGHEFEIEGEGEGKPYEGTQTAKLKVTGGPLPFAWDILSPQFXSK
VYTKHPADIPDYKKLSFPEGFKWERVMNFEDGGVVTVTQDSSLQDGTFIYHVKFIGVNFP
SDGPVMQKKTGWEPSTERLYPRDGVLKGEIHKALKLKGGGHYLCEFKSIYMAKKPVKPGY
YYVDSKLDITSHNYTVVEQYERTEARHHLF
>gi|211938971|pdb|2V4E|B|Discosoma_sp.|Cnidarian-Anthozoa
IKPFMRFKVHMEGSVNGHEFEIEGEGEGKPYEGTQTAKLQVTGGPLPFAWDILSPQFXSK
VYTKHPADIPDYKKLSFPEGFKWERVMNFEDGGVVTVTQDSSLQDGVFIYHVKFIGVNFP
SDGPVMQKKTGWEPSTERLYPRDGVLKGEIHKALKLKGGGHYLCEFKSIYMAKKPVKPGY
YYVDSKLDITSHNYTVVEQYERTEARHHLF
>gi|211938972|pdb|2V4E|C|Discosoma_sp.|Cnidarian-Anthozoa
IKPFMRFKVHMEGSVNGHEFEIEGEGEGKPYEGTQTAKLQVTGGPLPFAWDILSPQFXSK
VYTKHPADIPDYKKLSFPEGFKWERVMNFEDGGVVTVTQDSSLQDGTFIYHVKFIGVNFP
SDGPVMQKKTGWEPSTERLYPRDGVLKGEIHKALKLKGGGHYLCEFKSIYMAKKPVKPGY
YYVDSKLDITSHNYTVVEQYERTEARHHLF
>gi|290790214|pdb|3LF4|A|Discosoma_sp.|Cnidarian-Anthozoa
IKEFMRFKVHMEGSVNGHEFEIEGEGEGRPYEGTQTAKLKVTGGPLPFAWDILSPQF---

>gi|51472045|gb|AAU04443.1||Discosoma_sp._RC-2004|Cnidarian-Anthozoa
IKEFMRFKVRMEGTVNGHEFEIEGEGEGRPYEGHNTVKLKVTGGPLPFAWDILSPQFGSK
VYVKHPADIPDYKKLSFPEGFKWERVMNFEDGGVVTVTQDPSLQDGCFIYKVKFIGVNFP
SDGPVMQKKTGWEASTERLYPRDGVLKGEIHKALKLKDGGHYLVEFKTIYMAKKPVQPGY
YYVDSKLDITSHNYTIVEQYERTEGRHHLF
>gi|33333764|gb|AAQ11987.1||Discosoma_sp._JW-2002|Cnidarian-Anthozoa
IKEFMRFKVRMEGTVNGHEFEIEGEGEGRPYGGHNTVKLKVTGGPLPFAWDILSPQFGSK
VYVKHPADIPDYKKLSFPEGFKWERVMNFEDGGVVTVTQDSSLQDGCFIYKVKFIGVNFP
SDGPVMQKKTGWEASTERLYPRDGVLKGDIHKALKLKDGGHYLVEFKSIYMAKKPVQPGY
YYVDSKLDITSHNYTIVEQYERTEGRHHLF
>gi|51472047|gb|AAU04444.1||Discosoma_sp._RC-2004|Cnidarian-Anthozoa
IKEFMRFKVRMEGTVNGHEFEIEGEGEGRPYEGHNTVKLKVTGGPLPFAWDILSPQFGSK
VYVKHPADIPDYKKLSFPEGFKWERVMNFEDGGVVTVTQDSSLQDGCFIYKVKFIGVNFP
SDGPVMQKKTGWEASTERLYPRDGVLKGEIHKALKLKDGGHYLVEFKTIYMAKKPVQPGY
YYVDSKLDITSHNYTIVEQYERTEGRHHLF
>gi|85002146|gb|ABC68474.1||Discosoma_sp._RC-2004|Cnidarian-Anthozoa
IKEFMRFKVRMEGTVNGHEFEIEGEGEGRPYEGHNTVKLKVTGGPLPFAWDILSPQFGSK

VYVKHPADIPDYKKLSFPEGFKWERVMNFEDGGVVTVTQDPSLQDGCFIYKVKFIGVNFP
SDGPVMQKKTGWEASTERLYPRDGVLKGEIHKALKLKDGGHYLVEFKTIYMAKKPVQPGY
YYVDSKLDITSHNYTIVEQYERTEGRHHLF
>gi|255917793|pdb|2WHS|A|Montipora_sp._20|Cnidarian-Anthozoa
IAKQMTYKVYMSGTVNGHYFEVEGDGKGKPYEGEQTVKLTVTGGPLPFAWDILSPQLXSI
PFTKYPEDIPDYFKQSFPEGYTWERSMNFEDGAVCTVSNDSSIQGNCFIYNVKISGENFP
PNGPVMQKKTGWEPSTERLFARDGMLIGNDYMALKLEGGGHYLCEFKSTYKAKKPVRPGR
HEIDRKLDVTSHNYTSVEQCEIAIARHSLL
>gi|91754017|dbj|BAE93224.1||Montipora_sp._20|Cnidarian-Anthozoa
IAKQMTYKVYMSGTVNGHYFEVEGDGKGKPYEGEQTVKLTVTGGPLPFAWDILSPLFGSI
PFTKYPEDIPDYVKQSFPEGYTWERTMNFEDGAVCTVSNDSSIQGNCFIYNVKISGVNFP
PNGPVMQKKTGWEPSTERLFARDGMLIGNDYMALKLEGGGHYLCEFKSTYKAKKPVRPGY
HYVDRKLDVTSHNYTSVEQCEIAIARHSLL
>gi|257472049|pdb|3IR8|A|Montipora_sp._20|Cnidarian-Anthozoa
IAKQMTYKVYMSGTVNGHYFEVEGDGKGKPYEGEQTVKLTVTGGPLPFAWDILSPQLXSI
PFTKYPEDIPDYFKQSFPEGYTWERSMNFEDGAVCTVSNDSSIQGNCFIYNVKISGENFP
PNGPVMQKKTGWEPSTERLFARDGMLIGNDYMALKLEGGGHYLCEFKSTYKAKKPVRPGR
HEIDRKLDVTSHNYTSVEQCEIAIARHSLL
>gi|91754019|dbj|BAE93225.1||Montipora_sp._20|Cnidarian-Anthozoa
IAKQMTYKVYMSGTVNGHYFEVEGDGKGKPYEGEQTVKLTVTGGPLPFAWDILSPLFGSI
PFTKYPEDIPDYVKQSFPEGYTWERTMNFEDGAVCTVSNDSSIQGNCFIYNVKISGTNFP
PNGPVMQKKTGWEPSTERLFARDGMLIGNDYMALKLEGGGHYLCEFKSTYKAKKPVRPGY
HYIDRKLDVTSHNYTSVEQCEIAIARHSLL
>gi|91754021|dbj|BAE93226.1||Montipora_sp._20|Cnidarian-Anthozoa
IAKQMTYKVYMSGTVNGHYFEVEGDGKGKPYEGEQTVKLTVTGGPLPFAWDILSPQLGSI
PFTKYPEDIPDYFKQSFPEGYTWERSMNFEDGAVCTVSNDSSIQGNCFIYNVKISGENFP
PNGPVMQKKTGWEPSTERLFARDGMLIGNDYMALKLEGGGHYLCEFKSTYKAKKPVRPGR
HEIDRKLDVTSHNYTSVEQCEIAIARHSLL
>gi|95102036|dbj|BAE94262.1||Montipora_sp._20|Cnidarian-Anthozoa
IAKQMTYKVYMSGTVNGHYFEVEGDGKGKPYEGEQTVKLTVTGGPLPFAWDILSPLMGSI
PFTKYPEDIPDYVKQSFPEGYTWERTMNFEDGAVCTVSNDSSIQGNCFIYNVKISGTNFP
PNGPVMQKKTGWEPSTERLFARDGMLIGNDYMALKLEGGGHYLCEFKSTYKAKKPVRPGY
HYVDRKLDVTSHNYTSVEQCEIAIARHSLL
>gi|199581447|gb|ACH89425.1||Acropora_pulchra|Cnidarian-Anthozoa
IAKQMTYKVYMSGTVNGHYFEVEGDGKGKPYEGEQTVRLAVTGGPLPFAWDILSPQCGSI
PFTKYPEDIPDYVKQSFPEGYTWERIMNFEDGAVCTVSNDSSIQGNCFIYHVKFSGLNFP
PNGPVMQKKTGWEPNTERLFARDGMLIGNNFMALKLEGGGHYLCEFKSTYKAKKPVKPGY
HYVDRKLDVTNHNYTSVEQCEISIARKPVV
>gi|383502329|dbj|BAM10197.1||Acropora_digitifera|Cnidarian-Anthozoa
----MTYKVYMSGTVNGHYFEVEGDGKGKPYEGEQTVKLTVTGGPLTFAWDILSPQSGSI
PFTKYPEDIPDYVKQSFPEGYTWERIMNFEDGAVCTVSNDSSIQGNCFIYHVKFSGLNFP
PNGPVMQKKTGWEPNTERLFARDGMLIGNNFMALKLEGGGHYLCEFKSTYKAKKPVKPGY
HYVDRKLDVTNHNYTSVEQCEISIARKPVV
>gi|51593132|gb|AAU06853.1||Acropora_tenuis|Cnidarian-Anthozoa
IAKQMTYKVYMSGTVNGHYFEVEGDGKGKPYEGEQTVKLTVTGGPLPFAWDILSPQFGSI
PFTKYPEDIPDYVKQSFPEGYTWERIMNFEDDAVCTVSNDSSIQGNCFIYHVKFSGVNFP

PNGPVMQKKTGWEPNTERLFARDGMLIGNNFMALKLEGGGHYLCEFKSTYKAKKPVRPGY
HYVDRKLDVTNHNYTSVEQCEISIARKPLV
>gi|51593136|gb|AAU06855.1||Acropora_hyacinthus|Cnidarian-Anthozoa
IATQMTYKVYMSGTVNGHYFEVEGDGKGKPYEGEQTVRLTVTGGPLPFAWDILSPQSGSI
PFTKYPEDIPDYVKQSFPEGYTWERIMNFEDGAVCTVSNDSSIQGNCFIYHVKFSGLNFP
PNGPVMQKKTGWEPNTERLFARDGVLIGNNFMALKLEGGGHYLCEFKSTYKAKKPVKPGY
HFVDRKLDVTNHNYTSVEQREISIARKPVV
>gi|51593138|gb|AAU06856.1||Acropora_aculeus|Cnidarian-Anthozoa
IAKQMTYKVYMSGTVNGHYFEVEGDGKGKPYEGEQTVKLTVTGGPLPFAWDILSPQSGSI
PFTKYPDDIPDYVKQSFPEGYTWERIMNFEDGAVCTVSNDSSIQGNCFIYNVKFSGLNFP
PNGPVMQKKTGWEPNTERLFARDGMLIGNNFMALKLEGGGHYLCEFKSTYKAKKPVRPGY
HYVDRKLDVTNHNYTSVEQCEISIARKPVV
>gi|55976566|sp|Q95P04.1|NFCP_GONTE|Goniopora_tenuidens|Cnidarian-Anthozoa
IAKQMTYKVYMSGTVNGHYFEVQGDGKGKPYEGEQTVKLTVTGGPLPFAWDILSPQSGSI
PFTKYPEDIPDYVKQSFPEGYTWERIMNFEDGAVCTVSNDSSIQGNCFIYNVKFSGLNFP
PNGPVMQKKTGWEPNTERLFARDGMLIGNNFMALKLEGGGHYLCEFKSTYKAKKPVKPGY
HYVDRKLDVTNHNYTSVEQCEISIARKPVV
>gi|78059144|gb|ABB17949.1||Goniopora_djiboutiensis|Cnidarian-Anthozoa
IAKQMTYKVYMSGTVNGHYFEVQGDGKGKPYEGEQTVKLTVTGGPLPFAWDILSPQAGSI
PFTKYPEDIPDYVKQSFPEGYTWERIMNFEDGAVCTVSNDSSIQGNCFIYNVKFSGLNFP
PSGPVMQKKTGWEPNTERLLARDGMLIGNNFMALKLEGGGHYLCEFKSTYKAKKPVKPGY
HFVDRKLDVTNHNYTSVEQCEISIARKPVV
>gi|114793409|pdb|2ARL|A|Montipora_efflorescens|Cnidarian-Anthozoa
IATQMTYKVYMSGTVNGHYFEVEGDGKGKPYEGEQTVKLTVTGGPLPFAWDILSPQCXSI
PFTKYPEDIPDYVKQSFPEGFTWERIMNFEDGAVCTVSNDSSIQGNCFTYHVKFSGLNFP
PNGPVMQKKTGWEPSSERLFARGGMLIGNNFMALKLEGGGHYLCEFKTTYKAKKPVKPGY
HYVDRKLDVTNHNYTSVEQCEISIARKPVV
>gi|145580459|pdb|2P4M|A|Montipora_efflorescens|Cnidarian-Anthozoa
IATQMTYKVYMSGTVNGHYFEVEGDGKGKPYEGEQTVKLTVTGGPLPFAWDILSPQCXSI
PFTKYPEDIPDYVKQSFPEGFTWERIMNFEDGAVCTVSNDSSIQGNCFTYHVKFSGLNFP
PNGPVMQKKTGWEPSSERLFARGGMLIGNNFMALKLEGGGHYLCEFKTTYKAKKPVKPGY
HYVDRKLDVTNHNYTSVEQCEISIARKPVV
>gi|30749491|pdb|1MOU|A|Montipora_efflorescens|Cnidarian-Anthozoa
IATQMTYKVYMSGTVNGHYFEVEGDGKGRPYEGEQTVKLTVTGGPLPFAWDILSPQCXSI
PFTKYPEDIPDYVKQSFPEGFTWERIMNFEDGAVCTVSNDSSIQGNCFTYHVKFSGLNFP
PNGPVMQKKTGWEPHSERLFARGGMLIGNNFMALKLEGGGHYLCEFKTTYKAKKPVKPGY
HYVDRKLDVTNHNYTSVEQCEISIARKPVV
>gi|30749492|pdb|1MOV|A|Montipora_efflorescens|Cnidarian-Anthozoa
IATQMTYKVYMSGTVNGHYFEVEGDGKGRPYEGEQTVKLTVTGGPLPFAWDILSPQCXSI
PFTKYPEDIPDYVKQSFPEGFTWERIMNFEDGAVCTVSNDSSIQGNCFTYHVKFSGLNFP
PNGPVMQKKTGWEPSSERLFARGGMLIGNNFMALKLEGGGHYLCEFKTTYKAKKPVKPGY
HYVDRKLDVTNHNYTSVEQCEISIARKPVV
>gi|187564309|gb|ACD13196.1||Echinopora_forskaliana|Cnidarian-Anthozoa
IKQVMKTKLHLEGTVNGHDFTIEGKGEGKPYEGLQHMKMTVTGAPLPFSVHILTPSHGSK
PFNKYPADIPDYHKQSFPEGMSWERSMIFEDGGVCTASNHSSINLQE-IYDVKFHGVNLP
PDGPVMQKTIGWEPSVETLYVRDGMLKSDTAMVFKLKGGGHHRVDFKTTYKAKKPVKPEF

HFVEHRLELTKHDFTTWDQQEAAEGHFSPL
>gi|193506694|pdb|3CGL|A|Discosoma_striata|Cnidarian-Anthozoa
IKEEMLIDLHLEGTFNGHYFEIKGKGKGQPNEGTNTVTLEVTGGPLPFGWHILCPQFXNK
AFVHHPDNIHDYLKLSFPEGYTWERSMHFEDGGLCCITNDISLTGNCFYYDIKFTGLNFP
PNGPVVQKKTGWEPSTERLYPRDGVLIGDIHHALTVEGGGHYACDIKTVYRAKAALKPGY
HYVDTKLVIWNNDFMKVEEHEIAVARHHPF
>gi|56749104|sp|Q9U6Y7.1|GFPL_DISST|Discosoma_striata|Cnidarian-Anthozoa
IKEEMLIDLHLEGTFNGHYFEIKGKGKGQPNEGTNTVTLEVTGGPLPFGWHILCPQFGNK
AFVHHPDNIHDYLKLSFPEGYTWERSMHFEDGGLCCITNDISLTGNCFYYDIKFTGLNFP
PNGPVVQKKTGWEPSTERLYPRDGVLIGDIHHALTVEGGGHYACDIKTVYRAKAALKPGY
HYVDTKLVIWNNDFMKVEEHEIAVARHHPF
>gi|257221227|gb|ACV52379.1||Scleractinia_sp._Lizard_Island_50|Cnidarian-Anthozoa
IAKQMTYKVYMSGTVNGHYFEVEGDGKGKPYEGEQTVXLTVTGGPLPFXWDILSPQTGSI
PFTKYPEDIPDYVKQSFPEGYTWERIMNFEDGAVCTVSNDSSIQGNCFIYNVKISGLNFP
PNGPVMQKKTGWEPNTERLFARDGMLIGNNFMALKLEGGGHYLCEFKSTYKAKKPVRPGY
HYVDRKLDVTSHBYTXVEQCEISIARHSLL
>gi|78059146|gb|ABB17950.1||Montipora_efflorescens|Cnidarian-Anthozoa
IAKQMTYKVYMSGTVNGHYFEVEGDGKGKPYEGEQTVKLTVTGGPLPFAWDILSPLSGSI
PFTKYPEDIPDYVKQSFPEGYTWERIMNFEDGAVCTVSNDSSIQGNCFIYNVKISGVNFP
PNGPVMQKKTGWEPNTERLFARDGMLIGNNFMALKLEGGGYYLCEFKSTYKAKKPVRPGY
HYVDRKLDVTSHNYTFVEQCEISIARHSLL
>gi|78059148|gb|ABB17951.1||Stylocoeniella_sp._NOA-2005|Cnidarian-Anthozoa
IAKQMTYKVYMSGTVNGHYFEVQGDGKGKPYEGEQTVRLTVTGGPLPFAWDILSPLSGSI
PFTKYPEDIPDYVKQSFPEGYTWERIMNFEDGAVCTVSNDSSIQGNCFIYNVKISGLNFP
PNGPVMQKKTGWEPNTERLFARDGMLIGNNFMALKLEGGGHYLCEFKSTYKAKKPVRPGY
HYVDRKLDVTSHNYTSVEQCEISIARHSLL
>gi|383872176|tpd|FAA00744.1||Acropora_digitifera|Cnidarian-Anthozoa
---ILSPQSGSI
PFTKYPEDIPDYVKQSFPEGYTWERIMNFEDGAVCTVSNDSSIQGNCFIYHVKLSGLNFP
PNGPVMQKKTGWEPNTERLFARDGMLIRNSLMALKLEGGGHYLCEFKSTYKAKKPVKPGY
HYVDRKLDVTNHNYTSVEQCEISIARKPVV
>gi|383872178|tpd|FAA00745.1||Acropora_digitifera|Cnidarian-Anthozoa
----
--------------------------MNFEDGAVCTVTNDSSIQGNCFIYHVKFSGLNFP
PNGPVMQKKTGWEPHSERLFARDGMLIGNNFMALKLEGGGHYLCEFKTTYKAKKPVKPGY
HYVDRKLDVTNHNYTSVEQREISIARKPLV
>gi|390136286|pdb|3VIC|A|Montipora_efflorescens|Cnidarian-Anthozoa
IATQMTYKVYMSGTVNGHYFEVEGDGKGRPYEGEQTVKLTVTGGPLPFAWDILSPQCXSI
PFTKYPEDIPDYVKQSFPEGFTWERIMNFEDGAVCTVSNDSSIQGNCFTYHVKFSGLNFP
PNGPVMQKKTGWEPHSERLFARGGMLIGNNFMALKLEGGGHYLCEFKTTYKAKKPVKPGY
HYVDRKLDVTNHNYTSVEQCEISIARKPVV
>gi|390136294|pdb|3VK1|A|Montipora_efflorescens|Cnidarian-Anthozoa
IATQMTYKVYMSGTVNGHYFEVEGDGKGRPYEGEQTVKLTVTGGPLPFAWDILSPQCXSI
PFTKYPEDIPDYVKQSFPEGFTWERIMNFEDGAVCTVSNDSSIQGNCFTYHVKFSGLNFP
PNGPVMQKKTGWEPSSERLFARGGMLIGNNFMALKLEGGGHYLCEFKTTYKAKKPVKPGY
HYVDRKLDVTNHNYTSVEQCEISIARKPVV

>gi|469473782|gb|AGH32876.1||Acropora_millepora|Cnidarian-Anthozoa
IAKQMTYKAYMSGTVNGHYFEVEGDGKGKPYEGEQTEKLTVTGGPLPFAWDILSPQCGSI
PFTKYPEDIPDYVKQSFPEGFTWERIMNFEDGAVCTVTNDSSIQGNCFIYHVKFSGLNFP
PNGPVMQKKTGWEPHSERLLARDGMLIGNNFMALKLEGGGHYLCEFKTTYKAKKPVKPGY
HYVDRKLDVTNHNYTSVEQREISIARKPVV
>gi|469473784|gb|AGH32877.1||Acropora_millepora|Cnidarian-Anthozoa
IAKQMTYKVYMSGTVNGHYFEVEGDGKGKPYEGEQTVKLTVTGGPLPFAWDILSPQSGSI
PFTKYPEDIPDYVKQSFPEGYTWERIMNFEDGAVCTVSNDSSIQGNCFIYHVKFSGLNFP
PNGPVMQKKTGWEPNTERLFARDGMLIGNNFMALKLEGGGHYLCEFKSTYKAKKPVKPGY
HYVDRKLDVTNHNYTSVEQREISIARKPVV
>gi|469473786|gb|AGH32878.1||Acropora_millepora|Cnidarian-Anthozoa
IAKQMTYKVYVSGTVNGHYFEVEGDGKGKPYEGEQTVRLAVTGGPLPFAWDILSPQCGSI
PFTKYPEDIPDYVKQSFPEGYTWERIMNFEDGAVCTVSNDSSIQGNCFIYHVKFSGLNFP
PNGPVMQKKTGWEPNTERLFARDGMLIGNNFMALKLEGGGHYLCEFKSTYKAKKPVKPGY
HYVDRKLDVTNHNYTSVEQREISIARKPVV
>gi|51593134|gb|AAU06854.1||Acropora_millepora|Cnidarian-Anthozoa
IAKQMTYKVYMSGTVNGHYFEVEGDGKGKPYEGEQTVKLTVTGGPLPFAWDILSPQCGSI
PFTKYPEDIPDYVKQSFPEGYTWERIMNFEDGAVCTVSNDSSIQGNCFIYHVKFSGLNFP
PNGPVMQKKTGWEPNTERLFARDGMLLGNNFMALKLEGGGHYLCEFKTTYKAKKPVKPGY
HYVDRKLDVTNHNYTSVEQCEISIARKPVV
>gi|55976263|sp|P83690.2|NFCP_MONEF|Montipora_efflorescens|Cnidarian-Anthozoa
IATQMTYKVYMSGTVNGHYFEVEGDGKGRPYEGEQTVKLTVTGGPLPFAWDILSPQCGSI
PFTKYPEDIPDYVKQSFPEGFTWERIMNFEDGAVCTVSNDSSIQGNCFTYHVKFSGLNFP
PNGPVMQKKTGWEPHSERLFARGGMLIGNNFMALKLEGGGHYLCEFKTTYKAKKPVKPGY
HYVDRKLDVTNHNYTSVEQCEISIARKPVV
>gi|78059180|gb|ABB17967.1||Galaxea_fascicularis|Cnidarian-Anthozoa
IAKQMTYKVYMSGTVNGHYFEVEGDGKGKPYEGEQTVKLTVTGGPLPFAWDILSPQSGSI
PFTKYPEDIPDYVKQSFPEGYTWERIMNFEDGAVCTVSNDSSIQGNCFIYHVKFSGLNFP
PNGPVMQKKTGWEPNTERLFARDGMLIGNNFMALKLEGGGHYLCEFKSTYKAKKPVKPGY
HYVDRKLDVTNHNYTSVEQCEISIARKSVV
>gi|51472049|gb|AAU04445.1||Discosoma_striata|Cnidarian-Anthozoa
IKEEMLINLHLEGTFNGHYFEIKGKGKGNPNEGTNTVTLEVTGGPLPFGWHILCPQFGNK
AFVHHPDDIPDYLKLSFPEGYTWERSMHFEDGGLCCITNDISLTGNCFNYDIKFTGLNFP
PNGPVVQKKTGWEPSTERLYPRDGVLIGDIHHALTVAGGGHYVCDIKTVYRAKXPVKPGY
HYVDPKLVIRSNDFMKVEEHEIAVARXHPL
>gi|78059152|gb|ABB17953.1||Porites_porites|Cnidarian-Anthozoa
VKDVMNTELHMDGIVNGHPFEIKGKGKGNPYKGVQTMKLTVIGAPLPFSIDILLPQHGSK
PFIKYPESIPDYIKLSFPEGITWERSMTFEDGAVCTVSNDSRLDGDSFIYEVRFLGVNFP
RDGPVMQKKTGWDPSTERLYECGGWQRGDVHMALKLENGGHYTCDFKTTYKSKKGLKPPY
HFVDHKLDLLSHNFEEFEQREIAHAHLSNL
>gi|78059174|gb|ABB17964.1||Porites_porites|Cnidarian-Anthozoa
LSKQVTMKYHMDGRFEDKEFTIEGEGTGKPYEGKQTVTLWVTGAPLPFSFDILSAVFGNR
AFTDYPKGIVDYFKPSFPEGYSFERTLEFEDGGYCTASADISLDSAS-IHKSSFKGVKFP
DNGPVKQKKTNWEPSIEKMTVRDGILKGDVTMFLSLTDGGNHRCQFSTLYKAKKAVKPES
HYVEHRLVRTDLPNGKVQLEEHAAARLNTV
>gi|237640434|pdb|2ZO6|A|Verrillofungia_concinna|Cnidarian-Anthozoa

IKPEMKMKYFMDGSVNGHEFTVEGEGTGKPYEGKHKITLDVTGGPLPFAFDLLSTVFXNR
CLTKYPDDIPDYFKQCFPGGYSWERKFEFEDGGLAIAKAEISLKGNCFEHKSTIEGT-FP
DSSPIAQNKTGWEPSTEKMTVRDGSMKGDDAAYLKLVGGGNHKCYFTTTYTAKKIPNPQS
HFIGHRISSVVNG-TKIGVMEDAIAHLYPF
>gi|237640435|pdb|2ZO7|A|Verrillofungia_concinna|Cnidarian-Anthozoa
IKPEMKMRYYMDGSVNGHEFTVEGEGTGRPYEGKQKITLDVTGGPLPFAFDLLSTVFXNR
ALTKYPDDIPDYFKQCFPGGYSWERKFEFEDGGLAIAKAEISLKGNCFEHKSTIEGT-FP
DSSPIMQNKTGWEPSTEKMTVRDGSMKGDDASYLKLVGGGNHKCYFTTTYTAKKIPNPGS
HFIGHRISSVVE-GTKIKVMEDAIAHLYPF
>gi|49257061|dbj|BAD24721.1||Verrillofungia_concinna|Cnidarian-Anthozoa
IKPEMKMKYFMDGSVNGHEFTVEGEGTGKPYEGHQEMTLRVTGGPMPFSFDLVSHTFGHR
PFTKYPEEIPDYFKQAFPEGLSWERSLQFEDGGFAAVSAHISLRGNCFEHKSKFVGVNFP
ADGPVMQNQSDWEPSTEKITTCDGVLKGDVTMFLKLAGGGNHKCQFKTTYKAAKILKPQS
HFIGHRLVRKTE-GNITELVEDAVAHC---
>gi|49257059|dbj|BAD24720.1||Verrillofungia_concinna|Cnidarian-Anthozoa
----MKMKYFMDGSVNGHEFTIEGEGTGKPYEGHQEMTLRVTGGPMPFSFDLVSHTFGHR
PFTKYPEEIPDYFKQAFPEGLSWERSLQFEDGGFAAVSAHISLRGNCFEHKSKFVGVNFP
ADGPVMQNQSDWEPSTEKITTYDGVLKGDVTMFLKLEGGGNHKCQFKTTYKAAKVLKPQS
HFIGHRLVRKTK-GNITELVEDAVAHC---
>gi|209870298|pdb|2ZMU|A|Verrillofungia_concinna|Cnidarian-Anthozoa
IKPEMKMRYYMDGSVNGHEFTIEGEGTGRPYEGHQEMTLRVTGGPMPFAFDLVSHV-XHR
PFTKYPEEIPDYFKQAFPEGLSWERSLEFEDGGSASVSAHISLRGNTFYHKSKFTGVNFP
ADGPIMQNQSDWEPSTEKITASDGVLKGDVTMYLKLEGGGNHKCQFKTTYKAAKILKPGS
HYISHRLVRKTE-GNITELVEDAVAHSLEH
>gi|156622646|dbj|BAF76140.1||Verrillofungia_concinna|Cnidarian-Anthozoa
IKPEMKMRYYMDGSVNGHEFTIEGEGTGRPYEGHQEMTLRVTGGPMPFAFDLVSHVFGHR
VFTKYPEEIPDYFKQAFPEGLSWERSLEFEDGGSASVSAHISLRGNTFYHKSKFTGVNFP
ADGPIMQNQSDWEPSTEKITASDGVLKGDVTMYLKLEGGGNHKCQFKTTYKAAEILEPGD
HYIGHRLVRKTE-GNITELVEDAVAHS---
>gi|326327841|pdb|3MGF|A|Verrillofungia_concinna|Cnidarian-Anthozoa
IKPEMKMRYYMDGSVNGHEFTIEGEGTGRPYEGHQEMTLRVTGGPMPFAFDLVSHV-XHR
PFTKYPEEIPDYFKQAFPEGLSWERSLEFEDGGSASVSAHISLRGNTFYHKSKFTGVNFP
ADGPIMQNQSDWEPSTEKITASDGVLKGDVTMYLKLEGGGNHKCQFKTTYKAAKILKPGS
HYISHRLVRKTE-GNITELVEDAVAHS---
>gi|49257063|dbj|BAD24722.1||Verrillofungia_concinna|Cnidarian-Anthozoa
IKPEMKMRYYMDGSVNGHEFTIEGEGTGRPYEGHQEMTLRVTGGPMPFAFDLVSHVFGHR
PFTKYPEEIPDYFKQAFPEGLSWERSLEFEDGGSASVSAHISLRGNTFYHKSKFTGVNFP
ADGPIMQNQSDWEPSTEKITASDGVLKGDVTMYLKLEGGGNHKCQFKTTYKAAKILKPGS
HYISHRLVRKTE-GNITELVEDAVAHS---
>gi|78059160|gb|ABB17957.1||Danafungia_horrida|Cnidarian-Anthozoa
IGKDMKINYFMDGSVNGHEFTVKGEGIGKPYEGHHEMTLRVTGGPLPFSFDLLSHTFGNR
PFTKYPEEIPDYFKQAFPEGLSWERSLQFEDGGFAAVNANISLKGDCFEHNSKFVGVNFP
AEGPVMQNKSDWEPSTEKITVSDGVLKGDVPMFLKLVGGGNHKCQFTTTYKAAKVLDPQS
HFIFHRLVRKTE-GNITKLVEDVEAHN---
>gi|78059188|gb|ABB17971.1||Stylophora_pistillata|Cnidarian-Anthozoa
LADTMKMTWLMEGSVNGHAFTIEGEGTGKPYEGKQSGTFRVTGGPLPFAFDIVAPTLGFK

CFMKYPADIPDYFKLAFPEGLTYDRKIAFEDGGCATATVEMSLKGNTLVHKTNFQGGNFP
IDGPVMQKRTGWEPTSEKMTPCDGIIKGDTIMYLMVEGGKTLKCRYENNYRANKPVLPPS
HFVDLRLT-RTNLGLAFKLEEYAVARVLEV
>gi|70905462|gb|AAZ14788.1||Corynactis_californica|Cnidarian-Anthozoa
VKEDMKMTYHMDGCVNGHYFTIEGEGTGKPFKGQKTLKLRVTGGPLPFAFDILSATFGNR
CFCDYPEDMPDYFKQSLPEGYSWERTMMYEDGACGTASAHISLDKNGFVHNSTFHGVNFP
ANGPVMKKKGNWEPSSEKITACDGILKGDVTMFLVLEGGHRLKCLFQTTYKADKVVKPPN
HIIEHRLVRSED-GDAVQIQEHAVAKYFTV
>gi|114228559|gb|ABI58282.1||Corynactis_californica|Cnidarian-Anthozoa
VKQDMNMTYHMDGCVNGHSFTIEGEGRGNPFKGQQTLKLRVTGGPLPFAFDILSATFGNR
CFCDYPEDMPDYFKQSLPEGYSWERTMMYEDGGCGTASAHISLNKTGFVHKSTFHGVNFP
ANGPVMQRKTNWEPSTEKITACDGTLKGDVTMFLLLGGGYQQKCQFQTTYRTDKTVKPPN
HIIEHRLVRSED-GDAVQIQEHAVAKYFTV
>gi|70905460|gb|AAZ14787.1||Corynactis_californica|Cnidarian-Anthozoa
LPHDVRMRYHMDGCVNGHSFTIEGEGAGKPYEGKKTLKLRVTGGPLPFAFDILSATFGNR
CFCEYPEDMPDYFKQSLPEGYSWERTMMYEDGGCGTSSAHIRLEKNCFVHQSTFLGVNFP
ANGPVMQKKANWEPSSELITPCDGILKGDVTMFLMLEGGHRQKCQFTTSYKASKAVKPPN
HIIEHVVWKGED-SDGFQIKEHAVAKHFTV
>gi|70905464|gb|AAZ14789.1||Corynactis_californica|Cnidarian-Anthozoa
LPRDVKMRFHMDGCVNGHSFTIEGEGTGKPYEGKKTLKLRVTGGPLPFAFDILSATFGNR
CFCDYPEEMPDYFKQSLPEGYSWERTMMYEDGACSTASAHISLDKDCFIHNSTFHGVNFP
ANGPVMQKKANWEPSSELITPCDGILKGDVTMFLLQEGGHRHKCQFTTSYKAHKAVKPPN
HIIEHRLVRKEV-GDAVQIQEHAVAKHFTV
>gi|72256904|gb|AAZ67342.1||Corynactis_californica|Cnidarian-Anthozoa
LPRDVKMRYHMDGCVNGHQFIIEGEGTGKPYEGKKILELRVTGGPLPFAFDILSSVFGNR
CFCEYPEDMPDYFKQSLPEGHSWERTLMFEDGGCGTASAHISLDKNCFVHKSTFHGVNFP
ANGPVMQKKTNWEPSSELITAGDGILKGDVTMFLMLEGGHRLKCQFTTSYKAKKAVKPPN
HIIEHRLVRKEV-ADAVQIQEHAVAKHFIV
>gi|72256906|gb|AAZ67343.1||Corynactis_californica|Cnidarian-Anthozoa
ITQEMKMVYHMDGCVNGHSFTIEGEGTGKPYEGNQTLKLRVTGGPLPFAFDILTATFGNR
CFCEYPEDMPDYYKQSFPEGYSFERTMMFEDGACCTTSVHLSLTKNCFVHNSTFHGVNFP
ANGPVMQKKTNWEPSSEKITPFEGNLKGDVTMFLKLEGGQQHRCQFQTTYKAHKAVKPPN
HIIEHRLVRSQD-GDAVQLKEHAVAKCFTA
>gi|33333766|gb|AAQ11988.1||Entacmaea_quadricolor|Cnidarian-Anthozoa
VNGNLKIKMFMNGSVNKHKFKCEAEGEGNIHSGTHIMKITVTGGPLPFAFDILSRTFGHK
MFLDNSGNIPDFFKQSLKDGYSWERETTYEDGGVLNSKQKHTLKGDCITTYSEVTGT-FP
PNGPVMKKKTGWEPSCEMVIPRDGGLIMYDALKLDDDHGGHLTGNFKATYKSNPNINPKH
HFVENRVEMLEGRGNYVKLHERNKARYSAL
>gi|55976569|sp|Q95W85.1|NFCP_HETCR|Heteractis_crispa|Cnidarian-Anthozoa
LKESMRIKMYMEGTVNGHYFKCEGEGDGNPFTGTQSMRIHVTGAPLPFAFDILAPCCGSR
TFVHHTAEIPDFFKQSFPEGFTWERTTTYEDGGILTAHQDTSLEGNCLIYKVKVLGTNFP
ADGPVMKNKSGWEPCTEVVYPENGVLCGRNVMAVGDRR---LICHLYTSYRSKKAVRPGF
HFTDIRLQMPRKKDEYFELYEASVARYSDL
>gi|67464343|pdb|1YZW|A|Heteractis_crispa|Cnidarian-Anthozoa
LKESMRIKMYMEGTVNGHYFKCEGEGDGNPFAGTQSMRIHVTGAPLPFAFDILAPCCXSR
TFVHHTAEIPDFFKQSFPEGFTWERTTTYEDGGILTAHQDTSLEGNCLIYKVKVHGTNFP

ADGPVMKNKSGWEPSTEVVYPENGVLCGRNVMAVGDRH---LICHHYTSYRSKKAVRPGF
HFTDIRLQMLRKKDEYFELYEASVARYSDL
>gi|42540751|gb|AAS19276.1||Heteractis_magnifica|Cnidarian-Anthozoa
IKETMRSKVYMEGNVNNHAFKCTAEGEGKPYKGSQNLTITVTGGPLPFAFDILSHAFGNK
VFTKYPDDIPDFFKQSLSGGFTWKRVSNYEDGGVLTVTQETSLKGDCIICNIKVHGTNFP
ADGPVMQKRTGWEPSTETVIPRDGEIRMRDPALKLLDNKGHLLCVMETTYKPNKKVNPKL
HFHHLRMEKDSISEKTIKQHEHVVASYFNV
>gi|28627998|gb|AAO16871.1||Heteractis_magnifica|Cnidarian-Anthozoa
IKETMRSKVYMEGNVNNHAFKCTAEGEGKPYKGSQKLTITVTGGPLPFAFDILSHAFGNK
VFTKYPDDIPDFFKQSLSGGFTWKRVSNYEDGGVLTVDQKTSLEGDCIICNIKVHGTNFP
ADGPVMQKQTGWEPSTETVIPRGEGILLRDPALKLRNNKGHLLCVMETTYKPNKRVNPKL
HFHHLRMEKDSISEKTIKQHEDVRASYFNV
>gi|49035249|gb|AAM10626.2|AF420592_1|Heteractis_crispa|Cnidarian-Anthozoa
IKETMQSKVYMEGKVNDHNFKCTAEGKGEPYKGSQSLTITVTGGPLPFAFDILSHAFGNK
VFAKYPKDHPDFFKQSLPEGFTWERVSNYEDGGVLTVKQETSLEGDCIICKIKAHGTNFP
ADGPVMQKRTGWEPSTETVIPRGGGILMRDPALKLLGNKGHLLCVMETTYKSKKKVNPKP
HFHHLRMEKDSVSEKTIEQHENVRASYFN-
>gi|19982644|gb|AAK71342.1||Condylactis_gigantea|Cnidarian-Anthozoa
IKETMRSKVYMEGDVNNHAFKCTAVGEGKPYKGSQDLTITVTGGPLPFAFDILSHAFGNK
VFTDYPDDIPDFFKQSLSDGFTWRRVSXYXXGGVLTVTQDTSLKGDCIICNIKVHGTNFP
ENGPVMQNKTGWEPSTETVIPQDGGIVAARSPALRLRDKGHLICHMETTYKPNKEVKPEL
HFHHLRMEKLSVSGKTIKQHEYVVASYSKV
>gi|156388065|ref|XP_001634522.1||Nematostella_vectensis|Cnidarian-Anthozoa
SKDAMKLHLILEGSVNGHCFEIHGEGEGKAFEGEQWSKFTVKGGPLPFSFDLIAPCLGSK
PFVKYPDDMTDFFKAAVEGGLSWERTMSLEDGGFCSVVNTSKLDKDGLHYHMTFQGINLD
PNGPVMKKMTGWLPSVETNIPRGNTLVGDINMLLKVNDGSFLRVKFVTVYRFMKPVPPPH
HFIAFKLTRVENDCNVVLLHEWGKAFSCFL
>gi|156385591|ref|XP_001633713.1||Nematostella_vectensis|Cnidarian-Anthozoa
SKDAMKLHFKMEGSVNGHCFEIQGVGEGKAFDGEHWSKLCVVGKHLPFSFDILMPSMGTK
QFAKYPAGMTDFFKAAVEGGLSWERTMTFEDGGYCTIVNTSELKDGSLHYHTNFHGINLK
PDGPVMQKRTGWLPSVETNIPRRDTLLGDINMLLKVNDGSFLRVQFETVYRFMKPVPPPH
HFMAYRLTRVDNDCDTVIQHEWSEAFSCFL
>gi|156388161|ref|XP_001634570.1||Nematostella_vectensis|Cnidarian-Anthozoa
--MGSK
AFAKYPDGMTDFFKAAFEGGLSWERTKTFEDGGYCT---HSTHQNGSLHYHVTFHGINPK
TDGPVMQKITGWSPSVE---

>gi|156407364|ref|XP_001641514.1||Nematostella_vectensis|Cnidarian-Anthozoa
----MKLHFKLEGSVNGHCFEIQGEGEGKPFEGEQWAKHCVVGKHLPFSLDIIMPNI---
-FAKYPDGMTGFFKAAVVGGLSWERTMAFEDRGYCTAVNTSELKNGRLHYHTTFHGINLN
PEKPLMQKRTGWLPSVETNIPRGDTLVGDINMLLKVNDGSFLRVKFETVYRYV-------
--NLYQKSQVSS------------------
>gi|21303784|gb|AAK71343.1||Condylactis_gigantea|Cnidarian-Anthozoa
LKESMRIKIYMEGTVNGYHFKCEGEGDGNPFEGTQNMRIRVTGAPLPFAFDILSPCCGSK
TFIKHTSGIPDYFKRSFPEGFTWEGTTIYEDGRVLTAHQDTSLEGNCPIYKVKVLGTNFP
AASPVMKKVSGWEPSTEIVYQDNGVLRGRNVMALKVSGRPPLICHLHSTYRSKACALPGF

HFADLRIQMPKKKDEYFELYEASVARYSDV
>gi|55976570|sp|Q95W86.1|NFCP_CONGI|Condylactis_gigantea|Cnidarian-Anthozoa
LKESMRIKIYMEGTVNGYHFKCEGEGDGNPFEGTQNMRIRVTGAPLPFAFDILSPCCGSK
TFIKHTSGIPDYFKQSFPEGFTWERTTIYEDGGVLTAHQDTSLEGNCLIYKVKVLGTNFP
ADGPVMKKISGWEPCTEIVYQDNGVLRGRNVMALKVSGRPPLICHLHSTYRSKACALPGF
HFADLRIQMPKKKDEYFELYEASVARYSDV
>gi|55976568|sp|Q95W11.1|NFCP_CONPS|Condylactis_passiflora|Cnidarian-Anthozoa
LKESMRIKIYMEGTVNGYHFKCEGEGDGNPYEGTQNMRIRVTGAPLPFAFDILSPCCGSK
TFIKHTSGIPDYFKQSFPEGFTWERTTIYEDGGVLTAHQDTSLEGNCLNYKVKVLGTNFP
ADGPVMKNISGWEPCTEIVYQDNGVLRGRNVMALKVSGRPPLICHLHSTYRSKACALPGF
HFADLRIQMPKKKDEYFELYEASVARYSDL
>gi|224983428|pdb|3CFA|L|Anemonia_sulcata|Cnidarian-Anthozoa
LTETMPFRMTMEGTVNGHHFKCTGKGEGNPFEGTQDMKIEVIGGPLPFAFDILSTSC---

>gi|75766026|pdb|2A50|A|Anemonia_sulcata|Cnidarian-Anthozoa
LKKTMPFKTTIEGTVNGHYFKCTGKGEGNPFEGTQEMKIEVIGGPLPFAFHILSTSC---

>gi|78059154|gb|ABB17954.1||Montipora_efflorescens|Cnidarian-Anthozoa
LPSDMKLIYHMDGNVNGHSFVIKGEGEGKPYEGTHTIKLQVVGSPLPFSADILSTVFGNR
CFTKYPPNIVDYFKNSCSGGYKFGRSFLYEDGAVCTASGDITLSADK-EHKSKFLGVNFP
ADGPVMKKETNWEPSCEKMTPNGMTLIGDVTGFLLKEDGKRYKCQFHTFHDAKSKKMPDF
HFVQHKIERKDLPMQTWRLTEHAAACKTCF
>gi|78059176|gb|ABB17965.1||Montipora_millepora|Cnidarian-Anthozoa
LPKQMKLTYHMEGTVNGHFFIIKGEGGGEPYEGTHTIKLQVVGSPLPFSPDILSTVFGNR
CFTKYPPNIVDYFKNSCSGGYTFGRSFLYEDGAVCTASGDITLSSDK-EHKSKFLGVNFP
ADGPVMKKETNWEPSCEKMTPNGMTLIGDVTEFLLKKDGKRYKCQFHTFHDAKSRNMPDF
HFVQHEIERKDLPMQTWQLTEHAAACKNCF
>gi|187564251|gb|ACD13191.1||Psammocora_sp._NA-2008|Cnidarian-Anthozoa
LPNMMTLTYHMEGSVNGHNFEIIGEGTGNPKEGKHTITLQVVGGPLPFSVDILSTVFGNR
CFTKYPPNTVDYFKNSCPPGYTFERSFLYEDGAVCTASGDITLSDDK-HHKSKFFGVNFP
DDGPVMKKKTDWEPSCEKMTPSGKTLKGDVIEFLLLEGGGRYKCQFHTVYRAKEPKRPEF
HFVQHKLTRTDVSKQQWQLTEDAAACESCF
>gi|356467179|gb|AET09720.1||Acropora_millepora|Cnidarian-Anthozoa
LTKDMTMKYRMEGCVDGHKFVITGHGNGSPFEGKQTINLCVVGGPLPFSEDILSAVFGNR
VFTEYPQGMVDFFKNSCPAGYTWQRSLLFEDGAVCTASADITVSVEE-YHESKFQGVNFP
ADGPVMKKMTNWEPCCEKIIPVPRILKGDVAMYLLLKDGGRYRCQFDTVYKAKDPKKPEW
HFIQHKLTREDRSNQKWQLAEHSVASRSAL
>gi|383212105|dbj|BAM08939.1||Acropora_tenuis|Cnidarian-Anthozoa
LTKDMTMKYRMEGCVDGHKFVITGHGNGNPFEGKQTINLCVVGGPLPFSEDILSAVFGNR
VFTEYPQGMVDFFKNSCPAGYTWQRSLLFEDGAVCTASADITVSVEE-YHESKFLGVNVP
ADGPVMIKMTNWEPCCEKIIPVPRILKGDVAMYLLLKDGGRYRCQFNTVYKARDPKKPEW
RFIQHKLTREDRSNQKWQLAEHSVASRSAL

>gi|475653236|gb|AGI60203.1||Acropora_millepora|Cnidarian-Anthozoa
LTRDMTMKYRMEGCVDGHKFVITGHGNGSPFEGKQTINLCVVGGPLPFSEDILSAVFGNR
VFTEYPQGMVDFFKNSCPAGYTWQRSLLFEDGAVCTASADITVSVEE-YHESKFHGVNFP
ADGPVMKKMTNWEPCCEKIIPVPRILKGDVPMYLLLKDGGRYRCQFDSVYKAKDPKKPEW
HFIQHKLTREDRSNQKWQLAEHSVASRSAL
>gi|475653238|gb|AGI60204.1||Acropora_millepora|Cnidarian-Anthozoa
LTKDMTMKYRMEGCVDGHKFVITGHGNGSPFEGKQTINLCVVGGPLPFSEDILSVVFGNR
VFTEYPQGMVDFFKNSCPAGYTWQRSLLFEDGAVCTASADITVSVEE-YHESKFHGVNFP
ADGPVMKKMTNWEPCCEKIIPVPRILKGDVAMYLLLKDGGRYRCQFDTVYKAKDPKKPEW
HFIQHKLTREDRSNQKWQLAEHSVASRSAL
>gi|158428729|pdb|2ICR|A|Zoanthus_sp.|Cnidarian-Anthozoa
LTDDMTMHFRMEGCVDGHKFVIEGNGNGNPFKGKQFINLCVIGGPLPFSEDILSAAFXNR
LFTEYPEGIVDYFKNSCPAGYTWHRSFRFEDGAVCICSADITVNVRE-YHESTFYGVNFP
ADGPVMKKMTNWEPSCEKIIPINSILKGDVSMYLLLKDGGRYRCQFDTIYKAKEPKEPDW
HFIQHKLNREDRSNQKWQLIEHAIASRSAL
>gi|126030402|pdb|2FL1|A|Zoanthus_sp.|Cnidarian-Anthozoa
LTDDMTMHFRMEGCVDGHKFVIEGNGNGNPFKGKQFINLCVIGGPLPFSEDILSAAFXNR
LFTEYPEGIVDYFKNSCPAGYTWHRSFRFEDGAVCICSADITVNVRE-YHESTFYGVNFP
ADGPVMKKMTNWEPSCEKIIPINSILKGDVSMYLLLKDGGRYRCQFDTIYKAKEPKEPDW
HFIQHKLNREDRSNQKWQLIEHAIASRSAL
>gi|19982650|gb|AAL23574.1||Zoanthus_sp._SAL-2001|Cnidarian-Anthozoa
LTDDMTMHFRMEGCVDGHKFVIEGNGNGNPFKGKQFINLCVIGGPLPFSEDILSAAFGNR
LFTEYPEGIVDYFKNSCPAGYTWHRSFRFEDGAVCICSADITVNVRE-YHESTFYGVNFP
ADGPVMKKMTNWEPSCEKIIPINSILKGDVSMYLLLKDGGRYRCQFDTIYKAKEPKEPDW
HFIQHKLNREDRSNQKWQLIEHAIASRSAL
>gi|187372791|gb|ACD03133.1||Zoanthus_sp._EB-2008|Cnidarian-Anthozoa
LTDNMTMKYQMEGCVDGHQFVITGHGKGNPFTGKQTLNLCVDGGPLPFSEDILSAVFGNR
IFADYPQDIDDYFKNSCPAGYTWTRSFLFEDGAVAIASADIRLSVQE-HHVSRFYGVNFP
ADGPVMKKMTDWEPSTEKIIPVPSILKGDASMYLLLKDGGRYRCQFDSVYKAKKPKVPDW
HFIQHKLTREDRSNQKWQLVEHASASRSAL
>gi|187372793|gb|ACD03134.1||Zoanthus_sp._EB-2008|Cnidarian-Anthozoa
LTNDMTMKFRMEGCVDGHQFVITGHGNGNPFTGEQTINLCVDGGPLPFSEDILSAAFGNR
LFTEYPQGIVDYFKNSCPAGYTWQRSFLFEDGAVCIASADISVKENCIHHYSTFYGVNFP
VDGPVMKKVTNWEPSCEKIIPIPSILKGDVSMYLLLKDGGRYRCQFDTIYKAKKPKVPDW
HFIQHKLTREDRSNQKWQLVEHAVASRSAL
>gi|197253917|gb|ACH53607.1||Acropora_millepora|Cnidarian-Anthozoa
---GNR
VFTEYPQGMVDFFKNSCPAGYTWHRSLLFEDGAVCTTSADITVSVEE-YHNSKFHGVNFP
ADGPVMKKMTNWEPSCEKIIPVPRILKGDIAMYLLLKDGGRYRCQFDTIYKAKDPKEPEW
HFIQHKLTREDRSNQKWQLVEHAVASRSAL
>gi|199581455|gb|ACH89429.1||Acropora_millepora|Cnidarian-Anthozoa
LTKDMTMKYHMEGSVDGHKFVITGHGNGNPFEGKQTVNLCVAGGPLPFSEDILSAAFGNR
VFTEYPQGMVDFFKNSCPAGYTWHRSLLFEDGAVCTTSADITVSVEE-YHNSKFHGVNFP
ADGPVMKKMTNWEPSCEKIIPVPRILKGDIAMYLLLKDGGRYRCQFDTIYKAKDPKEPEW
HFIQHKLTREDRSNQKWQLVEHAVASRSAL
>gi|475653234|gb|AGI60202.1||Acropora_millepora|Cnidarian-Anthozoa

LTKDMTMKYHMEGSVDGHKFVITGHGNGNPFEGKQTMNLCVAGGPLPFSEDILSAAFGNR
VFTEYPQGMVDFFKNSCPAGYTWHRSLLFEDGAVCTTSADITVSVEE-YHNSKFHGVNFP
ADGPVMKKMTNWEPSCEKIIPVPRILKGDIAMYLLLKDGGRYRCQFDTIYKAKDPKEPEW
HFIQHKLTREDRSNQKWQLVEHAVASRSAL
>gi|50512979|gb|AAT77753.1||Acropora_millepora|Cnidarian-Anthozoa
LTKDMTMKYHMEGSVDGHKFVITGHGDGNPFEGKQTINLCVAGGPLPFSEDILSAAFGNR
VFTEYPQGMVDFFKNSCPAGYTWHRSLLFEDGAVCTTSADITVSVEE-YHNSKFHGVNFP
ADGPVMKKMTNWEPSCEKIIPVPRILKGDIAMYLLLKDGGRYRCQFDTIYKAKDPKEPEW
HFIQHKLTREDRSNQKWQLVEHAVASRSAL
>gi|51593130|gb|AAU06852.1||Acropora_millepora|Cnidarian-Anthozoa
LTKDMTMKYHMEGSVDGHKFVITGHGNGNPFEGKQTMNLCVVGGPLPFSEDILSAAFGNR
VFTEYPQGMVDFFKNSCPAGYTWHRSLLFEDGAVCTTSADITVSVEE-YHNSKFHGVNFP
ADGPVMKKMTNWEPSCEKIIPVPRILKGDIAMYLLLKDGGRYRCQFDTIYKAKDPKEPEW
HFIQHKLTREDRSNQKWQLVEHAVASRSAL
>gi|28628725|gb|AAO49327.1|AF482451_1|Zoanthus_sp._KL-2002|Cnidarian-Anthozoa
LDFDMTMKYRMEGCVDGHKFVITGHGTGNPFAGKQNVNLCVIGGPLPFSEDILSTVFGNR
IFTEYPNSIVDYFKNSCPAGHTWQRSLLFEDGAVCIASADISLSVEE-YHESKFNGVNFP
ADGPVMKKMTNWEPSCEKIIPVPNILKGDVSSYLLLKDGGRYHCQIDSVYKAKEPKKPDR
HFIQHKLTREDRSNQKWQLKERAVASLSAL
>gi|383872180|tpd|FAA00746.1||Acropora_digitifera|Cnidarian-Anthozoa
----------MEGSVDGHKFVITGHGNGNPFEGKQTMNLCVVGGPLPFSEDILSAVFGNR
VFTEYPQGMVDFFKNSCPAGYTWQRSLLFEDGAVCTASADITVSVEE-YHESQFHGVNFP
ADGPVMKKITNWEPSCEKIIPVPRILKGDIAMYLLLKDGGRYRCQFDTVYKANDPKKPGW
HFIQHKLIREDRGNQKWQLVEHAVASRSAL
>gi|56749101|sp|Q9U6Y4.1|GFPL2_ZOASP|Zoanthus_sp.|Cnidarian-Anthozoa
LKEEMTMKYHMEGCVNGHKFVITGEGIGYPFKGKQTINLCVIGGPLPFSEDILSAGFGDR
IFTEYPQDIVDYFKNSCPAGYTWGRSFLFEDGAVCICNVDITVSVKE-YHKSIFNGMNFP
ADGPVMKKMTNWEASCEKIMPVPKILKGDVSMYLLLKDGGRYRCQFDTVYKAKVPSKPEW
HFIQHKLLREDRSNQKWQLTEHAIAFPSAL
>gi|158429229|pdb|2OGR|A|Zoanthus_sp.|Cnidarian-Anthozoa
LKEEMTMKYHMEGCVNGHKFVITGEGIGYPFKGKQTINLCVIGGPLPFSEDILSAG-XDR
IFTEYPQDIVDYFKNSCPAGYTWGRSFLFEDGAVCICNVDITVSVKE-YHKSIFNGMNFP
ADGPVMKKMTNWEASCEKIMPVPKILKGDVSMYLLLKDGGRYRCQFDTVYKAKVPSKPEW
HFIQHKLLREDRSNQKWQLTEHAIAFPSAL
>gi|61680322|pdb|1XA9|A|Zoanthus_sp.|Cnidarian-Anthozoa
LKEEMTMKYHMEGCVNGHKFVITGEGIGYPFKGKQTINLCVIGGPLPFSEDILSAG-XDR
IFTEYPQDIVDYFKNSCPAGYTWGRSFLFEDGAVCICNVDITVSVKE-YHKSIFNGMNFP
ADGPVMKKMTNWEASCEKIMPVPKILKGDVSMYLLLKDGGRYRCQFDTVYKAKVPSKPEW
HFIQHKLLREDRSNQKWQLTEHAIAFPSAL
>gi|61680323|pdb|1XAE|A|Zoanthus_sp.|Cnidarian-Anthozoa
LKEEMTMKYHMEGXVNGHKFVITGEGIGYPFKGKQTINLXVIGGPLPFSEDILSAG-XDR
IFTEYPQDIVDYFKNSCPAGYTWGRSFLFEDGAVCICNVDITVSVKE-YHKSIFNGMNFP
ADGPVMKKMTNWEASCEKIMPVPKILKGDVSMYLLLKDGGRYRCQFDTVYKAKVPSKPEW
HFIQHKLLREDRSNQKWQLTEHAIAFPSAL
>gi|56749102|sp|Q9U6Y5.1|GFPL1_ZOASP|Zoanthus_sp.|Cnidarian-Anthozoa
LTKEMTMKYRMEGCVDGHKFVITGEGIGYPFKGKQAINLCVVGGPLPFAEDILSAAFGNR

VFTEYPQDIVDYFKNSCPAGYTWDRSFLFEDGAVCICNADITVSVEE-YHESKFYGVNFP
ADGPVMKKMTNWEPSCEKIIPVPKILKGDVSMYLLLKDGGRLRCQFDTVYKAKVPRKPDW
HFIQHKLTREDRSNQKWHLTEHAIASGSAL
>gi|158429238|pdb|2OJK|A|Zoanthus_sp.|Cnidarian-Anthozoa
LTKEMTMKYRMEGCVDGHKFVITGEGIGYPFKGKQAINLCVVGGPLPFAEDILSAAFXNR
VFTEYPQDIVDYFKNSCPAGYTWDRSFLFEDGAVCICNADITVSVEE-YHESKFYGVNFP
ADGPVMKKMTNWEPSCEKIIPVPKILKGDVSMYLLLKDGGRLRCQFDTVYKAKVPRKPDW
HFIQHKLTREDRSNQKWHLTEHAIASGSAL
>gi|158429540|pdb|2PXS|A|Zoanthus_sp.|Cnidarian-Anthozoa
LTKEMTMKYRMEGCVDGHKFVITGEGIGYPFKGKQAINLCVVGGPLPFAEDILSAAFXNR
VFTEYPQDIVDYFKNSCPAGYTWDRSFLFEDGAVCICNADITVSVEE-YHESKFYGVNFP
ADGPVMKKMTNWEPSCEKIIPVPKILKGDVSMYLLLKDGGRLRCQFDTVYKAKVPRKPDW
HFIQHKLTREDRSNQKWHLTEHAIASGSAL
>gi|78059150|gb|ABB17952.1||Montipora_efflorescens|Cnidarian-Anthozoa
LTKNMTTKYRMEGCVDGHKFVITGDGIGDPFEGKQTIDLCVVGGPLPFSEDILSAVFGNR
VFTKYPQDLVDYFKNSCPAGYTWQRSFLFEDGAVCTASADITVSVEE-YHESKFHGVNFP
ADGPVMKKMTNWEPSCEKITPIPNILKGDVTMFLLLKDGGRYRCQFDTVYKAKDPK-PDW
HFIQHKLNREDRSHQKWRLVENAIAYRSTL
>gi|78059178|gb|ABB17966.1||Montipora_efflorescens|Cnidarian-Anthozoa
VKDRMKLKFHMEGSVNGHEFTIKGEGTGQPYEGTQSIQLRVEGGPLPFSVDILSAVFGNR
VFTKYPQDLVDYFKNSCPAGYTWQRSFLFEDGAVCTASADITVSVEE-YHESKFHGVNFP
ADGPVMKKMTNWEPSCEKITPIPNILKGDVTMFLLLKDGGRYRCQFDTVYKAKDPK-PDW
HFIQHKLNREDRSHQKWRLVENAIAYRSTL
>gi|21303780|gb|AAK71340.1||Ricordea_florida|Cnidarian-Anthozoa
LQEEMKIKLTMVGVVNGQSFKIDGKGKGKPYEGSQELTLKVVGGPLLFSYDILTTIFGNR
AFVNYPKDIPDIFKQTSGDGYSWQRTMTYEDGGVCTATSNVSVVGDTFNYEIHFMGANFP
PNGPVMQKRTKWEPSXEIXFERDGLLRGDVPMSLLLKGGDHYRCDFKTIYKPNKKVKPGY
HFVDHCIEIKSQEYNMVALFEDAVAHYSPL
>gi|51593114|gb|AAU06844.1||Ricordea_florida|Cnidarian-Anthozoa
LQEEMKIKLTMVGVVNGQSFKIDGKGKGKPYEGSQELTLKVVGGPLLFSYDILTTIFGNR
AFVNYPKDIPDIFKQTSGDGYSWQRTMTYEDGGVCTATSNVSVVGDTFNYEIHFMGANFP
PNGPVMQKRTKWEPSTEIXFERDGLLRGDVPMSLLLKGGDHYRCDFKTIYKPNKKVKPGY
HFVDHCIEIKSQEYNMVALFEDAVAHYSPL
>gi|19982622|gb|AAK71338.1||Ricordea_florida|Cnidarian-Anthozoa
LKEEMKIKLKMVGVVNGQSFQIDGEGKGKPYEGSQKLTLEVVGGPLLFSYDILTTIFGNR
AFVNYPKDIPDIFKQTSGDGFSWQRTMTYEDGGVCTASNHISVDGDTFYYVIRFNGENFP
PNGPVMQKRTKWEPSTEIMFERDGLLRGDIAMSLLLKGGGHYRCDFKTIYTPKRKVNPGY
HFVDHCIEIQKHDYNMAVLSEDAVAHNSPL
>gi|19982635|gb|AAK71339.1||Ricordea_florida|Cnidarian-Anthozoa
LKEEMKIXLTLVGVVNGHPFKIIGDGKGKPYEGSQELTLAVVGGPLPFSYDILTTIVGNR
AFVNYPKDIPDIFKQTSGGGYSWQRTMSFEDGGVCTATSHIRVDGDTFNYDIHFMGADFP
LNGPVMQKRTKWEPSTEIMFQCDGLLRGDVAMSLLLKGGGHYRCDFKTIYKPKKNVKPGY
HFVDHCIEITSQQYNVVELYEGAVAHYSPL
>gi|20086758|gb|AAM10627.1|AF420593_1|Discosoma_sp._SSAL-2002|Cnidarian-Anthozoa
LKEEMKINLTMEGVVNGLPFKIRGDGKGKPYQGSQELTLTVVGGPLPFSYDILTTMFGNR

AFVNYPEDIPDIFKQTSGNGYSWQRTMTYEDGGVCTATSNISVVGDTFNYDIHFMGANFP
LDGPVMQKRTKWEPSTEIMFERDGMLRGDIAMSLLLKGGGHYRCDFETIYKPNKVVKPDY
HFVDHCIEITSQQYNVVELTEVAEARYSSL
>gi|197318592|gb|ACH67606.1||Ceriantharia_sp._JW-2008|Cnidarian-Anthozoa
FPEYLPVTVHMRGNVNNLEFEYDGEGGGDPRAGQFTMNMQLRRKPLPFSYDIITTGFGVR
AFTKYPDSIADYFKGSFPEAFQWNRRIEFEDGGVINMSSDITFKDNRVYGDVWALGVNFP
PNGPVMKNEIMEEPAEETLIPQNGVLVGFCPKAYLLKDGSYYYGKMTTFYRSKSGQAPGF
HFIQHRLVKTKVEFKMVEQSEYATAFVSDL
>gi|257221213|gb|ACV52372.1||Scleractinia_sp._Lizard_Island_28|Cnidarian-Anthozoa
IKPNMKIKLRMEGDVNGHPFVITGEGSGKPYEGTHAIDLKVKGGPLPFAYDILTAAFGNR
AFTKYPADIPDYFKQSFPDGYCWERSMVFEDQGICVVKSVISLDKKEFNYDIRFYGVNFP
ATGPVMKKKTKWEPSTQTMYERDGVLVGDVNMALLLEGGGHHRCDFKSTYRAKVVLNPGN
HYVDHRIEIIHHDYNSVTVHESAEARHCSL
>gi|31616579|gb|AAP55761.1||Cerianthus_sp._DW-2003|Cnidarian-Anthozoa
LSKNVSVSVYMKGNVNNHEFEYDGEGGGDPYTGKYSMKMTLRQNCLPFSYDIITTAFGFR
VFTKYPEGIVDYFKDSLPDAFQWNRRIVFEDGGVLNMSSDITYKDNVLHGDVWAVGVNFP
PNGPVMKNEIMEEPTEETFTPKNGVLVGFCPKAYLLKDGSYYYGNMTTFYRSKSGQAPGY
HFVKHRLVKTNVGFKTVEQTEYATAHVSDL
>gi|471179765|gb|AGI04884.1||Helioseris_cucullata|Cnidarian-Anthozoa
---LPFSFDLLTPAFGNR
VFTKYPADIQDYFKQSFPEGYSWERDVTFEDQAACTVISQI-------------------

>gi|471179855|gb|AGI04928.1||Helioseris_cucullata|Cnidarian-Anthozoa
---LPFSFDXLTPAFGNR
VFTKYPADIQDYFKQSFPEGYSWERDVTFEDQAACTVISQI-------------------

>gi|471179867|gb|AGI04934.1||Helioseris_cucullata|Cnidarian-Anthozoa
---LPFSFXXXTPAFGNR
VFTKYPADIQDYFKQSFPEGYSWERDVTFEDQAACTVISQI-------------------

>gi|537360937|dbj|BAN81727.1||Cavernularia_obesa|Cnidarian-Anthozoa
-TEEMPAQMNLEGVVNGHAFSMEGIGGGNILTGIQKLDIRVIGDPLPFSFDILSVAFGNR
TYTSYPAKIPDYFVQSFPEGFTFERTLSFEDGAIVKVESDISIEDGKFVGKIKYNGESFP
EDGPVMKKEVKLEPSSESMYVCDDTLVGEVVLSYKTQ-STHYTCHMKTIYRSKPVENPKF
HYVHHRLEKKKVEGNYYEQHETAIAKP---
>gi|347582956|gb|AEP13895.1||Abylopsis_eschscholtzi|Cnidarian-Hydrozoa
KEQSVPFKMRIRGTVNGKKVTITGQGSGDARTGKMRGKWVVSPDVCPMSYAALQPTFGYR
VYGQYPDNVIQWFQQCMPEGYTQRRITRFTEEGTLTTFQTIRLEEAMVCTNDTTMEANFR
EGSVLLQAGSSSPSSSSIEKAIPFGLKNYCQFFYPIKDGDVIIATQMTQNHPINKSIPPA
HWKRVECKQFKDPKDHIVQDEI--NKYYEF

Supplementary file 5

Fig. S1. Early stage (about 24 hrs) larvae of *Asymmetron lucayanum* in bright field reflectance, and in fluorescence when excited at 390nm, and at 470nm (from top to bottom). Scale bar: 70 μm (this larval stage is about 250-300 μm in length). Excitation at 355nm also triggers blue fluorescence, but with lesser intensity of fluorescence.

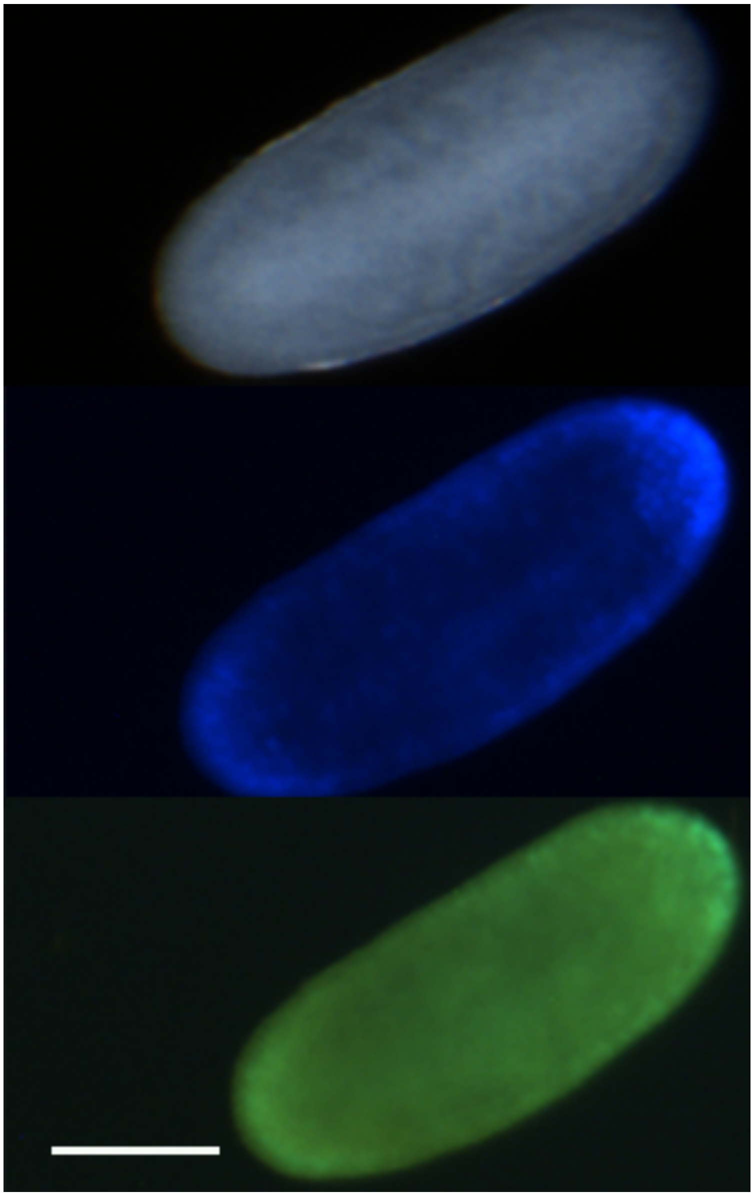

Supplementary file 6

Fig. S2. The full version of the phylogenetic tree of cephalochordate GFP-encoding genes without branch collapsing, with cnidarian and copepod GFP-encoding genes as outgroups. Branches representing the two GFP-encoding genes from *A. lucayanum* are highlighted in red. Branches corresponding to *B. lanceolatum* GFP-encoding sequences were collapsed and the collapsed nodes were represented by triangles in the tree. For each internal node, the local support value was calculated by 100 rapid bootstrapping via RAxML. The clades are highlighted with colors previously designating *B. floridae* GFP clades (following our earlier study[25]).

-----

25 Bomati, E. K., Manning, G. & Deheyn, D. D. Amphioxus encodes the largest known family of green fluorescent proteins, which have diversified into distinct functional classes. *BMC Evol. Biol.* **9**, 1-11, doi:10.1186/1471-2148-9-77 (2009).

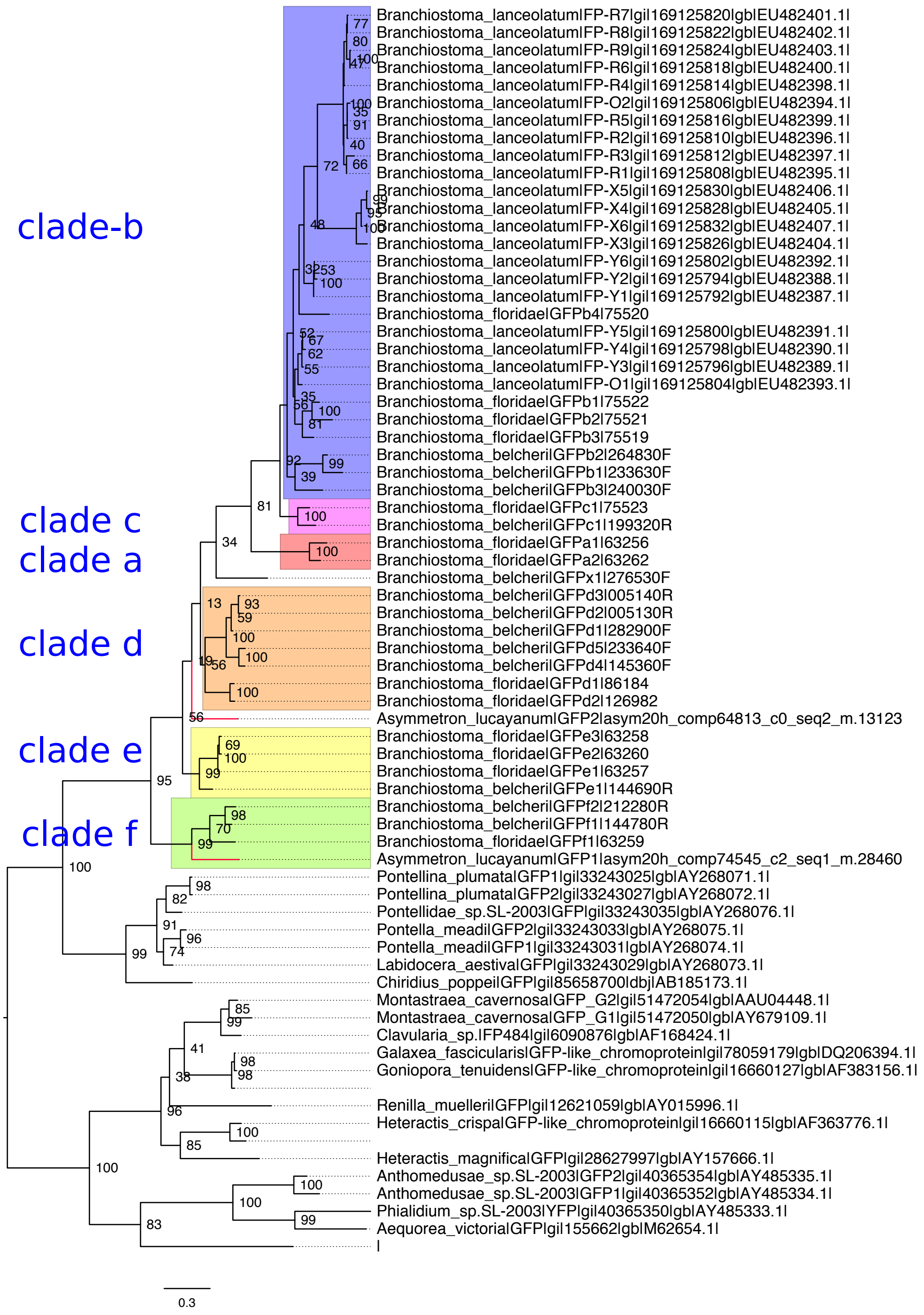
clade-b
clade c
clade a
clade d
clade e
clade f
Branchiostoma_lanceolatum|FP-R7|gi|169125820|gb|EU482401.1|
Branchiostoma_lanceolatum|FP-R8|gi|169125822|gb|EU482402.1|
Branchiostoma_lanceolatum|FP-R9|gi|169125824|gb|EU482403.1|
Branchiostoma_lanceolatum|FP-R6|gi|169125818|gb|EU482400.1|
Branchiostoma_lanceolatum|FP-R4|gi|169125814|gb|EU482398.1|
Branchiostoma_lanceolatum|FP-O2|gi|169125806|gb|EU482394.1|
Branchiostoma_lanceolatum|FP-R5|gi|169125816|gb|EU482399.1|
Branchiostoma_lanceolatum|FP-R2|gi|169125810|gb|EU482396.1|
Branchiostoma_lanceolatum|FP-R3|gi|169125812|gb|EU482397.1|
Branchiostoma_lanceolatum|FP-R1|gi|169125808|gb|EU482395.1|
Branchiostoma_lanceolatum|FP-X5|gi|169125830|gb|EU482406.1|
Branchiostoma_lanceolatum|FP-X4|gi|169125828|gb|EU482405.1|
Branchiostoma_lanceolatum|FP-X6|gi|169125832|gb|EU482407.1|
Branchiostoma_lanceolatum|FP-X3|gi|169125826|gb|EU482404.1|
Branchiostoma_lanceolatum|FP-Y6|gi|169125802|gb|EU482392.1|
Branchiostoma_lanceolatum|FP-Y2|gi|169125794|gb|EU482388.1|
Branchiostoma_lanceolatum|FP-Y1|gi|169125792|gb|EU482387.1|
Branchiostoma_floridae|GFPb4|75520
Branchiostoma_lanceolatum|FP-Y5|gi|169125800|gb|EU482391.1|
Branchiostoma_lanceolatum|FP-Y4|gi|169125798|gb|EU482390.1|
Branchiostoma_lanceolatum|FP-Y3|gi|169125796|gb|EU482389.1|
Branchiostoma_lanceolatum|FP-O1|gi|169125804|gb|EU482393.1|
Branchiostoma_floridae|GFPb1|75522
Branchiostoma_floridae|GFPb2|75521
Branchiostoma_floridae|GFPb3|75519
Branchiostoma_belcheri|GFPb2|264830F
Branchiostoma_belcheri|GFPb1|233630F
Branchiostoma_belcheri|GFPb3|240030F
Branchiostoma_floridae|GFPc1|75523
Branchiostoma_belcheri|GFPc1|199320R
Branchiostoma_floridae|GFPa1|63256
Branchiostoma_floridae|GFPa2|63262
Branchiostoma_belcheri|GFPx1|276530F
Branchiostoma_belcheri|GFPd3|005140R
Branchiostoma_belcheri|GFPd2|005130R
Branchiostoma_belcheri|GFPd1|282900F
Branchiostoma_belcheri|GFPd5|233640F
Branchiostoma_belcheri|GFPd4|145360F
Branchiostoma_floridae|GFPd1|86184
Branchiostoma_floridae|GFPd2|126982
Asymmetron_lucayanum|GFP2|asym20h_comp64813_c0_seq2_m.13123
Branchiostoma_floridae|GFPe3|63258
Branchiostoma_floridae|GFPe2|63260
Branchiostoma_floridae|GFPe1|63257
Branchiostoma_belcheri|GFPe1|144690R
Branchiostoma_belcheri|GFPf2|212280R
Branchiostoma_belcheri|GFPf1|144780R
Branchiostoma_floridae|GFPf1|63259
Asymmetron_lucayanum|GFP1|asym20h_comp74545_c2_seq1_m.28460
Pontellina_plumata|GFP1|gi|33243025|gb|AY268071.1|
Pontellina_plumata|GFP2|gi|33243027|gb|AY268072.1|
Pontellidae_sp.SL-2003|GFP|gi|33243035|gb|AY268076.1|
Pontella_meadi|GFP2|gi|33243033|gb|AY268075.1|
Pontella_meadi|GFP1|gi|33243031|gb|AY268074.1|
Labidocera_aestiva|GFP|gi|33243029|gb|AY268073.1|
Chiridius_poppei|GFP|gi|85658700|dbj|AB185173.1|
Montastraea_cavernosa|GFP_G2|gi|51472054|gb|AAU04448.1|
Montastraea_cavernosa|GFP_G1|gi|51472050|gb|AY679109.1|
Clavularia_sp.|FP484|gi|6090876|gb|AF168424.1|
Galaxea_fascicularis|GFP-like_chromoprotein|gi|78059179|gb|DQ206394.1|
Goniopora_tenuidens|GFP-like_chromoprotein|gi|16660127|gb|AF383156.1|
Renilla_muelleri|GFP|gi|12621059|gb|AY015996.1|
Heteractis_crispa|GFP-like_chromoprotein|gi|16660115|gb|AF363776.1|
Heteractis_magnifica|GFP|gi|28627997|gb|AY157666.1|
Anthomedusae_sp.SL-2003|GFP2|gi|40365354|gb|AY485335.1|
Anthomedusae_sp.SL-2003|GFP1|gi|40365352|gb|AY485334.1|
Phialidium_sp.SL-2003|YFP|gi|40365350|gb|AY485333.1|
Aequorea_victoria|GFP|gi|155662|gb|M62654.1|
0.3

Supplementary file 7

Table S1. Sequence information for all GFP-encoding genes used in Fig. 1 and Fig. S1.

Species name	Gene model ID	Gene name	GenBank accession number
Asymmetron lucayanum	asym20h_comp74545_c2_seq1_m.28460	GFP1	
Asymmetron lucayanum	asym20h_comp64813_c0_seq2_m.13123	GFP2	
Branchiostoma belcheri	005130R	GFPd2	
Branchiostoma belcheri	005140R	GFPd3	
Branchiostoma belcheri	144690R	GFPe1	
Branchiostoma belcheri	144780R	GFPf1	
Branchiostoma belcheri	145360F	GFPd4	
Branchiostoma belcheri	199320R	GFPc1	
Branchiostoma belcheri	212280R	GFPf2	
Branchiostoma belcheri	233630F	GFPb1	
Branchiostoma belcheri	233640F	GFPd5	
Branchiostoma belcheri	240030F	GFPb3	
Branchiostoma belcheri	264830F	GFPb2	
Branchiostoma belcheri	276530F	GFPa1	
Branchiostoma belcheri	282900F	GFPd1	
Branchiostoma floridae	126982	GFPd2	
Branchiostoma floridae	63256	GFPa1	
Branchiostoma floridae	63257	GFPe1	
Branchiostoma floridae	63258	GFPe3	
Branchiostoma floridae	63259	GFPf1	
Branchiostoma floridae	63260	GFPe2	
Branchiostoma floridae	63262	GFPa2	

Branchiostoma floridae	75519	GFPb3	
Branchiostoma floridae	75520	GFPb4	
Branchiostoma floridae	75521	GFPb2	
Branchiostoma floridae	75522	GFPb1	
Branchiostoma floridae	75523	GFPc1	
Branchiostoma floridae	86184	GFPd1	
Branchiostoma lanceolatum		FP-O1	gi|169125804|gb|EU482393.1|
Branchiostoma lanceolatum		FP-O2	gi|169125806|gb|EU482394.1|
Branchiostoma lanceolatum		FP-R1	gi|169125808|gb|EU482395.1|
Branchiostoma lanceolatum		FP-R2	gi|169125810|gb|EU482396.1|
Branchiostoma lanceolatum		FP-R3	gi|169125812|gb|EU482397.1|
Branchiostoma lanceolatum		FP-R4	gi|169125814|gb|EU482398.1|
Branchiostoma lanceolatum		FP-R5	gi|169125816|gb|EU482399.1|
Branchiostoma lanceolatum		FP-R6	gi|169125818|gb|EU482400.1|
Branchiostoma lanceolatum		FP-R7	gi|169125820|gb|EU482401.1|
Branchiostoma lanceolatum		FP-R8	gi|169125822|gb|EU482402.1|
Branchiostoma lanceolatum		FP-R9	gi|169125824|gb|EU482403.1|
Branchiostoma lanceolatum		FP-X3	gi|169125826|gb|EU482404.1|
Branchiostoma lanceolatum		FP-X4	gi|169125828|gb|EU482405.1|
Branchiostoma lanceolatum		FP-X5	gi|169125830|gb|EU482406.1|
Branchiostoma lanceolatum		FP-X6	gi|169125832|gb|EU482407.1|
Branchiostoma lanceolatum		FP-Y1	gi|169125792|gb|EU482387.1|
Branchiostoma lanceolatum		FP-Y2	gi|169125794|gb|EU482388.1|
Branchiostoma lanceolatum		FP-Y3	gi|169125796|gb|EU482389.1|
Branchiostoma lanceolatum		FP-Y4	gi|169125798|gb|EU482390.1|
Branchiostoma lanceolatum		FP-Y5	gi|169125800|gb|EU482391.1|
Branchiostoma lanceolatum		FP-Y6	gi|169125802|gb|EU482392.1|
Aequorea victoria		GFP	gi|155662|gb|M62654.1|

Anthomedusae sp.SL-2003	GFP-like_chromoprotein	gi|40365356|gb|AY485336.1|
Anthomedusae sp.SL-2003	GFP1	gi|40365352|gb|AY485334.1|
Anthomedusae sp.SL-2003	GFP2	gi|40365354|gb|AY485335.1|
Chiridius poppei	GFP	gi|85658700|dbj|AB185173.1|
Clavularia sp.	FP484	gi|6090876|gb|AF168424.1|
Condylactis passiflora	GFP-like_chromoprotein	gi|16660124|gb|AF383155.1|
Galaxea fascicularis	GFP-like_chromoprotein	gi|78059179|gb|DQ206394.1|
Goniopora tenuidens	GFP-like_chromoprotein	gi|16660127|gb|AF383156.1|
Heteractis crispa	GFP-like_chromoprotein	gi|16660115|gb|AF363776.1|
Heteractis magnifica	GFP	gi|28627997|gb|AY157666.1|
Labidocera aestiva	GFP	gi|33243029|gb|AY268073.1|
Montastraea cavernosa	GFP_G1	gi|51472050|gb|AY679109.1|
Montastraea cavernosa	GFP_G2	gi|51472054|gb|AAU04448.1|
Montipora efflorescens	GFP-like_chromoprotein	gi|78059145|gb|DQ206377.1|
Phialidium sp.SL-2003	YFP	gi|40365350|gb|AY485333.1|
Pontella meadi	GFP1	gi|33243031|gb|AY268074.1|
Pontella meadi	GFP2	gi|33243033|gb|AY268075.1|

Pontellidae sp.SL-2003	GFP	gi|33243035|gb|AY268076.1|
Pontellina plumata	GFP1	gi|33243025|gb|AY268071.1|
Pontellina plumata	GFP2	gi|33243027|gb|AY268072.1|
Renilla muelleri	GFP	gi|12621059|gb|AY015996.1|
